# Supplementary material for: Characterization and Physiological Differences of Two Primary Cultures of Human Normal and Hypertrophic Scar Dermal Fibroblasts: A Pilot Study
Source: Biomedicines. 2024 Oct 10;12(10):2295. doi: 10.3390/biomedicines12102295 (PMC11504723; doi:10.3390/biomedicines12102295)
Supplement: Supplementary file 1 [file biomedicines-12-02295-s001.zip › biomedicines-3211266-supplementary.pdf]

SUPPLEMENTARY INFORMATION

Table S1. Phenotype of DFs (6th passage).

Surface Markers	CD105	CD90	CD73	CD44	CD34	HLA-ABC	HLA-DR	SSEA-3
**NDFs**	89.8%	89.8%	91.37%	90.67%	0.25%	56.67%	0.31%	1.75%
**HTSFs**	93.07%	93.24%	92.89%	93.73%	0.13%	47.55%	0.14%	1.56%

Note: The data are presented as a mean value (%) of cell population.

Table S2. Total proteins revealed by mass spectrometric analysis.

Protein Group	Accession	-10LgP	Coverage(%	Coverage(%) sHTSF	Coverage(%) sNDF	Coverage(%) vHTSF	Coverage(%) vNDF	#Peptide	Area sHTSF	Area sNDF	Area vHTSF	Area vNDF	#Uniqu	#Spec sHTSF	#Spec sNDF	#Spec vHTSF	#Spec vNDF	PTM	Average Mas	Description
640	Q8NCU7|C2C	104,0878	5,15	5,15	0	0	0	2	12418,486				1	4				Acetylatio	39743,727	C2 calcium-dependent domain-containing protein 4A OS=Homo sapiens OX=9606 GN=C2CD4A PE=2 SV=2
41	Q9NPA3|M1I	40,03629	3,28	0	0	3,28	0	1					1			1			20201,63	Mid1-interacting protein 1 OS=Homo sapiens OX=9606 GN=MID1IP1 PE=1 SV=1
198	Q9BQE3|TBA	331,6978	24,72	6,68	3,34	24,72	15,37	9	3913,0781		681043,4		2	3	1	13	6	Oxidation	49895,324	Tubulin alpha-1C chain OS=Homo sapiens OX=9606 GN=TUBA1C PE=1 SV=1
519	Q9P219|DAP	135,2232	0,89	0,59	0,3	0,3	0,3	3			100736,945		1	6	7	1	2		228230,3	Protein Daple OS=Homo sapiens OX=9606 GN=CCDC88C PE=1 SV=3
370	O60565|GREI	217,9588	21,74	21,74	21,74	0	15,76	3	1718390,5	1564426,2			3	9	9		3	Deamidat	20696,752	Gremlin-1 OS=Homo sapiens OX=9606 GN=GREM1 PE=1 SV=1
985	Q969V4|TEKT	29,46925	1,44	0	0	1,44	0	1					1			1			48283,098	Tektin-1 OS=Homo sapiens OX=9606 GN=TEKT1 PE=1 SV=1
653	Q8IWY4|SCU	65,07384	0,61	0,61	0	0	0	1	53057,44				1	1					107910,234	Signal peptide, CUB and EGF-like domain-containing protein 1 OS=Homo sapiens OX=9606 GN=SCUBE1 PE=1 SV=3
334	O14495|PLPP	292,7539	18,65	0	0	0	18,65	6				2284528,5	6				13	Carbamid	35116,008	Phospholipid phosphatase 3 OS=Homo sapiens OX=9606 GN=PLPP3 PE=1 SV=1
83	Q9Y4D1|DAA	59,50863	0,56	0	0,56	0	0	1		20622,098			1		1				123473,45	Disheveled-associated activator of morphogenesis 1 OS=Homo sapiens OX=9606 GN=DAAM1 PE=1 SV=2
13	P04150|GCR_	29,92118	0,77	0	0	0,77	0	1			98454,58		1			1			85659,29	Glucocorticoid receptor OS=Homo sapiens OX=9606 GN=NR3C1 PE=1 SV=1
276	Q96AY3|FKB1	311,826	14,09	14,09	0	0	0	9	952419,94				8	15					64244,984	Peptidyl-prolyl cis-trans isomerase FKBP10 OS=Homo sapiens OX=9606 GN=FKBP10 PE=1 SV=1
119	P20930|FILA_	371,6942	2,73	0	0,17	2,22	2,34	21			6471126	3722607,2	20		1	49	27	Deamidat	435169,53	Filaggrin OS=Homo sapiens OX=9606 GN=FLG PE=1 SV=3
9	Q9NY12|GAR	128,4565	3,69	3,69	3,69	3,69	3,69	2	70073,84	124248,49	4028480,5	3482731,8	1	1	3	2	2		22347,873	H/ACA ribonucleoprotein complex subunit 1 OS=Homo sapiens OX=9606 GN=GAR1 PE=1 SV=1
27	Q16363|LAM	536,7838	27,54	22,27	17,99	14,32	19,42	60	9667603	5272266	2656423,2	7402549	57	65	73	42	60	Carbamid	202524,22	Laminin subunit alpha-4 OS=Homo sapiens OX=9606 GN=LAMA4 PE=1 SV=4
7	P19827|ITIH1	114,1269	0,77	0,77	0,77	0,77	0,77	1	314349,75	105456,45	374493,16	2142808	1	1	1	1	2	Oxidation	101389,125	Inter-alpha-trypsin inhibitor heavy chain H1 OS=Homo sapiens OX=9606 GN=ITIH1 PE=1 SV=3
986	Q9BRZ2|TRI5	32,14673	0,79	0,79	0	0	0	1					1	1					81487,7	E3 ubiquitin-protein ligase TRIM56 OS=Homo sapiens OX=9606 GN=TRIM56 PE=1 SV=3
84	Q765P7|MTS	92,82074	0,8	0,8	0	0	0	1	43984,78				1	1					79929,37	Protein MTSS 2 OS=Homo sapiens OX=9606 GN=MTSS2 PE=1 SV=1
377	Q8N7X0|ADG	23,57562	0,36	0	0,36	0	0	1					1		1				189713,1	Androglobin OS=Homo sapiens OX=9606 GN=ADGB PE=2 SV=3
770	Q86WJ1|CHD	42,8563	0,67	0	0	0,67	0	1					1			1			101000,01	Chromodomain-helicase-DNA-binding protein 1-like OS=Homo sapiens OX=9606 GN=CHD1L PE=1 SV=3
987	Q5BKZ1|ZN3	66,54484	1,03	0	1,03	0	1,03	1		24584,418		165526,06	1		1		2		65653,8	DBIRD complex subunit ZNF326 OS=Homo sapiens OX=9606 GN=ZNF326 PE=1 SV=2
988	Q2VIR3|IF2G	29,20635	1,27	0	0	1,27	0	1			88342,03		1			1			51228,59	Eukaryotic translation initiation factor 2 subunit 3B OS=Homo sapiens OX=9606 GN=EIF2S3B PE=2 SV=2
11	O95251|KAT	109,1754	0,98	0,98	0,98	0,98	0,98	1	129407,34	68761,875	195285,42	306537,03	1	2	2	2	2		70642,38	Histone acetyltransferase KAT7 OS=Homo sapiens OX=9606 GN=KAT7 PE=1 SV=1
643	Q9Y6V0|PCL(	44,31478	0,27	0,12	0	0	0,16	2				52218,465	1	1			1	Acetylatio	560699,44	Protein piccolo OS=Homo sapiens OX=9606 GN=PCLO PE=1 SV=5
989	P00747|PLMI	51,69386	0,99	0,99	0	0	0	1					1	1				Carbamid	90568,984	Plasminogen OS=Homo sapiens OX=9606 GN=PLG PE=1 SV=2
849	Q5UAW9|GP	29,93155	1,79	1,79	0	0	0	1	608444,94				1	1					36623,156	G-protein coupled receptor 157 OS=Homo sapiens OX=9606 GN=GPR157 PE=2 SV=2
182	Q3ZCM7|TBB	229,2762	9,91	0	0	9,91	5,41	5			459862,12	176848,97	5			6	4	Oxidation	49776,004	Tubulin beta-8 chain OS=Homo sapiens OX=9606 GN=TUBB8 PE=1 SV=2
174	Q8NFW1|CO	300,2915	5,35	3,51	2,28	3,26	1,91	15	4692515	750517,5	670730,1	339704,66	4	21	12	12	10		161145,33	Collagen alpha-1(XXII) chain OS=Homo sapiens OX=9606 GN=COL22A1 PE=2 SV=2
335	P20742|PZP_	253,4173	3,31	1,96	1,08	3,31	2,9	6	1790091,8	1110366,1	1532804,1	2137783,8	2	10	7	16	12	Oxidation	163862,95	Pregnancy zone protein OS=Homo sapiens OX=9606 GN=PZP PE=1 SV=4
933	Q96JQ5|M4A	24,05968	2,51	0	0	2,51	0	1			27898,795		1			1			25440,834	Membrane-spanning 4-domains subfamily A member 4A OS=Homo sapiens OX=9606 GN=MS4A4A PE=1 SV=1
990	Q9P121|NTRI	32,19333	2,62	2,62	0	0	0	1	48956,63				1	1					37971,473	Neurotrimin OS=Homo sapiens OX=9606 GN=NTM PE=1 SV=1
249	P0CG48|UBC_	312,1252	8,91	8,91	7,59	8,91	8,91	8	7896435,5	3367563,2	1,28E+07	5,91E+07	8	34	29	35	49	Oxidation	77038,61	Polyubiquitin-C OS=Homo sapiens OX=9606 GN=UBC PE=1 SV=3
644	P62491|RB11	71,51145	8,8	0	0	0	8,8	2				152922,4	2				2		24393,518	Ras-related protein Rab-11A OS=Homo sapiens OX=9606 GN=RAB11A PE=1 SV=3
28	Q00341|VIGL	97,37936	0,55	0,55	0,55	0,55	0,55	1	635892	274838,97	445449,97	721488,56	1	3	4	2	2		141455,64	Vigilin OS=Homo sapiens OX=9606 GN=HDLBP PE=1 SV=2
991	Q14699|RFTN	46,8987	1,38	0	0	0	1,38	1					1				1		63145,617	Raftlin OS=Homo sapiens OX=9606 GN=RFTN1 PE=1 SV=4
72	Q8NGA1|OR1	104,1393	2,24	0	0	2,24	2,24	1			294399	841298,3	1			3	5	Oxidation	34839,83	Olfactory receptor 1M1 OS=Homo sapiens OX=9606 GN=OR1M1 PE=2 SV=1
402	P02787|TRFE	243,7829	4,58	4,58	4,58	4,58	2,15	5	6948964	209535,83	208142,08	36526,035	3	20	11	4	2	Carbamid	77049,86	Serotransferrin OS=Homo sapiens OX=9606 GN=TF PE=1 SV=4
150	Q8TEU8|WFK	45,67938	1,04	0	1,04	0	0	1					1		1				63941,062	WAP, Kazal, immunoglobulin, Kunitz and NTR domain-containing protein 2 OS=Homo sapiens OX=9606 GN=WFIKKN2 PE=1 SV=1
250	P27487|DPP4	314,1796	12,01	0	0	10,44	4,05	10			969625,9	1227237,2	10			13	5	Deamidat	88278,63	Dipeptidyl peptidase 4 OS=Homo sapiens OX=9606 GN=DPP4 PE=1 SV=2
646	P31948|STIP1	103,6127	2,21	1,1	0	0	1,1	2				65216,375	1	1			1		62639,25	Stress-induced-phosphoprotein 1 OS=Homo sapiens OX=9606 GN=STIP1 PE=1 SV=1
301	P16234|PGFR	259,1522	6,61	0	0	0	6,61	8				459425,06	6				9	Carbamid	122669,58	Platelet-derived growth factor receptor alpha OS=Homo sapiens OX=9606 GN=PDGFRA PE=1 SV=1
503	O94993|SOX	46,7746	0,8	0	0	0	0,8	1					1				1		81854,22	Transcription factor SOX-30 OS=Homo sapiens OX=9606 GN=SOX30 PE=1 SV=1
992	Q9Y2E5|MA2	26,16275	0,69	0	0,69	0	0	1		21964,492			1		1				113979	Epididymis-specific alpha-mannosidase OS=Homo sapiens OX=9606 GN=MAN2B2 PE=1 SV=4
520	P17931|LEG3	86,86533	8	8	0	0	2,8	3	546084,5			48354,867	3	3			1	Carbamid	26152,33	Galectin-3 OS=Homo sapiens OX=9606 GN=LGALS3 PE=1 SV=5
993	P62753|RS6_	55,05623	4,82	0	0	0	4,82	1				51123,406	1				1		28680,637	40S ribosomal protein S6 OS=Homo sapiens OX=9606 GN=RPS6 PE=1 SV=1
994	A0A075B6P5|	36,45289	5,83	5,83	0	0	0	1	92659,414				1	1					12956,743	Immunoglobulin kappa variable 2-28 OS=Homo sapiens OX=9606 GN=IGKV2-28 PE=3 SV=1
139	Q6NSJ2|PHLI	90,0454	0,94	0	0	0	0,94	1				600038,7	1				2		71911,75	Pleckstrin homology-like domain family B member 3 OS=Homo sapiens OX=9606 GN=PHLDB3 PE=1 SV=3
371	Q15517|CDSI	287,7884	14,93	0	0	14,93	10,4	6			2562001,5	2709296,5	6			13	11	Carbamid	51522,08	Corneodesmosin OS=Homo sapiens OX=9606 GN=CDSN PE=1 SV=3
995	Q6P3W7|SCY	89,21452	1,94	1,94	0	0	0	1					1	4				Deamidat	103708,7	SCY1-like protein 2 OS=Homo sapiens OX=9606 GN=SCYL2 PE=1 SV=1
996	O14924|RGS1	80,99365	0,41	0,41	0,41	0,41	0,41	1	6230665,5	2880302,8	1864814,6	5602360,5	1	1	1	1	2		156357,39	Regulator of G-protein signaling 12 OS=Homo sapiens OX=9606 GN=RGS12 PE=1 SV=1
521	P40189|IL6RE	116,3683	3,27	0,65	0	0,65	3,27	3				42478,84	2	2		1	3	Carbamid	103536,59	Interleukin-6 receptor subunit beta OS=Homo sapiens OX=9606 GN=IL6ST PE=1 SV=2
128	P49221|TGM	28,65675	0,88	0	0	0,88	0	1					1			1			77145,12	Protein-glutamine gamma-glutamyltransferase 4 OS=Homo sapiens OX=9606 GN=TGM4 PE=1 SV=2
522	P22314|UBA1	139,9558	1,98	0,57	0,57	0,85	0,57	3			53854,137	11323,703	2	1	1	1	1		117849	Ubiquitin-like modifier-activating enzyme 1 OS=Homo sapiens OX=9606 GN=UBA1 PE=1 SV=3
997	Q04828|AK1(	35,18394	1,86	0	1,86	0	0	1					1		1				36788,35	Aldo-keto reductase family 1 member C1 OS=Homo sapiens OX=9606 GN=AKR1C1 PE=1 SV=1
998	Q9H444|CHN	57,42603	6,25	0	0	0	6,25	1				15100,43	1				1		24950,15	Charged multivesicular body protein 4b OS=Homo sapiens OX=9606 GN=CHMP4B PE=1 SV=1
143	P81605|DCD_	334,0136	76,36	47,27	63,64	76,36	58,18	15	1105668,5	2807455	1,01E+07	1,92E+07	13	5	17	18	18	Deamidat	11283,857	Dermcidin OS=Homo sapiens OX=9606 GN=DCD PE=1 SV=2
999	Q6IQ55|TTBK	44,83564	0,56	0,56	0,56	0	0,56	1	77391,94	303497,2		229608,75	1	2	6		2		137412,45	Tau-tubulin kinase 2 OS=Homo sapiens OX=9606 GN=TTBK2 PE=1 SV=2
523	P49746|TSP3_	131,2832	1,46	0,73	0,73	0	0,73	2					1	6	1		3	Oxidation	104201,2	Thrombospondin-3 OS=Homo sapiens OX=9606 GN=THBS3 PE=1 SV=1
206	P0DN37|PAL	238,0188	19,51	11,59	11,59	19,51	19,51	5	212291,48	104125,625	406293,38	588672,7	3	3	3	6	5	Carbamid	18165,705	Peptidyl-prolyl cis-trans isomerase A-like 4G OS=Homo sapiens OX=9606 GN=PPIAL4G PE=3 SV=1
388	Q6UWL6|KIR	58,9107	0,85	0,85	0	0	0	1	83421,74				1	1					75093,06	Kin of IRRE-like protein 2 OS=Homo sapiens OX=9606 GN=KIRREL2 PE=1 SV=3

372	Q16881|TRXF	237,381	8,01	7,09	3,54	0	0	6	258621,94	21512,354			6	6	3			Carbamid	70874,93	Thioredoxin reductase 1, cytoplasmic OS=Homo sapiens OX=9606 GN=TXNRD1 PE=1 SV=3
66	Q5T749|KPRF	462,1384	56,48	0	0	56,48	41,28	29			2,39E+07	1,57E+07	28			104	47	Carbamid	64135,734	Keratinocyte proline-rich protein OS=Homo sapiens OX=9606 GN=KPRP PE=1 SV=1
651	O94832|MYO	61,24146	1,59	0	0	1,59	0	2			46257,434		1			2			116202,19	Unconventional myosin-Id OS=Homo sapiens OX=9606 GN=MYO1D PE=1 SV=2
1000	Q6UX72|B3G	22,82203	3,73	3,73	0	0	0	1	137205,6				1	1					43751,26	UDP-GlcNAc:betaGal beta-1,3-N-acetylglucosaminyltransferase 9 OS=Homo sapiens OX=9606 GN=B3GNT9 PE=2 SV=1
652	Q2M2Z5|KIZ_	54,25065	1,78	0	0	0,89	0,89	2				41473,586	2			1	1		75111,01	Centrosomal protein kizuna OS=Homo sapiens OX=9606 GN=KIZ PE=1 SV=2
788	P30876|RPB2	29,62739	0,51	0,51	0	0	0	1	726644,5				1	1					133896,56	DNA-directed RNA polymerase II subunit RPB2 OS=Homo sapiens OX=9606 GN=POLR2B PE=1 SV=1
524	O15031|PLXE	72,99485	1,31	0,98	0,33	0	0	3					1	2	1			Acetylatic	205127,2	Plexin-B2 OS=Homo sapiens OX=9606 GN=PLXNB2 PE=1 SV=3
150	Q92630|DYRK	45,67938	1	0	1	0	0	1					1		1				66651,61	Dual specificity tyrosine-phosphorylation-regulated kinase 2 OS=Homo sapiens OX=9606 GN=DYRK2 PE=1 SV=3
653	Q15121|PEA1	77,39186	10	10	0	0	0	2	53057,44				2	2					15040,095	Astrocytic phosphoprotein PEA-15 OS=Homo sapiens OX=9606 GN=PEA15 PE=1 SV=2
630	Q8N357|S35F	50,61808	1,62	0	0	1,62	1,62	1			116166,08	198165,22	1			1	1		40214,188	Solute carrier family 35 member F6 OS=Homo sapiens OX=9606 GN=SLC35F6 PE=1 SV=1
712	Q9P0L9|PK2L	33,73256	0,75	0	0,75	0	0	1		56877,754			1		2				91981,96	Polycystic kidney disease 2-like 1 protein OS=Homo sapiens OX=9606 GN=PKD2L1 PE=1 SV=1
926	Q5T6L9|EMA	24,46977	0,88	0	0	0	0,88	1				95245,69	1				1		77787,75	Endoplasmic reticulum membrane-associated RNA degradation protein OS=Homo sapiens OX=9606 GN=ERMARD PE=1 SV=1
1001	Q8TEW8|PAF	27,50919	0,58	0,58	0	0	0	1	84579,14				1	1					132494,44	Partitioning defective 3 homolog B OS=Homo sapiens OX=9606 GN=PARD3B PE=1 SV=2
17	Q008S8|ECT2	150,6932	0,88	0,88	0,66	0,66	0,88	2	5245152	1402151,8	421829,3	4153430,5	2	6	3	2	8		104879,69	Epithelial cell-transforming sequence 2 oncogene-like OS=Homo sapiens OX=9606 GN=ECT2L PE=2 SV=2
8	Q9Y2X0|MED	76,61245	0,68	0	0	0,68	0,68	1			261959,2	184425,8	1			1	1		96793,15	Mediator of RNA polymerase II transcription subunit 16 OS=Homo sapiens OX=9606 GN=MED16 PE=1 SV=2
1002	Q9BXR6|FHR	57,26463	1,05	0	1,05	1,05	0	1		7086127	1306608,6		1		1	1			64419,406	Complement factor H-related protein 5 OS=Homo sapiens OX=9606 GN=CFHR5 PE=1 SV=1
147	A6NEV1|PR2	44,43359	2,26	2,26	2,26	0	0	1	837157,56	270945,03			1	1	1				28154,78	Proline-rich protein 23A OS=Homo sapiens OX=9606 GN=PRR23A PE=3 SV=1
770	O43929|ORC	42,8563	1,38	0	0	1,38	0	1					1			1			50377,34	Origin recognition complex subunit 4 OS=Homo sapiens OX=9606 GN=ORC4 PE=1 SV=2
343	Q92974|ARH	92,08601	0,61	0	0	0	0,61	1				113910,414	1				1		111542,55	Rho guanine nucleotide exchange factor 2 OS=Homo sapiens OX=9606 GN=ARHGEF2 PE=1 SV=4
144	P22692|IBP4_	367,2387	46,9	46,9	27,52	0	2,71	14	2,01E+07	3886618,2		30057,504	12	66	18		1	Carbamid	27933,97	Insulin-like growth factor-binding protein 4 OS=Homo sapiens OX=9606 GN=IGFBP4 PE=1 SV=2
1003	Q86TJ2|TAD2	57,74165	1,67	0	0	1,67	1,67	1			1136429,6	1072534	1			4	4		48470,33	Transcriptional adapter 2-beta OS=Homo sapiens OX=9606 GN=TADA2B PE=1 SV=2
504	O15014|ZN6(	44,5827	0,43	0	0	0,43	0	1			714981,25		1			3			151190,97	Zinc finger protein 609 OS=Homo sapiens OX=9606 GN=ZNF609 PE=1 SV=2
3	Q9BQS8|FYC	85,38101	0,41	0,41	0,41	0,41	0,41	1	2702878,5	3448463	1,37E+08	1,56E+08	1	3	2	5	5	Deamidat	166982,62	FYVE and coiled-coil domain-containing protein 1 OS=Homo sapiens OX=9606 GN=FYCO1 PE=1 SV=3
150	Q6EMK4|VAS	376,3661	21,25	15,3	10,25	0	4,9	16	3071426,2	1654921,5		1156222,6	14	31	19		6	Deamidat	71712,65	Vasorin OS=Homo sapiens OX=9606 GN=VASN PE=1 SV=1
450	P09651|ROA	94,55196	8,06	4,57	1,61	1,88	0	4	17078,705		853550,9		3	3	1	2			38746,645	Heterogeneous nuclear ribonucleoprotein A1 OS=Homo sapiens OX=9606 GN=HNRNPA1 PE=1 SV=5
140	O00142|KITM	104,2493	2,64	2,64	0	0	0	1	145197,19				1	1					31004,768	Thymidine kinase 2, mitochondrial OS=Homo sapiens OX=9606 GN=TK2 PE=1 SV=4
403	P21926|CD9_	162,2655	13,6	0	0	13,6	13,16	5			1216111,2	520786	5			9	4	Carbamid	25416,053	CD9 antigen OS=Homo sapiens OX=9606 GN=CD9 PE=1 SV=4
1004	P20839|IMDH	25,61178	1,17	0	0	0	1,17	1				50510,844	1				1		55405,79	Inosine-5'-monophosphate dehydrogenase 1 OS=Homo sapiens OX=9606 GN=IMPDH1 PE=1 SV=2
1005	Q96JG6|VPS	25,77142	0,62	0	0,62	0	0	1		38138,117			1		1				111174,01	Syndetin OS=Homo sapiens OX=9606 GN=VPS50 PE=1 SV=3
230	Q00653|NFKB	39,20821	0,67	0	0	0,67	0	1			46140,46		1			1			96749,17	Nuclear factor NF-kappa-B p100 subunit OS=Homo sapiens OX=9606 GN=NFKB2 PE=1 SV=4
235	O95197|RTN	78,36797	0,58	0,58	0,58	0	0	1	201011,17				1	1	3				112611,05	Reticulon-3 OS=Homo sapiens OX=9606 GN=RTN3 PE=1 SV=2
526	P06702|S10A	58,03199	22,81	0	0	22,81	11,4	3			484079,88	248315,27	3			4	2		13241,986	Protein S100-A9 OS=Homo sapiens OX=9606 GN=S100A9 PE=1 SV=1
541	P16455|MGM	96,59887	3,38	3,38	3,38	3,38	3,38	1	797892,56	305862,9	126546,97		1	1	1	1	1		21645,967	Methylated-DNA--protein-cysteine methyltransferase OS=Homo sapiens OX=9606 GN=MGMT PE=1 SV=1
1006	P07108|ACBP	40,62066	22,99	22,99	0	0	0	1	5346,449				1	1					10044,416	Acyl-CoA-binding protein OS=Homo sapiens OX=9606 GN=DBI PE=1 SV=2
36	P36406|TRI2	71,37429	1,05	1,05	1,05	1,05	1,05	1	486364,47	238729,16	48239,188	258262,4	1	1	1	1	1		64066,555	E3 ubiquitin-protein ligase TRIM23 OS=Homo sapiens OX=9606 GN=TRIM23 PE=1 SV=1
1007	Q96IC2|REXC	33,04628	0,78	0	0	0,78	0	1			37836,117		1			1			86885,9	RNA exonuclease 5 OS=Homo sapiens OX=9606 GN=REXO5 PE=2 SV=1
657	O95166|GBR	46,31821	11,11	0	0	0	11,11	2				73724,94	1				2		13918,036	Gamma-aminobutyric acid receptor-associated protein OS=Homo sapiens OX=9606 GN=GABARAP PE=1 SV=1
658	Q6ZRS2|SRC	66,48558	0,37	0,19	0,19	0,19	0,19	2	341350,28			476980,78	1	2	1	1	1	Acetylatic	343554,7	Helicase SRCAP OS=Homo sapiens OX=9606 GN=SRCAP PE=1 SV=3
1008	P50452|SPB8	22,84489	1,6	0	1,6	0	0	1		143685,67			1		1			Deamidat	42766,92	Serpin B8 OS=Homo sapiens OX=9606 GN=SERPINB8 PE=1 SV=2
120	P05067|A4_H	388,9002	26,49	26,49	12,99	0	0	21	2996842,2	1439004,9			18	27	21			Carbamid	86943,24	Amyloid-beta precursor protein OS=Homo sapiens OX=9606 GN=APP PE=1 SV=3
2	Q9UH62|ARM	36,16522	1,58	1,58	1,58	1,58	0	1	2536857,2	576682,5	524109,53		1	1	1	1			42500,613	Armadillo repeat-containing X-linked protein 3 OS=Homo sapiens OX=9606 GN=ARMCX3 PE=1 SV=1
220	Q96DF8|ESS2	33,75548	1,26	0	0	0	1,26	1				11011,109	1				1		52568,05	Splicing factor ESS-2 homolog OS=Homo sapiens OX=9606 GN=ESS2 PE=1 SV=1
84	Q9NVI1|FAN	92,82074	0,45	0,45	0	0	0	1	43984,78				1	1					149323,89	Fanconi anemia group I protein OS=Homo sapiens OX=9606 GN=FANCI PE=1 SV=4
59	Q8N1N2|DYN	116,6565	2,86	2,86	2,86	2,86	2,86	1	1394272,4	26281,844	253590,48	174674,47	1	6	1	2	2		22529,418	Dynactin-associated protein OS=Homo sapiens OX=9606 GN=DYNAP PE=1 SV=1
5	Q15654|TRIP	36,81825	1,26	0	1,26	0	0	1		44194,324			1		1				50287,875	Thyroid receptor-interacting protein 6 OS=Homo sapiens OX=9606 GN=TRIP6 PE=1 SV=3
336	P04439|HLAA	293,4755	23,56	0	2,47	11,51	23,56	7		31626,627	182647,66	329021,12	3		1	5	8	Deamidat	40840,723	HLA class I histocompatibility antigen, A alpha chain OS=Homo sapiens OX=9606 GN=HLA-A PE=1 SV=2
1009	Q2NL82|TSR1	31,61936	0,75	0	0	0,75	0	1			63620,664		1			1			91810,02	Pre-rRNA-processing protein TSR1 homolog OS=Homo sapiens OX=9606 GN=TSR1 PE=1 SV=1
78	Q9P2K1|C2D	83,70251	0,37	0,37	0,37	0	0	1					1	1	1				186185,31	Coiled-coil and C2 domain-containing protein 2A OS=Homo sapiens OX=9606 GN=CC2D2A PE=1 SV=3
659	Q6P158|DHX	61,18055	0,87	0	0,87	0	0	2		832982,8			2		4				155604,36	Putative ATP-dependent RNA helicase DHX57 OS=Homo sapiens OX=9606 GN=DHX57 PE=1 SV=2
404	P62834|RAP1	241,0913	20,65	0	0	20,65	16,85	5			6225,622	8822,583	1			5	4	Carbamid	20987,176	Ras-related protein Rap-1A OS=Homo sapiens OX=9606 GN=RAP1A PE=1 SV=1
264	Q68CP9|ARID	40,76168	0,33	0,33	0	0	0	1	24294,291				1	1					197390,86	AT-rich interactive domain-containing protein 2 OS=Homo sapiens OX=9606 GN=ARID2 PE=1 SV=2
436	P02671|FIBA_	29,38902	0,69	0,69	0,69	0	0	1	607323,06	239014,47			1	3	1				94973,03	Fibrinogen alpha chain OS=Homo sapiens OX=9606 GN=FGA PE=1 SV=2
405	O95967|FBLN	254,8677	11,29	8,8	11,29	0	0	5	448397,38	177544,12			5	5	8			Carbamid	49405,47	EGF-containing fibulin-like extracellular matrix protein 2 OS=Homo sapiens OX=9606 GN=EFEMP2 PE=1 SV=3
84	P01833|PIGR_	28,22482	0,79	0,79	0	0	0	1	472656,12				1	1					83283,57	Polymeric immunoglobulin receptor OS=Homo sapiens OX=9606 GN=PIGR PE=1 SV=4
1010	Q5U4P2|ASP	32,29033	1,54	0	0	1,54	0	1			22121,885		1			2			41127,746	Aspartate beta-hydroxylase domain-containing protein 1 OS=Homo sapiens OX=9606 GN=ASPHD1 PE=1 SV=3
1011	Q6P3W6|NBF	41,75044	0,16	0	0	0,16	0,16	1			5235523	7,28E+07	1			2	4		435634,25	Neuroblastoma breakpoint family member 10 OS=Homo sapiens OX=9606 GN=NBPF10 PE=3 SV=3
594	Q8IV31|TM1	26,71946	2,78	0	0	0	2,78	1					1				1		23729,104	Transmembrane protein 139 OS=Homo sapiens OX=9606 GN=TMEM139 PE=1 SV=1
530	Q96BM9|ARL	167,26	12,9	0	0	0	12,9	3					1				4		21415,89	ADP-ribosylation factor-like protein 8A OS=Homo sapiens OX=9606 GN=ARL8A PE=1 SV=1
24	P19174|PLCG	89,44501	0,47	0,47	0,47	0,47	0,47	1	7477650,5	692940,2	1582380,1		1	7	4	5	2		148532,08	1-phosphatidylinositol 4,5-bisphosphate phosphodiesterase gamma-1 OS=Homo sapiens OX=9606 GN=PLCG1 PE=1 SV=1
660	O60333|KIF1I	52,32411	0,66	0	0	0,66	0	2			89165,93		1			2		Deamidat	204476,31	Kinesin-like protein KIF1B OS=Homo sapiens OX=9606 GN=KIF1B PE=1 SV=5
34	Q7L8L6|FAKD	70,54435	0,79	0,79	0,79	0	0	1	7138,5107	12579,764			1	1	1			Carbamid	86574,29	FAST kinase domain-containing protein 5, mitochondrial OS=Homo sapiens OX=9606 GN=FASTKD5 PE=1 SV=1
1012	P32320|CDD_	35,16632	4,79	0	0	0	4,79	1				28638,916	1				1		16184,679	Cytidine deaminase OS=Homo sapiens OX=9606 GN=CDA PE=1 SV=2
593	P22531|SPR2	180,0902	30,56	0	0	30,56	30,56	2			894421,7	1087446,5	2			5	5	Carbamid	7855,3535	Small proline-rich protein 2E OS=Homo sapiens OX=9606 GN=SPRR2E PE=2 SV=2
661	Q99436|PSB7	69,99883	6,86	0	0	3,61	3,25	2			37479,21	51290,785	2			1	1		29965,42	Proteasome subunit beta type-7 OS=Homo sapiens OX=9606 GN=PSMB7 PE=1 SV=1
1013	O76076|CCN	23,21277	4	4	0	0	0	1					1	1				Carbamid	26825,025	CCN family member 5 OS=Homo sapiens OX=9606 GN=CCN5 PE=1 SV=1
208	P25311|ZA2G	335,4103	35,57	0	0	9,06	31,88	11			286032,72	963102,6	11			3	13	Deamidat	34258,715	Zinc-alpha-2-glycoprotein OS=Homo sapiens OX=9606 GN=AZGP1 PE=1 SV=2
1014	Q9H1E3|NUC	40,16772	3,7	3,7	0	0	0	1	196563,72				1	1					27296,379	Nuclear ubiquitous casein and cyclin-dependent kinase substrate 1 OS=Homo sapiens OX=9606 GN=NUCKS1 PE=1 SV=1
652	Q16543|CDC	29,76723	1,59	0	0	1,59	0	1					1			1			44468,3	Hsp90 co-chaperone Cdc37 OS=Homo sapiens OX=9606 GN=CDC37 PE=1 SV=1
1015	Q9NUZ1|ACC	24,13204	1,1	0	0	1,1	0	1					1			1			61795,465	Acyl-coenzyme A oxidase-like protein OS=Homo sapiens OX=9606 GN=ACOXL PE=2 SV=3
257	Q9BXJ5|C1Q	64,18456	2,11	0	2,11	0	0	1		46058,367			1		2				29951,84	Complement C1q tumor necrosis factor-related protein 2 OS=Homo sapiens OX=9606 GN=C1QTNF2 PE=1 SV=1
662	Q6ZVX7|FBX	77,0438	6,18	0	0	6,18	0	2			136787,1		2			2			30847,053	F-box only protein 50 OS=Homo sapiens OX=9606 GN=NCCRP1 PE=1 SV=1
1016	Q92847|GHS	30,14074	1,64	0	1,64	0	0	1					1		1				41328,504	Growth hormone secretagogue receptor type 1 OS=Homo sapiens OX=9606 GN=GHSR PE=1 SV=1
64	Q9NPH2|INC	34,14182	1,08	0	0	0	1,08	1					1				3		61067,805	Inositol-3-phosphate synthase 1 OS=Homo sapiens OX=9606 GN=ISYNA1 PE=1 SV=1
664	Q8WXI7|MU	84,0438	0,08	0,04	0,08	0,04	0,04	2		34864,406			1	1	2	1	1		1519174,8	Mucin-16 OS=Homo sapiens OX=9606 GN=MUC16 PE=1 SV=3
81	P07585|PGS2	416,9053	42,34	40,67	40,67	25,07	28,69	22	1,52E+08	9,29E+07	2258832,5	6478304,5	21	140	208	19	22	Deamidat	39746,777	Decorin OS=Homo sapiens OX=9606 GN=DCN PE=1 SV=1
246	Q15056|IF4H	92,18658	2,42	2,42	0	0	0	1	59444,12				1	1					27385,059	Eukaryotic translation initiation factor 4H OS=Homo sapiens OX=9606 GN=EIF4H PE=1 SV=5
188	Q8WYQ9|ZCH	78,02858	0,63	0,63	0,63	0,63	0,63	1		193457,72	81789,61	329750,56	1	1	1	1	1		100042,08	Zinc finger CCHC domain-containing protein 14 OS=Homo sapiens OX=9606 GN=ZCCHC14 PE=1 SV=1
608	Q5TBA9|FRY_	23,88448	0,2	0,2	0	0	0	1	115692,805				1	1					338874,66	Protein furry homolog OS=Homo sapiens OX=9606 GN=FRY PE=1 SV=1
376	Q8IUE6|H2A	267,1768	32,31	11,54	12,31	6,92	32,31	5	259248,44	294746,4	250080,11	2697408	5	4	6	2	7		13995,302	Histone H2A type 2-B OS=Homo sapiens OX=9606 GN=H2AC21 PE=1 SV=3
21	P98160|PGBM	589,0399	20,66	3,53	3,1	5,83	19,08	85	2581855	509183	1266292,6	1,66E+07	81	25	16	30	124	Oxidation	468829,97	Basement membrane-specific heparan sulfate proteoglycan core protein OS=Homo sapiens OX=9606 GN=HSPG2 PE=1 SV=4
249	Q9H347|UBQ	100,8806	0,92	0,92	0,92	0,92	0,92	1	1704077,6	705186,9	3805421,8	1,56E+07	1	6	7	6	4		70841,06	Ubiquilin-3 OS=Homo sapiens OX=9606 GN=UBQLN3 PE=1 SV=2
32	Q9BZ95|NSD	98,00636	0,42	0,42	0,42	0	0	1	91666,22	71625,22			1	2	2			Carbamid	161612,98	Histone-lysine N-methyltransferase NSD3 OS=Homo sapiens OX=9606 GN=NSD3 PE=1 SV=1
666	Q9NP84|TNR	112,1548	16,28	16,28	0	0	0	2	273321,6				2	3				Carbamid	13911,412	Tumor necrosis factor receptor superfamily member 12A OS=Homo sapiens OX=9606 GN=TNFRSF12A PE=1 SV=1
1017	Q99969|RAR	36,43278	4,29	4,29	0	0	0	1	34391,82				1	1				Carbamid	18617,578	Retinoic acid receptor responder protein 2 OS=Homo sapiens OX=9606 GN=RARRES2 PE=1 SV=1
10	O00268|TAF4	26,08867	0,55	0,55	0	0,55	0	1					1	1		1			110114,33	Transcription initiation factor TFIID subunit 4 OS=Homo sapiens OX=9606 GN=TAF4 PE=1 SV=2
1018	Q9BYK8|HEL	23,98892	0,23	0	0	0	0,23	1					1				1		294650,97	Helicase with zinc finger domain 2 OS=Homo sapiens OX=9606 GN=HELZ2 PE=1 SV=6
151	P23284|PPIB_	354,6461	59,26	56,48	37,5	24,54	28,7	15	1,51E+07	3735700	421522,38	525134,3	13	54	51	7	10	Oxidation	23742,533	Peptidyl-prolyl cis-trans isomerase B OS=Homo sapiens OX=9606 GN=PPIB PE=1 SV=2
1019	Q02779|M3K	41,38631	0,63	0	0,63	0,63	0,63	1		2922544,2	1586872,9		1		1	1	1		103694,49	Mitogen-activated protein kinase kinase kinase 10 OS=Homo sapiens OX=9606 GN=MAP3K10 PE=1 SV=3
49	P02458|CO2A	448,3052	18,09	13,99	14,26	8,34	8,07	42	2,67E+07	1,94E+07	3599600,2	1812419	9	86	91	31	26	Acetylatic	141785,38	Collagen alpha-1(II) chain OS=Homo sapiens OX=9606 GN=COL2A1 PE=1 SV=3
337	P40926|MDH	253,529	22,78	16,86	8,88	0	0	7	86452,61	101471,79			7	6	3			Carbamid	35503,277	Malate dehydrogenase, mitochondrial OS=Homo sapiens OX=9606 GN=MDH2 PE=1 SV=3
6	Q99543|DNJ	76,21806	0,97	0,97	0	0,97	0,97	1	95524,04		1177229,9	711697,1	1	1		2	1		71996,37	DnaJ homolog subfamily C member 2 OS=Homo sapiens OX=9606 GN=DNAJC2 PE=1 SV=4
229	Q01995|TAG	341,5031	50,75	50,75	0	0	0	11	1202235,6				11	14					22610,896	Transgelin OS=Homo sapiens OX=9606 GN=TAGLN PE=1 SV=4
20	Q5T8P6|RBM	65,05981	0,6	0	0	0,6	0,6	1			245865,69	188108,73	1			1	1		113597,3	RNA-binding protein 26 OS=Homo sapiens OX=9606 GN=RBM26 PE=1 SV=3
338	P35443|TSP4	261,067	6,04	3,43	2,81	2,71	5,1	6		2482460,2			3	19	8	8	10	Oxidation	105869,305	Thrombospondin-4 OS=Homo sapiens OX=9606 GN=THBS4 PE=1 SV=2
1020	O43768|ENS	36,01293	7,44	0	0	0	7,44	1				268290,6	1				1		13389,014	Alpha-endosulfine OS=Homo sapiens OX=9606 GN=ENSA PE=1 SV=1

1021	Q10471|GAL	43,4443	1,4	0	1,4	0	0	1					1		1				64732,754	Polypeptide N-acetylgalactosaminyltransferase 2 OS=Homo sapiens OX=9606 GN=GALNT2 PE=1 SV=1
1022	Q96S06|LMF	28,43442	1,06	0	1,06	0	0	1		122104,8			1		1				64873,465	Lipase maturation factor 1 OS=Homo sapiens OX=9606 GN=LMF1 PE=1 SV=1
51	Q07954|LRP1	478,6284	8,41	4,8	2,31	2,4	5,94	42	3484592,2	793301,44	413026,44	1212404,2	37	28	16	12	35	Carbamid	504606,3	Prolow-density lipoprotein receptor-related protein 1 OS=Homo sapiens OX=9606 GN=LRP1 PE=1 SV=2
531	P62269|RS18	161,0604	16,45	0	0	0	16,45	3				53478,33	3				3		17718,67	40S ribosomal protein S18 OS=Homo sapiens OX=9606 GN=RPS18 PE=1 SV=3
1023	Q5T5U3|RHG	93,72565	0,31	0,31	0	0	0	1	19121,674				1	1					217462,12	Rho GTPase-activating protein 21 OS=Homo sapiens OX=9606 GN=ARHGAP21 PE=1 SV=2
39	Q9H7B4|SMY	128,3908	1,64	0	0	1,4	1,64	2			89503,984	692991,6	2			1	4		49097,137	Histone-lysine N-methyltransferase SMYD3 OS=Homo sapiens OX=9606 GN=SMYD3 PE=1 SV=4
669	O94804|STK1	98,75842	1,24	0	0	0,62	1,24	2				92112,805	1			1	3		112135,234	Serine/threonine-protein kinase 10 OS=Homo sapiens OX=9606 GN=STK10 PE=1 SV=1
930	O75486|SUP	23,69546	1,89	0	1,89	0	0	1		37245,836			1		1				35792,78	Transcription initiation protein SPT3 homolog OS=Homo sapiens OX=9606 GN=SUPT3H PE=1 SV=3
117	P35556|FBN2	412,6862	6,25	6,25	1,99	0,48	1,41	21	1106012,2				12	46	15	3	8	Carbamid	314774,88	Fibrillin-2 OS=Homo sapiens OX=9606 GN=FBN2 PE=1 SV=3
1024	Q12888|TP53	22,81436	0,3	0	0	0,3	0	1					1			1		Carbamid	213574,4	TP53-binding protein 1 OS=Homo sapiens OX=9606 GN=TP53BP1 PE=1 SV=2
1025	Q8NGE8|OR4	27,11249	1,91	0	0	0	1,91	1				76108,34	1				2		35666,45	Olfactory receptor 4D9 OS=Homo sapiens OX=9606 GN=OR4D9 PE=3 SV=3
101	P67936|TPM	401,7059	69,76	69,76	52,02	13,71	5,24	25	8652904	1174149,6	76208,27		8	50	29	5	2	Acetylatic	28521,812	Tropomyosin alpha-4 chain OS=Homo sapiens OX=9606 GN=TPM4 PE=1 SV=3
167	P49006|MRP_	115,7252	5,13	5,13	5,13	0	0	1	393438,4	15452,1455			1	7	1			Deamidat	19528,791	MARCKS-related protein OS=Homo sapiens OX=9606 GN=MARCKSL1 PE=1 SV=2
256	Q15434|RBM	59,05715	1,47	0	1,47	0	0	1					1		1				43958,613	RNA-binding motif, single-stranded-interacting protein 2 OS=Homo sapiens OX=9606 GN=RBMS2 PE=1 SV=1
652	O60296|TRA	29,76723	0,66	0	0	0,66	0	1					1			1			101419,04	Trafficking kinesin-binding protein 2 OS=Homo sapiens OX=9606 GN=TRAK2 PE=1 SV=2
1026	Q92544|TM9	35,52444	0,93	0	0	0	0,93	1				63282,344	1				1		74518,75	Transmembrane 9 superfamily member 4 OS=Homo sapiens OX=9606 GN=TM9SF4 PE=1 SV=2
1027	O14523|C2C2	35,86885	0,85	0,85	0	0	0	1					1	1					76181,3	Phospholipid transfer protein C2CD2L OS=Homo sapiens OX=9606 GN=C2CD2L PE=1 SV=3
65	Q53SZ7|PRR3	111,5003	1,46	1,46	1,46	1,46	1,46	1	1533685,8	1106172,5	150388,16	214873,88	1	15	18	2	2		44689,68	Proline-rich protein 30 OS=Homo sapiens OX=9606 GN=PRR30 PE=1 SV=1
689	P15259|PGA	95,70122	3,95	3,95	3,95	3,95	3,95	1	203973,62	80420,01	65523,617		1	1	1	1	1		28766,166	Phosphoglycerate mutase 2 OS=Homo sapiens OX=9606 GN=PGAM2 PE=1 SV=3
368	P41229|KDM	86,18004	0,38	0	0	0	0,38	1				76225,984	1				1		175720,31	Lysine-specific demethylase 5C OS=Homo sapiens OX=9606 GN=KDM5C PE=1 SV=2
36	P01100|FOS_	115,9746	1,84	1,84	1,84	1,84	1,84	2	3167589,2	860580,6	105287,28	1092379,6	1	8	4	2	3	Deamidat	40695,406	Protein c-Fos OS=Homo sapiens OX=9606 GN=FOS PE=1 SV=1
376	Q7L7L0|H2A	236,4661	32,31	6,92	6,92	6,92	32,31	4	230969,52	229668,45	250080,11	2471626,2	4	3	4	2	9		14121,463	Histone H2A type 3 OS=Homo sapiens OX=9606 GN=H2AC25 PE=1 SV=3
540	Q8N1W1|AR	34,7939	0,35	0	0	0	0,35	1				34089,43	1				3		191890,6	Rho guanine nucleotide exchange factor 28 OS=Homo sapiens OX=9606 GN=ARHGEF28 PE=1 SV=3
373	P55290|CAD	260,4775	7,85	7,85	0,84	0,84	0	6	191631,8				5	7	1	1		Carbamid	78286,85	Cadherin-13 OS=Homo sapiens OX=9606 GN=CDH13 PE=1 SV=1
7	O00534|VMA	102,55	0,76	0,76	0,76	0,76	0,76	1	408923,62	171858,38		1874509,9	1	4	1	4	3		86489,26	von Willebrand factor A domain-containing protein 5A OS=Homo sapiens OX=9606 GN=VWA5A PE=1 SV=2
1028	Q9UEE5|ST1	57,44026	1,45	1,45	0	0	0	1	348172,53				1	1					46558,168	Serine/threonine-protein kinase 17A OS=Homo sapiens OX=9606 GN=STK17A PE=1 SV=2
301	P36888|FLT3_	137,6605	1,71	0	0	0	1,71	2				90602,195	1				2	Carbamid	112903,45	Receptor-type tyrosine-protein kinase FLT3 OS=Homo sapiens OX=9606 GN=FLT3 PE=1 SV=2
672	P50991|TCP	108,8514	2,23	1,11	1,11	2,23	1,11	2	74019,93	25637,965	564522,4		2	1	1	2	1		57924,23	T-complex protein 1 subunit delta OS=Homo sapiens OX=9606 GN=CCT4 PE=1 SV=4
816	Q9GZZ0|HXD	27,74358	1,83	0	1,83	0	0	1		970712,75			1		1				34093,49	Homeobox protein Hox-D1 OS=Homo sapiens OX=9606 GN=HOXD1 PE=2 SV=1
1028	Q92530|PSM	57,44026	2,21	2,21	0	0	0	1	348172,53				1	1					29816,727	Proteasome inhibitor PI31 subunit OS=Homo sapiens OX=9606 GN=PSMF1 PE=1 SV=2
673	Q8WXX0|DY	45,02374	0,3	0	0	0,15	0,15	2				1253976	1			1	1	Deamidat	461158,78	Dynein axonemal heavy chain 7 OS=Homo sapiens OX=9606 GN=DNAH7 PE=1 SV=2
532	D6RIA3|CD0	83,35093	1	0	0,33	0,67	0	3		111716,15			1		1	2		Acetylatic	190074,3	Uncharacterized protein C4orf54 OS=Homo sapiens OX=9606 GN=C4orf54 PE=2 SV=1
407	Q96RF0|SNX	250,1573	6,85	0	0	0	6,85	5				209221,11	5				5		68894,43	Sorting nexin-18 OS=Homo sapiens OX=9606 GN=SNX18 PE=1 SV=2
398	P04626|ERBB	32,09123	0,48	0,48	0,48	0	0	1	887625,8	83099,29			1	1	1				137910,48	Receptor tyrosine-protein kinase erbB-2 OS=Homo sapiens OX=9606 GN=ERBB2 PE=1 SV=1
533	P15880|RS2_	149,7886	8,19	0	0	0	8,19	3				164132,8	2				3		31324,422	40S ribosomal protein S2 OS=Homo sapiens OX=9606 GN=RPS2 PE=1 SV=2
1002	Q86UP6|CUZ	57,26463	0,99	0	0,99	0,99	0	1		7086127	1306608,6		1		1	1			68153,09	CUB and zona pellucida-like domain-containing protein 1 OS=Homo sapiens OX=9606 GN=CUZD1 PE=2 SV=1
1029	Q96NL6|SCLT	44,95101	0,87	0	0	0,87	0	1			10344,2705		1			1			80910,43	Sodium channel and clathrin linker 1 OS=Homo sapiens OX=9606 GN=SCLT1 PE=1 SV=2
675	Q86Y38|XYLT	79,67537	1,36	0,73	0,73	0	0,63	2	492383,7	356132,2			1	2	4		1		107569,23	Xylosyltransferase 1 OS=Homo sapiens OX=9606 GN=XYLT1 PE=1 SV=1
56	Q9BQ87|TBL1	86,54307	1,15	0	0	1,15	1,15	1			211660,44	1450422,8	1			1	1		56688,37	F-box-like/WD repeat-containing protein TBL1Y OS=Homo sapiens OX=9606 GN=TBL1Y PE=1 SV=1
27	Q86VS3|IQC	57,95885	0,58	0	0	0,58	0	1			19414,406		1			1			117348,59	IQ domain-containing protein H OS=Homo sapiens OX=9606 GN=IQCH PE=2 SV=2
1030	P54727|RD23	35,93669	2,2	2,2	0	0	0	1	108066,305				1	1					43171,176	UV excision repair protein RAD23 homolog B OS=Homo sapiens OX=9606 GN=RAD23B PE=1 SV=1
87	A6QL64|AN3	40,01609	0,31	0	0	0,31	0,31	1			127495,2	284008,75	1			1	1	Acetylatic	214500,23	Ankyrin repeat domain-containing protein 36A OS=Homo sapiens OX=9606 GN=ANKRD36 PE=2 SV=4
483	Q14696|MES	34,82591	2,56	0	2,56	0	2,56	1		403608,97		70899,88	1		1		1		26076,625	LRP chaperone MESD OS=Homo sapiens OX=9606 GN=MESD PE=1 SV=2
1031	Q17RY0|CPE	33,52007	0,82	0	0	0	0,82	1					1				1		80151,99	Cytoplasmic polyadenylation element-binding protein 4 OS=Homo sapiens OX=9606 GN=CPEB4 PE=1 SV=1
987	P51116|FXR2	66,54484	0,89	0	0,89	0	0,89	1		24584,418		165526,06	1		1		2		74223,305	RNA-binding protein FXR2 OS=Homo sapiens OX=9606 GN=FXR2 PE=1 SV=2
1031	O14647|CHD	33,52007	0,33	0	0	0	0,33	1					1				1		211343,5	Chromodomain-helicase-DNA-binding protein 2 OS=Homo sapiens OX=9606 GN=CHD2 PE=1 SV=2
32	B1ATL7|PRR3	95,24703	2,01	2,01	0	2,01	2,01	1			753473,44	2035653,6	1	2		4	5		31890,277	Proline-rich protein 32 OS=Homo sapiens OX=9606 GN=PRR32 PE=1 SV=1
726	O60938|KER	71,65733	1,99	1,99	0	0	0	1					1	1					40508,94	Keratocan OS=Homo sapiens OX=9606 GN=KERA PE=1 SV=1
676	P13473|LAM	89,99673	1,95	0	0	1,95	1,95	1			255514,83	1166309,6	1			1	6	Deamidat	44960,727	Lysosome-associated membrane glycoprotein 2 OS=Homo sapiens OX=9606 GN=LAMP2 PE=1 SV=2
303	P22626|ROA	289,6665	17,28	17,28	1,7	0	1,7	7	1029120,8	35414,516		108173,195	7	11	1		1	Oxidation	37429,695	Heterogeneous nuclear ribonucleoproteins A2/B1 OS=Homo sapiens OX=9606 GN=HNRNPA2B1 PE=1 SV=2
304	P80303|NUC	158,7996	14,05	14,05	6,9	0	0	8	521105,75	61284,61			7	9	5				50222,727	Nucleobindin-2 OS=Homo sapiens OX=9606 GN=NUCB2 PE=1 SV=3
39	Q8WUM4|PD	507,3757	45,62	0	0	20,39	45,62	44			2600329	2,20E+07	43			21	90	Carbamid	96023,164	Programmed cell death 6-interacting protein OS=Homo sapiens OX=9606 GN=PDCD6IP PE=1 SV=1
152	O75326|SEM	349,6609	21,92	19,22	4,95	0	0,9	17	1477170	2374884,8			16	18	5		1	Oxidation	74823,766	Semaphorin-7A OS=Homo sapiens OX=9606 GN=SEMA7A PE=1 SV=1
78	Q92619|HM	80,14496	0,53	0,53	0,53	0	0	1					1	1	1				124614,28	Rho GTPase-activating protein 45 OS=Homo sapiens OX=9606 GN=ARHGAP45 PE=1 SV=2
1032	Q8WXS8|ATS	47,494	0,49	0,49	0,49	0	0	1	304970,78	254798,3			1	1	2				133888,12	A disintegrin and metalloproteinase with thrombospondin motifs 14 OS=Homo sapiens OX=9606 GN=ADAMTS14 PE=2 SV=2
251	Q9BUD6|SP	318,8117	23,87	20,24	19,34	0	0	9	6591624	2176532,2			9	29	30			Deamidat	35788,31	Spondin-2 OS=Homo sapiens OX=9606 GN=SPON2 PE=1 SV=4
535	Q8NI08|NCO	36,07795	0,74	0,74	0	0	0	1					1	1					106161,5	Nuclear receptor coactivator 7 OS=Homo sapiens OX=9606 GN=NCOA7 PE=1 SV=2
679	P52272|HNR	115,9291	3,01	2,19	0,82	0	0	2	38311,12				1	1	1			Acetylatic	77515,52	Heterogeneous nuclear ribonucleoprotein M OS=Homo sapiens OX=9606 GN=HNRNPM PE=1 SV=3
374	P05109|S10A	201,9282	46,24	0	0	34,41	26,88	5			492291,03	746663,7	5			10	6	Carbamid	10834,506	Protein S100-A8 OS=Homo sapiens OX=9606 GN=S100A8 PE=1 SV=1
29	Q9NQC7|CYL	99,15334	0,63	0,63	0,63	0,63	0	1	271126,72	163930,73	26240,553		1	1	1	1			107315,56	Ubiquitin carboxyl-terminal hydrolase CYLD OS=Homo sapiens OX=9606 GN=CYLD PE=1 SV=1
311	Q9HB09|B2L1	107,3952	2,1	2,1	2,1	0	0	1					1	1	1				36821,48	Bcl-2-like protein 12 OS=Homo sapiens OX=9606 GN=BCL2L12 PE=1 SV=1
409	Q75N90|FBN	237,2011	1,17	1,17	0,53	0,53	0,53	5	255768,94				1	6	2	2	2	Carbamid	300355,66	Fibrillin-3 OS=Homo sapiens OX=9606 GN=FBN3 PE=2 SV=3
1033	P39019|RS19	71,46648	6,9	0	0	0	6,9	1				47568,645	1				1		16060,495	40S ribosomal protein S19 OS=Homo sapiens OX=9606 GN=RPS19 PE=1 SV=2
429	Q9UPU3|SOR	54,15968	0,49	0	0	0,49	0	1			26059,824		1			1			135754,98	VPS10 domain-containing receptor SorCS3 OS=Homo sapiens OX=9606 GN=SORCS3 PE=1 SV=2
681	Q96SI9|STRB	67,6836	1,93	0	0	0,89	1,04	2			116246,125		2			1	1		73652,52	Spermatid perinuclear RNA-binding protein OS=Homo sapiens OX=9606 GN=STRBP PE=1 SV=1
7	P17600|SYN1	67,49271	0,85	0	0	0	0,85	1				40116,016	1				1		74111,39	Synapsin-1 OS=Homo sapiens OX=9606 GN=SYN1 PE=1 SV=3
249	P62979|RS27	312,1252	39,1	39,1	33,33	39,1	39,1	8	7896435,5	3367563,2	1,28E+07	5,91E+07	8	34	29	35	49	Oxidation	17964,914	Ubiquitin-40S ribosomal protein S27a OS=Homo sapiens OX=9606 GN=RPS27A PE=1 SV=2
682	P62942|FKB1	98,17356	17,59	0	0	0	17,59	2				113129,516	2				2		11950,701	Peptidyl-prolyl cis-trans isomerase FKBP1A OS=Homo sapiens OX=9606 GN=FKBP1A PE=1 SV=2
1029	Q96MN5|TEA	44,95101	2,88	0	0	2,88	0	1			10344,2705		1			1			24150,01	Transcription elongation factor A N-terminal and central domain-containing protein 2 OS=Homo sapiens OX=9606 GN=TCEANC2 PE=1 SV=1
1034	Q9H492|MLP	34,97028	5,79	0	0	0	5,79	1				227544,36	1				1		14272,452	Microtubule-associated proteins 1A/1B light chain 3A OS=Homo sapiens OX=9606 GN=MAP1LC3A PE=1 SV=2
452	Q6UVK1|CSP	98,31236	1,16	0	0	0	1,16	4				142834,08	4				4		250536,77	Chondroitin sulfate proteoglycan 4 OS=Homo sapiens OX=9606 GN=CSPG4 PE=1 SV=2
93	Q8WV24|PHL	26,88499	1,5	0	0	0	1,5	1				476980,78	1				1		45016,223	Pleckstrin homology-like domain family A member 1 OS=Homo sapiens OX=9606 GN=PHLDA1 PE=1 SV=4
50	Q8IVG5|SAM	60,12066	0,38	0,38	0	0	0,38	1					1	1			1		184532,56	Sterile alpha motif domain-containing protein 9-like OS=Homo sapiens OX=9606 GN=SAMD9L PE=1 SV=2
1035	Q6ZTZ1|MSD	23,76196	2,16	0	0	2,16	0	1			231974,8		1			1			31632,188	Myb/SANT-like DNA-binding domain-containing protein 1 OS=Homo sapiens OX=9606 GN=MSANTD1 PE=2 SV=2
1007	Q8IYK2|CC10	33,04628	1,2	0	0	1,2	0	1			37836,117		1			1			56908,75	Coiled-coil domain-containing protein 105 OS=Homo sapiens OX=9606 GN=CCDC105 PE=1 SV=3
71	Q5VV43|K03	112,7286	0,56	0,56	0,56	0,56	0,56	1	63075,8	134069,75	54991,742	140715,61	1	1	1	2	2		117762,734	Dyslexia-associated protein KIAA0319 OS=Homo sapiens OX=9606 GN=KIAA0319 PE=1 SV=1
255	Q17R55|F187	52,44891	1,63	1,63	1,63	1,63	1,63	1				236152,39	1	1	1	1	1		42386,754	Protein FAM187B OS=Homo sapiens OX=9606 GN=FAM187B PE=2 SV=2
22	Q9BWP8|CO	36,56846	2,21	2,21	0	0	2,21	1					1	1			1		28665,367	Collectin-11 OS=Homo sapiens OX=9606 GN=COLEC11 PE=1 SV=1
277	P16870|CBPE	290,9862	15,13	15,13	0	0	0	9	573924,06				8	9					53150,65	Carboxypeptidase E OS=Homo sapiens OX=9606 GN=CPE PE=1 SV=1
775	Q8NFD5|ARI	76,97985	0,26	0,26	0	0	0	1	21360,566				1	1					243943,14	AT-rich interactive domain-containing protein 1B OS=Homo sapiens OX=9606 GN=ARID1B PE=1 SV=3
305	P29508|SPB3	224,9275	14,1	1,54	0	12,56	3,85	8	253790,36		95940,875	176123,56	7	3		7	3		44564,594	Serpin B3 OS=Homo sapiens OX=9606 GN=SERPINB3 PE=1 SV=2
27	O00750|P3C2	73,06244	0,37	0	0,37	0	0	1		204628,92			1		2				184767,84	Phosphatidylinositol 4-phosphate 3-kinase C2 domain-containing subunit beta OS=Homo sapiens OX=9606 GN=PIK3C2B PE=1 SV=2
3	P04264|K2C1	677,4097	84,16	56,37	56,68	80,43	82,61	199	3,84E+07	5,66E+07	3,43E+09	2,72E+09	153	132	207	1277	1066	Deamidat	66038,73	Keratin, type II cytoskeletal 1 OS=Homo sapiens OX=9606 GN=KRT1 PE=1 SV=6
519	A2RRD8|ZN3	32,15548	1,18	0	0	1,18	0	1			100736,945		1			1			59326,414	Zinc finger protein 320 OS=Homo sapiens OX=9606 GN=ZNF320 PE=1 SV=1
685	P31431|SDC4	163,9699	13,13	13,13	0	6,06	13,13	2	46875,895		99544,34		2	2		3	2		21641,555	Syndecan-4 OS=Homo sapiens OX=9606 GN=SDC4 PE=1 SV=2
535	P33908|MA1	86,97114	4,59	4,59	1,07	0	0	3	49081,867	37318,97			3	4	1				72968,52	Mannosyl-oligosaccharide 1,2-alpha-mannosidase IA OS=Homo sapiens OX=9606 GN=MAN1A1 PE=1 SV=3
410	Q9ULI3|HEG1	94,7842	2,82	0,43	0	0	2,39	5	1095459,2			73133,766	4	1			4	Carbamid	147460,56	Protein HEG homolog 1 OS=Homo sapiens OX=9606 GN=HEG1 PE=1 SV=3
78	Q8TD31|CCH	80,14496	0,77	0,77	0,77	0	0	1					1	1	1				88670,93	Coiled-coil alpha-helical rod protein 1 OS=Homo sapiens OX=9606 GN=CCHCR1 PE=1 SV=2
450	Q32P51|RA1	65,55508	5,31	3,12	0	2,19	0	2	17078,705		853550,9		2	2		2			34225,33	Heterogeneous nuclear ribonucleoprotein A1-like 2 OS=Homo sapiens OX=9606 GN=HNRNPA1L2 PE=2 SV=2
339	Q9H0U4|RAB	271,251	33,33	0	0	27,86	24,38	7			38113,543	60400,61	1			7	5		22171,156	Ras-related protein Rab-1B OS=Homo sapiens OX=9606 GN=RAB1B PE=1 SV=1
1036	Q9UGP5|DP	27,37833	1,04	0	0	1,04	0	1			31607,432		1			1			63482,254	DNA polymerase lambda OS=Homo sapiens OX=9606 GN=POLL PE=1 SV=1
1037	O75116|ROC	30,05041	0,94	0	0	0	0,94	1					1				1		160899,83	Rho-associated protein kinase 2 OS=Homo sapiens OX=9606 GN=ROCK2 PE=1 SV=4
801	Q69YN4|VIR_	23,08419	0,33	0	0,33	0	0	1		191882,12			1		1				202024,97	Protein virilizer homolog OS=Homo sapiens OX=9606 GN=VIRMA PE=1 SV=2
102	P09486|SPRC	423,3851	54,79	54,79	35,97	0	8,25	22	1,04E+08	6556876,5		145479,48	22	151	58		2	Oxidation	34632,156	SPARC OS=Homo sapiens OX=9606 GN=SPARC PE=1 SV=1
87	P01579|IFNG	100,6363	3,61	3,61	0	3,61	3,61	1	112304,12			279099,3	1	1		1	1		19348,299	Interferon gamma OS=Homo sapiens OX=9606 GN=IFNG PE=1 SV=1
436	P02679|FIBG	30,03086	1,32	1,32	1,32	0	0	1	972326,75	250111,97			1	4	2				51511,652	Fibrinogen gamma chain OS=Homo sapiens OX=9606 GN=FGG PE=1 SV=3
1038	A8MWX3|WA	32,29613	1,26	0	1,26	0	0	1		2596631			1		1				51595,46	Putative WAS protein family homolog 4 OS=Homo sapiens OX=9606 GN=WASH4P PE=5 SV=1
850	Q4G0A6|MIN	47,90064	0,79	0	0	0,79	0	1					1			1			84371,734	Probable ubiquitin carboxyl-terminal hydrolase MINDY-4 OS=Homo sapiens OX=9606 GN=MINDY4 PE=1 SV=2
375	Q9UKX5|ITA1	164,8693	3,87	0	0	3,03	0,84	6			219265,28		5			6	1		133470,16	Integrin alpha-11 OS=Homo sapiens OX=9606 GN=ITGA11 PE=1 SV=2
1039	O75508|CLD1	39,60198	3,86	0	0	0	3,86	1				220490,47	1				2		21993,201	Claudin-11 OS=Homo sapiens OX=9606 GN=CLDN11 PE=1 SV=2

813	Q8WXI2|CNK	37,56862	0,58	0,58	0,58	0	0,58	1	154735,44				1	1	2		1		117534,5	Connector enhancer of kinase suppressor of ras 2 OS=Homo sapiens OX=9606 GN=CNKSR2 PE=1 SV=1
504	Q6U841|S4A	44,5827	0,54	0	0	0,54	0	1			714981,25		1			3			125946,266	Sodium-driven chloride bicarbonate exchanger OS=Homo sapiens OX=9606 GN=SLC4A10 PE=1 SV=1
252	P62807|H2B1	317,3283	66,67	34,13	27,78	29,37	66,67	10	117890,07	89813,125	87343,49	987559,8	1	7	7	7	17	Oxidation	13906,135	Histone H2B type 1-C/E/F/G/I OS=Homo sapiens OX=9606 GN=H2BC4 PE=1 SV=4
1040	Q9P1V8|SAM	23,64662	0,89	0	0	0	0,89	1				91289,55	1				1		77150,69	Sterile alpha motif domain-containing protein 15 OS=Homo sapiens OX=9606 GN=SAMD15 PE=2 SV=1
306	P08195|4F2_I	261,3747	13,02	0	0	8,1	7,94	8			150671,48	177967,75	7			5	5	Deamidat	67994	4F2 cell-surface antigen heavy chain OS=Homo sapiens OX=9606 GN=SLC3A2 PE=1 SV=3
1041	Q8IXT2|DMR	31,60079	1,63	0	0	0	1,63	1				3539239,2	1				2		39124,035	Doublesex- and mab-3-related transcription factor C2 OS=Homo sapiens OX=9606 GN=DMRTC2 PE=2 SV=2
719	P02008|HBAZ	92,47081	4,93	0	0	0	4,93	1				224168,84	1				1		15637,0205	Hemoglobin subunit zeta OS=Homo sapiens OX=9606 GN=HBZ PE=1 SV=2
686	P62857|RS28	84,18407	33,33	33,33	0	0	0	2	20810,162				2	3				Carbamid	7841,022	40S ribosomal protein S28 OS=Homo sapiens OX=9606 GN=RPS28 PE=1 SV=1
81	Q969X0|RIPL	93,6294	2,84	2,84	2,84	2,84	2,84	1	1,58E+07	9244560	462313,25	1002603,1	1	6	5	3	3		23986,17	RILP-like protein 2 OS=Homo sapiens OX=9606 GN=RILPL2 PE=1 SV=1
412	P45984|MK0	106,6074	1,65	1,65	0	1,65	1,65	1	85046,65		226533,25	201726,97	1	1		1	1		48139,06	Mitogen-activated protein kinase 9 OS=Homo sapiens OX=9606 GN=MAPK9 PE=1 SV=2
672	Q9NPC8|SIX2	66,51751	2,06	0	0	2,06	0	1			480016,22		1			1			32286,324	Homeobox protein SIX2 OS=Homo sapiens OX=9606 GN=SIX2 PE=1 SV=1
453	Q5JWF2|GNA	173,7386	3,09	0	0	3,09	1,64	4			19952,809		2			6	3		111024,516	Guanine nucleotide-binding protein G(s) subunit alpha isoforms XLas OS=Homo sapiens OX=9606 GN=GNAS PE=1 SV=2
411	Q9H4M9|EHD	220,6329	8,05	0	0	0	8,05	5				437980,22	5				8		60626,86	EH domain-containing protein 1 OS=Homo sapiens OX=9606 GN=EHD1 PE=1 SV=2
594	Q9Y6Y0|NS1B	26,71946	0,93	0	0	0	0,93	1					1				1		71729,37	Influenza virus NS1A-binding protein OS=Homo sapiens OX=9606 GN=IVNS1ABP PE=1 SV=3
1042	Q63HN1|S31I	32,33427	1,08	0	0	1,08	0	1			62760,535		1			1			61860,17	Putative protein SPATA31F2P OS=Homo sapiens OX=9606 GN=SPATA31F2P PE=5 SV=1
307	P35321|SPR1	314,7575	61,8	0	0	61,8	59,55	8			109808,48	108541,93	1			27	12	Carbamid	9877,475	Cornifin-A OS=Homo sapiens OX=9606 GN=SPRR1A PE=1 SV=2
70	Q5CZC0|FSIP	83,96447	0,09	0	0	0,09	0,09	1			64976,27	250123	1			3	1		780607,44	Fibrous sheath-interacting protein 2 OS=Homo sapiens OX=9606 GN=FSIP2 PE=2 SV=4
253	Q17RW2|CO	247,383	4,14	3,09	1,75	0,7	1,4	10	183642,27				1	14	9	4	9	Deamidat	175496,38	Collagen alpha-1(XXIV) chain OS=Homo sapiens OX=9606 GN=COL24A1 PE=1 SV=2
153	P21810|PGS1	371,3147	39,4	39,4	25,82	2,17	7,34	15	1,69E+07	4987725		168975,62	14	75	60	3	4	Deamidat	41654,06	Biglycan OS=Homo sapiens OX=9606 GN=BGN PE=1 SV=2
687	P78324|SHPS	56,69019	2,38	0	1,19	0	1,19	2		58114,875			2		1		1		54966,85	Tyrosine-protein phosphatase non-receptor type substrate 1 OS=Homo sapiens OX=9606 GN=SIRPA PE=1 SV=2
184	O43852|CALU	364,3093	38,41	38,41	27,3	0	2,54	14	1,55E+07	7004337		23732,883	13	50	35		1		37106,78	Calumenin OS=Homo sapiens OX=9606 GN=CALU PE=1 SV=2
340	P02788|TRFL	265,9682	8,17	6,76	5,92	3,38	3,52	7	7757996,5	374787,25	182607,4	50437,082	5	19	14	4	3	Carbamid	78181,95	Lactotransferrin OS=Homo sapiens OX=9606 GN=LTF PE=1 SV=6
81	Q5EBL4|RIPL	93,6294	1,49	1,49	1,49	1,49	1,49	1	1,58E+07	9244560	462313,25	1002603,1	1	6	5	3	3		47107,934	RILP-like protein 1 OS=Homo sapiens OX=9606 GN=RILPL1 PE=1 SV=1
29	Q86UK0|ABC	71,94605	0,23	0,23	0	0,23	0,23	1	793722,7			332417,66	1	1		1	1		293236,6	Glucosylceramide transporter ABCA12 OS=Homo sapiens OX=9606 GN=ABCA12 PE=1 SV=3
807	Q6ZVT0|TTL1	24,75468	0,89	0	0	0,89	0	1			108264,164		1			1			75041,516	Inactive polyglycylase TTLL10 OS=Homo sapiens OX=9606 GN=TTLL10 PE=1 SV=2
537	O15400|STX7	156,919	14,18	0	0	2,3	11,88	3				80684,93	2			1	2	Acetylatic	29815,53	Syntaxin-7 OS=Homo sapiens OX=9606 GN=STX7 PE=1 SV=4
185	P01034|CYTC	339,5981	50,68	50,68	34,93	0	0	11	1,28E+07	1207859,8			10	49	20			Oxidation	15799,219	Cystatin-C OS=Homo sapiens OX=9606 GN=CST3 PE=1 SV=1
1043	Q00765|REEP	33,64715	4,23	0	0	4,23	4,23	1				33926,047	1			1	1		21493,12	Receptor expression-enhancing protein 5 OS=Homo sapiens OX=9606 GN=REEP5 PE=1 SV=3
271	O60911|CATL	127,8568	3,89	2,1	3,89	0	2,1	2					1	2	4		1		37329,35	Cathepsin L2 OS=Homo sapiens OX=9606 GN=CTSV PE=1 SV=2
539	Q00610|CLH1	71,76734	1,61	0	0	1,13	0,48	3			88896,5		3			2	1	Carbamid	191614,62	Clathrin heavy chain 1 OS=Homo sapiens OX=9606 GN=CLTC PE=1 SV=5
689	P18669|PGAM	115,1301	10,24	10,24	3,94	3,94	3,94	2	203973,62	80420,01	65523,617		2	2	1	1	1	Deamidat	28803,928	Phosphoglycerate mutase 1 OS=Homo sapiens OX=9606 GN=PGAM1 PE=1 SV=2
175	Q14315|FLNC	339,3004	4,26	3,56	0	1,58	0,22	14	166038,94		17487,328		5	12		7	1	Carbamid	291022,22	Filamin-C OS=Homo sapiens OX=9606 GN=FLNC PE=1 SV=3
428	Q9Y573|IPP_	39,77482	1,03	1,03	0	0	0	1	195295,67				1	1					65260,895	Actin-binding protein IPP OS=Homo sapiens OX=9606 GN=IPP PE=1 SV=1
1044	Q5VSY0|GKA	40,36036	1,64	0	0	1,64	0	1			63496,605		1			1			42078,09	G kinase-anchoring protein 1 OS=Homo sapiens OX=9606 GN=GKAP1 PE=1 SV=2
459	Q9UHK6|AMA	28,68871	1,57	0	0	1,57	1,57	1			3851886	187572,34	1			1	1	Acetylatio	42386,707	Alpha-methylacyl-CoA racemase OS=Homo sapiens OX=9606 GN=AMACR PE=1 SV=2
195	Q5KU26|COL	279,5203	11,73	3,5	10,92	2,7	1,89	13	116585,27	1773052,5	261307,69		8	7	20	6	4	Carbamid	81515,16	Collectin-12 OS=Homo sapiens OX=9606 GN=COLEC12 PE=1 SV=3
692	O75095|MEG	42,84867	0,97	0,97	0	0	0	2	150456,89				2	2					161185,19	Multiple epidermal growth factor-like domains protein 6 OS=Homo sapiens OX=9606 GN=MEGF6 PE=1 SV=4
232	Q13882|PTK6	110,2703	1,33	1,33	1,33	0	0	1	815245,1	442052,6			1	3	2			Carbamid	51834,168	Protein-tyrosine kinase 6 OS=Homo sapiens OX=9606 GN=PTK6 PE=1 SV=1
430	Q9P2N4|ATS	29,12412	0,31	0	0	0	0,31	1				41747,074	1				1		216491,16	A disintegrin and metalloproteinase with thrombospondin motifs 9 OS=Homo sapiens OX=9606 GN=ADAMTS9 PE=1 SV=4
26	Q9UQ52|CNT	99,03803	0,58	0	0	0,58	0	1			60504,12		1			1			113956,2	Contactin-6 OS=Homo sapiens OX=9606 GN=CNTN6 PE=1 SV=1
67	Q92626|PXDN	480,9147	21,97	8,72	1,69	6,09	21,43	35	646316,8	81672,55	598717	7901555	34	18	3	13	52	Deamidat	165274,61	Peroxidasin homolog OS=Homo sapiens OX=9606 GN=PXDN PE=1 SV=2
693	O00429|DNM	113,816	1,63	0,82	0	0,82	0	2			57405,086		1	1		1			81877,15	Dynamin-1-like protein OS=Homo sapiens OX=9606 GN=DNM1L PE=1 SV=2
694	A1A5C7|S22A	64,55109	1,75	0	0	0,87	0,87	2				52255,74	1			1	1		73748,195	Solute carrier family 22 member 23 OS=Homo sapiens OX=9606 GN=SLC22A23 PE=1 SV=2
163	O94985|CSTN	378,6401	14,37	14,37	9,79	0	0	16	4606897,5	1063762,6			15	31	16			Carbamid	109792,75	Calsyntenin-1 OS=Homo sapiens OX=9606 GN=CLSTN1 PE=1 SV=1
454	O95996|APCL	113,2015	1,04	0,52	0,52	0,78	0,52	4	127115,24	68007,836			1	2	3	4	2	Acetylatic	243948,64	Adenomatous polyposis coli protein 2 OS=Homo sapiens OX=9606 GN=APC2 PE=1 SV=1
5	Q93052|LPP_	36,81825	0,98	0	0,98	0	0	1		44194,324			1		1				65746,33	Lipoma-preferred partner OS=Homo sapiens OX=9606 GN=LPP PE=1 SV=1
696	Q9Y6J0|CABI	63,23907	0,68	0,27	0,41	0	0	2		150656,62			1	1	1				246352,08	Calcineurin-binding protein cabin-1 OS=Homo sapiens OX=9606 GN=CABIN1 PE=1 SV=1
29	P06727|APOA	71,94605	1,52	1,52	0	1,52	1,52	1	793722,7			332417,66	1	1		1	1		45372,03	Apolipoprotein A-IV OS=Homo sapiens OX=9606 GN=APOA4 PE=1 SV=4
430	Q2YD98|UVSS	29,12412	0,85	0	0	0	0,85	1				41747,074	1				1		80591,484	UV-stimulated scaffold protein A OS=Homo sapiens OX=9606 GN=UVSSA PE=1 SV=2
125	P09493|TPM1	380,9138	54,58	54,58	28,52	6,69	4,58	21	418986,44	29393,629			4	39	19	3	2	Acetylatic	32708,564	Tropomyosin alpha-1 chain OS=Homo sapiens OX=9606 GN=TPM1 PE=1 SV=2
84	P55268|LAMB	419,0171	13,18	9,07	0,33	3,45	3,56	28	1579844		176646,73	227606,38	27	27	2	8	8	Carbamid	195981,06	Laminin subunit beta-2 OS=Homo sapiens OX=9606 GN=LAMB2 PE=1 SV=2
1045	Q86T82|UBP3	29,49904	0,61	0,61	0	0	0	1					1	1					110169,65	Ubiquitin carboxyl-terminal hydrolase 37 OS=Homo sapiens OX=9606 GN=USP37 PE=1 SV=2
230	P35580|MYH1	269,8598	4,1	1,06	0,3	4,1	0,3	11			93582,52		2	3	1	12	1	Acetylatic	228999,11	Myosin-10 OS=Homo sapiens OX=9606 GN=MYH10 PE=1 SV=3
1046	P02790|HEM	59,77851	1,3	1,3	0	0	0	1					1	2					51676,367	Hemopexin OS=Homo sapiens OX=9606 GN=HPX PE=1 SV=2
305	P48594|SPB4	214,7286	12,05	1,54	0	10,51	3,85	7	253790,36		95940,875	176123,56	6	3		6	3		44853,926	Serpin B4 OS=Homo sapiens OX=9606 GN=SERPINB4 PE=1 SV=2
499	Q8N8H1|ZN3	26,02027	3,66	3,66	0	0	0	1	20594,729				1	1				Carbamid	18964,146	Putative protein ZNF321 OS=Homo sapiens OX=9606 GN=ZNF321P PE=5 SV=3
881	Q9UPQ0|LIM	26,81628	0,55	0	0	0,55	0	1					1			1			121867,19	LIM and calponin homology domains-containing protein 1 OS=Homo sapiens OX=9606 GN=LIMCH1 PE=1 SV=4
101	Q2TAC2|CCD	94,92599	0,77	0,77	0,77	0,77	0	1	1244120,8	138677,22	76208,27		1	2	1	1			103038,92	Coiled-coil domain-containing protein 57 OS=Homo sapiens OX=9606 GN=CCDC57 PE=1 SV=3
455	Q9NZN4|EHD	131,3778	6,26	0	0	1,47	6,26	4			24368,479	170787,16	4			1	5		61161,395	EH domain-containing protein 2 OS=Homo sapiens OX=9606 GN=EHD2 PE=1 SV=2
341	O14498|ISLR	274,9862	14,25	14,25	14,25	0	0	6	2167093,5	729456,75			6	14	13			Carbamid	45997,31	Immunoglobulin superfamily containing leucine-rich repeat protein OS=Homo sapiens OX=9606 GN=ISLR PE=2 SV=1
171	P50336|PPOX	85,21694	1,26	0	0	0	1,26	1				314946,9	1				2		50765,3	Protoporphyrinogen oxidase OS=Homo sapiens OX=9606 GN=PPOX PE=1 SV=1
24	P01031|CO5_	89,44501	0,36	0,36	0,36	0,36	0,36	1	7477650,5	692940,2	1582380,1		1	7	4	5	2		188305,3	Complement C5 OS=Homo sapiens OX=9606 GN=C5 PE=1 SV=4
1047	Q9NY28|GALT	25,96144	0,94	0	0,94	0	0	1		48452,582			1		1				72851,414	Probable polypeptide N-acetylgalactosaminyltransferase 8 OS=Homo sapiens OX=9606 GN=GALNT8 PE=2 SV=1
412	P32119|PRDX	251,1012	20,2	9,09	5,56	20,2	20,2	5	85046,65		473421,8	392429,34	3	2	1	6	5		21891,916	Peroxiredoxin-2 OS=Homo sapiens OX=9606 GN=PRDX2 PE=1 SV=5
770	O75185|AT2C	42,8563	0,63	0	0	0,63	0	1					1			1			103169,38	Calcium-transporting ATPase type 2C member 2 OS=Homo sapiens OX=9606 GN=ATP2C2 PE=1 SV=3
376	Q99878|H2A1	279,6636	38,28	11,72	12,5	7,03	38,28	6	259248,44	294746,4	250080,11	2714422,5	6	4	6	2	11		13936,277	Histone H2A type 1-J OS=Homo sapiens OX=9606 GN=H2AC14 PE=1 SV=3
50	Q9HBL0|TENS	40,68839	0,33	0	0	0	0,33	1				84711,664	1				1		197022,6	Tensin-1 OS=Homo sapiens OX=9606 GN=TNS1 PE=1 SV=3
1	P12111|CO6A	741,1123	63,58	39,82	30,06	50,14	61,32	314	8,34E+07	2,95E+07	2,67E+08	2,49E+09	298	370	280	565	1401	Acetylatic	343669,38	Collagen alpha-3(VI) chain OS=Homo sapiens OX=9606 GN=COL6A3 PE=1 SV=5
729	Q04656|ATP7	23,46282	0,4	0	0,4	0	0	1		141745,42			1		1				163372,77	Copper-transporting ATPase 1 OS=Homo sapiens OX=9606 GN=ATP7A PE=1 SV=4
8	Q6S5L8|SHC4	60,21952	0,95	0	0	0,95	0,95	1			1629398,8	554100,6	1			2	1		68784,945	SHC-transforming protein 4 OS=Homo sapiens OX=9606 GN=SHC4 PE=1 SV=1
314	P10643|CO7_	83,04704	0,83	0	0	0,83	0,83	1			180821,11	241853,95	1			1	1		93518,234	Complement component C7 OS=Homo sapiens OX=9606 GN=C7 PE=1 SV=2
706	B2RXH8|HNR	43,84666	3,41	3,41	0	0	0	1	79983,87				1	2					32072,305	Heterogeneous nuclear ribonucleoprotein C-like 2 OS=Homo sapiens OX=9606 GN=HNRNPCL2 PE=1 SV=1
308	Q9P2P6|STAR	197,2234	1,02	0,87	0,26	0,13	0,47	8	433709,2			245467,38	6	9	5	2	3	Acetylatio	516343,4	StAR-related lipid transfer protein 9 OS=Homo sapiens OX=9606 GN=STARD9 PE=1 SV=3
453	P63092|GNA	147,0857	5,08	0	0	5,08	2,79	2			19952,809		1			4	2		45664,58	Guanine nucleotide-binding protein G(s) subunit alpha isoforms short OS=Homo sapiens OX=9606 GN=GNAS PE=1 SV=1
1002	Q96GS4|BOR	57,26463	1,68	0	1,68	1,68	0	1		7086127	1306608,6		1		1	1			37226,273	BLOC-1-related complex subunit 6 OS=Homo sapiens OX=9606 GN=BORCS6 PE=1 SV=2
150	Q15884|EREP	45,67938	1,33	0	1,33	0	0	1					1		1				49703,4	Endosomal transmembrane epsin interactor 1 OS=Homo sapiens OX=9606 GN=ENTREP1 PE=1 SV=3
1048	Q10570|CPSF	49,37188	0,49	0,49	0	0,49	0,49	1	184615,56		150096,05		1	1		1	1		160883,88	Cleavage and polyadenylation specificity factor subunit 1 OS=Homo sapiens OX=9606 GN=CPSF1 PE=1 SV=2
278	P35241|RADI	282,504	12,35	7,72	3,77	4,63	1,03	9					1	9	3	4	1		68563,9	Radixin OS=Homo sapiens OX=9606 GN=RDX PE=1 SV=1
1049	P06681|CO2_	41,2469	0,93	0	0,93	0	0	1		21646,436			1		1				83267,83	Complement C2 OS=Homo sapiens OX=9606 GN=C2 PE=1 SV=2
540	Q6NZY4|ZCH	113,8266	2,55	0,85	0,85	0	1,7	3				56336,125	2	1	1		4		78577,375	Zinc finger CCHC domain-containing protein 8 OS=Homo sapiens OX=9606 GN=ZCCHC8 PE=1 SV=2
567	Q9BXB5|OSB	45,91409	0,79	0	0	0	0,79	1				103208,62	1				1	Acetylatio	83970,055	Oxysterol-binding protein-related protein 10 OS=Homo sapiens OX=9606 GN=OSBPL10 PE=1 SV=2
556	Q9H8M9|EVA	116,8178	7,24	0	0	0	7,24	1					1				1		17469,688	Protein eva-1 homolog A OS=Homo sapiens OX=9606 GN=EVA1A PE=1 SV=1
459	Q9NZL9|MAT	28,68871	1,8	0	0	1,8	1,8	1			3851886	187572,34	1			1	1	Acetylatio	37551,8	Methionine adenosyltransferase 2 subunit beta OS=Homo sapiens OX=9606 GN=MAT2B PE=1 SV=1
447	P16402|H13_	217,6627	17,65	5,43	0	3,17	17,65	4				57619,617	2	1		1	4		22349,9	Histone H1.3 OS=Homo sapiens OX=9606 GN=H1-3 PE=1 SV=2
38	Q96JM7|LMB	71,83782	0,77	0,77	0,77	0,77	0,77	1	174143,88	289360,03	61608,51	72641,82	1	8	7	1	1		88336,66	Lethal(3)malignant brain tumor-like protein 3 OS=Homo sapiens OX=9606 GN=L3MBTL3 PE=1 SV=2
541	Q9H299|SH3B	191,7003	26,88	26,88	26,88	7,53	7,53	3	2578422,2	1203245	126546,97		3	4	4	1	1		10437,712	SH3 domain-binding glutamic acid-rich-like protein 3 OS=Homo sapiens OX=9606 GN=SH3BGRL3 PE=1 SV=1
542	Q86UQ4|ABC	73,09314	0,36	0	0	0,36	0	3			29270,012		1			4			576159,3	ATP-binding cassette sub-family A member 13 OS=Homo sapiens OX=9606 GN=ABCA13 PE=2 SV=3
209	Q9C075|K1C2	302,4784	20,62	0	0	15,64	13,98	10			116061,94	63418,93	6			13	10	Oxidation	48131,105	Keratin, type I cytoskeletal 23 OS=Homo sapiens OX=9606 GN=KRT23 PE=1 SV=2
102	Q14515|SPRL	47,93971	0,9	0,9	0,9	0	0	1	5021865,5				1	8	1				75207,74	SPARC-like protein 1 OS=Homo sapiens OX=9606 GN=SPARCL1 PE=1 SV=2
543	Q15084|PDIA	179,4323	5,68	5,68	0	0	0	3	222479,1				3	3					48121,32	Protein disulfide-isomerase A6 OS=Homo sapiens OX=9606 GN=PDIA6 PE=1 SV=1
283	Q9H0A8|COM	40,32876	3,02	0	0	3,02	0	1			115794,38		1			1			21764,342	COMM domain-containing protein 4 OS=Homo sapiens OX=9606 GN=COMMD4 PE=1 SV=1
93	Q8N2C3|DEP	26,88499	2,04	0	0	0	2,04	1				476980,78	1				1		33693,848	DEP domain-containing protein 4 OS=Homo sapiens OX=9606 GN=DEPDC4 PE=2 SV=1
1050	P0DOX8|IGL1	87,25446	2,78	2,78	0	0	0	1					1	1					22830,168	Immunoglobulin lambda-1 light chain OS=Homo sapiens OX=9606 PE=1 SV=1
164	P00751|CFAB	367,4451	17,15	15,97	15,18	0	0	15	2191185,2	1168818,9			15	24	24			Carbamid	85532,87	Complement factor B OS=Homo sapiens OX=9606 GN=CFB PE=1 SV=2
279	P39059|COFA	316,9997	6,12	6,12	0	0,43	0	9	764004,25				7	16		1		Carbamid	141720,14	Collagen alpha-1(XV) chain OS=Homo sapiens OX=9606 GN=COL15A1 PE=1 SV=2
1051	Q04900|MUC	24,85836	5,08	5,08	0	0	0	1	27934,96				1	1				Carbamid	20916,701	Sialomucin core protein 24 OS=Homo sapiens OX=9606 GN=CD164 PE=1 SV=2
1052	P42345|MTO	25,04543	0,24	0,24	0	0	0	1	18750,098				1	1					288891,6	Serine/threonine-protein kinase mTOR OS=Homo sapiens OX=9606 GN=MTOR PE=1 SV=1
421	P30203|CD6_	46,69381	0,9	0,9	0	0	0	1	31704,02				1	1					71800,9	T-cell differentiation antigen CD6 OS=Homo sapiens OX=9606 GN=CD6 PE=1 SV=3
176	P01023|A2M	354,6368	7,12	6,58	5,29	7,06	6,65	13	1,50E+07	5813278,5	1,31E+07	1,09E+07	10	35	31	36	26	Deamidat	163291	Alpha-2-macroglobulin OS=Homo sapiens OX=9606 GN=A2M PE=1 SV=3
704	Q9Y5Y9|SCN	38,03729	0,36	0	0	0,36	0	1			266009,94		1			2		Oxidation	220625,86	Sodium channel protein type 10 subunit alpha OS=Homo sapiens OX=9606 GN=SCN10A PE=1 SV=2
545	O95980|RECK	83,86667	2,57	0	1,75	0,62	1,44	3		19046,146		1451948,9	2		2	1	2	Carbamid	106457,336	Reversion-inducing cysteine-rich protein with Kazal motifs OS=Homo sapiens OX=9606 GN=RECK PE=1 SV=1

153	Q6P4F1|FUT	94,5107	1,25	1,25	1,25	0	0	1	1323350,4	257456,61			1	4	4				56094,36	Alpha-(1,3)-fucosyltransferase 10 OS=Homo sapiens OX=9606 GN=FUT10 PE=1 SV=2
706	P07910|HNR	71,88212	6,86	6,86	0	0	0	2	79983,87				2	3					33670	Heterogeneous nuclear ribonucleoproteins C1/C2 OS=Homo sapiens OX=9606 GN=HNRNPC PE=1 SV=4
1053	Q8TD26|CHD	54,78917	0,26	0	0,26	0	0	1		9745,465			1		1				305412,16	Chromodomain-helicase-DNA-binding protein 6 OS=Homo sapiens OX=9606 GN=CHD6 PE=1 SV=4
707	Q9NZV1|CRI	92,84943	1,45	0	0	0	1,45	2				170926,47	2				3		113738,125	Cysteine-rich motor neuron 1 protein OS=Homo sapiens OX=9606 GN=CRIM1 PE=1 SV=1
376	Q96KK5|H2A	279,6636	38,28	11,72	12,5	7,03	38,28	6	259248,44	294746,4	250080,11	2714422,5	6	4	6	2	11		13906,251	Histone H2A type 1-H OS=Homo sapiens OX=9606 GN=H2AC12 PE=1 SV=3
1054	Q9Y287|ITM2	64,69755	3,76	0	0	0	3,76	1				615986,6	1				2		30338,035	Integral membrane protein 2B OS=Homo sapiens OX=9606 GN=ITM2B PE=1 SV=1
53	P43652|AFA	96,63606	1,17	1,17	0	0	0	1	83501,875				1	1					69069,11	Afamin OS=Homo sapiens OX=9606 GN=AFM PE=1 SV=1
17	A6NGB9|WIP	55,47955	1,24	1,24	1,24	0	0	1	1735353,4	453682,16			1	3	3				49457,64	WAS/WASL-interacting protein family member 3 OS=Homo sapiens OX=9606 GN=WIPF3 PE=1 SV=4
413	Q16695|H31	118,3275	23,53	4,41	4,41	5,15	23,53	5	340159,38	464861,53	729542	5116208	4	1	1	1	7	Oxidation	15508,248	Histone H3.1t OS=Homo sapiens OX=9606 GN=H3-4 PE=1 SV=3
1	Q9H156|SLIK	100,1838	0,71	0,71	0,71	0,71	0,71	1	1291231,8	600428,8	2056739,1	2,87E+07	1	2	2	1	2		95404,4	SLIT and NTRK-like protein 2 OS=Homo sapiens OX=9606 GN=SLITRK2 PE=1 SV=1
165	P07686|HEXE	367,9767	24,46	18,53	20,32	0	0	15	4244166	1992031,2			13	23	26			Carbamid	63137,414	Beta-hexosaminidase subunit beta OS=Homo sapiens OX=9606 GN=HEXB PE=1 SV=4
1055	P46821|MAP	46,86472	0,32	0,32	0	0	0	1	38988,316				1	1					270634,4	Microtubule-associated protein 1B OS=Homo sapiens OX=9606 GN=MAP1B PE=1 SV=2
16	Q9Y6R7|FCGI	70,16532	0,11	0	0	0,11	0,11	1			93377,58	72732,484	1			1	1		572016,7	IgGFc-binding protein OS=Homo sapiens OX=9606 GN=FCGBP PE=1 SV=3
692	P08151|GLI1_	29,85037	0,54	0,54	0	0	0	1	62285,676				1	1					117904,47	Zinc finger protein GLI1 OS=Homo sapiens OX=9606 GN=GLI1 PE=1 SV=1
414	Q9Y646|CBP	196,2447	10,59	8,26	6,57	0	0	5	131133,17	69675,055			5	5	3			Carbamid	51887,56	Carboxypeptidase Q OS=Homo sapiens OX=9606 GN=CPQ PE=1 SV=1
483	Q9H300|PAR	34,82591	1,58	0	1,58	0	1,58	1		403608,97		70899,88	1		1		1		42190,027	Presenilins-associated rhomboid-like protein, mitochondrial OS=Homo sapiens OX=9606 GN=PARL PE=1 SV=2
6	Q15532|SSXT	38,92721	1,44	0	0	1,44	1,44	1			151096,02	92072,516	1			2	1		45929,188	Protein SSXT OS=Homo sapiens OX=9606 GN=SS18 PE=1 SV=3
171	O75093|SLIT	185,21694	0,39	0	0	0	0,39	1				314946,9	1				2		167926,03	Slit homolog 1 protein OS=Homo sapiens OX=9606 GN=SLIT1 PE=1 SV=4
547	P60900|PSA	108,5783	11,79	3,66	0	8,13	3,25	3	17598,793				3	1		2	1	Carbamid	27399,445	Proteasome subunit alpha type-6 OS=Homo sapiens OX=9606 GN=PSMA6 PE=1 SV=1
104	P54687|BCAT	90,14129	1,55	1,55	1,55	1,55	1,55	1	272529,6		100229,48	94495,805	1	2	1	1	1		42966,145	Branched-chain-amino-acid aminotransferase, cytosolic OS=Homo sapiens OX=9606 GN=BCAT1 PE=1 SV=3
99	P49674|KC1E	78,47889	1,44	1,44	1,44	0	0	1	59342,84	35403,457			1	1	1				47315,082	Casein kinase I isoform epsilon OS=Homo sapiens OX=9606 GN=CSNK1E PE=1 SV=1
6	P33897|ABCD	76,21806	0,81	0,81	0	0,81	0,81	1	95524,04		1177229,9	711697,1	1	1		2	1		82936,92	ATP-binding cassette sub-family D member 1 OS=Homo sapiens OX=9606 GN=ABCD1 PE=1 SV=2
987	Q9Y520|PRC	66,54484	0,21	0	0,21	0	0,21	1		24584,418		165526,06	1		1		2		316911,12	Protein PRRC2C OS=Homo sapiens OX=9606 GN=PRRC2C PE=1 SV=4
196	Q9Y232|CDYL	74,10828	1	0	0	1	1	1			436587,1	770382	1			1	1		66481,67	Chromodomain Y-like protein OS=Homo sapiens OX=9606 GN=CDYL PE=1 SV=2
77	Q9NPP4|NLR	74,67117	0,59	0,59	0,59	0	0	1	324550,38	52481,477			1	1	1				116159,09	NLR family CARD domain-containing protein 4 OS=Homo sapiens OX=9606 GN=NLRC4 PE=1 SV=2
312	P63096|GNAI	234,3827	10,45	0	0	8,76	10,45	5			149788,33	162786,88	1			7	7		40361,08	Guanine nucleotide-binding protein G(i) subunit alpha-1 OS=Homo sapiens OX=9606 GN=GNAI1 PE=1 SV=2
1056	Q5FYB0|ARS	28,58745	1	0	1	0	0	1		20771,395			1		1				67235,445	Arylsulfatase J OS=Homo sapiens OX=9606 GN=ARSJ PE=2 SV=1
377	Q96RW7|HM	181,5761	0,89	0,78	0,32	0	0	6	1031947,4				5	7	3			Carbamid	613389,56	Hemicentin-1 OS=Homo sapiens OX=9606 GN=HMCN1 PE=1 SV=2
708	O00159|MYO	87,95969	2,07	0	0	2,07	0	2					1			2			121681,67	Unconventional myosin-Ic OS=Homo sapiens OX=9606 GN=MYO1C PE=1 SV=4
250	Q15406|NR6	68,71788	1,25	0	0	0	1,25	1				184185,14	1				1		54382,93	Nuclear receptor subfamily 6 group A member 1 OS=Homo sapiens OX=9606 GN=NR6A1 PE=1 SV=2
309	P02462|CO4	262,7341	3	1,5	1,5	0,72	0,72	8				19222,78	1	11	9	4	4		160610,77	Collagen alpha-1(IV) chain OS=Homo sapiens OX=9606 GN=COL4A1 PE=1 SV=4
1057	Q9UKA4|AKA	30,05671	0,32	0	0	0,32	0	1			24439,955		1			1		Deamidat	210512,22	A-kinase anchor protein 11 OS=Homo sapiens OX=9606 GN=AKAP11 PE=1 SV=1
548	P09874|PARI	69,85248	2,66	0	0	0,59	2,07	3				229625,56	2			2	2		113083,79	Poly [ADP-ribose] polymerase 1 OS=Homo sapiens OX=9606 GN=PARP1 PE=1 SV=4
1058	Q96KE9|BTB	30,21216	1,12	0	0	0	1,12	1					1				1		58776,277	BTB/POZ domain-containing protein 6 OS=Homo sapiens OX=9606 GN=BTBD6 PE=1 SV=4
166	P60174|TPIS_	372,0751	70,28	67,47	30,92	14,06	24,1	15	4860393,5	1445484,6	188700,34	234861,9	15	36	16	3	4	Carbamid	26669,484	Triosephosphate isomerase OS=Homo sapiens OX=9606 GN=TPI1 PE=1 SV=4
210	Q05682|CALD	352,9566	16,52	16,52	1,64	3,28	0	12	2312707,5	14272,522	71969,84		12	15	1	3			93231,08	Caldesmon OS=Homo sapiens OX=9606 GN=CALD1 PE=1 SV=3
988	P41091|IF2G	29,20635	1,27	0	0	1,27	0	1			88342,03		1			1			51109,492	Eukaryotic translation initiation factor 2 subunit 3 OS=Homo sapiens OX=9606 GN=EIF2S3 PE=1 SV=3
66	Q8IZD0|SAM	110,3961	1,44	0	0	1,44	1,44	1			1289964,5	1255841,4	1			2	2	Carbamid	45055,88	Sterile alpha motif domain-containing protein 14 OS=Homo sapiens OX=9606 GN=SAMD14 PE=2 SV=2
710	Q6ZRI0|OTO	65,32408	0,41	0,21	0	0,21	0	2					1	1		1			314793,8	Otogelin OS=Homo sapiens OX=9606 GN=OTOG PE=1 SV=3
1059	Q969G5|CAV	26,05076	2,3	0	0	2,3	2,3	1			28698,484	80936,39	1			1	1		27701,162	Caveolae-associated protein 3 OS=Homo sapiens OX=9606 GN=CAVIN3 PE=1 SV=3
711	Q96AM1|MR	164,135	5,83	0	0	0	5,83	2				287979,9	2				4	Carbamid	38171,445	Mas-related G-protein coupled receptor member F OS=Homo sapiens OX=9606 GN=MRGPRF PE=2 SV=1
4	O00257|CBX	98,53243	1,07	1,07	1,07	1,07	0	1	44638,28	49256,445	45390,445		1	2	2	1			61367,51	E3 SUMO-protein ligase CBX4 OS=Homo sapiens OX=9606 GN=CBX4 PE=1 SV=3
444	Q6PML9|ZNT	77,08476	1,06	1,06	0	0	0	1	116446,97				1	1					63515,11	Proton-coupled zinc antiporter SLC30A9, mitochondrial OS=Homo sapiens OX=9606 GN=SLC30A9 PE=1 SV=1
687	Q96HD1|CRE	38,66667	1,43	0	1,43	0	0	1		58114,875			1		1				45439,523	Protein disulfide isomerase CRELD1 OS=Homo sapiens OX=9606 GN=CRELD1 PE=1 SV=3
331	O43464|HTR	114,9023	2,18	2,18	0	0	0	1	78726,67				1	3					48840,848	Serine protease HTRA2, mitochondrial OS=Homo sapiens OX=9606 GN=HTRA2 PE=1 SV=2
119	Q7KZ85|SPT6	88,07253	0,35	0	0	0,35	0,35	1			119086,47	115492,39	1			1	1		199072,75	Transcription elongation factor SPT6 OS=Homo sapiens OX=9606 GN=SUPT6H PE=1 SV=2
712	Q9NSI6|BRW	47,15541	0,6	0	0,26	0	0,34	2		56877,754		1760109	2		2		2	Acetylatic	262936,1	Bromodomain and WD repeat-containing protein 1 OS=Homo sapiens OX=9606 GN=BRWD1 PE=1 SV=4
476	O43897|TLL1_	84,0716	1,28	1,28	0	0	0	1					1	1					114708,7	Tolloid-like protein 1 OS=Homo sapiens OX=9606 GN=TLL1 PE=1 SV=1
182	Q13509|TBB	291,9334	17,11	0	0	17,11	6,22	7			877483,9	189163	7			11	4	Oxidation	50432,676	Tubulin beta-3 chain OS=Homo sapiens OX=9606 GN=TUBB3 PE=1 SV=2
90	P04406|G3P_	428,2024	61,49	17,91	4,78	61,49	54,93	21	429892,62	47486,656	1,40E+07	9470545	20	8	2	58	40	Deamidat	36053,207	Glyceraldehyde-3-phosphate dehydrogenase OS=Homo sapiens OX=9606 GN=GAPDH PE=1 SV=3
174	Q15937|ZNF	48,87747	1,2	0	0	1,2	0	1			47665,32		1			1			55349,96	Zinc finger protein 79 OS=Homo sapiens OX=9606 GN=ZNF79 PE=1 SV=2
87	Q4V328|GRA	40,01609	0,71	0	0	0,71	0,71	1			127495,2	284008,75	1			1	1	Acetylatic	96005,55	GRIP1-associated protein 1 OS=Homo sapiens OX=9606 GN=GRIPAP1 PE=1 SV=2
311	P36222|CH3L	297,302	19,06	19,06	3,92	0	0	8	791010,3	19050,924			8	14	2				42625,465	Chitinase-3-like protein 1 OS=Homo sapiens OX=9606 GN=CHI3L1 PE=1 SV=2
468	Q6NSI3|FA53	22,78029	1,51	0	0	0	1,51	1				1443703,5	1				2		42585,387	Protein FAM53A OS=Homo sapiens OX=9606 GN=FAM53A PE=1 SV=1
1060	P49767|VEGF	50,26862	1,91	1,91	0	0	0	1	38629				1	1					46883,367	Vascular endothelial growth factor C OS=Homo sapiens OX=9606 GN=VEGFC PE=1 SV=1
456	P60903|S10A	177,4659	26,8	10,31	0	26,8	17,53	4	125824,77		3103266	356827,56	4	4		9	5	Carbamid	11203,1	Protein S100-A10 OS=Homo sapiens OX=9606 GN=S100A10 PE=1 SV=2
475	A6NC98|CC8	25,13488	0,41	0	0	0	0,41	1				2624083,2	1				1		164809,12	Coiled-coil domain-containing protein 88B OS=Homo sapiens OX=9606 GN=CCDC88B PE=1 SV=1
407	Q5HYK7|SH3	116,4291	0,76	0	0	0	0,76	1				98768,42	1				1		86525,42	SH3 domain-containing protein 19 OS=Homo sapiens OX=9606 GN=SH3D19 PE=1 SV=2
103	O43707|ACTI	426,2782	26,45	21,95	9,55	6,81	1,32	26	490320,66	188953,98	35440,49		11	40	15	8	2	Carbamid	104854,04	Alpha-actinin-4 OS=Homo sapiens OX=9606 GN=ACTN4 PE=1 SV=2
715	Q9P1Y5|CAM	67,14017	0,96	0,48	0,48	0	0,48	2					1	1	1		1		134749,66	Calmodulin-regulated spectrin-associated protein 3 OS=Homo sapiens OX=9606 GN=CAMSAP3 PE=1 SV=2
254	Q9UBR2|CAT	319,4087	27,06	27,06	25,41	0	3,96	9	5648179,5	4966466		78966,38	9	22	22		1	Carbamid	33867,926	Cathepsin Z OS=Homo sapiens OX=9606 GN=CTSZ PE=1 SV=1
182	A6NNZ2|TBB	229,2762	9,91	0	0	9,91	5,41	5			459862,12	176848,97	5			6	4	Oxidation	49572,61	Tubulin beta 8B OS=Homo sapiens OX=9606 GN=TUBB8B PE=1 SV=1
206	A0A0B4J2A2|	269,5659	28,05	11,59	11,59	28,05	28,05	6	212291,48	104125,625	604586,06	671093,94	4	3	3	9	8	Carbamid	18155,754	Peptidyl-prolyl cis-trans isomerase A-like 4C OS=Homo sapiens OX=9606 GN=PPIAL4C PE=2 SV=1
503	Q8TF50|ZN5	246,7746	0,9	0	0	0	0,9	1					1				1		73621,81	Zinc finger protein 526 OS=Homo sapiens OX=9606 GN=ZNF526 PE=1 SV=2
1061	Q7Z7E8|UB2	28,85608	1,42	0	0	1,42	0	1			67875,92		1			1			46126,906	Ubiquitin-conjugating enzyme E2 Q1 OS=Homo sapiens OX=9606 GN=UBE2Q1 PE=1 SV=1
70	Q13948|CASI	66,2057	0,88	0,88	0	0	0	1	56448,73				1	1				Deamidat	77455,3	Protein CASP OS=Homo sapiens OX=9606 GN=CUX1 PE=1 SV=2
126	P12107|COB	371,2466	7,81	5,98	3,82	1,44	1,55	21					3	27	18	9	8	Acetylatic	181064,83	Collagen alpha-1(XI) chain OS=Homo sapiens OX=9606 GN=COL11A1 PE=1 SV=4
1062	O60318|GAN	31,49678	0,3	0,3	0	0,3	0	1	100652,836				1	1		1			218404,64	Germinal-center associated nuclear protein OS=Homo sapiens OX=9606 GN=MCM3AP PE=1 SV=2
652	Q99466|NOT	39,36704	0,3	0	0	0	0,3	1				41473,586	1				1		209621,84	Neurogenic locus notch homolog protein 4 OS=Homo sapiens OX=9606 GN=NOTCH4 PE=1 SV=2
1063	Q14558|KPR	24,44333	1,69	0	0	1,69	0	1			245290,78		1			1			39393,71	Phosphoribosyl pyrophosphate synthase-associated protein 1 OS=Homo sapiens OX=9606 GN=PRPSAP1 PE=1 SV=2
128	Q6Q8B3|MO	79,71534	2,21	0	0	2,21	2,21	1			45551,754	49995,15	1			1	1	Carbamid	29919,764	Cell surface glycoprotein CD200 receptor 2 OS=Homo sapiens OX=9606 GN=CD200R1L PE=1 SV=2
256	Q9Y2D1|ATF	62,79268	2,13	0	2,13	0	0	1		145790,38			1		2				30674,252	Cyclic AMP-dependent transcription factor ATF-5 OS=Homo sapiens OX=9606 GN=ATF5 PE=1 SV=4
549	P38606|VAT	92,98253	3,73	0	0	2,27	2,43	3				50714,953	2			2	3		68304,08	V-type proton ATPase catalytic subunit A OS=Homo sapiens OX=9606 GN=ATP6V1A PE=1 SV=2
458	P06703|S10A	113,9967	24,44	24,44	24,44	24,44	24,44	3	1,05E+07	2997581,2	3520250,5	3077557	2	9	11	9	7	Oxidation	10179,734	Protein S100-A6 OS=Homo sapiens OX=9606 GN=S100A6 PE=1 SV=1
1050	B9A064|IGLL	87,25446	2,8	2,8	0	0	0	1					1	1					23063,24	Immunoglobulin lambda-like polypeptide 5 OS=Homo sapiens OX=9606 GN=IGLL5 PE=2 SV=2
378	P60660|MYL6	280,438	39,07	39,07	0	39,07	0	6	257065,27		268069,47		4	9		7		Carbamid	16930,045	Myosin light polypeptide 6 OS=Homo sapiens OX=9606 GN=MYL6 PE=1 SV=2
177	P68104|EF1A	369,0201	28,79	8,23	0	28,79	14,72	15	528966,6		3656404	2602554,5	15	6		25	12		50140,86	Elongation factor 1-alpha 1 OS=Homo sapiens OX=9606 GN=EEF1A1 PE=1 SV=1
70	O15550|KDM	83,96447	0,43	0	0	0,43	0,43	1			64976,27	250123	1			3	1		154176,53	Lysine-specific demethylase 6A OS=Homo sapiens OX=9606 GN=KDM6A PE=1 SV=2
1064	Q8IYW2|CFA	26,49577	0,22	0	0	0,22	0	1			60916,53		1			1			303500,3	Cilia- and flagella-associated protein 46 OS=Homo sapiens OX=9606 GN=CFAP46 PE=2 SV=3
415	O14556|G3P	160,0943	5,39	0	0	5,39	1,72	3			515970,62		2			10	3	Oxidation	44501,152	Glyceraldehyde-3-phosphate dehydrogenase, testis-specific OS=Homo sapiens OX=9606 GN=GAPDHS PE=1 SV=2
255	Q14993|COJ	225,1815	5,34	3,5	3,24	2,28	0,53	10	1368863,1	344897,47	99000,65	236152,39	3	8	5	4	1		115220,734	Collagen alpha-1(XIX) chain OS=Homo sapiens OX=9606 GN=COL19A1 PE=1 SV=3
235	F2Z3M2|CQ1	94,14457	5,26	5,26	5,26	0	0	1	229314,33	22136,396			1	1	3				12667,54	Uncharacterized protein encoded by LINC02876 OS=Homo sapiens OX=9606 GN=LINC02876 PE=5 SV=1
1065	Q02543|RL18	37,54115	4,55	0	0	0	4,55	1				42340,125	1				1		20762,352	60S ribosomal protein L18a OS=Homo sapiens OX=9606 GN=RPL18A PE=1 SV=2
416	P35613|BASI	256,5216	11,43	0	0	11,43	11,43	5			108173,43	791026,2	5			5	7		42200,363	Basigin OS=Homo sapiens OX=9606 GN=BSG PE=1 SV=2
1024	Q2M296|MTI	22,81436	1,57	0	0	1,57	0	1					1			1		Carbamid	42172,645	Methenyltetrahydrofolate synthase domain-containing protein OS=Homo sapiens OX=9606 GN=MTHFSD PE=1 SV=2
673	Q8TD57|DYH	33,06132	0,15	0	0	0	0,15	1				1253976	1				1	Deamidat	470771,16	Dynein axonemal heavy chain 3 OS=Homo sapiens OX=9606 GN=DNAH3 PE=2 SV=1
132	P06753|TPM	365,3358	42,81	42,81	24,91	9,47	4,56	19	58050,05				1	38	17	4	2	Deamidat	32949,973	Tropomyosin alpha-3 chain OS=Homo sapiens OX=9606 GN=TPM3 PE=1 SV=2
1066	P36021|MOT	29,33739	2,04	0	0	0	2,04	1				46830,2	1				1	Acetylatic	59511,406	Monocarboxylate transporter 8 OS=Homo sapiens OX=9606 GN=SLC16A2 PE=1 SV=2
1067	Q05397|FAK	24,35381	0,57	0	0	0,57	0	1			37122,6		1			1			119233,01	Focal adhesion kinase 1 OS=Homo sapiens OX=9606 GN=PTK2 PE=1 SV=2
717	P54709|AT1B	64,93613	5,02	0	0	0	5,02	2				55576,492	2				2	Carbamid	31512,578	Sodium/potassium-transporting ATPase subunit beta-3 OS=Homo sapiens OX=9606 GN=ATP1B3 PE=1 SV=1
441	P05156|CFAI	109,4496	1,37	1,37	0	0	0	1	515851,16				1	2				Carbamid	65750,25	Complement factor I OS=Homo sapiens OX=9606 GN=CFI PE=1 SV=2
1068	Q8WWY7|WI	36,74178	9,01	0	0	0	9,01	1				160698,9	1				1	Carbamid	12049,853	WAP four-disulfide core domain protein 12 OS=Homo sapiens OX=9606 GN=WFDC12 PE=1 SV=1
6	Q9HCK1|ZDB	38,19467	0,25	0	0	0,25	0,25	1			109276,72	136783,45	1			1	1		265617,75	DBF4-type zinc finger-containing protein 2 OS=Homo sapiens OX=9606 GN=ZDBF2 PE=1 SV=3
1011	Q3BBV2|NBP	41,75044	0,69	0	0	0,69	0,69	1			5235523	7,28E+07	1			2	4		99214,64	Putative neuroblastoma breakpoint family member 8 OS=Homo sapiens OX=9606 GN=NBPF8 PE=5 SV=1
282	P19883|FST_	329,4435	26,16	26,16	0	0	0	9	810947,1				9	13				Carbamid	38007,176	Follistatin OS=Homo sapiens OX=9606 GN=FST PE=1 SV=2
283	Q14533|KRT8	305,0909	11,29	1,39	1,39	6,34	8,91	8			185751,38	356090,03	6	2	2	8	9	Carbamid	54928,277	Keratin, type II cuticular Hb1 OS=Homo sapiens OX=9606 GN=KRT81 PE=1 SV=3
85	P30101|PDIA	444,8163	49,11	49,11	28,91	6,34	0	28	1,13E+07	822743,8	64487,766		24	63	25	4		Deamidat	56782,383	Protein disulfide-isomerase A3 OS=Homo sapiens OX=9606 GN=PDIA3 PE=1 SV=4
99	Q9P109|GCN	132,711	1,55	1,55	1,55	0	0	2	426734,2	235827,72			1	3	3				53052,23	Beta-1,3-galactosyl-O-glycosyl-glycoprotein beta-1,6-N-acetylglucosaminyltransferase 4 OS=Homo sapiens OX=9606 GN=GCNT4 PE=2 SV=1
551	P30040|ERP2	158,4167	11,11	11,11	0	0	0	3	932502,1				3	6					28993,428	Endoplasmic reticulum resident protein 29 OS=Homo sapiens OX=9606 GN=ERP29 PE=1 SV=4
256	O00300|TR11	223,241	17,46	0	17,46	0	0	10		900152,4			10		11				46026,016	Tumor necrosis factor receptor superfamily member 11B OS=Homo sapiens OX=9606 GN=TNFRSF11B PE=1 SV=3
1069	Q8WXE9|STC	28,22043	0,66	0	0	0	0,66	1				2043663,5	1				1		101165,09	Stonin-2 OS=Homo sapiens OX=9606 GN=STON2 PE=1 SV=1

255	Q7RTS9|DYM	52,44891	0,9	0,9	0,9	0,9	0,9	1				236152,39	1	1	1	1	1		75935,45	Dymeclin OS=Homo sapiens OX=9606 GN=DYM PE=1 SV=1
719	P69905|HBA_	144,126	11,27	0	0	0	11,27	2				548665	2				5	Oxidation	15257,544	Hemoglobin subunit alpha OS=Homo sapiens OX=9606 GN=HBA1 PE=1 SV=2
809	Q14397|GCKI	44,9006	0,96	0	0,96	0	0	1		222599,11			1		2				68685,26	Glucokinase regulatory protein OS=Homo sapiens OX=9606 GN=GCKR PE=1 SV=6
1026	Q99805|TM9:	35,52444	0,9	0	0	0	0,9	1				63282,344	1				1		75775,664	Transmembrane 9 superfamily member 2 OS=Homo sapiens OX=9606 GN=TM9SF2 PE=1 SV=1
960	P35611|ADD/	27,65908	0,81	0	0	0,81	0	1			223699,64		1			1			80955,13	Alpha-adducin OS=Homo sapiens OX=9606 GN=ADD1 PE=1 SV=2
1063	Q9UQ10|DHD	24,44333	1,8	0	0	1,8	0	1			245290,78		1			1			36381,98	Trans-1,2-dihydrobenzene-1,2-diol dehydrogenase OS=Homo sapiens OX=9606 GN=DHDH PE=1 SV=1
13	Q70E73|RAPI	29,92118	0,48	0	0	0,48	0	1			98454,58		1			1			135255,89	Ras-associated and pleckstrin homology domains-containing protein 1 OS=Homo sapiens OX=9606 GN=RAPH1 PE=1 SV=3
10	P43489|TNR4	26,08867	2,17	2,17	0	2,17	0	1					1	1		1			29340,506	Tumor necrosis factor receptor superfamily member 4 OS=Homo sapiens OX=9606 GN=TNFRSF4 PE=1 SV=1
721	Q99584|S10A	70,25294	19,39	8,16	19,39	0	11,22	2	121625,7	67231,836			2	1	2		1		11471,187	Protein S100-A13 OS=Homo sapiens OX=9606 GN=S100A13 PE=1 SV=1
93	Q8WV16|DC/	96,03992	1,21	1,21	1,21	1,21	1,21	1			42627,023	262097,48	1	1	1	1	1		55693,504	DDB1- and CUL4-associated factor 4 OS=Homo sapiens OX=9606 GN=DCAF4 PE=1 SV=3
605	P01877|IGHA	75,26572	4,09	0	0	2,3	4,09	2			112046,2	176871,72	2			1	2	Carbamid	42333,926	Immunoglobulin heavy constant alpha 2 OS=Homo sapiens OX=9606 GN=IGHA2 PE=1 SV=5
47	P14923|PLAK	486,8312	53,29	0	0	48,46	47,11	35			1,18E+07	9755011	29			76	63	Oxidation	81744,74	Junction plakoglobin OS=Homo sapiens OX=9606 GN=JUP PE=1 SV=3
198	Q71U36|TBA:	344,4702	29,93	6,65	3,33	29,93	15,3	10	3913,0781		681043,4		2	3	1	14	6	Deamidat	50135,63	Tubulin alpha-1A chain OS=Homo sapiens OX=9606 GN=TUBA1A PE=1 SV=1
1070	Q8IXJ9|ASXL	24,50114	0,39	0	0,39	0	0	1					1		1				165431,72	Polycomb group protein ASXL1 OS=Homo sapiens OX=9606 GN=ASXL1 PE=1 SV=4
1071	Q9NX08|CON	27,42501	3,28	0	3,28	0	0	1					1		1				21090,361	COMM domain-containing protein 8 OS=Homo sapiens OX=9606 GN=COMMD8 PE=1 SV=1
231	Q9UBX5|FBLI	330,8541	22,1	22,1	11,83	2,01	5,13	11	2492674,2	556336,3	12786,109	81446,234	11	14	9	1	3	Carbamid	50180,484	Fibulin-5 OS=Homo sapiens OX=9606 GN=FBLN5 PE=1 SV=1
137	P50993|AT1A	293,3419	7,55	0	0	0,78	7,55	7				238548,84	5			1	7	Carbamid	112265,44	Sodium/potassium-transporting ATPase subunit alpha-2 OS=Homo sapiens OX=9606 GN=ATP1A2 PE=1 SV=1
211	P23528|COF1	342,107	62,65	26,51	0	56,02	24,1	12	1,55E+07		2665092,8	2094957	8	6		13	5	Acetylatic	18502,488	Cofilin-1 OS=Homo sapiens OX=9606 GN=CFL1 PE=1 SV=3
966	Q8N8D1|PDC	28,72969	1,24	0	0	0	1,24	1				2,36E+07	1				2		54699,98	Programmed cell death protein 7 OS=Homo sapiens OX=9606 GN=PDCD7 PE=1 SV=1
552	Q68BL8|OLM	140,1163	3,33	0	3,33	0	0,93	3		104346,64		277422,03	3		4		1		83998,914	Olfactomedin-like protein 2B OS=Homo sapiens OX=9606 GN=OLFML2B PE=2 SV=2
606	P15531|NDK/	115,2967	19,08	19,08	0	0	0	2	19049,527				2	3					17148,72	Nucleoside diphosphate kinase A OS=Homo sapiens OX=9606 GN=NME1 PE=1 SV=1
186	P63104|1433	346,5358	44,9	37,55	21,63	36,33	26,53	12	1439952,5	341147,72	488235,44	123433,66	6	18	12	13	9	Carbamid	27745,1	14-3-3 protein zeta/delta OS=Homo sapiens OX=9606 GN=YWHAZ PE=1 SV=1
1072	Q7Z7N9|T179	27,34611	2,74	0	0	0	2,74	1				132547,64	1				1		23550,396	Transmembrane protein 179B OS=Homo sapiens OX=9606 GN=TMEM179B PE=1 SV=1
1073	P23381|SYW(	42,31266	1,49	0	0	1,49	0	1					1			1			53165,414	Tryptophan--tRNA ligase, cytoplasmic OS=Homo sapiens OX=9606 GN=WARS1 PE=1 SV=2
379	Q13443|ADA	260,3599	7,2	1,71	0	0	5,49	6	4478,9185			1908625,5	6	1			5	Carbamid	90555,98	Disintegrin and metalloproteinase domain-containing protein 9 OS=Homo sapiens OX=9606 GN=ADAM9 PE=1 SV=1
380	P61916|NPC2	269,4119	36,42	36,42	36,42	0	5,96	6	4887741,5	3931406,5		127486,61	6	12	15		1	Carbamid	16570,205	NPC intracellular cholesterol transporter 2 OS=Homo sapiens OX=9606 GN=NPC2 PE=1 SV=1
256	P29558|RBMS	59,05715	1,48	0	1,48	0	0	1					1		1				44505,297	RNA-binding motif, single-stranded-interacting protein 1 OS=Homo sapiens OX=9606 GN=RBMS1 PE=1 SV=3
495	B1AL46|NTM	53,03056	0,68	0	0	0	0,68	1				29151,854	1				1	Deamidat	93979	NUT family member 2E OS=Homo sapiens OX=9606 GN=NUTM2E PE=3 SV=3
1074	Q9UF56|FXL1	29,53156	0,86	0	0	0,86	0	1			11944,6		1			1			75694,99	F-box/LRR-repeat protein 17 OS=Homo sapiens OX=9606 GN=FBXL17 PE=1 SV=3
1075	P08294|SODI	23,1155	2,5	0	2,5	0	0	1					1		1				25850,908	Extracellular superoxide dismutase [Cu-Zn] OS=Homo sapiens OX=9606 GN=SOD3 PE=1 SV=2
475	Q9UHD4|CID	25,13488	2,74	0	0	0	2,74	1				2624083,2	1				1		24678,209	Lipid transferase CIDEB OS=Homo sapiens OX=9606 GN=CIDEB PE=1 SV=2
1076	Q96Q06|PLIN	28,2948	0,51	0	0	0,51	0	1					1			1			135892	Perilipin-4 OS=Homo sapiens OX=9606 GN=PLIN4 PE=1 SV=3
417	Q5T750|XP32	266,5855	21,2	0	0	21,2	18,4	5			2334689,5	1719746,8	5			11	6	Carbamid	26238,035	Skin-specific protein 32 OS=Homo sapiens OX=9606 GN=XP32 PE=1 SV=1
233	O60942|MCE	33,12619	1,01	1,01	0	0	0	1	278157,72				1	1					68556,73	mRNA-capping enzyme OS=Homo sapiens OX=9606 GN=RNGTT PE=1 SV=1
1077	C9J7I0|UMAD	108,5724	10,22	0	0	0	10,22	1				62832,74	1				1		15199,782	UBAP1-MVB12-associated (UMA)-domain containing protein 1 OS=Homo sapiens OX=9606 GN=UMAD1 PE=1 SV=2
342	P19823|ITIH2	254,4683	4,86	4,86	0,63	2,33	2,33	7	968834,6		197958,06	125416,39	6	8	1	4	4		106463,47	Inter-alpha-trypsin inhibitor heavy chain H2 OS=Homo sapiens OX=9606 GN=ITIH2 PE=1 SV=2
108	Q9BZ29|DOC	60,04453	0,29	0,29	0	0	0	1	544477,3				1	1					236445,62	Dedicator of cytokinesis protein 9 OS=Homo sapiens OX=9606 GN=DOCK9 PE=1 SV=2
553	O15230|LAM	80,13606	0,49	0,16	0,32	0	0	3					1	1	3				399737,22	Laminin subunit alpha-5 OS=Homo sapiens OX=9606 GN=LAMA5 PE=1 SV=8
119	Q8NHV4|NEI	77,93327	1,06	0	1,06	1,06	1,06	1			428770,06	691625,1	1		1	1	1		71966,086	Protein NEDD1 OS=Homo sapiens OX=9606 GN=NEDD1 PE=1 SV=1
91	Q12841|FSTL	415,7506	54,55	52,27	38,96	0	9,74	22	4,91E+07	9666246		105761,305	21	74	53		4	Carbamid	34985,508	Follistatin-related protein 1 OS=Homo sapiens OX=9606 GN=FSTL1 PE=1 SV=1
182	Q9BUF5|TBB(	247,7385	11,21	0	0	11,21	3,59	5			473598,38	176848,97	5			6	3	Oxidation	49857,1	Tubulin beta-6 chain OS=Homo sapiens OX=9606 GN=TUBB6 PE=1 SV=1
283	Q5QJU3|ACE	40,32876	2,18	0	0	2,18	0	1			115794,38		1			1			31309,21	Alkaline ceramidase 2 OS=Homo sapiens OX=9606 GN=ACER2 PE=1 SV=2
1078	A4D0S4|LAM	24,02705	0,34	0	0,34	0	0	1		98101			1		1				193539,73	Laminin subunit beta-4 OS=Homo sapiens OX=9606 GN=LAMB4 PE=2 SV=1
634	Q96HR3|MED	71,26441	3,37	0	3,37	3,37	3,37	1		4961949	219925,28	3924922	1		11	8	1	Deamidat	20277,229	Mediator of RNA polymerase II transcription subunit 30 OS=Homo sapiens OX=9606 GN=MED30 PE=1 SV=1
1079	Q86XE0|SNX:	24,10124	1,49	0	0	1,49	0	1			223339,5		1			1		Deamidat	46398,867	Sorting nexin-32 OS=Homo sapiens OX=9606 GN=SNX32 PE=1 SV=1
1045	O15119|TBX3	29,49904	0,81	0,81	0	0	0	1					1	1					79388,805	T-box transcription factor TBX3 OS=Homo sapiens OX=9606 GN=TBX3 PE=1 SV=4
1044	Q9UM73|ALK	40,36036	0,37	0	0	0,37	0	1			63496,605		1			1			176441,94	ALK tyrosine kinase receptor OS=Homo sapiens OX=9606 GN=ALK PE=1 SV=3
973	Q96EM0|T3H	59,688	1,69	0	0	0	1,69	1				96269,87	1				1		38137,74	Trans-3-hydroxy-L-proline dehydratase OS=Homo sapiens OX=9606 GN=L3HYPDH PE=1 SV=2
220	A8K855|EFCB	97,98536	0,95	0	0	0,95	0,95	1			74041,9	72786,56	1			1	1		71981,29	EF-hand calcium-binding domain-containing protein 7 OS=Homo sapiens OX=9606 GN=EFCAB7 PE=1 SV=1
7	Q99715|COC.	672,9036	49,72	37,19	23,34	36,6	48,51	161	4,33E+07	1,02E+07	3,38E+07	2,24E+08	156	248	170	211	473	Oxidation	333146,75	Collagen alpha-1(XII) chain OS=Homo sapiens OX=9606 GN=COL12A1 PE=1 SV=2
24	P49747|COM	534,9579	71,99	68,43	37,65	57,6	28,27	52	1,72E+08	1,05E+07	2,83E+07	2633433,8	49	240	68	113	27	Deamidat	82860,484	Cartilage oligomeric matrix protein OS=Homo sapiens OX=9606 GN=COMP PE=1 SV=2
418	P26447|S10A	200,1854	35,64	35,64	24,75	0	15,84	4	2100338,5	912854,4		121406,39	4	9	7		2	Oxidation	11728,511	Protein S100-A4 OS=Homo sapiens OX=9606 GN=S100A4 PE=1 SV=1
723	Q9P266|JCAI	57,37908	0,88	0	0,44	0	0,44	2				53598,746	1		1		1		148350,8	Junctional cadherin 5-associated protein OS=Homo sapiens OX=9606 GN=JCAD PE=1 SV=3
1080	P49848|TAF6	33,03733	0,89	0	0	0,89	0	1			176723		1			1			72668,44	Transcription initiation factor TFIID subunit 6 OS=Homo sapiens OX=9606 GN=TAF6 PE=1 SV=1
724	Q53TN4|CYBI	149,8656	7,69	0	0	7,69	0	2			277933,56		2			2			31641,307	Plasma membrane ascorbate-dependent reductase CYBRD1 OS=Homo sapiens OX=9606 GN=CYBRD1 PE=1 SV=1
1081	Q99683|M3K	23,83986	0,44	0	0	0,44	0	1					1			1			154536,92	Mitogen-activated protein kinase kinase kinase 5 OS=Homo sapiens OX=9606 GN=MAP3K5 PE=1 SV=1
725	A7E2V4|ZSW	43,1751	0,65	0,33	0	0	0,33	2				1,48E+07	1	1			2	Acetylatic	197297,33	Zinc finger SWIM domain-containing protein 8 OS=Homo sapiens OX=9606 GN=ZSWIM8 PE=1 SV=1
1082	Q3T906|GNP	25,46424	1,43	0	0	0	1,43	1					1				1	Carbamid	143621,86	N-acetylglucosamine-1-phosphotransferase subunits alpha/beta OS=Homo sapiens OX=9606 GN=GNPTAB PE=1 SV=1
1083	P62913|RL11_	28,76667	5,06	0	0	0	5,06	1					1				1		20252,383	60S ribosomal protein L11 OS=Homo sapiens OX=9606 GN=RPL11 PE=1 SV=2
1063	A6NGD5|ZSA	24,44333	1,21	0	0	1,21	0	1			245290,78		1			1			55729,047	Zinc finger and SCAN domain-containing protein 5C OS=Homo sapiens OX=9606 GN=ZSCAN5C PE=3 SV=1
56	O94911|ABC/	80,91282	0,37	0	0	0,37	0,37	1			1044845,06	7799888	1			2	4		183676,62	ABC-type organic anion transporter ABCA8 OS=Homo sapiens OX=9606 GN=ABCA8 PE=1 SV=4
176	Q12830|BPTF	40,70679	0,2	0,2	0	0,2	0,2	1	2279525,8		646554	731160,5	1	1		1	2		338262,44	Nucleosome-remodeling factor subunit BPTF OS=Homo sapiens OX=9606 GN=BPTF PE=1 SV=3
1032	Q4G0S4|C27(	47,494	1,11	1,11	1,11	0	0	1	304970,78	254798,3			1	1	2				60920,32	Cytochrome P450 27C1 OS=Homo sapiens OX=9606 GN=CYP27C1 PE=1 SV=3
639	Q8IWB9|TEX:	26,4958	0,53	0	0,53	0	0	1					1		1				125302,695	Testis-expressed protein 2 OS=Homo sapiens OX=9606 GN=TEX2 PE=1 SV=2
68	P09238|MMP	223,8009	5,88	4,41	5,88	0	4,41	4		2854690,2		786028,75	3	5	29		6		54151,242	Stromelysin-2 OS=Homo sapiens OX=9606 GN=MMP10 PE=1 SV=1
96	Q02388|CO7/	370,3708	5,81	3,91	4,31	1,83	1,29	26	6165164	458961,56	56913,457		6	29	33	13	12	Acetylatic	295219,6	Collagen alpha-1(VII) chain OS=Homo sapiens OX=9606 GN=COL7A1 PE=1 SV=2
36	O43681|GET3	68,97637	1,72	1,72	1,72	1,72	0	1	1507078,2	497451,88	100308,79		1	1	1	1			38792,773	ATPase GET3 OS=Homo sapiens OX=9606 GN=GET3 PE=1 SV=2
726	Q06828|FMO	93,83523	3,99	3,99	0	0	0	2					2	3					43178,793	Fibromodulin OS=Homo sapiens OX=9606 GN=FMOD PE=1 SV=2
213	P23634|AT2B	352,66	9,83	0	0	0	9,83	11				307189,28	8				15	Deamidat	137920,16	Plasma membrane calcium-transporting ATPase 4 OS=Homo sapiens OX=9606 GN=ATP2B4 PE=1 SV=2
178	P13667|PDIA	366,7356	20,78	20,78	4,19	0,93	0	15	1122656,8	125221,734			13	20	5	1			72932,49	Protein disulfide-isomerase A4 OS=Homo sapiens OX=9606 GN=PDIA4 PE=1 SV=2
83	A6NES4|MRC	78,4077	0,36	0,36	0,36	0	0	1	207145,14	1469270,9			1	1	3				189560,98	Maestro heat-like repeat-containing protein family member 2A OS=Homo sapiens OX=9606 GN=MROH2A PE=4 SV=4
1084	P20933|ASPC	39,18121	2,31	0	2,31	0	0	1		21063,516			1		1				37208,27	N(4)-(beta-N-acetylglucosaminyl)-L-asparaginase OS=Homo sapiens OX=9606 GN=AGA PE=1 SV=2
505	Q96MG2|JSP	31,26732	1,81	0	0	1,81	1,81	1			102164,09	89182,87	1			1	1		36318,676	Junctional sarcoplasmic reticulum protein 1 OS=Homo sapiens OX=9606 GN=JSRP1 PE=1 SV=1
652	Q9UPN4|CP1	29,76723	0,55	0	0	0,55	0	1					1			1			122148,74	Centrosomal protein of 131 kDa OS=Homo sapiens OX=9606 GN=CEP131 PE=1 SV=3
738	P46092|CCR1	27,59454	1,66	0	0	0	1,66	1					1				1		38415,74	C-C chemokine receptor type 10 OS=Homo sapiens OX=9606 GN=CCR10 PE=1 SV=3
411	Q3SY69|AL1L	29,13054	0,65	0	0	0	0,65	1				114344,34	1				1		101745,72	Mitochondrial 10-formyltetrahydrofolate dehydrogenase OS=Homo sapiens OX=9606 GN=ALDH1L2 PE=1 SV=2
459	O00468|AGRI	57,40987	0,92	0,29	0,34	0,29	0,29	3			3851886	187572,34	2	1	1	1	1	Acetylatic	217319,53	Agrin OS=Homo sapiens OX=9606 GN=AGRN PE=1 SV=6
404	Q86YS7|C2CI	63,28127	0,6	0	0	0,6	0,6	1			6225,622	8822,583	1			1	1		110447,01	C2 domain-containing protein 5 OS=Homo sapiens OX=9606 GN=C2CD5 PE=1 SV=1
554	O43657|TSN(	169,7765	11,43	0	0	0	11,43	3				1286899,2	3				3	Carbamid	27563,266	Tetraspanin-6 OS=Homo sapiens OX=9606 GN=TSPAN6 PE=1 SV=1
1085	P61978|HNRI	33,58034	1,73	1,73	0	0	0	1	53644,734				1	1					50976,242	Heterogeneous nuclear ribonucleoprotein K OS=Homo sapiens OX=9606 GN=HNRNPK PE=1 SV=1
555	Q9H3U5|MFS	139,918	5,81	0	0	0	5,81	3				103754,28	2				3	Acetylatic	51208,582	Major facilitator superfamily domain-containing protein 1 OS=Homo sapiens OX=9606 GN=MFSD1 PE=2 SV=2
746	Q9P212|PLCE	28,48551	0,26	0	0	0	0,26	1					1				1		258714,55	1-phosphatidylinositol 4,5-bisphosphate phosphodiesterase epsilon-1 OS=Homo sapiens OX=9606 GN=PLCE1 PE=1 SV=3
376	P0C0S5|H2AZ	155,9647	12,5	7,03	7,03	7,03	12,5	2	230969,52	229668,45	250080,11	1776298	2	3	4	2	7		13552,734	Histone H2A.Z OS=Homo sapiens OX=9606 GN=H2AZ1 PE=1 SV=2
117	P23276|KELL_	25,85045	0,82	0,82	0	0	0	1					1	1				Carbamid	82823,85	Kell blood group glycoprotein OS=Homo sapiens OX=9606 GN=KEL PE=1 SV=2
1086	Q07617|SPA(	23,09253	1,08	0	0	0	1,08	1					1				1		103638,5	Sperm-associated antigen 1 OS=Homo sapiens OX=9606 GN=SPAG1 PE=1 SV=3
1025	Q8NGB8|O4F	27,11249	1,92	0	0	0	1,92	1				76108,34	1				2		35369,42	Olfactory receptor 4F15 OS=Homo sapiens OX=9606 GN=OR4F15 PE=2 SV=1
1087	Q9BQ52|RNZ	26,57442	0,73	0	0	0	0,73	1				346691,56	1				1		92219	Zinc phosphodiesterase ELAC protein 2 OS=Homo sapiens OX=9606 GN=ELAC2 PE=1 SV=2
301	P32248|CCR7	68,35734	1,59	0	0	0	1,59	1					1				1		42873,996	C-C chemokine receptor type 7 OS=Homo sapiens OX=9606 GN=CCR7 PE=1 SV=2
729	P35670|ATP7	57,92875	0,82	0	0,41	0,41	0	2		141745,42			1		1	1			157262,66	Copper-transporting ATPase 2 OS=Homo sapiens OX=9606 GN=ATP7B PE=1 SV=4
85	Q9H9Q2|CSN	53,14703	2,27	2,27	2,27	0	0	1	357639,53	93954,47			1	1	1				29621,953	COP9 signalosome complex subunit 7b OS=Homo sapiens OX=9606 GN=COPS7B PE=1 SV=1
179	P10909|CLUS	371,6241	36,53	36,53	1,78	0	0	14	5657001				14	27	1			Carbamid	52494,574	Clusterin OS=Homo sapiens OX=9606 GN=CLU PE=1 SV=1
107	P13646|K1C1	375,9141	16,38	3,06	3,28	12,88	15,94	17					1	3	8	118	88	Deamidat	49588,207	Keratin, type I cytoskeletal 13 OS=Homo sapiens OX=9606 GN=KRT13 PE=1 SV=4
127	P07602|SAP_	409,4005	34,92	34,92	24,62	0	4,2	21	2,05E+07	3421840,8			20	55	41		2	Carbamid	58112,637	Prosaposin OS=Homo sapiens OX=9606 GN=PSAP PE=1 SV=2
556	Q9NVM1|EV/	214,4449	32,73	0	0	0	32,73	3				482813,3	3				4		18373,602	Protein eva-1 homolog B OS=Homo sapiens OX=9606 GN=EVA1B PE=1 SV=1
730	P36578|RL4_I	67,82168	2,81	0	1,41	0	2,81	2				1,78E+07	2		1		2		47697,324	60S ribosomal protein L4 OS=Homo sapiens OX=9606 GN=RPL4 PE=1 SV=5
1088	Q8WWI1|LM	56,95756	0,48	0,48	0	0	0	1					1	1					192695,64	LIM domain only protein 7 OS=Homo sapiens OX=9606 GN=LMO7 PE=1 SV=3
167	P29966|MAR	383,7219	53,01	51,81	43,98	50	22,89	14	6053692,5	505314,7	2295810,5	425773,7	14	35	10	19	6	Deamidat	31554,656	Myristoylated alanine-rich C-kinase substrate OS=Homo sapiens OX=9606 GN=MARCKS PE=1 SV=4
3	O14653|GOSI	93,56394	2,83	2,83	2,83	2,83	2,83	1	1416632,1	4961949	1,55E+08	1,59E+08	1	1	17	7	10		24775,396	Golgi SNAP receptor complex member 2 OS=Homo sapiens OX=9606 GN=GOSR2 PE=1 SV=2
343	P61224|RAP1	292,4075	25	0	0	25	21,2	7			123477,21	291905,78	3			6	6	Carbamid	20824,783	Ras-related protein Rap-1b OS=Homo sapiens OX=9606 GN=RAP1B PE=1 SV=1
1031	Q8NE35|CPEI	33,52007	0,86	0	0	0	0,86	1					1				1		76014,28	Cytoplasmic polyadenylation element-binding protein 3 OS=Homo sapiens OX=9606 GN=CPEB3 PE=1 SV=2

59	Q12800|TFCP	70,64884	1,2	0	1,2	0	0	1					1		2				57255,656	Alpha-globin transcription factor CP2 OS=Homo sapiens OX=9606 GN=TFCP2 PE=1 SV=2
669	Q92777|SYN	28,71587	1,03	0	0	0	1,03	1				92112,805	1				1		62996,38	Synapsin-2 OS=Homo sapiens OX=9606 GN=SYN2 PE=1 SV=4
1069	Q9BYV8|CEP	28,22043	1,61	0	0	0	1,61	1				2043663,5	1				1		41368,402	Centrosomal protein of 41 kDa OS=Homo sapiens OX=9606 GN=CEP41 PE=1 SV=1
495	A1L443|NTM	53,03056	0,79	0	0	0	0,79	1				29151,854	1				1	Deamidat	80769,89	NUT family member 2F OS=Homo sapiens OX=9606 GN=NUTM2F PE=2 SV=2
1089	Q9GZM5|YIPF	38,7034	2,86	2,86	2,86	0	0	1	86253,38	39304,77			1	1	2				38247,496	Protein YIPF3 OS=Homo sapiens OX=9606 GN=YIPF3 PE=1 SV=1
1090	B5MCY1|TDR	33,40335	0,31	0	0	0,31	0	1					1			1			221721,95	Tudor domain-containing protein 15 OS=Homo sapiens OX=9606 GN=TDRD15 PE=2 SV=1
186	O00213|APBI	97,96496	0,85	0,85	0,85	0,85	0,85	1	404544,56	132516,72	213150,33	123433,66	1	1	2	1	1		77244,195	Amyloid beta precursor protein binding family B member 1 OS=Homo sapiens OX=9606 GN=APBB1 PE=1 SV=2
86	P18564|ITB6	118,4794	1,14	0	0	1,14	1,14	1			23385,28	269106,3	1			1	2		85935,984	Integrin beta-6 OS=Homo sapiens OX=9606 GN=ITGB6 PE=1 SV=2
764	Q9UHP3|UBP	37,26005	0,57	0	0	0,57	0	1			49049,004		1			1			122217,9	Ubiquitin carboxyl-terminal hydrolase 25 OS=Homo sapiens OX=9606 GN=USP25 PE=1 SV=4
970	Q9Y6I0|VPK6	27,98863	3,85	3,85	0	0	0	1	24682,994				1	2					16972,43	Endogenous retrovirus group K member 6 Pro protein OS=Homo sapiens OX=9606 GN=ERVK-6 PE=3 SV=2
731	P09341|GRO	65,14258	13,08	13,08	0	0	0	2	343283,28				2	2					11301,412	Growth-regulated alpha protein OS=Homo sapiens OX=9606 GN=CXCL1 PE=1 SV=1
298	A0A2R8Y619|	147,6055	19,67	13,93	0	13,93	19,67	3				36598,527	1	3		4	6		13491,466	Histone H2B type 2-K1 OS=Homo sapiens OX=9606 GN=H2BK1 PE=3 SV=1
1091	Q7Z4I7|LIMS	33,75166	1,76	0	0	0	1,76	1				1383267,9	1				1		38916,04	LIM and senescent cell antigen-like-containing domain protein 2 OS=Homo sapiens OX=9606 GN=LIMS2 PE=1 SV=1
732	Q9UJJ9|GNP	80,51206	1,97	1,97	1,97	1,97	1,97	1	110671,62		217832,83	95613,336	1	1	1	1	1	Deamidat	33973,55	N-acetylglucosamine-1-phosphotransferase subunit gamma OS=Homo sapiens OX=9606 GN=GNPTG PE=1 SV=1
174	P42658|DPP6	33,9707	0,69	0	0	0,69	0	1			235804,4		1			1			97588,19	Dipeptidyl aminopeptidase-like protein 6 OS=Homo sapiens OX=9606 GN=DPP6 PE=1 SV=2
180	P19021|AMD	343,9403	12,54	7,81	9,35	0	0,72	15	1528575,2	380884,25		46140,496	14	12	18		1	Deamidat	108332,055	Peptidyl-glycine alpha-amidating monooxygenase OS=Homo sapiens OX=9606 GN=PAM PE=1 SV=2
232	O76061|STC2	311,701	24,5	24,5	15,56	0	0	9	6641453,5	2868487,5			9	26	13			Oxidation	33248,562	Stanniocalcin-2 OS=Homo sapiens OX=9606 GN=STC2 PE=1 SV=1
206	P0DN26|PAL	269,5659	28,05	11,59	11,59	28,05	28,05	6	212291,48	104125,625	604586,06	671093,94	4	3	3	9	8	Carbamid	18196,809	Peptidyl-prolyl cis-trans isomerase A-like 4F OS=Homo sapiens OX=9606 GN=PPIAL4F PE=3 SV=1
27	Q8IYJ1|CPNE	57,95885	1,08	0	0	1,08	0	1			19414,406		1			1			61864,105	Copine-9 OS=Homo sapiens OX=9606 GN=CPNE9 PE=1 SV=3
134	Q16610|ECM	395,6614	32,96	32,96	20,19	2,04	1,11	17	1,45E+07	1843534,2	110895,05	52273,69	17	65	23	3	1	Carbamid	60674,086	Extracellular matrix protein 1 OS=Homo sapiens OX=9606 GN=ECM1 PE=1 SV=2
29	Q13751|LAM	82,6759	0,51	0,51	0,51	0	0,51	1	200872,44	148834,94		231725,69	1	1	1		1		129572,48	Laminin subunit beta-3 OS=Homo sapiens OX=9606 GN=LAMB3 PE=1 SV=1
51	Q6P2I3|FAH2	97,32061	1,91	0	0	0	1,91	1				69994,57	1				2		34613,184	Fumarylacetoacetate hydrolase domain-containing protein 2B OS=Homo sapiens OX=9606 GN=FAHD2B PE=1 SV=1
626	P63027|VAM	167,619	20,69	0	0	0	20,69	2				49215,074	2				2		12662,726	Vesicle-associated membrane protein 2 OS=Homo sapiens OX=9606 GN=VAMP2 PE=1 SV=3
1035	Q9HDB9|GAK	23,76196	0,9	0	0	0,9	0	1			231974,8		1			1			73588,12	Endogenous retrovirus group K member 5 Gag polyprotein OS=Homo sapiens OX=9606 GN=ERVK-5 PE=1 SV=3
272	Q13162|PRD	173,042	5,54	2,95	0	5,54	5,54	2			736903,94	98883,18	2	3		3	3		30539,91	Peroxiredoxin-4 OS=Homo sapiens OX=9606 GN=PRDX4 PE=1 SV=1
1092	Q9H0E2|TOL	54,37845	2,92	0	0	0	2,92	1				70556,16	1				1		30281,805	Toll-interacting protein OS=Homo sapiens OX=9606 GN=TOLLIP PE=1 SV=1
27	Q8NB25|F18	88,01299	0,53	0,53	0,53	0,53	0,53	1	431669,16		123204,05	385846,25	1	2	3	1	2		132964,98	Protein FAM184A OS=Homo sapiens OX=9606 GN=FAM184A PE=1 SV=3
1093	Q9UJX2|CDC	55,59209	1,01	0	1,01	0	1,01	1					1		1		1		68834,164	Cell division cycle protein 23 homolog OS=Homo sapiens OX=9606 GN=CDC23 PE=1 SV=3
137	P54707|AT12	180,9017	2,31	0	0	1,44	2,31	3			100465,99	389080,62	2			2	3	Carbamid	115510,52	Potassium-transporting ATPase alpha chain 2 OS=Homo sapiens OX=9606 GN=ATP12A PE=1 SV=3
1094	Q99961|SH3	24,5869	2,17	0	0	0	2,17	1				32422,893	1				1		41489,95	Endophilin-A2 OS=Homo sapiens OX=9606 GN=SH3GL1 PE=1 SV=1
82	Q5XKE5|K2C	420,176	19,44	8,22	11,21	19,44	16,26	23	193270,89	267425,75	1,66E+07	256856,12	1	11	18	127	69	Oxidation	57835,598	Keratin, type II cytoskeletal 79 OS=Homo sapiens OX=9606 GN=KRT79 PE=1 SV=2
230	P55771|PAX	39,20821	1,76	0	0	1,76	0	1			46140,46		1			1			36310,117	Paired box protein Pax-9 OS=Homo sapiens OX=9606 GN=PAX9 PE=1 SV=3
202	Q9H0I9|TKTL	72,29531	1,28	1,28	0	0	0	1	101478,82				1	3					67877,24	Transketolase-like protein 2 OS=Homo sapiens OX=9606 GN=TKTL2 PE=2 SV=1
1095	P46020|KPB1	24,87802	0,49	0,49	0	0	0	1	328019,5				1	1					137311,97	Phosphorylase b kinase regulatory subunit alpha, skeletal muscle isoform OS=Homo sapiens OX=9606 GN=PHKA1 PE=1 SV=2
1096	Q96RQ3|MCC	30,07748	0,83	0	0,83	0	0	1		312877,5			1		2				80473,03	Methylcrotonoyl-CoA carboxylase subunit alpha, mitochondrial OS=Homo sapiens OX=9606 GN=MCCC1 PE=1 SV=3
1090	P78536|ADA	33,40335	0,73	0	0	0,73	0	1					1			1			93020,95	Disintegrin and metalloproteinase domain-containing protein 17 OS=Homo sapiens OX=9606 GN=ADAM17 PE=1 SV=1
214	Q13308|PTK7	314,7325	10,09	7,57	5,51	3,08	0	12	599597,94	190604,78	598722,75		10	10	7	4		Carbamid	118391,63	Inactive tyrosine-protein kinase 7 OS=Homo sapiens OX=9606 GN=PTK7 PE=1 SV=2
933	A0A1B0GV85	24,05968	1,14	0	0	1,14	0	1			27898,795		1			1			56836,715	Reelin domain-containing protein 1 OS=Homo sapiens OX=9606 GN=REELD1 PE=2 SV=2
735	P84098|RL19	67,21954	7,65	0	0	3,06	7,65	2				57508,484	1			1	2		23465,965	60S ribosomal protein L19 OS=Homo sapiens OX=9606 GN=RPL19 PE=1 SV=1
460	P62873|GBB1	218,311	10,59	0	0	10,59	7,65	4			225321,12	72456,77	3			5	2	Carbamid	37376,96	Guanine nucleotide-binding protein G(I)/G(S)/G(T) subunit beta-1 OS=Homo sapiens OX=9606 GN=GNB1 PE=1 SV=3
1097	Q92485|ASM	27,22453	1,32	0	0	0	1,32	1				378022,38	1				1		50813,844	Acid sphingomyelinase-like phosphodiesterase 3b OS=Homo sapiens OX=9606 GN=SMPDL3B PE=1 SV=2
1098	Q9NVX7|KBT	24,85937	1,16	0	0	1,16	0	1					1			1		Acetylatic	58143,973	Kelch repeat and BTB domain-containing protein 4 OS=Homo sapiens OX=9606 GN=KBTBD4 PE=1 SV=3
181	P14209|CD99	363,7888	50,27	42,16	33,51	5,41	29,73	15	1260361,9	1225350,5	223216,5	605625,25	15	16	15	1	6		18847,873	CD99 antigen OS=Homo sapiens OX=9606 GN=CD99 PE=1 SV=1
108	Q96P70|IPO	60,04453	0,58	0,58	0	0	0	1	544477,3				1	1					115962,85	Importin-9 OS=Homo sapiens OX=9606 GN=IPO9 PE=1 SV=3
559	Q7RTP6|MIC	121,9969	0,9	0,3	0,6	0,3	0,6	3					1	2	2	1	2		224294,83	[F-actin]-monooxygenase MICAL3 OS=Homo sapiens OX=9606 GN=MICAL3 PE=1 SV=2
50	P42858|HD_	110,4384	0,19	0,19	0,19	0	0	1	159328,34	50313,375			1	1	1				347603,28	Huntingtin OS=Homo sapiens OX=9606 GN=HTT PE=1 SV=2
198	Q6PEY2|TBA	284,3973	21,33	1,33	0	21,33	8,44	6			310849,75		1	1		9	2	Deamidat	49858,54	Tubulin alpha-3E chain OS=Homo sapiens OX=9606 GN=TUBA3E PE=1 SV=2
560	Q5VW32|BR	162,2584	4,38	0	1,46	0	4,38	3				17383,4	2		1		4		46476,086	BRO1 domain-containing protein BROX OS=Homo sapiens OX=9606 GN=BROX PE=1 SV=1
1099	Q9UGI0|ZRA	28,66452	0,85	0	0	0	0,85	1				25210,613	1				1		80967,164	Ubiquitin thioesterase ZRANB1 OS=Homo sapiens OX=9606 GN=ZRANB1 PE=1 SV=2
617	Q9UJT2|TSKS	83,19873	1,01	0	0	1,01	0	1					1			1			65050,16	Testis-specific serine kinase substrate OS=Homo sapiens OX=9606 GN=TSKS PE=1 SV=3
108	P51884|LUM	401,2968	45,56	45,56	26,33	0	0	19	1,26E+08	1,35E+07			19	129	55			Carbamid	38429,01	Lumican OS=Homo sapiens OX=9606 GN=LUM PE=1 SV=2
385	P20916|MAG	53,32415	0,96	0	0	0,96	0	1			73782,05		1			1			69068,516	Myelin-associated glycoprotein OS=Homo sapiens OX=9606 GN=MAG PE=1 SV=1
738	Q8WWB7|GL	66,80316	7,88	0	0	0	7,88	2					2				2	Carbamid	43864,168	Glycosylated lysosomal membrane protein OS=Homo sapiens OX=9606 GN=GLMP PE=1 SV=1
33	P11717|MPR	118,4235	0,32	0,32	0,32	0,32	0,32	1	3513453,5	362057,8	210599,3	590180,5	1	5	3	2	3	Deamidat	274374,8	Cation-independent mannose-6-phosphate receptor OS=Homo sapiens OX=9606 GN=IGF2R PE=1 SV=3
257	P39060|COIA	277,1235	3,42	2,39	2,34	1,2	0,46	10		46058,367			1	11	9	4	2		178187,66	Collagen alpha-1(XVIII) chain OS=Homo sapiens OX=9606 GN=COL18A1 PE=1 SV=5
1100	Q96CN9|GCC	28,92016	0,77	0	0	0	0,77	1				1687869,4	1				1	Acetylatic	87810,69	GRIP and coiled-coil domain-containing protein 1 OS=Homo sapiens OX=9606 GN=GCC1 PE=1 SV=1
1101	P04062|GBA	128,84751	2,43	2,43	0	0	0	1					1	1					59716,277	Lysosomal acid glucosylceramidase OS=Homo sapiens OX=9606 GN=GBA1 PE=1 SV=3
355	P58335|ANT	64,35437	1,23	0	0	0	1,23	1				233466,8	1				1		53666,168	Anthrax toxin receptor 2 OS=Homo sapiens OX=9606 GN=ANTXR2 PE=1 SV=5
36	O60673|REV	115,9746	0,22	0,22	0,22	0,22	0,22	2	3167589,2	860580,6	105287,28	1092379,6	1	8	4	2	3	Deamidat	352775,72	DNA polymerase zeta catalytic subunit OS=Homo sapiens OX=9606 GN=REV3L PE=1 SV=2
23	Q96GE5|ZN7	116,1863	0,93	0,93	0,93	0,93	0,93	1	137836,5	136673,25	5583690,5	5534616	1	1	1	2	1		74288,43	Zinc finger protein 799 OS=Homo sapiens OX=9606 GN=ZNF799 PE=2 SV=4
105	Q9UIA9|XPO	66,29748	0,55	0,55	0,55	0	0,55	1	132969,38	238432,69		43754,15	1	1	3		1		123907,375	Exportin-7 OS=Homo sapiens OX=9606 GN=XPO7 PE=1 SV=3
1102	O95084|PRS	33,50097	2,87	2,87	0	0	0	1	486980,66				1	1					43001,246	Serine protease 23 OS=Homo sapiens OX=9606 GN=PRSS23 PE=1 SV=1
1103	P60520|GBR	27,40327	6,84	0	0	0	6,84	1				77713,1	1				1		13666,835	Gamma-aminobutyric acid receptor-associated protein-like 2 OS=Homo sapiens OX=9606 GN=GABARAPL2 PE=1 SV=1
31	P08670|VIM	514,5629	67,81	65,67	50,21	64,81	57,94	45	1,18E+08	7605921,5	1,21E+07	1,80E+07	37	163	75	66	56	Deamidat	53651,676	Vimentin OS=Homo sapiens OX=9606 GN=VIM PE=1 SV=4
1104	P12034|FGF5	58,72685	2,24	2,24	2,24	0	0	1					1	2	33				29550,756	Fibroblast growth factor 5 OS=Homo sapiens OX=9606 GN=FGF5 PE=1 SV=4
1105	P00390|GSH	33,0232	1,53	1,53	0	0	0	1	14119,57				1	1					56257,008	Glutathione reductase, mitochondrial OS=Homo sapiens OX=9606 GN=GSR PE=1 SV=2
419	P20648|ATP4	201,1736	3,96	0	0	1,35	3,38	5				23755,602	1			2	4	Acetylatic	114118,87	Potassium-transporting ATPase alpha chain 1 OS=Homo sapiens OX=9606 GN=ATP4A PE=1 SV=5
262	Q58EX2|SDK	106,8753	0,28	0,28	0,28	0	0	1					1	2	3				239396,08	Protein sidekick-2 OS=Homo sapiens OX=9606 GN=SDK2 PE=1 SV=3
739	Q6P1R3|MSD	84,83708	1,25	1,07	1,07	1,07	1,25	2				938182,56	1	3	3	8	4		61319,348	Myb/SANT-like DNA-binding domain-containing protein 2 OS=Homo sapiens OX=9606 GN=MSANTD2 PE=1 SV=1
1106	P42785|PCP_	23,91519	2,42	2,42	0	0	0	1	31321,008				1	1					55799,79	Lysosomal Pro-X carboxypeptidase OS=Homo sapiens OX=9606 GN=PRCP PE=1 SV=1
461	P45877|PPIC	230,8996	15,57	15,57	9,43	2,83	2,83	4	45680,184				2	20	21	1	3		22763,258	Peptidyl-prolyl cis-trans isomerase C OS=Homo sapiens OX=9606 GN=PPIC PE=1 SV=1
741	Q9NUN5|LM	76,34281	4,81	0	0	0	4,81	2				83835,766	2				2		61388,74	Lysosomal cobalamin transport escort protein LMBD1 OS=Homo sapiens OX=9606 GN=LMBRD1 PE=1 SV=1
1011	Q86T75|NBP	41,75044	0,69	0	0	0,69	0,69	1			5235523	7,28E+07	1			2	4		99432,65	Neuroblastoma breakpoint family member 11 OS=Homo sapiens OX=9606 GN=NBPF11 PE=2 SV=3
169	Q8NFZ5|TNIF	56,70879	1,4	0	1,4	0	0	1					1		3				48699,56	TNFAIP3-interacting protein 2 OS=Homo sapiens OX=9606 GN=TNIP2 PE=1 SV=1
398	Q9H2S9|IKZF	32,09123	1,03	1,03	1,03	0	0	1	887625,8	83099,29			1	1	1				64105,977	Zinc finger protein Eos OS=Homo sapiens OX=9606 GN=IKZF4 PE=1 SV=2
159	O95861|BPN	48,53948	1,95	0	0	1,95	1,95	1			22217,67	92420,484	1			1	1		33392,324	3'(2'),5'-bisphosphate nucleotidase 1 OS=Homo sapiens OX=9606 GN=BPNT1 PE=1 SV=1
593	P22532|SPR2	180,0902	30,56	0	0	30,56	30,56	2			894421,7	1087446,5	2			5	5	Carbamid	7905,352	Small proline-rich protein 2D OS=Homo sapiens OX=9606 GN=SPRR2D PE=2 SV=2
1011	Q5TAG4|NBF	41,75044	0,41	0	0	0,41	0,41	1			5235523	7,28E+07	1			2	4		167688,6	Neuroblastoma breakpoint family member 12 OS=Homo sapiens OX=9606 GN=NBPF12 PE=2 SV=3
505	P11277|SPTB	31,26732	0,28	0	0	0,28	0,28	1			102164,09	89182,87	1			1	1		246468,11	Spectrin beta chain, erythrocytic OS=Homo sapiens OX=9606 GN=SPTB PE=1 SV=5
27	Q86VQ0|LCA	88,28559	0,86	0,86	0,86	0	0,86	1	493308,2			765013,2	1	1	1		1		80554,23	Lebercilin OS=Homo sapiens OX=9606 GN=LCA5 PE=1 SV=2
420	P07864|LDHC	185,6782	9,94	5,42	7,83	5,42	5,42	5				52371,27	2	5	4	3	2	Acetylatic	36311,29	L-lactate dehydrogenase C chain OS=Homo sapiens OX=9606 GN=LDHC PE=1 SV=4
329	Q8NB78|KDN	58,51812	0,73	0,73	0	0,73	0,73	1	133473,9		419747,28	265960,53	1	1		1	1		92098,305	Lysine-specific histone demethylase 2 OS=Homo sapiens OX=9606 GN=KDM1B PE=1 SV=3
75	P62736|ACTA	417,4441	35,54	32,36	9,55	35,54	29,71	21			1,07E+07	672794,6	1	34	7	119	42	Oxidation	42008,945	Actin, aortic smooth muscle OS=Homo sapiens OX=9606 GN=ACTA2 PE=1 SV=1
1107	Q9H8M2|BRD	22,80036	1,01	0	0	0	1,01	1				32934,953	1				1	Acetylatic	67000,1	Bromodomain-containing protein 9 OS=Homo sapiens OX=9606 GN=BRD9 PE=1 SV=2
61	Q7Z3B3|KAN	102,7979	0,54	0,54	0,54	0	0	1	153530,9	82106,97			1	1	2				121024,734	KAT8 regulatory NSL complex subunit 1 OS=Homo sapiens OX=9606 GN=KANSL1 PE=1 SV=2
462	P58107|EPIP	213,1215	0,63	0	0	0,63	0,28	4					1			4	2		555658,1	Epiplakin OS=Homo sapiens OX=9606 GN=EPPK1 PE=1 SV=3
1058	Q99504|EYA3	30,21216	1,05	0	0	0	1,05	1					1				1		62662,816	Eyes absent homolog 3 OS=Homo sapiens OX=9606 GN=EYA3 PE=1 SV=3
84	Q9UMY4|SN	28,22482	3,7	3,7	0	0	0	1	472656,12				1	1					18884,516	Sorting nexin-12 OS=Homo sapiens OX=9606 GN=SNX12 PE=1 SV=4
350	P30260|CDC2	27,74284	0,73	0	0	0,73	0	1			45709,625		1			1			91867,33	Cell division cycle protein 27 homolog OS=Homo sapiens OX=9606 GN=CDC27 PE=1 SV=2
1108	Q01650|LAT1	31,15429	1,97	0	0	1,97	0	1			57927,508		1			1			55010,24	Large neutral amino acids transporter small subunit 1 OS=Homo sapiens OX=9606 GN=SLC7A5 PE=1 SV=2
154	P80723|BASP	398,3837	78,85	72,25	78,85	53,74	66,08	17	2539863	2505741,5	1458749	2180050,2	17	17	27	15	17		22693,408	Brain acid soluble protein 1 OS=Homo sapiens OX=9606 GN=BASP1 PE=1 SV=2
672	O95347|SMC	66,51751	0,5	0	0	0,5	0	1			480016,22		1			1			135656,08	Structural maintenance of chromosomes protein 2 OS=Homo sapiens OX=9606 GN=SMC2 PE=1 SV=2
745	P0DME0|SETI	57,36063	5,3	3,31	0	1,99	0	2					1	1		1			34882,406	Protein SETSIP OS=Homo sapiens OX=9606 GN=SETSIP PE=1 SV=1
746	Q02880|TOP	41,37442	0,74	0	0	0	0,74	2					1				2		183267,17	DNA topoisomerase 2-beta OS=Homo sapiens OX=9606 GN=TOP2B PE=1 SV=3
258	P62258|1433	323,0962	36,08	33,73	19,22	20,78	23,14	9	943713,9	70640,74	213390,6	232274,05	6	16	7	9	7	Oxidation	29173,89	14-3-3 protein epsilon OS=Homo sapiens OX=9606 GN=YWHAE PE=1 SV=1
344	P27658|CO8	217,8359	6,05	3,63	1,75	0,81	0,81	7	151758,45	41584,84			2	6	2	1	1		73364,09	Collagen alpha-1(VIII) chain OS=Homo sapiens OX=9606 GN=COL8A1 PE=1 SV=2
747	P13489|RIN	88,45586	3,47	0	0	3,47	0	2			84565,74		2			2		Carbamid	49973,465	Ribonuclease inhibitor OS=Homo sapiens OX=9606 GN=RNH1 PE=1 SV=2
561	Q6W2J9|BCC	131,8517	1,08	0,4	0,74	0,34	0	3					1	5	5	1			192188,64	BCL-6 corepressor OS=Homo sapiens OX=9606 GN=BCOR PE=1 SV=1
562	A4UGR9|XIRF	75,15895	0,53	0,18	0	0,18	0,36	3					1	1		1	2		382299,62	Xin actin-binding repeat-containing protein 2 OS=Homo sapiens OX=9606 GN=XIRP2 PE=1 SV=2
750	P13797|PLST	140,2538	2,38	0,95	0	1,43	0	2			15519,787		1	1		2		Carbamid	70811,016	Plastin-3 OS=Homo sapiens OX=9606 GN=PLS3 PE=1 SV=4
1109	P61247|RS3A	47,65567	3,03	0	0	0	3,03	1					1				1		29944,906	40S ribosomal protein S3a OS=Homo sapiens OX=9606 GN=RPS3A PE=1 SV=2
1110	Q9BQJ4|TMM	41,99913	6,08	0	0	0	6,08	1				326473,44	1				1		19997,64	Transmembrane protein 47 OS=Homo sapiens OX=9606 GN=TMEM47 PE=1 SV=1
114	P41594|GRM	29,58683	0,5	0,5	0	0	0	1	19643,275				1	1					132468,98	Metabotropic glutamate receptor 5 OS=Homo sapiens OX=9606 GN=GRM5 PE=1 SV=2

563	O15523|DDX	132,771	3,33	1,36	1,36	1,36	3,33	3	44414,03	153360,47	33899,344	59671,547	3	1	3	1	2		73153,62	ATP-dependent RNA helicase DDX3Y OS=Homo sapiens OX=9606 GN=DDX3Y PE=1 SV=2
464	Q14980|NUM	161,1549	1,18	0,85	0,57	0,28	0,33	4					1	3	2	1	1		238259,5	Nuclear mitotic apparatus protein 1 OS=Homo sapiens OX=9606 GN=NUMA1 PE=1 SV=2
8	Q6AHZ1|Z51	45,22557	0,4	0	0	0,4	0,4	1					1			1	1		166781,64	Zinc finger protein 518A OS=Homo sapiens OX=9606 GN=ZNF518A PE=1 SV=2
751	Q9UPN9|TRI	45,88464	1,06	0,53	0	0,53	0	2	28356,68				1	1		1		Deamidat	122533,164	E3 ubiquitin-protein ligase TRIM33 OS=Homo sapiens OX=9606 GN=TRIM33 PE=1 SV=3
1090	Q00613|HSF	33,40335	1,13	0	0	1,13	0	1					1			1			57260,492	Heat shock factor protein 1 OS=Homo sapiens OX=9606 GN=HSF1 PE=1 SV=1
646	Q8TB92|HMC	23,25292	1,62	0	0	0	1,62	1				65216,375	1				1		39514,32	3-hydroxy-3-methylglutaryl-CoA lyase, cytoplasmic OS=Homo sapiens OX=9606 GN=HMGCLL1 PE=1 SV=3
178	A4D2P6|GRD	98,92593	0,5	0,5	0	0	0	1	143868,6				1	1					132276,42	Delphilin OS=Homo sapiens OX=9606 GN=GRID2IP PE=3 SV=2
1044	Q9BUL9|RPP	40,36036	3,02	0	0	3,02	0	1			63496,605		1			1			20632,393	Ribonuclease P protein subunit p25 OS=Homo sapiens OX=9606 GN=RPP25 PE=1 SV=1
155	P16035|TIMP	361,027	42,73	42,73	33,64	0	25,91	13	2,37E+07	8387447,5		473960,2	13	48	41		9	Carbamid	24399,254	Metalloproteinase inhibitor 2 OS=Homo sapiens OX=9606 GN=TIMP2 PE=1 SV=2
960	Q6UVY6|MO	27,65908	0,98	0	0	0,98	0	1			223699,64		1			1			69652,49	DBH-like monooxygenase protein 1 OS=Homo sapiens OX=9606 GN=MOXD1 PE=1 SV=1
753	P19971|TYPH	61,87759	4,36	0	0	4,36	0	2			149136,61		2			2		Carbamid	49955,477	Thymidine phosphorylase OS=Homo sapiens OX=9606 GN=TYMP PE=1 SV=2
142	Q96JQ0|PCD	88,34358	0,18	0	0,18	0,18	0,18	1		55211,223	28992,957	216936,48	1		1	1	2		346181	Protocadherin-16 OS=Homo sapiens OX=9606 GN=DCHS1 PE=1 SV=1
285	O15522|NKX	42,64934	2,93	0	0	0	2,93	1				70901,19	1				1	Deamidat	25866,207	Homeobox protein Nkx-2.8 OS=Homo sapiens OX=9606 GN=NKX2-8 PE=2 SV=2
252	Q6DRA6|H2B	161,9766	11,59	11,59	11,59	7,93	11,59	3	117890,07	89813,125	87343,49	987559,8	1	3	4	2	5		18018,08	Putative histone H2B type 2-D OS=Homo sapiens OX=9606 GN=H2BC19P PE=5 SV=3
252	Q16778|H2B	299,3754	59,52	34,13	20,63	29,37	59,52	9	117890,07	89813,125	87343,49	987559,8	1	7	6	7	15	Oxidation	13920,161	Histone H2B type 2-E OS=Homo sapiens OX=9606 GN=H2BC21 PE=1 SV=3
1050	A0M8Q6|IGL	87,25446	5,66	5,66	0	0	0	1					1	1					11253,593	Immunoglobulin lambda constant 7 OS=Homo sapiens OX=9606 GN=IGLC7 PE=1 SV=3
39	Q13416|ORC	93,51338	1,04	0	0	1,04	1,04	1			89503,984	675079,94	1			1	3		65971,53	Origin recognition complex subunit 2 OS=Homo sapiens OX=9606 GN=ORC2 PE=1 SV=2
659	Q5VXH5|PRA	42,47769	1,27	0	1,27	0	0	1		806622,25			1		3				53626,754	PRAME family member 7 OS=Homo sapiens OX=9606 GN=PRAMEF7 PE=2 SV=2
815	Q15700|DLG	36,73071	0,69	0	0	0,69	0	1					1			1			97552,27	Disks large homolog 2 OS=Homo sapiens OX=9606 GN=DLG2 PE=1 SV=3
880	Q9BVC6|TM1	45,71551	2,47	2,47	0	0	0	1	1249522,2				1	2					26209,984	Transmembrane protein 109 OS=Homo sapiens OX=9606 GN=TMEM109 PE=1 SV=1
1111	Q7Z4F1|LRP1	67,40704	1,68	0	0	0	1,68	1				35072,812	1				1		76193,37	Low-density lipoprotein receptor-related protein 10 OS=Homo sapiens OX=9606 GN=LRP10 PE=1 SV=2
413	P84243|H33_	118,3275	23,53	4,41	4,41	5,15	23,53	5	340159,38	464861,53	729542	5116208	4	1	1	1	7	Oxidation	15327,907	Histone H3.3 OS=Homo sapiens OX=9606 GN=H3-3A PE=1 SV=2
754	P49327|FAS_	61,65681	0,52	0	0,24	0,28	0	2					1		1	2		Carbamid	273426,6	Fatty acid synthase OS=Homo sapiens OX=9606 GN=FASN PE=1 SV=3
1112	Q7Z5J4|RAI1	34,88274	0,31	0	0	0	0,31	1					1				1		203352,12	Retinoic acid-induced protein 1 OS=Homo sapiens OX=9606 GN=RAI1 PE=1 SV=2
196	Q01469|FABI	344,6099	69,63	0	11,11	69,63	69,63	11		316862,1	5897312	5694286,5	10		2	25	24	Acetylatic	15164,425	Fatty acid-binding protein 5 OS=Homo sapiens OX=9606 GN=FABP5 PE=1 SV=3
312	P04899|GNAI	306,7835	22,82	0	0	18,03	22,82	8			258300,8	357133,7	4			9	11	Carbamid	40450,902	Guanine nucleotide-binding protein G(i) subunit alpha-2 OS=Homo sapiens OX=9606 GN=GNAI2 PE=1 SV=3
421	Q9UBG0|MR	175,1757	2,77	1,15	1,15	0	1,22	5	31704,02	19997,67		34769,234	5	2	2		3	Carbamid	166674,45	C-type mannose receptor 2 OS=Homo sapiens OX=9606 GN=MRC2 PE=1 SV=2
58	P11142|HSP7	476,9331	49,85	10,37	1,08	38,85	48,76	33	90013,31	24345,746	1923556,1	8327519	18	10	1	47	68	Deamidat	70898,086	Heat shock cognate 71 kDa protein OS=Homo sapiens OX=9606 GN=HSPA8 PE=1 SV=1
57	Q03252|LMN	81,07773	1,29	1,29	0	0	0	1	313491,56				1	1					69948,39	Lamin-B2 OS=Homo sapiens OX=9606 GN=LMNB2 PE=1 SV=4
617	P52735|VAV	83,19873	0,68	0	0	0,68	0	1					1			1			101288,99	Guanine nucleotide exchange factor VAV2 OS=Homo sapiens OX=9606 GN=VAV2 PE=1 SV=2
174	P22670|RFX1	24,2844	0,61	0	0	0,61	0	1			102462,85		1			1			104757,78	MHC class II regulatory factor RFX1 OS=Homo sapiens OX=9606 GN=RFX1 PE=1 SV=2
204	O95466|FMN	42,55261	0,55	0	0	0	0,55	1				226608,28	1				1		121853,56	Formin-like protein 1 OS=Homo sapiens OX=9606 GN=FMNL1 PE=1 SV=3
206	A0A075B759|	269,5659	28,05	11,59	11,59	28,05	28,05	6	212291,48	104125,625	604586,06	671093,94	4	3	3	9	8	Carbamid	18196,809	Peptidyl-prolyl cis-trans isomerase A-like 4E OS=Homo sapiens OX=9606 GN=PPIAL4E PE=3 SV=1
465	P05231|IL6_	218,0505	17,45	17,45	0	0	0	4	385021,56				4	9					23718,217	Interleukin-6 OS=Homo sapiens OX=9606 GN=IL6 PE=1 SV=1
542	O14530|TXN	24,90426	2,65	0	0	2,65	0	1			29270,012		1			1			26534,035	Thioredoxin domain-containing protein 9 OS=Homo sapiens OX=9606 GN=TXNDC9 PE=1 SV=2
7	P15248|IL9_	102,55	4,17	4,17	4,17	4,17	4,17	1	408923,62	171858,38		1874509,9	1	4	1	4	3		15908,798	Interleukin-9 OS=Homo sapiens OX=9606 GN=IL9 PE=2 SV=1
1003	P10828|THB_	57,74165	1,52	0	0	1,52	1,52	1			1136429,6	1072534	1			4	4		52787,594	Thyroid hormone receptor beta OS=Homo sapiens OX=9606 GN=THRB PE=1 SV=2
608	Q6WRI0|IGS	123,88448	0,23	0,23	0	0	0	1	115692,805				1	1					290837,94	Immunoglobulin superfamily member 10 OS=Homo sapiens OX=9606 GN=IGSF10 PE=1 SV=1
168	P08729|K2C7	335,1706	17,7	7,25	8,32	15,14	14,5	14			244421,31	1038475,06	1	11	12	40	25	Oxidation	51385,656	Keratin, type II cytoskeletal 7 OS=Homo sapiens OX=9606 GN=KRT7 PE=1 SV=5
182	P68371|TBB4	303,7053	19,78	0	0	19,78	8,09	9			1900095,6	189163	9			12	5	Oxidation	49831	Tubulin beta-4B chain OS=Homo sapiens OX=9606 GN=TUBB4B PE=1 SV=1
156	P18206|VINC	321,8366	12,43	11,2	2,47	2,56	0	17	704554,25	58300,355	257427,72		17	16	4	4		Deamidat	123799,3	Vinculin OS=Homo sapiens OX=9606 GN=VCL PE=1 SV=4
757	P53634|CATC	121,6073	3,89	0	3,89	0	0	2		215391,17			2		3				51853,816	Dipeptidyl peptidase 1 OS=Homo sapiens OX=9606 GN=CTSC PE=1 SV=2
299	Q9NQT8|KI1	110,7621	0,38	0	0	0,38	0,38	1				19401,559	1			1	1		202788,64	Kinesin-like protein KIF13B OS=Homo sapiens OX=9606 GN=KIF13B PE=1 SV=2
1113	Q92542|NICA	37,51555	0,99	0	0	0	0,99	1				24335,93	1				1		78410,92	Nicastrin OS=Homo sapiens OX=9606 GN=NCSTN PE=1 SV=2
1114	Q9UPA5|BSN	36,30267	0,25	0	0,25	0	0	1		620843			1		1				416469,3	Protein bassoon OS=Homo sapiens OX=9606 GN=BSN PE=1 SV=4
346	Q6N069|NAA	29,32948	0,69	0	0	0,69	0	1			147630,33		1			1			101462,016	N-alpha-acetyltransferase 16, NatA auxiliary subunit OS=Homo sapiens OX=9606 GN=NAA16 PE=1 SV=2
1010	Q13370|PDE	32,29033	0,54	0	0	0,54	0	1			22121,885		1			2			124333,46	cGMP-inhibited 3',5'-cyclic phosphodiesterase 3B OS=Homo sapiens OX=9606 GN=PDE3B PE=1 SV=2
1115	P09848|LPH_	36,93215	0,31	0	0	0,31	0	1			259379,88		1			1		Deamidat	218586,7	Lactase/phlorizin hydrolase OS=Homo sapiens OX=9606 GN=LCT PE=1 SV=3
758	Q15043|S39A	177,8843	4,07	0	0	0	4,07	2				98584,875	2				2		54212,29	Metal cation symporter ZIP14 OS=Homo sapiens OX=9606 GN=SLC39A14 PE=1 SV=3
215	P02794|FRIH	338,0588	56,83	0	0	56,83	33,88	10			7829659,5	2782722,5	10			32	14	Carbamid	21225,635	Ferritin heavy chain OS=Homo sapiens OX=9606 GN=FTH1 PE=1 SV=2
233	O95302|FKBF	233,8221	15,26	15,26	1,4	0	0	11	1910999,4	23603,893			10	16	1			Carbamid	63083,56	Peptidyl-prolyl cis-trans isomerase FKBP9 OS=Homo sapiens OX=9606 GN=FKBP9 PE=1 SV=2
423	P06454|PTM	233,1557	34,23	34,23	34,23	0	0	4	802052,5	782548,25			4	6	7			Deamidat	12202,957	Prothymosin alpha OS=Homo sapiens OX=9606 GN=PTMA PE=1 SV=2
1116	Q96QR8|PUR	73,89225	1,92	1,92	1,92	0	0	1	1549437,4	1131782,1			1	1	2				33240,797	Transcriptional activator protein Pur-beta OS=Homo sapiens OX=9606 GN=PURB PE=1 SV=3
1025	Q8NGD0|OR	27,11249	1,92	0	0	0	1,92	1				76108,34	1				2		35488,484	Olfactory receptor 4M1 OS=Homo sapiens OX=9606 GN=OR4M1 PE=2 SV=1
606	O60361|NDK	95,26889	17,52	17,52	5,11	0	0	2	46544,055	32134,826			2	2	1				15529,057	Putative nucleoside diphosphate kinase OS=Homo sapiens OX=9606 GN=NME2P1 PE=5 SV=1
190	Q8N196|SIX5	35,53645	0,95	0,95	0,95	0	0	1	194988,3	63932,81			1	1	1				74561,51	Homeobox protein SIX5 OS=Homo sapiens OX=9606 GN=SIX5 PE=1 SV=3
259	P50281|MMF	311,2694	14,95	0	4,98	0	11,68	10		187334,03		1668642,2	10		4		8	Carbamid	65893,92	Matrix metalloproteinase-14 OS=Homo sapiens OX=9606 GN=MMP14 PE=1 SV=3
70	Q96F24|NRBI	83,96447	2,09	0	0	2,09	2,09	1			64976,27	250123	1			3	1		32377,709	Nuclear receptor-binding factor 2 OS=Homo sapiens OX=9606 GN=NRBF2 PE=1 SV=1
94	Q8IY92|SLX4	64,53661	0,33	0	0	0,33	0,33	1			68159,61	66022,19	1			1	1	Deamidat	200011,89	Structure-specific endonuclease subunit SLX4 OS=Homo sapiens OX=9606 GN=SLX4 PE=1 SV=3
376	P04908|H2A1	236,4661	32,31	6,92	6,92	6,92	32,31	4	230969,52	229668,45	250080,11	2471626,2	4	3	4	2	9		14135,49	Histone H2A type 1-B/E OS=Homo sapiens OX=9606 GN=H2AC4 PE=1 SV=2
341	Q12791|KCM	95,19555	0,49	0,49	0,49	0	0	1	361223,1	198103,61			1	1	2				137559,53	Calcium-activated potassium channel subunit alpha-1 OS=Homo sapiens OX=9606 GN=KCNMA1 PE=1 SV=2
36	Q15582|BGH	491,6603	45,39	45,1	26,35	27,38	37,19	40	8,13E+07	8628632	3129353,2	2,14E+07	39	163	61	34	67	Deamidat	74680,9	Transforming growth factor-beta-induced protein ig-h3 OS=Homo sapiens OX=9606 GN=TGFBI PE=1 SV=1
260	Q86Y22|CON	255,9993	13,15	7,59	7,22	5,74	2,78	10	1,33E+07	6322208,5	1375647,8	1687869,4	3	5	6	4	2	Deamidat	51943,918	Collagen alpha-1(XXIII) chain OS=Homo sapiens OX=9606 GN=COL23A1 PE=1 SV=1
880	Q4AC94|C2C	45,71551	0,25	0,25	0	0	0	1	1249522,2				1	2					260388,75	C2 domain-containing protein 3 OS=Homo sapiens OX=9606 GN=C2CD3 PE=1 SV=4
760	Q9H6B4|CLM	140,3378	5,36	1,88	1,88	0	5,36	2				54006,61	1	3	1		2		41280,715	CXADR-like membrane protein OS=Homo sapiens OX=9606 GN=CLMP PE=1 SV=1
328	Q9H6S0|YTD	38,18213	0,42	0,42	0,42	0	0	1	517809,8				1	1	1				160248,44	3'-5' RNA helicase YTHDC2 OS=Homo sapiens OX=9606 GN=YTHDC2 PE=1 SV=2
1117	Q92995|UBP	29,75328	0,7	0,7	0	0	0	1					1	1					97327,09	Ubiquitin carboxyl-terminal hydrolase 13 OS=Homo sapiens OX=9606 GN=USP13 PE=1 SV=2
135	P13942|COB	305,9681	7,37	3,86	3,92	1,79	1,38	18	108191,6			64975,94	3	18	18	7	6	Acetylatic	171790,67	Collagen alpha-2(XI) chain OS=Homo sapiens OX=9606 GN=COL11A2 PE=1 SV=5
313	P62328|TYB4	251,7636	70,45	70,45	54,55	38,64	54,55	7	1,66E+07	7176524	2235955,8	2156330,5	2	12	15	5	5	Acetylatic	5052,6504	Thymosin beta-4 OS=Homo sapiens OX=9606 GN=TMSB4X PE=1 SV=2
314	Q12884|SEPR	265,033	8,55	0	0	8,55	2,76	8			758727,5	532536,75	6			9	4		87712,555	Prolyl endopeptidase FAP OS=Homo sapiens OX=9606 GN=FAP PE=1 SV=5
56	Q8IUA7|ABC	80,91282	0,37	0	0	0,37	0,37	1			1044845,06	7799888	1			2	4		184361,55	ATP-binding cassette sub-family A member 9 OS=Homo sapiens OX=9606 GN=ABCA9 PE=1 SV=1
1118	Q14517|FAT1	24,03826	0,15	0	0	0,15	0	1					1			1			506272,75	Protocadherin Fat 1 OS=Homo sapiens OX=9606 GN=FAT1 PE=1 SV=2
447	P16403|H12_	217,6627	18,31	5,63	0	3,29	18,31	4				57619,617	2	1		1	4		21364,748	Histone H1.2 OS=Homo sapiens OX=9606 GN=H1-2 PE=1 SV=2
377	Q8N3J5|PPM	23,57562	1,61	0	1,61	0	0	1					1		1				40997,4	Protein phosphatase 1K, mitochondrial OS=Homo sapiens OX=9606 GN=PPM1K PE=1 SV=1
564	P61626|LYSC	187,4101	18,24	6,08	6,08	18,24	6,08	3	17649,137		217131,06	45524,383	3	1	1	4	1		16537,014	Lysozyme C OS=Homo sapiens OX=9606 GN=LYZ PE=1 SV=1
8	P10745|RET3	88,15943	0,48	0	0	0,48	0,48	1			140361,56	238290,47	1			1	1		135362,66	Retinol-binding protein 3 OS=Homo sapiens OX=9606 GN=RBP3 PE=1 SV=2
70	Q15149|PLEC	432,1411	6,38	3,27	0,15	3,42	1,13	36	505512,1	10706,196	528338,6	268013,22	28	19	1	21	7	Deamidat	531790,75	Plectin OS=Homo sapiens OX=9606 GN=PLEC PE=1 SV=3
249	A3KMH1|VW	79,5391	0,31	0,31	0,31	0,31	0,31	1	1444697,2	550149,44	488433,5	2769301	1	1	1	1	1		214824,45	von Willebrand factor A domain-containing protein 8 OS=Homo sapiens OX=9606 GN=VWA8 PE=1 SV=2
1119	P58499|FAM	51,50066	2,55	2,55	0	2,55	0	1	131393,34				1	1		2			25981,854	Protein FAM3B OS=Homo sapiens OX=9606 GN=FAM3B PE=1 SV=2
970	P63125|VPK2	27,98863	3,85	3,85	0	0	0	1	24682,994				1	2					17076,652	Endogenous retrovirus group K member 25 Pro protein OS=Homo sapiens OX=9606 GN=ERVK-25 PE=3 SV=1
761	E7EW31|PRO	86,17917	1,18	1,18	0,59	0	0	2					1	2	1				106917,14	Proline-rich basic protein 1 OS=Homo sapiens OX=9606 GN=PROB1 PE=2 SV=2
376	P0C0S8|H2A1	279,6636	37,69	11,54	12,31	6,92	37,69	6	259248,44	294746,4	250080,11	2714422,5	6	4	6	2	11		14091,478	Histone H2A type 1 OS=Homo sapiens OX=9606 GN=H2AC11 PE=1 SV=2
361	P02549|SPTA	22,78696	0,25	0	0	0	0,25	1					1				1		280014,47	Spectrin alpha chain, erythrocytic 1 OS=Homo sapiens OX=9606 GN=SPTA1 PE=1 SV=5
213	P20020|AT2B	239,2638	4,02	0	0	0	4,02	4				77202,6	2				7	Deamidat	134684,77	Plasma membrane calcium-transporting ATPase 1 OS=Homo sapiens OX=9606 GN=ATP2B1 PE=1 SV=4
1015	O43175|SER	24,13204	1,13	0	0	1,13	0	1					1			1			56650,496	D-3-phosphoglycerate dehydrogenase OS=Homo sapiens OX=9606 GN=PHGDH PE=1 SV=4
1120	P33240|CSTF	27,41865	1,21	0	1,21	1,21	1,21	1		165806,78	350671,44	453535,7	1		1	1	1		60959,473	Cleavage stimulation factor subunit 2 OS=Homo sapiens OX=9606 GN=CSTF2 PE=1 SV=1
157	P09525|ANX	392,4087	45,45	1,88	1,88	39,18	32,6	17			669193,9	834451,8	12	1	1	22	20	Carbamid	35882,71	Annexin A4 OS=Homo sapiens OX=9606 GN=ANXA4 PE=1 SV=4
136	P01033|TIMP	392,6346	67,15	67,15	18,84	0	0	18	8,90E+07	6135586			18	81	28			Carbamid	23170,863	Metalloproteinase inhibitor 1 OS=Homo sapiens OX=9606 GN=TIMP1 PE=1 SV=1
14	A6NNM3|RIN	100,3808	0,37	0,37	0,37	0	0,37	1	688577,7			23365,338	1	2	1		1	Carbamid	180951,69	RIMS-binding protein 3B OS=Homo sapiens OX=9606 GN=RIMBP3B PE=3 SV=3
849	Q504Y2|PKD	29,93155	1,22	1,22	0	0	0	1	608444,94				1	1					54131,637	Extracellular tyrosine-protein kinase PKDCC OS=Homo sapiens OX=9606 GN=PKDCC PE=1 SV=2
764	P98161|PKD1	50,35457	0,3	0	0	0,3	0	2			49049,004		1			2			462529,28	Polycystin-1 OS=Homo sapiens OX=9606 GN=PKD1 PE=1 SV=3
563	O00571|DDX	120,9849	1,36	1,36	1,36	1,36	1,36	2	44414,03	153360,47	33899,344	36529,64	2	1	3	1	1		73243,414	ATP-dependent RNA helicase DDX3X OS=Homo sapiens OX=9606 GN=DDX3X PE=1 SV=3
1121	O15083|ERC	34,83939	0,63	0	0	0	0,63	1				131281,55	1				1		110557,586	ERC protein 2 OS=Homo sapiens OX=9606 GN=ERC2 PE=1 SV=3
1122	Q9UI38|TSP5	29,25815	1,56	0	0	0	1,56	1				173536,34	1				1		43087,766	Probable threonine protease PRSS50 OS=Homo sapiens OX=9606 GN=PRSS50 PE=1 SV=1
849	Q969F9|HPS	29,93155	0,6	0,6	0	0	0	1	608444,94				1	1					113735,8	BLOC-2 complex member HPS3 OS=Homo sapiens OX=9606 GN=HPS3 PE=1 SV=1
20	Q9Y512|SAM	65,05981	1,28	0	0	1,28	1,28	1			245865,69	188108,73	1			1	1		51976,152	Sorting and assembly machinery component 50 homolog OS=Homo sapiens OX=9606 GN=SAMM50 PE=1 SV=3
1123	P54578|UBP1	23,45577	1,21	0	0	1,21	0	1			51930,484		1			1			56069,066	Ubiquitin carboxyl-terminal hydrolase 14 OS=Homo sapiens OX=9606 GN=USP14 PE=1 SV=3
169	Q9Y4F3|MAR	56,70879	0,34	0	0,34	0	0	1					1		3				192859,23	Meiosis regulator and mRNA stability factor 1 OS=Homo sapiens OX=9606 GN=MARF1 PE=1 SV=6
543	A6QL63|ABT	68,53143	0,54	0,54	0	0	0	1	70058,26				1	1					120884,07	Ankyrin repeat and BTB/POZ domain-containing protein 3 OS=Homo sapiens OX=9606 GN=ABTB3 PE=2 SV=3
767	P48634|PRC2	64,99181	0,6	0,32	0,6	0	0	2	869540,1	527850,4			1	1	3				228863,19	Protein PRRC2A OS=Homo sapiens OX=9606 GN=PRRC2A PE=1 SV=3
565	Q14204|DYH	70,47466	0,41	0,13	0,15	0,13	0	3		60718,45			1	1	1	1			532407,7	Cytoplasmic dynein 1 heavy chain 1 OS=Homo sapiens OX=9606 GN=DYNC1H1 PE=1 SV=5

769	Q7Z407|CSM	66,72437	0,32	0,16	0	0	0,16	2					1	1			1	Deamidat	405999,72	CUB and sushi domain-containing protein 3 OS=Homo sapiens OX=9606 GN=CSMD3 PE=2 SV=3
1124	Q96SB4|SRPI	24,63967	0,92	0	0	0,92	0	1					1			1			74324,77	SRSF protein kinase 1 OS=Homo sapiens OX=9606 GN=SRPK1 PE=1 SV=2
770	Q14185|DOC	104,3909	0,64	0,32	0,32	0,32	0,32	2					1	4	7	1	2		215345,83	Dedicator of cytokinesis protein 1 OS=Homo sapiens OX=9606 GN=DOCK1 PE=1 SV=2
1125	Q6ZS11|RINL	26,42003	1,41	0	1,41	0	0	1		8539,721			1		1				62466,305	Ras and Rab interactor-like protein OS=Homo sapiens OX=9606 GN=RINL PE=2 SV=2
1126	Q96KN1|LRA	24,18318	1,94	1,94	0	0	0	1	2349274,5				1	1					34474,273	Protein LRATD2 OS=Homo sapiens OX=9606 GN=LRATD2 PE=1 SV=1
903	P53597|SUC	50,07074	1,73	0	1,73	0	0	1		22226,035			1		1				36249,734	Succinate--CoA ligase [ADP/GDP-forming] subunit alpha, mitochondrial OS=Homo sapiens OX=9606 GN=SUCLG1 PE=1 SV=4
1127	A7E2Y1|MYH	57,97638	0,3	0,3	0	0,3	0,3	1			35560,82	142329,81	1	1		1	1		225844,86	Myosin-7B OS=Homo sapiens OX=9606 GN=MYH7B PE=1 SV=4
466	P35052|GPC1	110,9565	5,73	5,73	5,73	0	4,66	4	103106,11	140087,62		88024,234	4	4	4		3	Carbamid	61680,223	Glypican-1 OS=Homo sapiens OX=9606 GN=GPC1 PE=1 SV=2
123	Q13459|MYO	29,86649	0,28	0	0,28	0	0	1		253011,45			1		1				243400,86	Unconventional myosin-IXb OS=Homo sapiens OX=9606 GN=MYO9B PE=1 SV=3
896	Q9GZR7|DDX	23,04849	0,7	0	0	0,7	0	1			340053,22		1			1			96331,766	ATP-dependent RNA helicase DDX24 OS=Homo sapiens OX=9606 GN=DDX24 PE=1 SV=1
504	Q5T4F7|SFRP	34,65174	1,89	0	0	1,89	0	1			44113,773		1			1			35562,883	Secreted frizzled-related protein 5 OS=Homo sapiens OX=9606 GN=SFRP5 PE=2 SV=3
1126	Q7Z6J6|FRMI	24,18318	1,05	1,05	0	0	0	1	2349274,5				1	1					65064,676	FERM domain-containing protein 5 OS=Homo sapiens OX=9606 GN=FRMD5 PE=1 SV=1
659	Q5R372|RBG	42,47769	0,74	0	0,74	0	0	1		806622,25			1		3				92512,91	Rab GTPase-activating protein 1-like OS=Homo sapiens OX=9606 GN=RABGAP1L PE=1 SV=1
67	Q15678|PTN	100,2547	0,51	0,51	0	0,51	0,51	1	89758,81		142451,39	485845,22	1	1		1	1		135261	Tyrosine-protein phosphatase non-receptor type 14 OS=Homo sapiens OX=9606 GN=PTPN14 PE=1 SV=2
1128	Q9UGM3|DM	27,78274	0,25	0	0	0	0,25	1				75465,14	1				1		260735,4	Deleted in malignant brain tumors 1 protein OS=Homo sapiens OX=9606 GN=DMBT1 PE=1 SV=2
77	O00712|NFIB	47,90091	1,43	1,43	0	0	0	1					1	1					47441,9	Nuclear factor 1 B-type OS=Homo sapiens OX=9606 GN=NFIB PE=1 SV=2
67	Q01064|PDE	164,14674	1,12	1,12	0	0	1,12	1	31350,719			46152,96	1	1			1		61379,812	Dual specificity calcium/calmodulin-dependent 3',5'-cyclic nucleotide phosphodiesterase 1B OS=Homo sapiens OX=9606 GN=PDE1B PE=1 SV=2
59	P54296|MYO	68,65833	0,41	0,41	0,41	0,41	0	1	496466,3		1380152,2		1	1	1	1			164869,28	Myomesin-2 OS=Homo sapiens OX=9606 GN=MYOM2 PE=1 SV=3
1129	Q92901|RL3L	43,35169	1,72	0	0	0	1,72	1				33642,63	1				1		46295,973	60S ribosomal protein L3-like OS=Homo sapiens OX=9606 GN=RPL3L PE=1 SV=3
273	Q58FF7|H90	264,4146	11,56	7,04	0	9,88	6,37	7			48131,22		2	4		7	4		68324,836	Putative heat shock protein HSP 90-beta-3 OS=Homo sapiens OX=9606 GN=HSP90AB3P PE=5 SV=1
1130	P11310|ACA	24,77332	1,43	0	0	0	1,43	1					1				1		46588,324	Medium-chain specific acyl-CoA dehydrogenase, mitochondrial OS=Homo sapiens OX=9606 GN=ACADM PE=1 SV=1
276	Q86V35|CAB	107,5578	3,26	3,26	0	0	0	1	183098,98				1	2					24453,07	Calcium-binding protein 7 OS=Homo sapiens OX=9606 GN=CABP7 PE=1 SV=1
1131	Q14152|EIF3	49,0162	0,43	0	0,43	0	0	1		210161,86			1		2				166569,38	Eukaryotic translation initiation factor 3 subunit A OS=Homo sapiens OX=9606 GN=EIF3A PE=1 SV=1
1132	Q9UIC8|LCM	28,13937	1,8	0	0	0	1,8	1					1				1		38379,09	Leucine carboxyl methyltransferase 1 OS=Homo sapiens OX=9606 GN=LCMT1 PE=1 SV=2
343	A6NIZ1|RP1B	274,4999	19,02	0	0	19,02	15,22	6			123477,21	291905,78	3			5	5	Carbamid	20924,86	Ras-related protein Rap-1b-like protein OS=Homo sapiens OX=9606 GN=RAP1BL PE=2 SV=1
567	P62424|RL7A	90,1572	8,65	0	0	0	8,65	3				278155,12	2				3	Acetylatio	29995,62	60S ribosomal protein L7a OS=Homo sapiens OX=9606 GN=RPL7A PE=1 SV=2
495	Q5VT03|NTN	53,03056	0,74	0	0	0	0,74	1				29151,854	1				1	Deamidat	86276,09	NUT family member 2D OS=Homo sapiens OX=9606 GN=NUTM2D PE=3 SV=2
1133	Q9BXK5|B2L	30,00013	1,44	0	0	0	1,44	1				1595043,9	1				1		52723,16	Bcl-2-like protein 13 OS=Homo sapiens OX=9606 GN=BCL2L13 PE=1 SV=1
1134	A7MD48|SRR	24,32108	0,98	0	0	0,98	0	1					1			1			68558,63	Serine/arginine repetitive matrix protein 4 OS=Homo sapiens OX=9606 GN=SRRM4 PE=1 SV=2
570	Q8NHU6|TDF	55,0774	0,55	0	0	0	0,55	1				44577,832	1				1		123585,53	Tudor domain-containing protein 7 OS=Homo sapiens OX=9606 GN=TDRD7 PE=1 SV=2
187	O95965|ITGB	368,6488	28,74	28,74	0	0	0	14	4781200				14	23				Carbamid	53921,387	Integrin beta-like protein 1 OS=Homo sapiens OX=9606 GN=ITGBL1 PE=2 SV=1
1135	P39748|FEN1	50,21028	1,58	1,58	1,58	0	1,58	1	183264,42	201280,48		259869,7	1	1	1		2		42592,977	Flap endonuclease 1 OS=Homo sapiens OX=9606 GN=FEN1 PE=1 SV=1
1034	Q9GZQ8|MLF	34,97028	5,6	0	0	0	5,6	1				227544,36	1				1		14688,023	Microtubule-associated proteins 1A/1B light chain 3B OS=Homo sapiens OX=9606 GN=MAP1LC3B PE=1 SV=3
250	Q9NS61|KCIF	49,34548	2,22	0	0	0	2,22	1				673270,6	1				1	Deamidat	30906,8	Kv channel-interacting protein 2 OS=Homo sapiens OX=9606 GN=KCNIP2 PE=1 SV=3
634	Q9HD64|XAG	71,26441	7,41	0	7,41	7,41	7,41	1		4961949	219925,28	3924922	1		11	8	1	Deamidat	9077,76	X antigen family member 1 OS=Homo sapiens OX=9606 GN=XAGE1A PE=1 SV=3
773	O75348|VAT	150,3617	19,49	0	0	0	19,49	2				46711,55	2				2	Acetylatio	13757,514	V-type proton ATPase subunit G 1 OS=Homo sapiens OX=9606 GN=ATP6V1G1 PE=1 SV=3
252	Q99879|H2B1	309,2992	61,9	34,13	27,78	29,37	61,9	9	117890,07	89813,125	87343,49	987559,8	1	7	7	7	16	Oxidation	13989,268	Histone H2B type 1-M OS=Homo sapiens OX=9606 GN=H2BC14 PE=1 SV=3
1136	O75342|LX12	43,54945	1	0	0	1	0	1			34901,02		1			1			80355,66	Arachidonate 12-lipoxygenase, 12R-type OS=Homo sapiens OX=9606 GN=ALOX12B PE=1 SV=1
1137	Q96QD8|S38	54,57559	4,15	0	0	0	4,15	1				49646,17	1				1	Deamidat	56026,004	Sodium-coupled neutral amino acid symporter 2 OS=Homo sapiens OX=9606 GN=SLC38A2 PE=1 SV=2
706	O60812|HNR	43,84666	3,41	3,41	0	0	0	1	79983,87				1	2					32142,357	Heterogeneous nuclear ribonucleoprotein C-like 1 OS=Homo sapiens OX=9606 GN=HNRNPCL1 PE=1 SV=1
132	O15479|MAG	87,14804	2,19	2,19	0	0	0	1	58050,05				1	1					35277,035	Melanoma-associated antigen B2 OS=Homo sapiens OX=9606 GN=MAGEB2 PE=1 SV=3
1010	Q9P2C4|TM1	32,29033	0,98	0	0	0,98	0	1			22121,885		1			2			69324,586	Transmembrane protein 181 OS=Homo sapiens OX=9606 GN=TMEM181 PE=1 SV=2
388	Q8IY34|S15A	58,9107	1,03	1,03	0	0	0	1	83421,74				1	1					63559,5	Solute carrier family 15 member 3 OS=Homo sapiens OX=9606 GN=SLC15A3 PE=1 SV=2
468	O15417|TNC	112,9237	0,81	0,4	0	0,2	0,2	4			97208,92	1443703,5	2	2		1	2	Oxidation	314519,16	Trinucleotide repeat-containing gene 18 protein OS=Homo sapiens OX=9606 GN=TNRC18 PE=1 SV=3
773	O95670|VAT	77,70241	12,71	0	0	0	12,71	1					1				1	Acetylatio	13604,344	V-type proton ATPase subunit G 2 OS=Homo sapiens OX=9606 GN=ATP6V1G2 PE=1 SV=1
1093	Q92670|ZN7	55,59209	1,41	0	1,41	0	1,41	1					1		1		1		49753,33	Putative zinc finger protein 75C OS=Homo sapiens OX=9606 GN=ZNF75CP PE=5 SV=2
1138	A0A494C071|	63,96597	0,29	0	0,29	0,29	0,29	1		73975,664		240608,62	1		2	1	1		217772,84	PWWP domain-containing DNA repair factor 4 OS=Homo sapiens OX=9606 GN=PWWP4 PE=3 SV=1
205	Q5U4N7|GD	51,6067	2,8	2,8	0	0	0	1					1	1					28211,25	Protein GDF5-AS1, mitochondrial OS=Homo sapiens OX=9606 GN=GDF5-AS1 PE=5 SV=2
255	Q8NGS3|OR1	52,44891	1,86	1,86	1,86	1,86	1,86	1				236152,39	1	1	1	1	1		35493,066	Olfactory receptor 1J1 OS=Homo sapiens OX=9606 GN=OR1J1 PE=2 SV=1
997	Q9UQ07|MO	35,18394	1,43	0	1,43	0	0	1					1		1				48013,777	MAPK/MAK/MRK overlapping kinase OS=Homo sapiens OX=9606 GN=MOK PE=2 SV=1
1139	A4D1E1|Z804	23,92085	0,44	0	0	0	0,44	1				398026,66	1				1		152572,81	Zinc finger protein 804B OS=Homo sapiens OX=9606 GN=ZNF804B PE=2 SV=2
285	P30086|PEBP	294,7018	41,71	41,71	4,28	0	4,28	9	1009934,9	164841,23		123555,83	8	12	1		2	Deamidat	21056,78	Phosphatidylethanolamine-binding protein 1 OS=Homo sapiens OX=9606 GN=PEBP1 PE=1 SV=3
182	Q13885|TBB2	303,7431	20,45	0	0	20,45	5,39	9			2161750,8	176848,97	9			13	4	Oxidation	49906,97	Tubulin beta-2A chain OS=Homo sapiens OX=9606 GN=TUBB2A PE=1 SV=1
382	Q9Y281|COF	233,7425	22,89	10,84	4,82	11,45	18,07	5				34022,58	2	3	1	3	4	Acetylatio	18736,611	Cofilin-2 OS=Homo sapiens OX=9606 GN=CFL2 PE=1 SV=1
5	Q6UXM1|LRI	95,18884	0,54	0,54	0,54	0	0	1	7970265	233452,2			1	5	1				123433,73	Leucine-rich repeats and immunoglobulin-like domains protein 3 OS=Homo sapiens OX=9606 GN=LRIG3 PE=1 SV=1
1044	Q13263|TIF1	40,36036	0,72	0	0	0,72	0	1			63496,605		1			1			88549,66	Transcription intermediary factor 1-beta OS=Homo sapiens OX=9606 GN=TRIM28 PE=1 SV=5
306	Q86XL3|ANK	29,98162	0,64	0	0	0,64	0	1			40413,023		1			1			104114,39	Ankyrin repeat and LEM domain-containing protein 2 OS=Homo sapiens OX=9606 GN=ANKLE2 PE=1 SV=4
774	P22033|MUT	52,41708	1,6	0,8	0,8	0	0	2					1	2	1			Acetylatio	83134,445	Methylmalonyl-CoA mutase, mitochondrial OS=Homo sapiens OX=9606 GN=MMUT PE=1 SV=4
56	Q9NYU2|UGC	118,1989	0,39	0	0	0,39	0,39	1			1004440,4	6379385	1			2	4	Oxidation	177189,6	UDP-glucose:glycoprotein glucosyltransferase 1 OS=Homo sapiens OX=9606 GN=UGGT1 PE=1 SV=3
470	Q13439|GOG	65,52822	1,08	0,27	0	0,54	0,54	4	1978782,8			2,25E+07	1	2		2	2	Acetylatio	261140,17	Golgin subfamily A member 4 OS=Homo sapiens OX=9606 GN=GOLGA4 PE=1 SV=1
775	P48736|PK3C	113,2987	1,09	0,54	0	0,54	0,54	2	21360,566				1	1		2	1		126453,64	Phosphatidylinositol 4,5-bisphosphate 3-kinase catalytic subunit gamma isoform OS=Homo sapiens OX=9606 GN=PIK3CG PE=1 SV=3
223	Q8TDN2|KCN	106,1755	1,1	1,1	0	0	0	1					1	1					62459,117	Potassium voltage-gated channel subfamily V member 2 OS=Homo sapiens OX=9606 GN=KCNV2 PE=1 SV=1
252	O60814|H2B1	317,3283	66,67	34,13	27,78	29,37	66,67	10	117890,07	89813,125	87343,49	987559,8	1	7	7	7	17	Oxidation	13890,135	Histone H2B type 1-K OS=Homo sapiens OX=9606 GN=H2BC12 PE=1 SV=3
249	P0CG47|UBB	312,1252	26,64	26,64	22,71	26,64	26,64	8	7896435,5	3367563,2	1,28E+07	5,91E+07	8	34	29	35	49	Oxidation	25761,639	Polyubiquitin-B OS=Homo sapiens OX=9606 GN=UBB PE=1 SV=1
182	Q9H4B7|TBB	182,0134	5,32	0	0	5,32	5,32	3			459862,12	176848,97	3			4	4	Oxidation	50326,914	Tubulin beta-1 chain OS=Homo sapiens OX=9606 GN=TUBB1 PE=1 SV=1
605	P0DOX2|IGA	46,03342	1,54	0	0	0	1,54	1				41042,277	1				1	Carbamid	48934,168	Immunoglobulin alpha-2 heavy chain OS=Homo sapiens OX=9606 PE=1 SV=2
495	A6NNL0|NTN	53,03056	0,68	0	0	0	0,68	1				29151,854	1				1	Deamidat	93984,164	NUT family member 2B OS=Homo sapiens OX=9606 GN=NUTM2B PE=3 SV=2
63	Q02413|DSG	453,9006	29,55	0	0	27,84	25,26	25			1,63E+07	1,02E+07	23			74	64	Deamidat	113747,586	Desmoglein-1 OS=Homo sapiens OX=9606 GN=DSG1 PE=1 SV=2
391	Q9H0B3|IQC	62,39488	0,59	0	0	0,59	0	1					1			1			127692,99	IQ domain-containing protein N OS=Homo sapiens OX=9606 GN=IQCN PE=1 SV=1
70	Q9HCH5|SYT	66,2057	0,64	0,64	0	0	0	1	56448,73				1	1				Deamidat	104929,89	Synaptotagmin-like protein 2 OS=Homo sapiens OX=9606 GN=SYTL2 PE=1 SV=3
801	O43909|EXTL	23,08419	0,65	0	0,65	0	0	1		191882,12			1		1				104749,19	Exostosin-like 3 OS=Homo sapiens OX=9606 GN=EXTL3 PE=1 SV=1
172	Q09428|ABC	31,37971	0,38	0,38	0	0	0	1	135038,73				1	1					176992,28	ATP-binding cassette sub-family C member 8 OS=Homo sapiens OX=9606 GN=ABCC8 PE=1 SV=6
433	Q9H172|ABC	90,19837	0,93	0	0	0	0,93	1				62850,957	1				1	Carbamid	71895,68	ATP-binding cassette sub-family G member 4 OS=Homo sapiens OX=9606 GN=ABCG4 PE=1 SV=2
26	P21333|FLNA	543,6929	26,41	16,47	0,34	22,52	3,29	63	2723909,8	9366,504	3196084,2	268122,06	50	53	1	63	9	Deamidat	280739	Filamin-A OS=Homo sapiens OX=9606 GN=FLNA PE=1 SV=4
131	Q05329|DCE	26,63197	1,03	0	0	1,03	0	1					1			1			65411,277	Glutamate decarboxylase 2 OS=Homo sapiens OX=9606 GN=GAD2 PE=1 SV=1
601	O75140|DEP	35,8799	0,37	0	0,37	0	0	1					1		1				181263,78	GATOR complex protein DEPDC5 OS=Homo sapiens OX=9606 GN=DEPDC5 PE=1 SV=2
226	P35998|PRS7	82,66166	1,39	1,39	0	0	0	1	544477,3				1	1					48633,848	26S proteasome regulatory subunit 7 OS=Homo sapiens OX=9606 GN=PSMC2 PE=1 SV=3
1140	Q32P28|P3H	25,35469	0,95	0,95	0	0	0	1					1	1					83393,93	Prolyl 3-hydroxylase 1 OS=Homo sapiens OX=9606 GN=P3H1 PE=1 SV=2
1141	Q13310|PAB	27,24865	0,93	0	0	0,93	0	1					1			1			70782,85	Polyadenylate-binding protein 4 OS=Homo sapiens OX=9606 GN=PABPC4 PE=1 SV=1
1011	Q9H094|NBP	41,75044	0,95	0	0	0,95	0,95	1			5235523	7,28E+07	1			2	4		72950,97	Neuroblastoma breakpoint family member 3 OS=Homo sapiens OX=9606 GN=NBPF3 PE=2 SV=1
360	P43146|DCC_	26,32999	0,48	0	0	0,48	0	1					1			1			158456,53	Netrin receptor DCC OS=Homo sapiens OX=9606 GN=DCC PE=1 SV=2
6	Q8N6M0|OT	76,21806	2,05	2,05	0	2,05	2,05	1	95524,04		1177229,9	711697,1	1	1		2	1		33812,633	Deubiquitinase OTUD6B OS=Homo sapiens OX=9606 GN=OTUD6B PE=1 SV=1
471	Q9BXJ0|C1Q	198,8713	12,76	10,29	12,76	2,47	2,47	4	39222,496	117288,734			2	4	7	2	1		25298,412	Complement C1q tumor necrosis factor-related protein 5 OS=Homo sapiens OX=9606 GN=C1QTNF5 PE=1 SV=1
78	Q16821|PPR	80,14496	0,53	0,53	0,53	0	0	1					1	1	1				125766,7	Protein phosphatase 1 regulatory subunit 3A OS=Homo sapiens OX=9606 GN=PPP1R3A PE=1 SV=3
1142	O60637|TSN	32,90882	2,77	0	0	2,77	2,77	1			665564,1	2422809,8	1			2	2		28017,695	Tetraspanin-3 OS=Homo sapiens OX=9606 GN=TSPAN3 PE=2 SV=1
849	Q9GZV4|IF5	65,07362	5,23	0	0	5,23	0	1			30442,506		1			1			16793,197	Eukaryotic translation initiation factor 5A-2 OS=Homo sapiens OX=9606 GN=EIF5A2 PE=1 SV=3
1143	Q9H1H9|KI13	112,4307	0,33	0	0	0,33	0,33	1					1			1	1		202308,45	Kinesin-like protein KIF13A OS=Homo sapiens OX=9606 GN=KIF13A PE=1 SV=2
472	Q16720|AT2	200,0217	1,97	0	0	0	1,97	3				749081,9	1				4	Acetylatio	134197,12	Plasma membrane calcium-transporting ATPase 3 OS=Homo sapiens OX=9606 GN=ATP2B3 PE=1 SV=3
70	Q7RTT9|S29A	83,96447	1,13	0	0	1,13	1,13	1			64976,27	250123	1			3	1		58058,64	Equilibrative nucleoside transporter 4 OS=Homo sapiens OX=9606 GN=SLC29A4 PE=1 SV=1
261	P11279|LAM	306,7297	16,55	7,91	0	11,03	14,63	9	816199,06		821087,56	1,13E+07	8	6		9	14	Deamidat	44882,35	Lysosome-associated membrane glycoprotein 1 OS=Homo sapiens OX=9606 GN=LAMP1 PE=1 SV=3
63	Q16666|IF16_	56,32478	0,76	0	0	0,76	0,76	1			723076	773307	1			1	1		88255,55	Gamma-interferon-inducible protein 16 OS=Homo sapiens OX=9606 GN=IFI16 PE=1 SV=3
933	Q5VW38|GP	24,05968	1	0	0	1	0	1			27898,795		1			1			66990,38	Protein GPR107 OS=Homo sapiens OX=9606 GN=GPR107 PE=1 SV=1
345	P63313|TYB1	206,3084	77,27	77,27	61,36	0	61,36	5	6003143,5	3011176		1015537,75	2	8	8		5	Acetylatio	5025,671	Thymosin beta-10 OS=Homo sapiens OX=9606 GN=TMSB10 PE=1 SV=2
780	O00592|POD	93,12636	3,41	0	0	0	3,41	2				56207,53	2				2		58635,254	Podocalyxin OS=Homo sapiens OX=9606 GN=PODXL PE=1 SV=2
231	Q8WZ60|KLH	77,12096	0,97	0,97	0	0	0	1	430456,84				1	1				Carbamid	70358,89	Kelch-like protein 6 OS=Homo sapiens OX=9606 GN=KLHL6 PE=1 SV=3
626	P23763|VAM	96,59403	5,93	0	0	0	5,93	1				49215,074	1				1		12901,905	Vesicle-associated membrane protein 1 OS=Homo sapiens OX=9606 GN=VAMP1 PE=1 SV=1
114	Q13255|GRM	29,58683	0,5	0,5	0	0	0	1	19643,275				1	1					132357,16	Metabotropic glutamate receptor 1 OS=Homo sapiens OX=9606 GN=GRM1 PE=1 SV=3
1144	P27701|CD82	60,20209	3,75	0	0	0	3,75	1				254787,03	1				1		29625,512	CD82 antigen OS=Homo sapiens OX=9606 GN=CD82 PE=1 SV=1
782	Q8IZF6|AGR	52,58992	0,39	0,19	0	0,19	0	2	138262,1				1	1		1			333368,06	Adhesion G-protein coupled receptor G4 OS=Homo sapiens OX=9606 GN=ADGRG4 PE=1 SV=2
3	Q9UBX0|HES	41,04887	3,24	0	3,24	0	0	1		31847,021			1		1			Deamidat	21409,316	Homeobox expressed in ES cells 1 OS=Homo sapiens OX=9606 GN=HESX1 PE=1 SV=1
1145	Q96JN8|NEU	57,00203	0,38	0,38	0,38	0	0	1	358666,94	338899,4			1	1	1				166906,83	Neuralized-like protein 4 OS=Homo sapiens OX=9606 GN=NEURL4 PE=1 SV=2

1146	Q96QU8|XPC	27,50031	0,62	0	0	0	0,62	1					1				1	Acetylatic	128883,07	Exportin-6 OS=Homo sapiens OX=9606 GN=XPO6 PE=1 SV=1
145	P07858|CATE	375,7745	38,35	38,35	24,48	17,99	20,35	15	4,55E+07	2,84E+07	1779914,9	2856713,2	15	61	63	12	14	Oxidation	37821,58	Cathepsin B OS=Homo sapiens OX=9606 GN=CTSB PE=1 SV=3
87	Q9P2E2|KIF1	40,01609	0,58	0	0	0,58	0,58	1			127495,2	284008,75	1			1	1	Acetylatic	115067,82	Kinesin-like protein KIF17 OS=Homo sapiens OX=9606 GN=KIF17 PE=2 SV=3
1147	P11182|ODB	57,85664	1,24	1,24	1,24	0	0	1					1	1	1				53487,066	Lipoamide acyltransferase component of branched-chain alpha-keto acid dehydrogenase complex, mitochondrial OS=Homo sapiens OX=9606 GN=DBT PE=1 SV=3
1148	P07477|TRY1	46,73671	3,24	3,24	0	3,24	3,24	1	705888,9		71171,73	70945,36	1	3		1	1		26558,076	Serine protease 1 OS=Homo sapiens OX=9606 GN=PRSS1 PE=1 SV=1
7	P21860|ERBB	29,77415	0,45	0	0	0,45	0	1					1			1			148098,19	Receptor tyrosine-protein kinase erbB-3 OS=Homo sapiens OX=9606 GN=ERBB3 PE=1 SV=1
346	P35900|K1C2	259,428	7,31	0	0	5,9	5,66	7			147630,33		1			23	16		48486,61	Keratin, type I cytoskeletal 20 OS=Homo sapiens OX=9606 GN=KRT20 PE=1 SV=1
1023	Q15262|PTPF	93,72565	0,42	0,42	0	0	0	1	19121,674				1	1					162102,14	Receptor-type tyrosine-protein phosphatase kappa OS=Homo sapiens OX=9606 GN=PTPRK PE=1 SV=2
232	Q9UIW2|PLX	99,11502	0,32	0,32	0	0	0	1	47504,8				1	1				Carbamid	211067,05	Plexin-A1 OS=Homo sapiens OX=9606 GN=PLXNA1 PE=1 SV=3
569	Q8NDH2|CC1	64,39674	0,27	0	0	0,1	0,17	3					1			1	3	Acetylatio	801911,75	Leucine-rich repeat transmembrane protein CCDC168 OS=Homo sapiens OX=9606 GN=CCDC168 PE=2 SV=3
210	P20711|DDC_	93,67598	1,46	1,46	0	0	0	1	235215,75				1	1					53926,34	Aromatic-L-amino-acid decarboxylase OS=Homo sapiens OX=9606 GN=DDC PE=1 SV=2
384	Q9HCY8|S10/	282,3607	56,73	0	0	56,73	15,38	6			1559515	93728,82	5			7	2	Carbamid	11662,089	Protein S100-A14 OS=Homo sapiens OX=9606 GN=S100A14 PE=1 SV=1
1149	Q9ULT6|ZNR	25,97557	0,75	0	0	0	0,75	1				66483,875	1				1	Deamidat	100573,555	E3 ubiquitin-protein ligase ZNRF3 OS=Homo sapiens OX=9606 GN=ZNRF3 PE=1 SV=3
262	P15586|GNS_	333,6491	15,58	15,58	14,31	1,09	0	10	872957,5	1085494,2			9	18	29	4		Carbamid	62082,086	N-acetylglucosamine-6-sulfatase OS=Homo sapiens OX=9606 GN=GNS PE=1 SV=3
786	Q13085|ACA	101,8058	0,51	0	0,26	0,26	0,26	2		204327,75			1		1	2	2		265553,8	Acetyl-CoA carboxylase 1 OS=Homo sapiens OX=9606 GN=ACACA PE=1 SV=2
787	A1X283|SPD	89,1443	1,32	0,66	0,66	0,66	1,32	2				264093,28	1	2	3	5	2		101579,086	SH3 and PX domain-containing protein 2B OS=Homo sapiens OX=9606 GN=SH3PXD2B PE=1 SV=3
146	P26022|PTX3	383,705	40,68	37,8	30,97	27,82	27,03	16	1,24E+07	4332764	1155696,6	892047,44	14	61	55	17	15	Carbamid	41975,598	Pentraxin-related protein PTX3 OS=Homo sapiens OX=9606 GN=PTX3 PE=1 SV=3
250	Q6PIL6|KCIP	49,34548	2,4	0	0	0	2,4	1				673270,6	1				1	Deamidat	28728,658	Kv channel-interacting protein 4 OS=Homo sapiens OX=9606 GN=KCNIP4 PE=1 SV=1
40	Q6P4I2|WDR	86,31996	1,59	0	0	1,59	1,59	1			150778,75	195502,83	1			1	1		41685,066	WD repeat-containing protein 73 OS=Homo sapiens OX=9606 GN=WDR73 PE=1 SV=1
416	Q6ZV73|FGD	67,33826	0,42	0	0	0,42	0,42	1			57205,96	191863,17	1			1	1		160816,33	FYVE, RhoGEF and PH domain-containing protein 6 OS=Homo sapiens OX=9606 GN=FGD6 PE=1 SV=2
104	P11021|BIP_	444,5968	42,66	38,84	19,88	29,05	16,67	26	4613616	562995,7	815101,3	267109,62	23	38	19	29	16		72332,95	Endoplasmic reticulum chaperone BiP OS=Homo sapiens OX=9606 GN=HSPA5 PE=1 SV=2
1150	Q9NSD9|SYF	23,18189	1,02	0	0	1,02	0	1			86965,13		1			1			66115,61	Phenylalanine--tRNA ligase beta subunit OS=Homo sapiens OX=9606 GN=FARSB PE=1 SV=3
305	P50453|SPB9	33,78748	1,6	1,6	0	0	0	1	253790,36				1	3					42403,605	Serpin B9 OS=Homo sapiens OX=9606 GN=SERPINB9 PE=1 SV=1
35	P40222|TXLN	88,33254	1,1	1,1	1,1	1,1	1,1	1	87085,7	56296,742	38004,312	115726,03	1	1	1	1	1		61891,11	Alpha-taxilin OS=Homo sapiens OX=9606 GN=TXLNA PE=1 SV=3
1011	Q3BBV0|NBF	41,75044	0,49	0	0	0,49	0,49	1			5235523	7,28E+07	1			2	4		139257,7	Neuroblastoma breakpoint family member 1 OS=Homo sapiens OX=9606 GN=NBPF1 PE=1 SV=2
788	A0A1B0GUA7	98,86685	7,23	7,23	3,61	0	0	2	726644,5				1	2	2				18841,781	Testis-expressed protein 51 OS=Homo sapiens OX=9606 GN=TEX51 PE=3 SV=1
137	Q13733|AT1/	240,635	3,98	0	0	0,78	3,98	5				127091,92	4			1	5	Carbamid	114166,46	Sodium/potassium-transporting ATPase subunit alpha-4 OS=Homo sapiens OX=9606 GN=ATP1A4 PE=1 SV=3
206	A0A075B767|	269,5659	28,05	11,59	11,59	28,05	28,05	6	212291,48	104125,625	604586,06	671093,94	4	3	3	9	8	Carbamid	18207,826	Peptidyl-prolyl cis-trans isomerase A-like 4H OS=Homo sapiens OX=9606 GN=PPIAL4H PE=3 SV=1
424	P61026|RAB1	173,7243	24	0	0	9	18,5	5				47354,258	2			3	4		22540,928	Ras-related protein Rab-10 OS=Homo sapiens OX=9606 GN=RAB10 PE=1 SV=1
411	Q9NZN3|EHD	100,7973	2,62	0	0	0	2,62	2				231048,6	2				4		60887,12	EH domain-containing protein 3 OS=Homo sapiens OX=9606 GN=EHD3 PE=1 SV=2
596	P04181|OAT_	28,70844	1,37	0	1,37	0	0	1					1		1				48534,832	Ornithine aminotransferase, mitochondrial OS=Homo sapiens OX=9606 GN=OAT PE=1 SV=1
78	A2RRP1|NBA	83,70251	0,25	0,25	0,25	0	0	1					1	1	1				268571,3	NBAS subunit of NRZ tethering complex OS=Homo sapiens OX=9606 GN=NBAS PE=1 SV=2
1050	P0CG04|IGLC	87,25446	5,66	5,66	0	0	0	1					1	1					11347,659	Immunoglobulin lambda constant 1 OS=Homo sapiens OX=9606 GN=IGLC1 PE=1 SV=1
782	O43638|FOX	26,82563	1,82	1,82	0	0	0	1	138262,1				1	1					35434,336	Forkhead box protein S1 OS=Homo sapiens OX=9606 GN=FOXS1 PE=2 SV=2
28	P63316|TNN	99,10413	4,35	4,35	0	4,35	4,35	1	231770,69		55830,977	247574,6	1	2		1	1		18402,506	Troponin C, slow skeletal and cardiac muscles OS=Homo sapiens OX=9606 GN=TNNC1 PE=1 SV=1
385	O96006|ZBED	53,32415	0,86	0	0	0,86	0	1			73782,05		1			1			78155,56	E3 SUMO-protein ligase ZBED1 OS=Homo sapiens OX=9606 GN=ZBED1 PE=1 SV=1
570	P62701|RS4X	96,2551	7,22	0	0	0	7,22	3				94240,53	2				3		29597,723	40S ribosomal protein S4, X isoform OS=Homo sapiens OX=9606 GN=RPS4X PE=1 SV=2
169	Q9HCU0|CD2	355,3056	17,04	10,96	13,87	0	2,11	16	1049398	1869714,1		407823,75	14	13	25		2	Carbamid	80859,234	Endosialin OS=Homo sapiens OX=9606 GN=CD248 PE=1 SV=1
138	Q12789|TF3C	116,2287	0,28	0	0	0,28	0,28	1				62876,43	1			1	1		238874,78	General transcription factor 3C polypeptide 1 OS=Homo sapiens OX=9606 GN=GTF3C1 PE=1 SV=4
105	Q15113|PCO	426,2405	48,78	48,78	40,09	7,35	26,5	22	2,01E+07	1,01E+07	201797,16	2071283,4	22	80	61	3	14	Carbamid	47972,484	Procollagen C-endopeptidase enhancer 1 OS=Homo sapiens OX=9606 GN=PCOLCE PE=1 SV=2
1151	Q0VF96|CGN	24,39987	0,46	0,46	0	0	0	1	1334153,1				1	1					149079,19	Cingulin-like protein 1 OS=Homo sapiens OX=9606 GN=CGNL1 PE=1 SV=2
29	Q12768|WAS	73,83007	0,52	0,52	0,52	0,52	0,52	1	353738,47	228728,16		221029,77	1	2	2	2	2		134286,47	WASH complex subunit 5 OS=Homo sapiens OX=9606 GN=WASHC5 PE=1 SV=1
571	P16401|H15_	136,1724	14,16	7,08	0	3,1	7,08	3	21698,6				1	1		1	2	Acetylatic	22580,17	Histone H1.5 OS=Homo sapiens OX=9606 GN=H1-5 PE=1 SV=3
1070	Q9P0U4|CXX	24,50114	0,91	0	0,91	0	0	1					1		1				75711,55	CXXC-type zinc finger protein 1 OS=Homo sapiens OX=9606 GN=CXXC1 PE=1 SV=2
1152	Q8WYR1|PI3	25,73304	0,68	0	0	0	0,68	1					1				1		97347,62	Phosphoinositide 3-kinase regulatory subunit 5 OS=Homo sapiens OX=9606 GN=PIK3R5 PE=1 SV=1
901	E9PJ23|NPIB	32,753	1,41	1,41	0	0	0	1	228840,3				1	1				Deamidat	49162,21	Nuclear pore complex-interacting protein family member B6 OS=Homo sapiens OX=9606 GN=NPIPB6 PE=3 SV=1
324	P07205|PGK2	100,7334	1,44	0	0	1,44	0	1					1			1			44796,113	Phosphoglycerate kinase 2 OS=Homo sapiens OX=9606 GN=PGK2 PE=1 SV=3
926	Q99459|CDC	24,46977	0,75	0	0	0	0,75	1				95245,69	1				1		92250,63	Cell division cycle 5-like protein OS=Homo sapiens OX=9606 GN=CDC5L PE=1 SV=2
1153	Q9NZU1|FLR	24,38223	0,93	0	0,93	0	0	1					1		1				71358,41	Leucine-rich repeat transmembrane protein FLRT1 OS=Homo sapiens OX=9606 GN=FLRT1 PE=1 SV=3
1154	P15291|B4GT	27,59225	1,76	1,76	1,76	0	0	1	29193,719				1	1	1			Carbamid	43920,266	Beta-1,4-galactosyltransferase 1 OS=Homo sapiens OX=9606 GN=B4GALT1 PE=1 SV=5
89	Q6ZTR7|CBA	62,23655	1,97	1,97	1,97	1,97	1,97	1	187174,31	68203,37			1	1	1	2	1		34754,582	CBY1-interacting BAR domain-containing protein 2 OS=Homo sapiens OX=9606 GN=CIBAR2 PE=1 SV=2
988	P14210|HGF_	29,20635	0,82	0	0	0,82	0	1			88342,03		1			1			83133,69	Hepatocyte growth factor OS=Homo sapiens OX=9606 GN=HGF PE=1 SV=2
43	P12035|K2C3	466,1995	24,84	7,17	10,99	23,73	22,13	35				53216,176	2	13	32	189	117	Oxidation	64416,99	Keratin, type II cytoskeletal 3 OS=Homo sapiens OX=9606 GN=KRT3 PE=1 SV=3
249	Q9UHD9|UBC	100,8806	0,96	0,96	0,96	0,96	0,96	1	1704077,6	705186,9	3805421,8	1,56E+07	1	6	7	6	4		65696,08	Ubiquilin-2 OS=Homo sapiens OX=9606 GN=UBQLN2 PE=1 SV=2
1155	Q96IS3|RAX2	43,57881	3,26	0	3,26	0	0	1		50454,98			1		1				20085,873	Retina and anterior neural fold homeobox protein 2 OS=Homo sapiens OX=9606 GN=RAX2 PE=1 SV=1
81	Q9P2F6|RHG	40,82134	0,5	0	0,5	0	0	1					1		2				132607,97	Rho GTPase-activating protein 20 OS=Homo sapiens OX=9606 GN=ARHGAP20 PE=1 SV=2
770	Q9NX58|LYA	42,8563	1,58	0	0	1,58	0	1					1			1			43633,844	Cell growth-regulating nucleolar protein OS=Homo sapiens OX=9606 GN=LYAR PE=1 SV=3
1145	P10243|MYB	57,00203	0,8	0,8	0,8	0	0	1	358666,94	338899,4			1	1	1				85886,9	Myb-related protein A OS=Homo sapiens OX=9606 GN=MYBL1 PE=1 SV=2
44	Q09666|AHN	502,4406	8,46	1,05	0,25	8,1	2,41	44	473670,62	28436,375	6713711,5	1382823,9	41	9	3	69	16	Oxidation	629101,25	Neuroblast differentiation-associated protein AHNAK OS=Homo sapiens OX=9606 GN=AHNAK PE=1 SV=2
42	P25391|LAM	77,16082	0,52	0,52	0	0	0	2					2	2					337083,8	Laminin subunit alpha-1 OS=Homo sapiens OX=9606 GN=LAMA1 PE=1 SV=2
849	Q92918|M4K	29,93155	0,72	0,72	0	0	0	1	608444,94				1	1					91296,43	Mitogen-activated protein kinase kinase kinase kinase 1 OS=Homo sapiens OX=9606 GN=MAP4K1 PE=1 SV=1
1067	Q9NT68|TEN	24,35381	0,22	0	0	0,22	0	1			37122,6		1			1			307787,47	Teneurin-2 OS=Homo sapiens OX=9606 GN=TENM2 PE=1 SV=3
1032	Q9BQD7|ANI	47,494	2,55	2,55	2,55	0	0	1	304970,78	254798,3			1	1	2				25130,025	Adenine nucleotide translocase lysine N-methyltransferase OS=Homo sapiens OX=9606 GN=ANTKMT PE=1 SV=1
793	Q15386|UBE	103,6519	1,2	0	0	0,55	1,2	2					1			2	2		123922,63	Ubiquitin-protein ligase E3C OS=Homo sapiens OX=9606 GN=UBE3C PE=1 SV=3
1035	Q6ZUS6|CC1	23,76196	1,27	0	0	1,27	0	1			231974,8		1			1			52796,19	Coiled-coil domain-containing protein 149 OS=Homo sapiens OX=9606 GN=CCDC149 PE=2 SV=2
473	P31949|S10A	178,0612	29,52	20,95	20,95	29,52	15,24	4	2189978,5	377368,3	1636584,1	363097,7	4	5	4	5	2		11740,44	Protein S100-A11 OS=Homo sapiens OX=9606 GN=S100A11 PE=1 SV=2
1010	Q6VY07|PAC	32,29033	0,62	0	0	0,62	0	1			22121,885		1			2			104898,44	Phosphofurin acidic cluster sorting protein 1 OS=Homo sapiens OX=9606 GN=PACS1 PE=1 SV=2
1156	Q9HCD6|TAN	27,61164	0,45	0	0	0	0,45	1					1				1		219649,61	Protein TANC2 OS=Homo sapiens OX=9606 GN=TANC2 PE=1 SV=3
1157	Q6P9H5|GIM	42,52576	2,05	0	0	2,05	0	1			129776,97		1			1			32948,797	GTPase IMAP family member 6 OS=Homo sapiens OX=9606 GN=GIMAP6 PE=2 SV=1
474	P13987|CD59	248,5769	35,94	0	0	35,94	35,94	4			1307848,5	5340297	4			5	14	Carbamid	14177,284	CD59 glycoprotein OS=Homo sapiens OX=9606 GN=CD59 PE=1 SV=1
1032	Q9UKP4|ATS	47,494	0,36	0,36	0,36	0	0	1	304970,78	254798,3			1	1	2				184094,83	A disintegrin and metalloproteinase with thrombospondin motifs 7 OS=Homo sapiens OX=9606 GN=ADAMTS7 PE=1 SV=2
1071	Q9NZ71|RTEI	27,42501	0,49	0	0,49	0	0	1					1		1				133682,83	Regulator of telomere elongation helicase 1 OS=Homo sapiens OX=9606 GN=RTEL1 PE=1 SV=2
1158	Q14409|GLPK	36,17757	1,27	0	0	1,27	0	1					1			1			60597,887	Glycerol kinase 3 OS=Homo sapiens OX=9606 GN=GK3 PE=2 SV=2
347	P35222|CTNE	281,3612	8,83	0	0	8,83	2,82	7			7829901,5		1			14	4	Carbamid	85496,58	Catenin beta-1 OS=Homo sapiens OX=9606 GN=CTNNB1 PE=1 SV=1
425	O95297|MPZ	144,5781	15,99	0	0	0	15,99	5				435361,25	5				5		29082,18	Myelin protein zero-like protein 1 OS=Homo sapiens OX=9606 GN=MPZL1 PE=1 SV=1
384	Q9NYB9|ABI	108,8513	1,17	0	0	1,17	0	1			1303591,6		1			2			55663,23	Abl interactor 2 OS=Homo sapiens OX=9606 GN=ABI2 PE=1 SV=1
933	Q6XQN6|PN	24,05968	1,12	0	0	1,12	0	1			27898,795		1			1			57578,223	Nicotinate phosphoribosyltransferase OS=Homo sapiens OX=9606 GN=NAPRT PE=1 SV=2
573	Q9BRK3|MXF	112,3165	6,33	0	1,81	0	4,52	3		27514,89		642700,1	3		1		4		49131,543	Matrix remodeling-associated protein 8 OS=Homo sapiens OX=9606 GN=MXRA8 PE=1 SV=1
516	Q9HAV0|GBE	150,8359	7,06	0	0	7,06	2,06	3			163266,73		1			4	1	Acetylatic	37567,18	Guanine nucleotide-binding protein subunit beta-4 OS=Homo sapiens OX=9606 GN=GNB4 PE=1 SV=3
574	Q5T0F9|C2D1	94,36231	2,1	1,4	0	0,7	0,7	3	127675,88		43950,754		1	2		1	1		94224,44	Coiled-coil and C2 domain-containing protein 1B OS=Homo sapiens OX=9606 GN=CC2D1B PE=1 SV=1
252	Q93079|H2B	317,3283	66,67	34,13	27,78	29,37	66,67	10	117890,07	89813,125	87343,49	987559,8	1	7	7	7	17	Oxidation	13892,107	Histone H2B type 1-H OS=Homo sapiens OX=9606 GN=H2BC9 PE=1 SV=3
796	P49411|EFTU	43,1811	5,49	0	0	5,49	0	2					2			2		Acetylatio	49874,934	Elongation factor Tu, mitochondrial OS=Homo sapiens OX=9606 GN=TUFM PE=1 SV=3
1159	Q8N697|S15	26,28301	1,39	0	0	0	1,39	1				49872,387	1				1	Carbamid	62033,54	Solute carrier family 15 member 4 OS=Homo sapiens OX=9606 GN=SLC15A4 PE=1 SV=1
475	A0AVI2|FR1L	201,35	1,46	0,29	0,29	0,29	1,17	4				2624083,2	1	1	1	1	4		237934,83	Fer-1-like protein 5 OS=Homo sapiens OX=9606 GN=FER1L5 PE=2 SV=3
1036	Q9Y5X3|SNX	27,37833	1,49	0	0	1,49	0	1			31607,432		1			1			46816,49	Sorting nexin-5 OS=Homo sapiens OX=9606 GN=SNX5 PE=1 SV=1
575	P23490|LORI	207,942	6,73	0	0	6,73	6,73	3			502759,88	992096,44	3			5	4	Carbamid	25760,658	Loricrin OS=Homo sapiens OX=9606 GN=LORICRIN PE=1 SV=2
382	Q9HCE1|MO	42,21737	0,6	0	0	0,6	0,6	1				34022,58	1			1	1	Acetylatic	113671,28	Helicase MOV-10 OS=Homo sapiens OX=9606 GN=MOV10 PE=1 SV=2
1095	P28161|GSTM	24,87802	2,75	2,75	0	0	0	1	328019,5				1	1					25744,65	Glutathione S-transferase Mu 2 OS=Homo sapiens OX=9606 GN=GSTM2 PE=1 SV=2
1160	P30048|PRD	24,41807	2,34	0	0	0	2,34	1				5785744,5	1				1	Acetylatic	27692,65	Thioredoxin-dependent peroxide reductase, mitochondrial OS=Homo sapiens OX=9606 GN=PRDX3 PE=1 SV=3
94	Q9H4A9|DPE	96,43889	1,44	0	0	0	1,44	1				75865,734	1				1		53365,027	Dipeptidase 2 OS=Homo sapiens OX=9606 GN=DPEP2 PE=1 SV=3
816	Q15116|PDCI	27,74358	2,08	0	2,08	0	0	1		970712,75			1		1				31646,908	Programmed cell death protein 1 OS=Homo sapiens OX=9606 GN=PDCD1 PE=1 SV=3
801	Q96DV4|RM3	53,12333	3,16	0	1,58	1,58	0	2		191882,12			1		1	1			44596,51	39S ribosomal protein L38, mitochondrial OS=Homo sapiens OX=9606 GN=MRPL38 PE=1 SV=2
158	P51149|RAB7	391,1432	73,91	0	0	50,72	66,18	15			1361391,4	7447109	14			19	31	Carbamid	23489,742	Ras-related protein Rab-7a OS=Homo sapiens OX=9606 GN=RAB7A PE=1 SV=1
56	O00560|SDCE	428,1867	66,78	0	0	59,73	66,78	23			9572979	6,04E+07	22			43	109	Carbamid	32444,385	Syntenin-1 OS=Homo sapiens OX=9606 GN=SDCBP PE=1 SV=1
263	P29400|CO4	278,2009	3,8	2,2	3,09	1,9	1,19	9	451397,22	286018			1	16	17	9	7	Acetylatic	161043,89	Collagen alpha-5(IV) chain OS=Homo sapiens OX=9606 GN=COL4A5 PE=1 SV=2
42	P24043|LAM	469,9443	12,04	10,03	3,88	3,04	2,4	47	2673547,2	383692,62	318459,62	54969,727	41	47	16	14	9	Carbamid	343905,4	Laminin subunit alpha-2 OS=Homo sapiens OX=9606 GN=LAMA2 PE=1 SV=4
283	O43790|KRT	305,0909	11,73	1,44	1,44	6,58	9,26	8			185751,38	356090,03	6	2	2	8	9	Carbamid	53500,62	Keratin, type II cuticular Hb6 OS=Homo sapiens OX=9606 GN=KRT86 PE=1 SV=1
1161	P40939|ECHA	30,80067	0,92	0	0	0,92	0	1			214268,1		1			2			82999,64	Trifunctional enzyme subunit alpha, mitochondrial OS=Homo sapiens OX=9606 GN=HADHA PE=1 SV=2
596	Q5VUE5|CA0	28,70844	4,14	0	4,14	0	0	1					1		1				15482,59	Uncharacterized protein C1orf53 OS=Homo sapiens OX=9606 GN=C1orf53 PE=1 SV=1
578	P22105|TENX	137,2248	0,59	0,21	0	0,21	0,16	3			31635,004	1155561,1	3	1		1	2	Carbamid	458388,5	Tenascin-X OS=Homo sapiens OX=9606 GN=TNXB PE=1 SV=5
692	Q8WXD9|CSI	29,85037	0,42	0,42	0	0	0	1	62285,676				1	1					149813,69	Caskin-1 OS=Homo sapiens OX=9606 GN=CASKIN1 PE=1 SV=1

1045	Q13207|TBX2	29,49904	0,84	0,84	0	0	0	1					1	1					75066,055	T-box transcription factor TBX2 OS=Homo sapiens OX=9606 GN=TBX2 PE=1 SV=3
220	Q9NRM1|EN	59,98161	0,53	0	0	0,53	0	1			32276,74		1			1			128784,875	Enamelin OS=Homo sapiens OX=9606 GN=ENAM PE=1 SV=3
1157	Q9Y6T7|DGKI	42,52576	0,75	0	0	0,75	0	1			129776,97		1			1			90595,305	Diacylglycerol kinase beta OS=Homo sapiens OX=9606 GN=DGKB PE=1 SV=2
87	Q8N2N9|AN	40,01609	0,44	0	0	0,44	0,44	1			127495,2	284008,75	1			1	1	Acetylatic	153582,4	Ankyrin repeat domain-containing protein 36B OS=Homo sapiens OX=9606 GN=ANKRD36B PE=1 SV=4
1162	Q9Y2H5|PKH	24,21256	0,57	0	0	0	0,57	1					1				1	Deamidat	117127,98	Pleckstrin homology domain-containing family A member 6 OS=Homo sapiens OX=9606 GN=PLEKHA6 PE=1 SV=4
579	Q9NVJ2|ARL	176,2725	12,9	0	0	4,84	12,9	3			156330,28	619376,06	1			1	4		21539,01	ADP-ribosylation factor-like protein 8B OS=Homo sapiens OX=9606 GN=ARL8B PE=1 SV=1
9	Q9BXX2|AN3	23,09759	0,43	0	0	0,43	0	1			30291,865		1			1			158048,56	Ankyrin repeat domain-containing protein 30B OS=Homo sapiens OX=9606 GN=ANKRD30B PE=2 SV=3
804	P04216|THY1	176,9376	14,91	0	0	14,91	14,91	2			327133,34	799828,9	2			7	8	Carbamid	17934,717	Thy-1 membrane glycoprotein OS=Homo sapiens OX=9606 GN=THY1 PE=1 SV=2
96	Q96P44|COL	41,76144	1,25	0,63	0,63	0	0	2	609901,94				1	1	1				99368,58	Collagen alpha-1(XXI) chain OS=Homo sapiens OX=9606 GN=COL21A1 PE=2 SV=1
234	P50454|SERP	333,5313	27,27	27,27	0	8,37	0	11	459671		90230,9		10	16		3		Carbamid	46440,535	Serpin H1 OS=Homo sapiens OX=9606 GN=SERPINH1 PE=1 SV=2
29	Q8TEC5|SH3F	76,83936	0,82	0,82	0,82	0,82	0,82	1	316596,94	143501,84	40038,086	92368,01	1	2	1	1	1	Carbamid	79319,7	E3 ubiquitin-protein ligase SH3RF2 OS=Homo sapiens OX=9606 GN=SH3RF2 PE=1 SV=3
426	Q9Y6C2|EMIL	183,2802	4,43	0	2,95	0,59	1,57	5		29613,393		81775,78	4		3	2	2	Acetylatic	106695,13	EMILIN-1 OS=Homo sapiens OX=9606 GN=EMILIN1 PE=1 SV=3
682	P68106|FKB1	59,72122	5,56	0	0	0	5,56	1					1				1		11782,503	Peptidyl-prolyl cis-trans isomerase FKBP1B OS=Homo sapiens OX=9606 GN=FKBP1B PE=1 SV=2
216	P09382|LEG1	311,2103	71,85	60	7,41	59,26	35,56	11	2593744,8	348144,7	2143241,5	713918,4	11	14	2	13	6	Acetylatic	14715,698	Galectin-1 OS=Homo sapiens OX=9606 GN=LGALS1 PE=1 SV=2
1163	Q8N1G4|LRC	27,92262	1,03	1,03	0	0	0	1	17615,307				1	1				Acetylatic	63472,938	Leucine-rich repeat-containing protein 47 OS=Homo sapiens OX=9606 GN=LRRC47 PE=1 SV=1
235	P10619|PPGE	320,0785	20,62	20,62	15,62	2,71	0	11	1716971,2	1167366,8			11	12	17	1		Carbamid	54466,074	Lysosomal protective protein OS=Homo sapiens OX=9606 GN=CTSA PE=1 SV=2
807	Q14789|GOG	88,11439	0,37	0	0	0,37	0,18	2			108264,164		1			2	6		376019,16	Golgin subfamily B member 1 OS=Homo sapiens OX=9606 GN=GOLGB1 PE=1 SV=2
1164	P02749|APOI	61,38475	2,61	2,61	0	0	0	1	128090,195				1	1				Carbamid	38298,152	Beta-2-glycoprotein 1 OS=Homo sapiens OX=9606 GN=APOH PE=1 SV=3
710	Q6IA69|NAD	27,04443	0,85	0	0	0,85	0	1					1			1			79284,73	Glutamine-dependent NAD(+) synthetase OS=Homo sapiens OX=9606 GN=NADSYN1 PE=1 SV=3
348	Q8WWI5|CTL	184,1016	10,05	0	0	1,07	10,05	7			36962,13	682648,75	7			1	8	Carbamid	73302,11	Choline transporter-like protein 1 OS=Homo sapiens OX=9606 GN=SLC44A1 PE=1 SV=1
8	Q9H583|HEA	111,9244	0,28	0	0	0	0,28	1				3680,5205	1				1		242370,23	HEAT repeat-containing protein 1 OS=Homo sapiens OX=9606 GN=HEATR1 PE=1 SV=3
1075	Q8IY17|PLPL	23,1155	0,44	0	0,44	0	0	1					1		1				150953,89	Patatin-like phospholipase domain-containing protein 6 OS=Homo sapiens OX=9606 GN=PNPLA6 PE=1 SV=3
808	Q9C093|SPEF	50,81756	0,66	0	0	0,33	0,33	2			100671,16	104995,77	2			1	1	Acetylatic	209810,83	Sperm flagellar protein 2 OS=Homo sapiens OX=9606 GN=SPEF2 PE=1 SV=2
580	P51153|RAB1	120,1895	13,3	0	0	3,45	13,3	3				22392,434	1			1	3		22774,115	Ras-related protein Rab-13 OS=Homo sapiens OX=9606 GN=RAB13 PE=1 SV=1
385	P38646|GRP7	213,4168	4,27	1,03	0	3,39	2,06	5			73782,05		2	1		4	4	Deamidat	73680,49	Stress-70 protein, mitochondrial OS=Homo sapiens OX=9606 GN=HSPA9 PE=1 SV=2
880	Q9NYN1|RAS	45,71551	2,26	2,26	0	0	0	1	1249522,2				1	2					29661,977	Ras-like protein family member 12 OS=Homo sapiens OX=9606 GN=RASL12 PE=1 SV=1
105	Q5SW79|CE1	66,29748	0,38	0,38	0,38	0	0,38	1	132969,38	238432,69		43754,15	1	1	3		1		175292,98	Centrosomal protein of 170 kDa OS=Homo sapiens OX=9606 GN=CEP170 PE=1 SV=1
1165	Q92841|DDX	26,4375	0,82	0	0,82	0	0	1					1		1				80272,44	Probable ATP-dependent RNA helicase DDX17 OS=Homo sapiens OX=9606 GN=DDX17 PE=1 SV=2
809	O43182|RHG	101,3617	1,23	0,62	1,23	0,62	0,62	2		222599,11			1	1	3	1	1		105947,484	Rho GTPase-activating protein 6 OS=Homo sapiens OX=9606 GN=ARHGAP6 PE=1 SV=3
190	Q01082|SPTE	41,12157	0,25	0,25	0	0	0	1					1	1					274609,22	Spectrin beta chain, non-erythrocytic 1 OS=Homo sapiens OX=9606 GN=SPTBN1 PE=1 SV=2
810	Q9HCL0|PCD	53,42294	2,03	0	0	0	2,03	2				61113,285	2				2	Deamidat	126149,18	Protocadherin-18 OS=Homo sapiens OX=9606 GN=PCDH18 PE=1 SV=3
137	P05023|AT1A	397,6642	19,26	0	0	4,2	19,26	19			262884,8	1447413,9	17			5	20	Carbamid	112896,09	Sodium/potassium-transporting ATPase subunit alpha-1 OS=Homo sapiens OX=9606 GN=ATP1A1 PE=1 SV=1
39	Q7Z3B4|NUP	45,5897	1,18	0	0	0	1,18	1				255148,89	1				1		55435,453	Nucleoporin p54 OS=Homo sapiens OX=9606 GN=NUP54 PE=1 SV=2
811	P28072|PSB6	150,5434	8,37	0	3,77	8,37	8,37	2		20966,059	99574,67	81121,74	2		1	2	2		25357,715	Proteasome subunit beta type-6 OS=Homo sapiens OX=9606 GN=PSMB6 PE=1 SV=4
617	Q6MZP7|LIN	83,19873	0,8	0	0	0,8	0	1					1			1			79494,21	Protein lin-54 homolog OS=Homo sapiens OX=9606 GN=LIN54 PE=1 SV=3
812	P40121|CAP	133,8559	6,61	6,61	0	0	0	2					2	2					38498,535	Macrophage-capping protein OS=Homo sapiens OX=9606 GN=CAPG PE=1 SV=2
813	Q9Y485|DMX	94,73164	0,43	0,2	0,2	0,23	0,43	2	154735,44				1	1	2	1	2		337838,66	DmX-like protein 1 OS=Homo sapiens OX=9606 GN=DMXL1 PE=1 SV=3
617	Q9Y4I5|MTL5	83,19873	1,18	0	0	1,18	0	1					1			1			55024,88	Tesmin OS=Homo sapiens OX=9606 GN=TESMIN PE=1 SV=2
815	Q12959|DLG1	49,8433	1,33	0,66	0	0,66	0	2					1	1		1			100455,016	Disks large homolog 1 OS=Homo sapiens OX=9606 GN=DLG1 PE=1 SV=2
816	Q14690|RRP	46,77705	0,64	0	0,32	0,32	0	2		970712,75			1		1	1			208700,78	Protein RRP5 homolog OS=Homo sapiens OX=9606 GN=PDCD11 PE=1 SV=3
1079	O60245|PCD	24,10124	0,56	0	0	0,56	0	1			223339,5		1			1		Deamidat	116070,93	Protocadherin-7 OS=Homo sapiens OX=9606 GN=PCDH7 PE=1 SV=2
197	Q9BRK5|CAB	339,2736	25,41	25,41	14,36	0	0	12	1489224,1	213278,39			11	19	8			Deamidat	41806,785	45 kDa calcium-binding protein OS=Homo sapiens OX=9606 GN=SDF4 PE=1 SV=1
664	Q52LA3|LIN5	34,54774	5,17	0	5,17	0	0	1		34864,406			1		1				13000,921	Protein lin-52 homolog OS=Homo sapiens OX=9606 GN=LIN52 PE=1 SV=1
1166	P46781|RS9_	28,35323	3,09	0	0	0	3,09	1				49577,066	1				1		22591,426	40S ribosomal protein S9 OS=Homo sapiens OX=9606 GN=RPS9 PE=1 SV=3
1167	O60391|NMD	41,05785	0,58	0	0,58	0	0	1					1		1				112992,33	Glutamate receptor ionotropic, NMDA 3B OS=Homo sapiens OX=9606 GN=GRIN3B PE=2 SV=2
428	P06748|NPM	158,1223	12,93	9,86	0	0	3,06	5	607523,3			73619,41	5	6			1		32575,02	Nucleophosmin OS=Homo sapiens OX=9606 GN=NPM1 PE=1 SV=2
386	P09603|CSF1	212,4426	11,91	10,29	5,6	0	0	6	279709,62	189466,06			6	5	4			Carbamid	60179,46	Macrophage colony-stimulating factor 1 OS=Homo sapiens OX=9606 GN=CSF1 PE=1 SV=2
252	P33778|H2B1	299,3754	59,52	34,13	20,63	29,37	59,52	9	117890,07	89813,125	87343,49	987559,8	1	7	6	7	15	Oxidation	13950,1875	Histone H2B type 1-B OS=Homo sapiens OX=9606 GN=H2BC3 PE=1 SV=2
1168	P40425|PBX2	28,99176	1,63	1,63	0	0	0	1					1	2					45881,285	Pre-B-cell leukemia transcription factor 2 OS=Homo sapiens OX=9606 GN=PBX2 PE=1 SV=2
793	Q14406|CSHI	33,14592	3,15	0	0	0	3,15	1					1				1		25390,83	Chorionic somatomammotropin hormone-like 1 OS=Homo sapiens OX=9606 GN=CSHL1 PE=2 SV=2
70	Q9HBD1|RC3	36,49049	0,5	0,5	0	0	0,5	1	10272,887			17890,227	1	1			1		131668,56	Roquin-2 OS=Homo sapiens OX=9606 GN=RC3H2 PE=1 SV=2
1169	P09496|CLCA	27,15515	2,82	2,82	0	0	0	1	39860,207				1	1					27076,69	Clathrin light chain A OS=Homo sapiens OX=9606 GN=CLTA PE=1 SV=1
77	Q14938|NFIX	47,90091	1,2	1,2	0	0	0	1					1	1					55098,234	Nuclear factor 1 X-type OS=Homo sapiens OX=9606 GN=NFIX PE=1 SV=2
264	P27816|MAP	313,3323	8,16	8,16	0	0	0	10	571416,8				9	11					121004,98	Microtubule-associated protein 4 OS=Homo sapiens OX=9606 GN=MAP4 PE=1 SV=3
1099	Q13936|CAC	28,66452	0,27	0	0	0	0,27	1				25210,613	1				1		248976,61	Voltage-dependent L-type calcium channel subunit alpha-1C OS=Homo sapiens OX=9606 GN=CACNA1C PE=1 SV=4
152	Q96IF1|AJUB	22,80711	1,12	0	1,12	0	0	1		18913,602			1		1				56933,973	LIM domain-containing protein ajuba OS=Homo sapiens OX=9606 GN=AJUBA PE=1 SV=1
1121	Q9Y2D5|AKA	34,83939	0,7	0	0	0	0,7	1				131281,55	1				1		94660,53	A-kinase anchor protein 2 OS=Homo sapiens OX=9606 GN=AKAP2 PE=1 SV=3
159	O75027|ABC	48,53948	0,8	0	0	0,8	0,8	1			22217,67	92420,484	1			1	1		82641,305	Iron-sulfur clusters transporter ABCB7, mitochondrial OS=Homo sapiens OX=9606 GN=ABCB7 PE=1 SV=2
634	Q9H5I1|SUV	71,26441	1,46	0	1,46	1,46	1,46	1		4961949	219925,28	3924922	1		11	8	1	Deamidat	46682,38	Histone-lysine N-methyltransferase SUV39H2 OS=Homo sapiens OX=9606 GN=SUV39H2 PE=1 SV=2
593	P35326|SPR2	180,0902	30,56	0	0	30,56	30,56	2			894421,7	1087446,5	2			5	5	Carbamid	7965,389	Small proline-rich protein 2A OS=Homo sapiens OX=9606 GN=SPRR2A PE=1 SV=1
110	P52789|HXK2	94,40527	0,87	0	0	0,87	0	1			19519,834		1			1			102379,8	Hexokinase-2 OS=Homo sapiens OX=9606 GN=HK2 PE=1 SV=2
236	P19338|NUC	240,4006	11,69	11,69	0,85	0,85	0,85	11	717125,4				10	15	1	2	2		76614,41	Nucleolin OS=Homo sapiens OX=9606 GN=NCL PE=1 SV=3
476	P13497|BMP	183,1423	3,96	3,96	0	0	0	4	37147,094				4	4					111248,695	Bone morphogenetic protein 1 OS=Homo sapiens OX=9606 GN=BMP1 PE=1 SV=2
1030	P54725|RD23	35,93669	2,48	2,48	0	0	0	1	108066,305				1	1					39609,2	UV excision repair protein RAD23 homolog A OS=Homo sapiens OX=9606 GN=RAD23A PE=1 SV=1
822	Q96FE7|P3IP	148,2215	6,84	6,84	4,18	0	0	2	178451,34	127470,58			2	2	1				28247,996	Phosphoinositide-3-kinase-interacting protein 1 OS=Homo sapiens OX=9606 GN=PIK3IP1 PE=1 SV=2
1170	P24534|EF1B	33,16039	3,11	3,11	0	0	0	1	63333,95				1	1					24763,725	Elongation factor 1-beta OS=Homo sapiens OX=9606 GN=EEF1B2 PE=1 SV=3
823	P00746|CFAD	36,544	11,07	8,7	2,37	0	0	2		140092,28			2	1	1			Carbamid	27032,928	Complement factor D OS=Homo sapiens OX=9606 GN=CFD PE=1 SV=5
1032	Q8TE60|ATS1	47,494	0,49	0,49	0,49	0	0	1	304970,78	254798,3			1	1	2				135167,12	A disintegrin and metalloproteinase with thrombospondin motifs 18 OS=Homo sapiens OX=9606 GN=ADAMTS18 PE=1 SV=3
97	P06733|ENO	434,3361	49,31	35,71	38,02	42,86	36,87	26	3221182,5	3593579	3331645,8	3253053,2	16	29	49	37	22	Deamidat	47168,957	Alpha-enolase OS=Homo sapiens OX=9606 GN=ENO1 PE=1 SV=2
283	A6NCN2|KR8	174,5054	7,06	0	0	0	7,06	2				202477,55	2				2	Carbamid	29117,041	Putative keratin-87 protein OS=Homo sapiens OX=9606 GN=KRT87P PE=5 SV=4
1171	P62851|RS25	51,29034	8	0	0	0	8	1				29273,486	1				1		13742,126	40S ribosomal protein S25 OS=Homo sapiens OX=9606 GN=RPS25 PE=1 SV=1
508	Q0IIM8|TBC8	42,00377	0,54	0	0	0	0,54	1				84432,63	1				1		128708,92	TBC1 domain family member 8B OS=Homo sapiens OX=9606 GN=TBC1D8B PE=1 SV=2
147	Q07092|COG	320,8289	8,17	6,3	4,61	1,87	1,12	18	979682	390414,44	60260,66		7	16	12	6	3	Deamidat	157751,38	Collagen alpha-1(XVI) chain OS=Homo sapiens OX=9606 GN=COL16A1 PE=1 SV=2
93	Q3SXM0|DC4	96,03992	1,52	1,52	1,52	1,52	1,52	1			42627,023	262097,48	1	1	1	1	1		44263,84	DDB1- and CUL4-associated factor 4-like protein 1 OS=Homo sapiens OX=9606 GN=DCAF4L1 PE=2 SV=1
286	O43854|EDIL	310,0782	17,71	0	0	17,71	0	8			746685,9		8			18		Carbamid	53764,863	EGF-like repeat and discoidin I-like domain-containing protein 3 OS=Homo sapiens OX=9606 GN=EDIL3 PE=1 SV=1
824	Q8TCT8|SPP	57,45968	4,04	0	0	0	4,04	2				57199,51	2				2	Carbamid	58143,152	Signal peptide peptidase-like 2A OS=Homo sapiens OX=9606 GN=SPPL2A PE=1 SV=2
1172	Q6L8Q7|PDE	29,77078	0,99	0	0	0,99	0	1			251203,05		1			1			67351,52	2',5'-phosphodiesterase 12 OS=Homo sapiens OX=9606 GN=PDE12 PE=1 SV=2
189	P02743|SAM	89,06924	2,69	2,69	2,69	0	0	1	200723,94				1	1	1				25387,125	Serum amyloid P-component OS=Homo sapiens OX=9606 GN=APCS PE=1 SV=2
639	Q70SY1|CR3L	38,73826	1,35	0	0	0	1,35	1				39475,867	1				1		57414,863	Cyclic AMP-responsive element-binding protein 3-like protein 2 OS=Homo sapiens OX=9606 GN=CREB3L2 PE=1 SV=3
301	P50552|VASP	68,35734	1,58	0	0	0	1,58	1					1				1		39829,723	Vasodilator-stimulated phosphoprotein OS=Homo sapiens OX=9606 GN=VASP PE=1 SV=3
825	Q14684|RRP1	40,57384	1,58	0,79	0,79	0	0	2	997072,7				1	3	1				84427,52	Ribosomal RNA processing protein 1 homolog B OS=Homo sapiens OX=9606 GN=RRP1B PE=1 SV=3
328	P11168|GTR2	38,18213	1,15	1,15	1,15	0	0	1	517809,8				1	1	1				57489,523	Solute carrier family 2, facilitated glucose transporter member 2 OS=Homo sapiens OX=9606 GN=SLC2A2 PE=1 SV=1
715	Q68CJ6|SLIP_	31,58539	0,75	0	0	0	0,75	1					1				1		91131,84	Nuclear GTPase SLIP-GC OS=Homo sapiens OX=9606 GN=NUGGC PE=2 SV=3
447	P22492|H1T_	164,0111	9,66	0	0	0	9,66	2				57619,617	1				2		22018,994	Histone H1t OS=Homo sapiens OX=9606 GN=H1-6 PE=2 SV=4
6	P29728|OAS	35,55188	0,83	0	0	0,83	0	1			13169,212		1			1			82430,62	2'-5'-oligoadenylate synthase 2 OS=Homo sapiens OX=9606 GN=OAS2 PE=1 SV=3
1173	Q8N398|VW	25,27597	0,48	0	0,48	0	0	1		21755,324			1		1				131667,75	von Willebrand factor A domain-containing protein 5B2 OS=Homo sapiens OX=9606 GN=VWA5B2 PE=2 SV=3
590	P80098|CCL7	96,79324	6,06	6,06	0	0	0	1	506907,44				1	3					11200,186	C-C motif chemokine 7 OS=Homo sapiens OX=9606 GN=CCL7 PE=1 SV=3
827	Q8WUJ3|CEN	48,88244	1,03	0	0	0	1,03	2				46769,684	2				2	Carbamid	152997,77	Cell migration-inducing and hyaluronan-binding protein OS=Homo sapiens OX=9606 GN=CEMIP PE=1 SV=2
6	O75151|PHF	49,33768	0,55	0,55	0,55	0	0	1	258931,9	88723,61			1	1	2				120774,99	Lysine-specific demethylase PHF2 OS=Homo sapiens OX=9606 GN=PHF2 PE=1 SV=4
71	O94759|TRP	96,1655	0,47	0	0	0,47	0	1			116862,125		1			1		Carbamid	171198,23	Transient receptor potential cation channel subfamily M member 2 OS=Homo sapiens OX=9606 GN=TRPM2 PE=1 SV=2
428	Q9C0A0|CNT	70,07965	0,54	0,54	0	0	0	1	250109,83				1	2					145274,39	Contactin-associated protein-like 4 OS=Homo sapiens OX=9606 GN=CNTNAP4 PE=1 SV=3
994	A0A087WW8	36,45289	5,79	5,79	0	0	0	1	92659,414				1	1					13310,112	Immunoglobulin kappa variable 2-40 OS=Homo sapiens OX=9606 GN=IGKV2-40 PE=3 SV=2
1027	P22695|QCR	35,86885	1,32	1,32	0	0	0	1					1	1					48443,004	Cytochrome b-c1 complex subunit 2, mitochondrial OS=Homo sapiens OX=9606 GN=UQCRC2 PE=1 SV=3
94	Q5SYC1|CLV	88,54646	1,83	0	0	1,83	1,83	1					1			2	2		37999,547	Clavesin-2 OS=Homo sapiens OX=9606 GN=CLVS2 PE=1 SV=1
1174	Q8TAD7|OCC	68,90275	19,05	0	0	0	19,05	1				28029,188	1				1		6406,9893	Overexpressed in colon carcinoma 1 protein OS=Homo sapiens OX=9606 GN=OCC1 PE=1 SV=2
78	Q8N264|RHG	80,14496	0,8	0,8	0,8	0	0	1					1	1	1				84258,266	Rho GTPase-activating protein 24 OS=Homo sapiens OX=9606 GN=ARHGAP24 PE=1 SV=2
739	Q6ZW76|AN	84,83708	1,07	0,91	0,91	0,91	1,07	2				938182,56	1	3	3	8	4		72038,04	Ankyrin repeat and SAM domain-containing protein 3 OS=Homo sapiens OX=9606 GN=ANKS3 PE=1 SV=1
121	P04083|ANX	415,8823	54,62	10,69	0	48,55	48,84	20	26053,703		4045497	5640190	20	3		35	29	Acetylatic	38714,242	Annexin A1 OS=Homo sapiens OX=9606 GN=ANXA1 PE=1 SV=2
159	P21589|5NTD	400,1183	27,7	0	0	18,47	27,7	17			1123451,8	3233327,5	17			13	30	Carbamid	63367,742	5'-nucleotidase OS=Homo sapiens OX=9606 GN=NT5E PE=1 SV=1
94	Q68DK2|ZFY	88,54646	0,24	0	0	0,24	0,24	1					1			2	2		284576,06	Zinc finger FYVE domain-containing protein 26 OS=Homo sapiens OX=9606 GN=ZFYVE26 PE=1 SV=3
61	O95568|MET	100,1197	1,88	1,88	1,88	0	0	1					1	1	1				42148,117	Histidine protein methyltransferase 1 homolog OS=Homo sapiens OX=9606 GN=METTL18 PE=1 SV=1

189	O95936|EOM	103,1225	0,87	0,87	0,87	0	0,87	1	79695,99	28457,154			1	1	1		1		72731,91	Eomesodermin homolog OS=Homo sapiens OX=9606 GN=EOMES PE=1 SV=3
349	Q9NQC3|RTN	255,7858	7,13	0	0	7,13	5,96	5			1236593,6	702021,7	4			10	5	Deamidat	129931,414	Reticulon-4 OS=Homo sapiens OX=9606 GN=RTN4 PE=1 SV=2
1175	O75954|TSN	34,08744	2,51	0	0	0	2,51	1					1				1	Carbamid	26778,916	Tetraspanin-9 OS=Homo sapiens OX=9606 GN=TSPAN9 PE=1 SV=1
830	P00813|ADA_	102,8834	3,86	3,86	1,65	0	0	2					1	2	1				40764,445	Adenosine deaminase OS=Homo sapiens OX=9606 GN=ADA PE=1 SV=3
66	Q13163|MP2	110,3485	1,34	0	0	1,34	1,34	1			1379749,9	1665554,9	1			3	4		50111,727	Dual specificity mitogen-activated protein kinase kinase 5 OS=Homo sapiens OX=9606 GN=MAP2K5 PE=1 SV=2
706	B7ZW38|HNF	43,84666	3,41	3,41	0	0	0	1	79983,87				1	2					32029,445	Heterogeneous nuclear ribonucleoprotein C-like 3 OS=Homo sapiens OX=9606 GN=HNRNPCL3 PE=2 SV=1
350	P09104|ENO	241,7096	12,9	6,91	5,99	9,68	6,91	7			45709,625		1	4	6	8	4	Carbamid	47268,574	Gamma-enolase OS=Homo sapiens OX=9606 GN=ENO2 PE=1 SV=3
1	Q969V6|MRT	42,41132	0,64	0	0	0	0,64	1				208673,22	1				1		98918,98	Myocardin-related transcription factor A OS=Homo sapiens OX=9606 GN=MRTFA PE=1 SV=1
583	Q99497|PARI	60,87629	25,4	25,4	0	0	0	3	79745,91				3	4				Carbamid	19891,047	Parkinson disease protein 7 OS=Homo sapiens OX=9606 GN=PARK7 PE=1 SV=2
1032	Q8N2I2|ZN6	147,494	1,07	1,07	1,07	0	0	1	304970,78	254798,3			1	1	2				63321,66	Zinc finger protein 619 OS=Homo sapiens OX=9606 GN=ZNF619 PE=2 SV=1
593	Q96RM1|SPR	119,4008	12,5	0	0	12,5	12,5	1			349350,56	524792,75	1			2	2	Carbamid	7805,25	Small proline-rich protein 2F OS=Homo sapiens OX=9606 GN=SPRR2F PE=3 SV=1
1176	O43572|AKA	34,12614	0,91	0,91	0,91	0	0,91	1	120189,29	65262,574		2043663,5	1	1	1		1		73818,18	A-kinase anchor protein 10, mitochondrial OS=Homo sapiens OX=9606 GN=AKAP10 PE=1 SV=2
315	P04075|ALD	302,1402	25,55	16,21	0	22,25	7,69	8	329287,94		576949,1	76915,71	7	6		9	4	Carbamid	39420,01	Fructose-bisphosphate aldolase A OS=Homo sapiens OX=9606 GN=ALDOA PE=1 SV=2
1075	Q96A29|FUC	23,1155	1,65	0	1,65	0	0	1					1		1				39808,836	GDP-fucose transporter 1 OS=Homo sapiens OX=9606 GN=SLC35C1 PE=1 SV=1
98	Q9NZM1|MY	449,284	12,86	0	0	3,83	12,28	27			164374,48	9,68E+07	22			9	34		234708,72	Myoferlin OS=Homo sapiens OX=9606 GN=MYOF PE=1 SV=1
1177	Q8WY41|NAI	42,31192	2,05	2,05	0	2,05	0	1	115653,875		424286,38		1	1		2			30230,205	Nanos homolog 1 OS=Homo sapiens OX=9606 GN=NANOS1 PE=1 SV=2
148	Q8WWN9|IC	42,83809	1,37	1,37	0	0	0	1	70875,31				1	1					48992,96	Interactor protein for cytohesin exchange factors 1 OS=Homo sapiens OX=9606 GN=IPCEF1 PE=1 SV=1
6	Q96N76|HUT	76,21806	0,89	0,89	0	0,89	0,89	1	95524,04		1177229,9	711697,1	1	1		2	1		74830,53	Urocanate hydratase OS=Homo sapiens OX=9606 GN=UROC1 PE=1 SV=1
1173	O75882|ATRI	25,27597	0,42	0	0,42	0	0	1		21755,324			1		1				158536,88	Attractin OS=Homo sapiens OX=9606 GN=ATRN PE=1 SV=2
1178	Q96PL5|ERM	53,57428	1,26	0	1,26	0	0	1		28655			1		1				52604,746	Erythroid membrane-associated protein OS=Homo sapiens OX=9606 GN=ERMAP PE=1 SV=1
387	P04792|HSPE	271,9281	30,24	22,44	0	30,24	17,07	5	350240,5		1059722,9	347369,7	5	5		8	4	Deamidat	22782,52	Heat shock protein beta-1 OS=Homo sapiens OX=9606 GN=HSPB1 PE=1 SV=2
52	Q08431|MFG	470,3384	67,44	7,49	0	67,44	43,67	33	82165,56		7,70E+07	1,67E+07	32	4		162	53	Carbamid	43104,95	Lactadherin OS=Homo sapiens OX=9606 GN=MFGE8 PE=1 SV=3
584	P08F94|PKH	95,3026	0,44	0	0	0,29	0,44	3				52988,613	1			2	3		446701,72	Fibrocystin OS=Homo sapiens OX=9606 GN=PKHD1 PE=1 SV=1
316	P27482|CALL	173,7484	10,07	10,07	10,07	10,07	10,07	2	3080727	1182869,6	1315807,8	1101725	2	8	6	5	6	Oxidation	16890,705	Calmodulin-like protein 3 OS=Homo sapiens OX=9606 GN=CALML3 PE=1 SV=2
128	Q08188|TGM	405,0927	24,96	0	0	20,63	16,02	21			1286697,5	1129873,9	21			18	12	Carbamid	76631,83	Protein-glutamine gamma-glutamyltransferase E OS=Homo sapiens OX=9606 GN=TGM3 PE=1 SV=4
283	Q9NSB4|KRT	40,32876	1,17	0	0	1,17	0	1			115794,38		1			1			56652,69	Keratin, type II cuticular Hb2 OS=Homo sapiens OX=9606 GN=KRT82 PE=1 SV=3
904	Q9Y5L3|ENT	53,43855	1,21	0	0	1,21	0	1			489244,44		1			1			53665,473	Ectonucleoside triphosphate diphosphohydrolase 2 OS=Homo sapiens OX=9606 GN=ENTPD2 PE=1 SV=1
36	Q86W24|NAI	81,35167	0,55	0,55	0,55	0,55	0,55	1					1	1	1	1	1		124733,086	NACHT, LRR and PYD domains-containing protein 14 OS=Homo sapiens OX=9606 GN=NLRP14 PE=1 SV=1
243	P10768|ESTD	80,71943	2,13	2,13	0	0	0	1	337568,3				1	1					31462,785	S-formylglutathione hydrolase OS=Homo sapiens OX=9606 GN=ESD PE=1 SV=2
1010	P48382|RFX5	32,29033	0,97	0	0	0,97	0	1			22121,885		1			2			65322,992	DNA-binding protein RFX5 OS=Homo sapiens OX=9606 GN=RFX5 PE=1 SV=1
1011	B4DH59|NBP	41,75044	0,67	0	0	0,67	0,67	1			5235523	7,28E+07	1			2	4		103816,46	Neuroblastoma breakpoint family member 26 OS=Homo sapiens OX=9606 GN=NBPF26 PE=5 SV=1
6	Q9NSE4|SYI	76,21806	0,59	0,59	0	0,59	0,59	1	95524,04		1177229,9	711697,1	1	1		2	1		113791,516	Isoleucine--tRNA ligase, mitochondrial OS=Homo sapiens OX=9606 GN=IARS2 PE=1 SV=2
27	A6NGG8|PCA	81,58185	0,47	0,47	0,47	0,47	0,47	1	126414,664	122217,4	29872,697	68153,32	1	1	1	1	1		139655,33	Photoreceptor cilium actin regulator OS=Homo sapiens OX=9606 GN=PCARE PE=1 SV=1
1179	Q9P107|GMI	37,06379	0,62	0,62	0	0	0	1	7278510,5				1	3					106682,9	GEM-interacting protein OS=Homo sapiens OX=9606 GN=GMIP PE=1 SV=2
1180	Q9BW92|SYT	24,63669	0,84	0	0	0,84	0	1			56772,37		1			1			81036,12	Threonine--tRNA ligase, mitochondrial OS=Homo sapiens OX=9606 GN=TARS2 PE=1 SV=1
1181	P46776|RL27	36,05008	7,43	0	0	0	7,43	1				56733,594	1				1		16561,38	60S ribosomal protein L27a OS=Homo sapiens OX=9606 GN=RPL27A PE=1 SV=2
388	Q8IZC6|COR	247,1888	2,47	1,67	1,45	0,65	0,32	6	1430438,8	1402827,2			3	5	5	3	1		186892,34	Collagen alpha-1(XXVII) chain OS=Homo sapiens OX=9606 GN=COL27A1 PE=1 SV=1
1182	Q13428|TCO	29,53123	0,4	0	0,4	0	0	1		133269,19			1		1				152106,03	Treacle protein OS=Homo sapiens OX=9606 GN=TCOF1 PE=1 SV=3
99	Q9UNW8|GP	78,47889	1,58	1,58	1,58	0	0	1	59342,84	35403,457			1	1	1				42498,99	Probable G-protein coupled receptor 132 OS=Homo sapiens OX=9606 GN=GPR132 PE=1 SV=1
65	Q709C8|VP1	117,2209	0,19	0,19	0,19	0	0,19	2	3223471,5	1891425			2	5	6		1		422390,1	Intermembrane lipid transfer protein VPS13C OS=Homo sapiens OX=9606 GN=VPS13C PE=1 SV=1
217	P14314|GLU2	351,7181	22,35	22,35	9,85	0	0	12	1441400,8	224460,7			11	18	7				59425,414	Glucosidase 2 subunit beta OS=Homo sapiens OX=9606 GN=PRKCSH PE=1 SV=2
129	P05121|PAI1_	394,23	42,54	42,54	24,38	0	2,49	17	3,40E+07	7974661		37639,99	16	84	38		1	Oxidation	45059,977	Plasminogen activator inhibitor 1 OS=Homo sapiens OX=9606 GN=SERPINE1 PE=1 SV=1
398	Q9UGJ0|AAK	32,09123	1,05	1,05	1,05	0	0	1	887625,8	83099,29			1	1	1				63066,21	5'-AMP-activated protein kinase subunit gamma-2 OS=Homo sapiens OX=9606 GN=PRKAG2 PE=1 SV=1
1183	Q96TA1|NIB	25,73077	0,8	0	0	0,8	0	1					1			1			84137,86	Protein Niban 2 OS=Homo sapiens OX=9606 GN=NIBAN2 PE=1 SV=3
77	Q12857|NFIA	47,90091	1,18	1,18	0	0	0	1					1	1					55944,164	Nuclear factor 1 A-type OS=Homo sapiens OX=9606 GN=NFIA PE=1 SV=2
351	P02765|FETU	294,8287	8,72	8,72	8,72	6,81	5,18	7	7,44E+07	8278939,5	474220	336331,75	7	45	25	4	3	Carbamid	39340,734	Alpha-2-HS-glycoprotein OS=Homo sapiens OX=9606 GN=AHSG PE=1 SV=2
316	P0DP24|CAL	295,4051	41,61	38,93	30,87	33,56	30,87	7	5591565,5	2249549,2	2484052,8	2222430,5	7	13	11	10	10	Acetylatic	16837,582	Calmodulin-2 OS=Homo sapiens OX=9606 GN=CALM2 PE=1 SV=1
9	P35908|K22E	613,31	83,1	47,26	56,34	82	77,46	119	3508031,8	4580794,5	5,05E+08	3,17E+08	81	61	100	632	390	Deamidat	65432,883	Keratin, type II cytoskeletal 2 epidermal OS=Homo sapiens OX=9606 GN=KRT2 PE=1 SV=2
53	P02768|ALBU	481,7393	51,56	26,27	14,94	42,2	33,17	39	3,73E+08	1,79E+07	1,28E+07	1,32E+07	38	141	47	54	39	Carbamid	69366,68	Albumin OS=Homo sapiens OX=9606 GN=ALB PE=1 SV=2
1157	Q96MV8|ZDH	42,52576	1,78	0	0	1,78	0	1			129776,97		1			1			39330,797	Palmitoyltransferase ZDHHC15 OS=Homo sapiens OX=9606 GN=ZDHHC15 PE=1 SV=1
1184	Q9Y2U5|M3K	70,34459	0,97	0	0	0,97	0	1			22210,875		1			1			69740,86	Mitogen-activated protein kinase kinase kinase 2 OS=Homo sapiens OX=9606 GN=MAP3K2 PE=1 SV=2
775	Q8IZW8|TEN	76,97985	0,84	0,84	0	0	0	1	21360,566				1	1					76763,77	Tensin-4 OS=Homo sapiens OX=9606 GN=TNS4 PE=1 SV=3
188	Q9NRN5|OLF	352,6106	28,33	25,12	17	14,04	17,73	14	3588732,5	1299133,8	166363,72	2159783	12	19	11	7	15	Carbamid	46010,25	Olfactomedin-like protein 3 OS=Homo sapiens OX=9606 GN=OLFML3 PE=2 SV=1
1185	O14795|UN1	50,3294	0,38	0	0,38	0	0	1		33754,23			1		1				180678,67	Protein unc-13 homolog B OS=Homo sapiens OX=9606 GN=UNC13B PE=1 SV=2
110	Q6KB66|K2C	426,6464	45,35	0	2,43	40,27	34,29	24			3283736,8	1843960,8	20		1	38	21	Deamidat	50525,3	Keratin, type II cytoskeletal 80 OS=Homo sapiens OX=9606 GN=KRT80 PE=1 SV=2
389	Q9NZT1|CAL	220,6271	30,82	0	8,9	30,82	30,82	4		74453,32	2312138,5	6519071,5	4		1	9	13	Acetylatio	15892,504	Calmodulin-like protein 5 OS=Homo sapiens OX=9606 GN=CALML5 PE=1 SV=2
430	Q86TL0|ATG	29,12412	1,27	0	0	0	1,27	1				41747,074	1				1		52922,363	Cysteine protease ATG4D OS=Homo sapiens OX=9606 GN=ATG4D PE=1 SV=1
51	Q96GK7|FAH	97,32061	1,91	0	0	0	1,91	1				69994,57	1				2		34596,22	Fumarylacetoacetate hydrolase domain-containing protein 2A OS=Homo sapiens OX=9606 GN=FAHD2A PE=1 SV=1
1011	Q8N660|NBP	41,75044	0,9	0	0	0,9	0,9	1			5235523	7,28E+07	1			2	4		77563,016	Neuroblastoma breakpoint family member 15 OS=Homo sapiens OX=9606 GN=NBPF15 PE=2 SV=2
515	O95785|WIZ_	26,32922	0,36	0,36	0	0	0	1					1	1					178674,19	Protein Wiz OS=Homo sapiens OX=9606 GN=WIZ PE=1 SV=2
182	P07437|TBB5	340,7442	29,28	0	0	26,58	14,19	12			2232866	237106,28	12			16	7	Oxidation	49670,812	Tubulin beta chain OS=Homo sapiens OX=9606 GN=TUBB PE=1 SV=2
1044	Q6ZN92|DUT	40,36036	4,26	0	0	4,26	0	1			63496,605		1			1			15492,056	Putative inactive deoxyuridine 5'-triphosphate nucleotidohydrolase-like protein FLJ16323 OS=Homo sapiens OX=9606 PE=5 SV=1
561	O60500|NPH	43,05391	0,48	0	0,48	0	0	1					1		1				134742,19	Nephrin OS=Homo sapiens OX=9606 GN=NPHS1 PE=1 SV=1
429	Q8N2Y8|RUS	150,8348	1,72	0,46	0,92	0,86	0,86	5	489938,22	568887,4	84891,89	314077,28	4	1	2	3	2		161225,34	AP-4 complex accessory subunit RUSC2 OS=Homo sapiens OX=9606 GN=RUSC2 PE=1 SV=3
177	Q5VTE0|EF1	369,0201	28,79	8,23	0	28,79	14,72	15	528966,6		3656404	2602554,5	15	6		25	12		50185,016	Putative elongation factor 1-alpha-like 3 OS=Homo sapiens OX=9606 GN=EEF1A1P5 PE=5 SV=1
390	P62805|H4_H	252,231	46,6	36,89	34,95	27,18	27,18	5	655130,75	481536,25	439196,72	1977760,6	5	6	10	3	8	Oxidation	11367,34	Histone H4 OS=Homo sapiens OX=9606 GN=H4C1 PE=1 SV=2
2	Q9UK17|KCN	62,11605	0,92	0	0	0,92	0,92	1			87982,59	110194,64	1			1	2		73451,32	Potassium voltage-gated channel subfamily D member 3 OS=Homo sapiens OX=9606 GN=KCND3 PE=1 SV=3
838	Q9P2V4|LRIT	75,18695	1,93	0,96	0	0,96	0	2	451087,34				1	1		1			68040,53	Leucine-rich repeat, immunoglobulin-like domain and transmembrane domain-containing protein 1 OS=Homo sapiens OX=9606 GN=LRIT1 PE=2 SV=1
198	P68363|TBA1	355,9322	32,82	6,65	3,33	32,82	15,3	11	3913,0781		681043,4		2	3	1	15	6	Deamidat	50151,63	Tubulin alpha-1B chain OS=Homo sapiens OX=9606 GN=TUBA1B PE=1 SV=1
1186	O75635|SPB7	23,69327	2,89	0	2,89	0	0	1					1		1				42904,785	Serpin B7 OS=Homo sapiens OX=9606 GN=SERPINB7 PE=1 SV=1
38	O75165|DJC1	71,83782	0,27	0,27	0,27	0,27	0,27	1	174143,88	289360,03	61608,51	72641,82	1	8	7	1	1		254414,89	DnaJ homolog subfamily C member 13 OS=Homo sapiens OX=9606 GN=DNAJC13 PE=1 SV=5
31	P41219|PERI_	190,5541	3,83	3,83	3,83	3,83	3,83	3	3928140,8	113294,46	1144560,6	599400,3	1	8	4	4	5		53650,883	Peripherin OS=Homo sapiens OX=9606 GN=PRPH PE=1 SV=2
87	P30613|KPYR	172,5402	2,96	1,92	0	2,96	2,96	2	69033,84		510842,78	464948,9	1	1		3	3		61830,16	Pyruvate kinase PKLR OS=Homo sapiens OX=9606 GN=PKLR PE=1 SV=2
514	Q96JP9|CDH	44,08848	0,7	0	0	0	0,7	1					1				1		93594,95	Cadherin-related family member 1 OS=Homo sapiens OX=9606 GN=CDHR1 PE=1 SV=2
59	Q15063|POS	475,4238	41,03	36,36	17,82	22,97	9,45	36	4,24E+07	6873379,5	3732348,2	2712371,5	35	135	31	31	12	Carbamid	93313,945	Periostin OS=Homo sapiens OX=9606 GN=POSTN PE=1 SV=2
1147	O75132|ZBE	57,85664	0,51	0,51	0,51	0	0	1					1	1	1				130322,07	Zinc finger BED domain-containing protein 4 OS=Homo sapiens OX=9606 GN=ZBED4 PE=1 SV=2
182	Q9BVA1|TBB	303,7431	20,45	0	0	20,45	5,39	9			2161750,8	176848,97	9			13	4	Oxidation	49953,055	Tubulin beta-2B chain OS=Homo sapiens OX=9606 GN=TUBB2B PE=1 SV=1
376	P20671|H2A1	236,4661	32,31	6,92	6,92	6,92	32,31	4	230969,52	229668,45	250080,11	2471626,2	4	3	4	2	9		14107,477	Histone H2A type 1-D OS=Homo sapiens OX=9606 GN=H2AC7 PE=1 SV=2
1069	P55201|BRPF	28,22043	0,49	0	0	0	0,49	1				2043663,5	1				1		137498,75	Peregrin OS=Homo sapiens OX=9606 GN=BRPF1 PE=1 SV=2
996	Q5STR5|SIM	80,99365	7,59	7,59	7,59	7,59	7,59	1	6230665,5	2880302,8	1864814,6	5602360,5	1	1	1	1	2		8949,504	Small integral membrane protein 40 OS=Homo sapiens OX=9606 GN=SMIM40 PE=1 SV=9
646	P43353|AL3B	23,25292	1,28	0	0	0	1,28	1				65216,375	1				1		51839,7	Aldehyde dehydrogenase family 3 member B1 OS=Homo sapiens OX=9606 GN=ALDH3B1 PE=1 SV=1
692	Q8N201|INT1	29,85037	0,27	0,27	0	0	0	1	62285,676				1	1					244297,33	Integrator complex subunit 1 OS=Homo sapiens OX=9606 GN=INTS1 PE=1 SV=2
841	Q7Z406|MYH	131,7	0,65	0,65	0	0	0,3	2	120144,61				1	2			2		227870,6	Myosin-14 OS=Homo sapiens OX=9606 GN=MYH14 PE=1 SV=2
1074	Q9ULE3|DEN	29,53156	0,59	0	0	0,59	0	1			11944,6		1			1			113852,85	DENN domain-containing protein 2A OS=Homo sapiens OX=9606 GN=DENND2A PE=1 SV=4
842	Q8TEK3|DOT	58,27677	0,78	0,39	0	0,39	0	2	77703,25				1	1		2			164856,12	Histone-lysine N-methyltransferase, H3 lysine-79 specific OS=Homo sapiens OX=9606 GN=DOT1L PE=1 SV=3
328	Q99633|PRP1	65,72373	1,75	0	0	1,75	1,75	1			64781,062	89241,63	1			1	1		39859,754	Pre-mRNA-splicing factor 18 OS=Homo sapiens OX=9606 GN=PRPF18 PE=1 SV=1
23	Q5JXA9|SIRB	88,97265	1,75	0	0	1,75	1,75	1			25847,486	25132,662	1			1	1		36968,125	Signal-regulatory protein beta-2 OS=Homo sapiens OX=9606 GN=SIRPB2 PE=2 SV=1
352	P51858|HDG	295,2671	30,83	30,83	8,75	0	0	7	590276,3	59762,67			7	8	2				26788,281	Hepatoma-derived growth factor OS=Homo sapiens OX=9606 GN=HDGF PE=1 SV=1
146	Q2TAC6|KIF1	27,22116	0,6	0	0	0	0,6	1				29655,746	1				1	Deamidat	111332,44	Kinesin-like protein KIF19 OS=Homo sapiens OX=9606 GN=KIF19 PE=2 SV=2
1187	O75592|MYC	50,75201	0,13	0,13	0,13	0	0,13	1	29039,11	15819,885		72394,5	1	1	1		1		513636	E3 ubiquitin-protein ligase MYCBP2 OS=Homo sapiens OX=9606 GN=MYCBP2 PE=1 SV=4
384	Q8NG31|KNL	108,8513	0,26	0	0	0,26	0	1			1303591,6		1			2			265391,28	Kinetochore scaffold 1 OS=Homo sapiens OX=9606 GN=KNL1 PE=1 SV=3
477	Q9BTY2|FUC	188,5263	7,71	4,71	1,71	0	1,28	4	6359,7065	61757,902			3	3	1		1	Carbamid	54066,684	Plasma alpha-L-fucosidase OS=Homo sapiens OX=9606 GN=FUCA2 PE=1 SV=2
1011	Q5TI25|NBP	41,75044	0,65	0	0	0,65	0,65	1			5235523	7,28E+07	1			2	4		105852,85	Neuroblastoma breakpoint family member 14 OS=Homo sapiens OX=9606 GN=NBPF14 PE=1 SV=2
687	Q5TFQ8|SIRB	38,66667	1,51	0	1,51	0	0	1		58114,875			1		1				43359,258	Signal-regulatory protein beta-1 isoform 3 OS=Homo sapiens OX=9606 GN=SIRPB1 PE=1 SV=1
673	Q6ZR08|DYH	33,06132	0,19	0	0	0	0,19	1				1253976	1				1	Deamidat	356941,94	Dynein axonemal heavy chain 12 OS=Homo sapiens OX=9606 GN=DNAH12 PE=2 SV=2
1188	P04179|SOD	34,90708	3,15	0	3,15	0	0	1					1		1				24750,139	Superoxide dismutase [Mn], mitochondrial OS=Homo sapiens OX=9606 GN=SOD2 PE=1 SV=3
293	P42892|ECE1	61,49014	1,04	0	0	0	1,04	1					1				1	Carbamid	87163,67	Endothelin-converting enzyme 1 OS=Homo sapiens OX=9606 GN=ECE1 PE=1 SV=2
846	O15020|SPT	43,03375	0,5	0	0	0,5	0	2			600705		2			4			271324,88	Spectrin beta chain, non-erythrocytic 2 OS=Homo sapiens OX=9606 GN=SPTBN2 PE=1 SV=3
430	P12036|NFH_	194,9428	2,34	1,66	1,66	1,75	2,24	4				41747,074	1	5	7	5	5	Deamidat	112478,914	Neurofilament heavy polypeptide OS=Homo sapiens OX=9606 GN=NEFH PE=1 SV=4
111	Q86Y46|K2C7	383,614	21,85	4,26	6,48	18,89	20,56	23				28356,54	2	6	14	39	33	Deamidat	58923,344	Keratin, type II cytoskeletal 73 OS=Homo sapiens OX=9606 GN=KRT73 PE=1 SV=1
327	P01857|IGHG	292,6693	16,54	0	0	16,54	2,01	7			680663		3			14	1	Carbamid	43911,902	Immunoglobulin heavy constant gamma 1 OS=Homo sapiens OX=9606 GN=IGHG1 PE=1 SV=2

1189	O95800|GPR	36,74253	1,11	0	0	0	1,11	1				1,67E+07	1				2		59358,98	Probable G-protein coupled receptor 75 OS=Homo sapiens OX=9606 GN=GPR75 PE=1 SV=1
659	Q12907|LMA	37,40571	1,69	0	1,69	0	0	1		26360,54			1		1				40228,664	Vesicular integral-membrane protein VIP36 OS=Homo sapiens OX=9606 GN=LMAN2 PE=1 SV=1
205	Q00722|PLCB	29,26519	0,51	0,51	0	0	0	1	26584,254				1	1					134024,3	1-phosphatidylinositol 4,5-bisphosphate phosphodiesterase beta-2 OS=Homo sapiens OX=9606 GN=PLCB2 PE=1 SV=2
32	P13611|CSPG	516,0583	10,6	7,95	5,74	10,39	9,36	46	1,35E+07	1510504,2	4,28E+07	4,15E+07	45	72	37	110	104	Carbamid	372820,03	Versican core protein OS=Homo sapiens OX=9606 GN=VCAN PE=1 SV=3
54	O95678|K2C7	451,5116	29,58	12,89	13,07	26,32	26,32	33	93608,92	424013,06		656515,56	3	14	32	127	81	Oxidation	59560,39	Keratin, type II cytoskeletal 75 OS=Homo sapiens OX=9606 GN=KRT75 PE=1 SV=2
933	Q9BX68|HIN	24,05968	3,68	0	0	3,68	0	1			27898,795		1			1			17161,764	Adenosine 5'-monophosphoramidase HINT2 OS=Homo sapiens OX=9606 GN=HINT2 PE=1 SV=1
513	P0C864|DAN	27,77183	3,68	3,68	0	0	0	1	351230,7				1	1				Carbamid	16785,336	Putative uncharacterized protein DANCR OS=Homo sapiens OX=9606 GN=DANCR PE=5 SV=1
64	P24821|TENA	495,0677	19,9	1,09	3,18	0	19,9	38	70944,16	113946,02		7112815,5	37	3	9		58	Carbamid	240853,4	Tenascin OS=Homo sapiens OX=9606 GN=TNC PE=1 SV=3
1	Q9NTZ6|RBM	115,2076	0,64	0	0	0,64	0,64	1			153088,5	835240,25	1			1	2		97395,48	RNA-binding protein 12 OS=Homo sapiens OX=9606 GN=RBM12 PE=1 SV=1
266	P08572|CO4A	244,8165	4,44	3,39	1,93	1,23	0,35	10	1,69E+07	7730206,5	2400890,2		4	14	10	5	4		167553,1	Collagen alpha-2(IV) chain OS=Homo sapiens OX=9606 GN=COL4A2 PE=1 SV=4
1095	Q96HA7|TON	24,87802	0,44	0,44	0	0	0	1	328019,5				1	1					150928,58	Tonsoku-like protein OS=Homo sapiens OX=9606 GN=TONSL PE=1 SV=2
142	Q9HD23|MRS	78,41393	1,35	1,35	1,35	1,35	1,35	1				274587,2	1	1	1	1	1		50317,64	Magnesium transporter MRS2 homolog, mitochondrial OS=Homo sapiens OX=9606 GN=MRS2 PE=1 SV=1
848	O15126|SCAM	80,8754	8,58	1,78	1,78	0	6,8	2	259197,53	127323,11			2	1	1		1		37920,426	Secretory carrier-associated membrane protein 1 OS=Homo sapiens OX=9606 GN=SCAMP1 PE=1 SV=2
252	Q99877|H2B	317,3283	66,67	34,13	27,78	29,37	66,67	10	117890,07	89813,125	87343,49	987559,8	1	7	7	7	17	Oxidation	13922,134	Histone H2B type 1-N OS=Homo sapiens OX=9606 GN=H2BC15 PE=1 SV=3
849	P63241|IF5A	80,0394	9,09	3,9	0	5,19	0	2	608444,94		30442,506		2	1		1			16832,246	Eukaryotic translation initiation factor 5A-1 OS=Homo sapiens OX=9606 GN=EIF5A PE=1 SV=2
1190	Q92820|GGH	26,26204	2,52	0	2,52	0	0	1		157820,47			1		1				35964,37	Gamma-glutamyl hydrolase OS=Homo sapiens OX=9606 GN=GGH PE=1 SV=2
33	P08253|MMP	500,4625	59,85	56,82	38,94	41,97	45,3	42	1,25E+08	5,33E+07	4145079,8	8605967	38	217	144	41	54	Carbamid	73882,3	72 kDa type IV collagenase OS=Homo sapiens OX=9606 GN=MMP2 PE=1 SV=2
33	P24347|MMF	112,5079	1,43	1,43	1,43	1,43	1,43	1	3021133		22937,043	157331,23	1	5	5	1	2		54589,92	Stromelysin-3 OS=Homo sapiens OX=9606 GN=MMP11 PE=1 SV=3
1191	Q8NGI9|OR5	33,91033	2,78	0	2,78	0	0	1		110039,3			1		2				36015,74	Olfactory receptor 5A2 OS=Homo sapiens OX=9606 GN=OR5A2 PE=2 SV=1
850	P98194|AT2C	64,83399	1,31	0,65	0	0,65	0	2	2162869				2	2		1			100577,27	Calcium-transporting ATPase type 2C member 1 OS=Homo sapiens OX=9606 GN=ATP2C1 PE=1 SV=3
391	Q14254|FLOT	191,0025	11,45	0	0	11,45	4,67	6			272731,5	52113,168	5			6	2		47064,16	Flotillin-2 OS=Homo sapiens OX=9606 GN=FLOT2 PE=1 SV=2
770	A0A1W2PR1	42,8563	2,49	0	0	2,49	0	1					1			1			27941,504	Glutathione S-transferase theta-4 OS=Homo sapiens OX=9606 GN=GSTT4 PE=3 SV=1
375	P56199|ITA1_	49,7324	0,51	0	0	0,51	0	1			58505,547		1			1			130847,78	Integrin alpha-1 OS=Homo sapiens OX=9606 GN=ITGA1 PE=1 SV=2
150	Q8WYA1|BM	75,6269	1,1	1,1	1,1	0	0	1	1541438,4	606839,8			1	6	2				70887,11	Basic helix-loop-helix ARNT-like protein 2 OS=Homo sapiens OX=9606 GN=BMAL2 PE=1 SV=2
51	Q5HYK9|ZN6	64,41823	1,31	1,31	0	0	0	1	622175,9				1	1				Deamidat	70160,984	Zinc finger protein 667 OS=Homo sapiens OX=9606 GN=ZNF667 PE=1 SV=2
431	P30041|PRD	227,5004	25,89	25,89	0	0	0	5	381515,78				5	8				Carbamid	25034,98	Peroxiredoxin-6 OS=Homo sapiens OX=9606 GN=PRDX6 PE=1 SV=3
1192	Q8IZJ3|CPM	55,16821	0,32	0,32	0,32	0,32	0,32	1	539557,75	293071,44	224863,53	291826,2	1	1	1	1	1		206701,92	C3 and PZP-like alpha-2-macroglobulin domain-containing protein 8 OS=Homo sapiens OX=9606 GN=CPAMD8 PE=1 SV=2
211	P25445|TNR	98,04495	1,79	1,79	0	0	0	1	66990,9				1	1				Carbamid	37732,2	Tumor necrosis factor receptor superfamily member 6 OS=Homo sapiens OX=9606 GN=FAS PE=1 SV=1
586	P01019|ANG	145,1996	5,15	1,44	5,15	0	0	3	36115,023	56749,445			2	1	4				53154,195	Angiotensinogen OS=Homo sapiens OX=9606 GN=AGT PE=1 SV=3
432	P01860|IGHG	225,017	6,95	0	0	6,95	3,14	5			41515,594	261197,16	1			9	2	Carbamid	49092,945	Immunoglobulin heavy constant gamma 3 OS=Homo sapiens OX=9606 GN=IGHG3 PE=1 SV=3
433	P13612|ITA4_	239,8706	4,55	0	0	0	4,55	5				100420,16	5				6	Carbamid	114899,65	Integrin alpha-4 OS=Homo sapiens OX=9606 GN=ITGA4 PE=1 SV=3
3	Q9UIW0|VA	49,43982	2,41	0	0	2,41	2,41	1			33408,87		1			2	1		30874,47	Ventral anterior homeobox 2 OS=Homo sapiens OX=9606 GN=VAX2 PE=1 SV=1
478	Q6NZI2|CAVI	84,43752	6,92	3,85	2,05	2,05	5,13	4	561667,94	94330,18	166069,53	165656,83	4	2	1	1	4		43476,133	Caveolae-associated protein 1 OS=Homo sapiens OX=9606 GN=CAVIN1 PE=1 SV=1
171	P08962|CD63	279,4099	20,59	0	0	15,13	20,59	7			2,40E+07	8,80E+07	7			22	40	Carbamid	25636,604	CD63 antigen OS=Homo sapiens OX=9606 GN=CD63 PE=1 SV=2
353	P07737|PROF	268,5612	45,71	45,71	8,57	20	20	5	1124818,9	205305,17	363478,44	179782,62	5	15	3	3	3	Deamidat	15054,226	Profilin-1 OS=Homo sapiens OX=9606 GN=PFN1 PE=1 SV=2
199	Q8NBS9|TXN	345,3069	24,77	24,77	9,95	1,39	0	13	1284258,1	81790,305			11	23	6	1		Carbamid	47628,86	Thioredoxin domain-containing protein 5 OS=Homo sapiens OX=9606 GN=TXNDC5 PE=1 SV=2
1002	Q92496|FHR	57,26463	1,04	0	1,04	1,04	0	1		7086127	1306608,6		1		1	1			65350,758	Complement factor H-related protein 4 OS=Homo sapiens OX=9606 GN=CFHR4 PE=1 SV=3
503	A6NNH2|F90	46,7746	1,31	0	0	0	1,31	1					1				1		49646,094	Protein FAM90A27P OS=Homo sapiens OX=9606 GN=FAM90A27P PE=3 SV=2
994	P01615|KVD	36,45289	5,83	5,83	0	0	0	1	92659,414				1	1					12956,743	Immunoglobulin kappa variable 2D-28 OS=Homo sapiens OX=9606 GN=IGKV2D-28 PE=1 SV=2
229	Q9UI15|TAGI	108,544	3,52	3,52	0	0	0	1	187947,52				1	1					22472,633	Transgelin-3 OS=Homo sapiens OX=9606 GN=TAGLN3 PE=1 SV=2
94	A6NL88|SHS	88,54646	1,12	0	0	1,12	1,12	1					1			2	2		56213,582	Protein shisa-7 OS=Homo sapiens OX=9606 GN=SHISA7 PE=2 SV=3
479	Q9ULL5|PRR	74,75519	1,33	0,69	1,28	0	0,64	4	493041,66	2,64E+07		8018212	4	1	9		2		211044,06	Proline-rich protein 12 OS=Homo sapiens OX=9606 GN=PRR12 PE=1 SV=3
1153	P09884|DPO	24,38223	0,41	0	0,41	0	0	1					1		1				165912,77	DNA polymerase alpha catalytic subunit OS=Homo sapiens OX=9606 GN=POLA1 PE=1 SV=2
136	P35625|TIMP	109,0967	3,79	3,79	0	0	0	1	889798,44				1	3				Carbamid	24144,979	Metalloproteinase inhibitor 3 OS=Homo sapiens OX=9606 GN=TIMP3 PE=1 SV=2
1155	O75534|CSD	43,57881	0,75	0	0,75	0	0	1		50454,98			1		1				88884,66	Cold shock domain-containing protein E1 OS=Homo sapiens OX=9606 GN=CSDE1 PE=1 SV=2
854	P12273|PIP_	57,61049	9,59	0	0	9,59	4,11	2			89314,39		1			2	1		16572,469	Prolactin-inducible protein OS=Homo sapiens OX=9606 GN=PIP PE=1 SV=1
153	Q9Y4C1|KDM	82,69222	0,45	0,45	0,45	0	0	1	2033418,5	970712,75			1	3	1				147341,16	Lysine-specific demethylase 3A OS=Homo sapiens OX=9606 GN=KDM3A PE=1 SV=4
459	Q6W0C5|DP	28,68871	3,77	0	0	3,77	3,77	1			3851886	187572,34	1			1	1	Acetylatic	17851,127	Developmental pluripotency-associated protein 3 OS=Homo sapiens OX=9606 GN=DPPA3 PE=1 SV=1
1031	Q6ZSK4|NTA	33,52007	4,29	0	0	0	4,29	1					1				1		15188,35	Putative uncharacterized protein NTM-AS1 OS=Homo sapiens OX=9606 GN=NTM-AS1 PE=5 SV=1
1183	Q9H3Q3|G3S	25,73077	1,51	0	0	1,51	0	1					1			1			46110,457	Galactose-3-O-sulfotransferase 2 OS=Homo sapiens OX=9606 GN=GAL3ST2 PE=1 SV=2
587	O75368|SH3L	145,5495	23,68	23,68	0	0	0	3	122357,34				3	3					12774,247	Adapter SH3BGRL OS=Homo sapiens OX=9606 GN=SH3BGRL PE=1 SV=1
267	P50995|ANX	306,9549	16,63	0	0	7,72	14,85	9			28320,877	394182,88	6			7	9	Carbamid	54389,707	Annexin A11 OS=Homo sapiens OX=9606 GN=ANXA11 PE=1 SV=1
1193	P02760|AMB	54,00289	1,99	1,99	0	0	1,99	1	343266,88			24181,637	1	1			1	Carbamid	38999,473	Protein AMBP OS=Homo sapiens OX=9606 GN=AMBP PE=1 SV=1
169	Q9Y4K4|M4K	106,0026	0,71	0,71	0,71	0	0	1		139313,66			1	1	2				95023,96	Mitogen-activated protein kinase kinase kinase kinase 5 OS=Homo sapiens OX=9606 GN=MAP4K5 PE=1 SV=2
1194	P29622|KAIN	24,41365	1,87	0	0	0	1,87	1				713587,1	1				1		48541,96	Kallistatin OS=Homo sapiens OX=9606 GN=SERPINA4 PE=1 SV=3
204	Q12770|SCA	42,55261	0,47	0	0	0	0,47	1				226608,28	1				1		139729,03	Sterol regulatory element-binding protein cleavage-activating protein OS=Homo sapiens OX=9606 GN=SCAP PE=1 SV=4
1195	P07384|CAN	28,41879	1,12	0	0	1,12	1,12	1			41528,89	34980,375	1			1	1		81890,05	Calpain-1 catalytic subunit OS=Homo sapiens OX=9606 GN=CAPN1 PE=1 SV=1
644	Q15907|RB1	71,51145	8,72	0	0	0	8,72	2				152922,4	2				2		24488,496	Ras-related protein Rab-11B OS=Homo sapiens OX=9606 GN=RAB11B PE=1 SV=4
72	P08758|ANX	466,3641	60,94	5,62	5,62	51,56	59,38	31	68463,305	15361,26	3,14E+07	1,12E+08	26	3	3	63	100	Acetylatic	35936,766	Annexin A5 OS=Homo sapiens OX=9606 GN=ANXA5 PE=1 SV=2
495	Q5VZR2|NTN	53,03056	0,81	0	0	0	0,81	1				29151,854	1				1	Deamidat	79010,945	NUT family member 2G OS=Homo sapiens OX=9606 GN=NUTM2G PE=3 SV=2
588	O14817|TSN	132,6364	6,72	0	0	0	6,72	3				528772,5	3				3	Carbamid	26117,785	Tetraspanin-4 OS=Homo sapiens OX=9606 GN=TSPAN4 PE=1 SV=1
189	Q14393|GAS	361,0589	19,91	19,91	11,36	0	0,88	14	1933787,2	342258,06			12	24	14		1	Carbamid	74925,26	Growth arrest-specific protein 6 OS=Homo sapiens OX=9606 GN=GAS6 PE=1 SV=3
1121	Q8NI77|KI18	34,83939	0,67	0	0	0	0,67	1				131281,55	1				1		102280,99	Kinesin-like protein KIF18A OS=Homo sapiens OX=9606 GN=KIF18A PE=1 SV=2
1032	Q86TH1|ATL	47,494	0,63	0,63	0,63	0	0	1	304970,78	254798,3			1	1	2				104620,89	ADAMTS-like protein 2 OS=Homo sapiens OX=9606 GN=ADAMTSL2 PE=1 SV=1
28	Q13395|TARE	51,41975	0,37	0,37	0,37	0	0	1	299906,97	120290,2			1	1	1				181674,75	Probable methyltransferase TARBP1 OS=Homo sapiens OX=9606 GN=TARBP1 PE=1 SV=1
177	Q6UWW9|TN	85,30539	4,11	4,11	0	4,11	4,11	1	114470,39		212701,7	228388,75	1	1		1	1		16115,85	Transmembrane protein 207 OS=Homo sapiens OX=9606 GN=TMEM207 PE=1 SV=1
86	P05556|ITB1_	452,3184	28,32	0	0	15,91	28,32	27			1340906,2	7665345,5	27			21	44	Carbamid	88415,266	Integrin beta-1 OS=Homo sapiens OX=9606 GN=ITGB1 PE=1 SV=2
543	Q9H0J9|PAR	68,53143	0,86	0,86	0	0	0	1	70058,26				1	1					79063,64	Protein mono-ADP-ribosyltransferase PARP12 OS=Homo sapiens OX=9606 GN=PARP12 PE=1 SV=1
257	Q9UHV2|SRT	64,18456	2,54	0	2,54	0	0	1		46058,367			1		2				24703,752	SERTA domain-containing protein 1 OS=Homo sapiens OX=9606 GN=SERTAD1 PE=1 SV=2
1081	Q6ZN16|M3K	23,83986	0,46	0	0	0,46	0	1					1			1			147437,08	Mitogen-activated protein kinase kinase kinase 15 OS=Homo sapiens OX=9606 GN=MAP3K15 PE=1 SV=2
859	Q01518|CAP	66,10849	3,16	0	0	3,16	0	2			63100,38		2			2			51901,457	Adenylyl cyclase-associated protein 1 OS=Homo sapiens OX=9606 GN=CAP1 PE=1 SV=5
444	Q96JG8|MAG	77,08476	0,81	0,81	0	0	0	1	116446,97				1	1					81377,555	Melanoma-associated antigen D4 OS=Homo sapiens OX=9606 GN=MAGED4 PE=1 SV=3
480	Q9UBI6|GBG	201,2273	45,83	0	0	45,83	45,83	3			238800,47	80623,11	3			4	3	Deamidat	8006,2163	Guanine nucleotide-binding protein G(I)/G(S)/G(O) subunit gamma-12 OS=Homo sapiens OX=9606 GN=GNG12 PE=1 SV=3
268	A1L4H1|SRCF	295,7555	4,77	1,34	0	0,51	4,77	9	250033,81		63979,64	572278,7	8	3		1	10	Carbamid	165742,73	Soluble scavenger receptor cysteine-rich domain-containing protein SSC5D OS=Homo sapiens OX=9606 GN=SSC5D PE=1 SV=3
904	P46939|UTRN	53,43855	0,17	0	0	0,17	0	1			489244,44		1			1			394466,2	Utrophin OS=Homo sapiens OX=9606 GN=UTRN PE=1 SV=2
925	Q96NA2|RILF	31,62601	1,75	0	0	1,75	0	1			154790,84		1			2			44200,39	Rab-interacting lysosomal protein OS=Homo sapiens OX=9606 GN=RILP PE=1 SV=1
933	Q9UGK8|SRG	24,05968	1,31	0	0	1,31	0	1			27898,795		1			1			48981,273	Secretion-regulating guanine nucleotide exchange factor OS=Homo sapiens OX=9606 GN=SERGEF PE=1 SV=2
1011	P0DPF3|NBP	41,75044	0,54	0	0	0,54	0,54	1			5235523	7,28E+07	1			2	4		127755,72	Neuroblastoma breakpoint family member 9 OS=Homo sapiens OX=9606 GN=NBPF9 PE=1 SV=1
392	Q8NBJ4|GOL	192,1799	10,22	8,73	7,98	0	0	6	241252,9	85664,38			5	5	4				45333,24	Golgi membrane protein 1 OS=Homo sapiens OX=9606 GN=GOLM1 PE=1 SV=1
693	Q7Z3V4|UBE	62,80522	0,56	0	0	0,56	0	1			57405,086		1			1			123098,05	Ubiquitin-protein ligase E3B OS=Homo sapiens OX=9606 GN=UBE3B PE=1 SV=3
861	P49862|KLK7	69,22672	7,11	0	0	3,56	7,11	2			114053,625	170335,47	2			1	2	Carbamid	27524,73	Kallikrein-7 OS=Homo sapiens OX=9606 GN=KLK7 PE=1 SV=1
862	Q9P267|MBD	137,4669	1,47	0	1,07	0,4	0,4	2		180356,27			1		1	1	1	Carbamid	159895,03	Methyl-CpG-binding domain protein 5 OS=Homo sapiens OX=9606 GN=MBD5 PE=1 SV=3
390	Q99525|H4G_	92,8242	8,16	8,16	8,16	8,16	8,16	1	312480,22	351365,9	212271,84	806305,2	1	1	2	1	1		11009,102	Histone H4-like protein type G OS=Homo sapiens OX=9606 GN=H4C7 PE=1 SV=1
44	P48552|NRIP	92,41126	0,6	0	0	0,6	0	1			146941,89		1			1			126941,914	Nuclear receptor-interacting protein 1 OS=Homo sapiens OX=9606 GN=NRIP1 PE=1 SV=2
163	Q9H4D0|CSTI	85,67709	0,73	0,73	0,73	0	0	1	285499,72	88216,09			1	1	1			Carbamid	107005,7	Calsyntenin-2 OS=Homo sapiens OX=9606 GN=CLSTN2 PE=1 SV=2
864	P21281|VATE	135,5365	3,33	0	0	0	3,33	2				120127,41	2				4		56500,727	V-type proton ATPase subunit B, brain isoform OS=Homo sapiens OX=9606 GN=ATP6V1B2 PE=1 SV=3
865	Q86UD1|OAF	62,50583	5,49	5,49	0	0	0	2	119230,41				2	2					30688,28	Out at first protein homolog OS=Homo sapiens OX=9606 GN=OAF PE=2 SV=1
1196	P35221|CTN	26,36778	0,66	0	0	0,66	0	1					1			1			100071,266	Catenin alpha-1 OS=Homo sapiens OX=9606 GN=CTNNA1 PE=1 SV=1
29	P05106|ITB3_	82,6759	0,76	0,76	0,76	0	0,76	1	200872,44	148834,94		231725,69	1	1	1		1		87057,81	Integrin beta-3 OS=Homo sapiens OX=9606 GN=ITGB3 PE=1 SV=2
1197	P30872|SSR1	29,5133	1,79	0	0	0	1,79	1				149312,11	1				1	Carbamid	42686,184	Somatostatin receptor type 1 OS=Homo sapiens OX=9606 GN=SSTR1 PE=1 SV=1
77	Q8N584|TT3	47,90091	1,03	1,03	0	0	0	1					1	1					65869,74	Tetratricopeptide repeat protein 39C OS=Homo sapiens OX=9606 GN=TTC39C PE=2 SV=2
74	P05787|K2C8	414,1141	20,91	6,63	8,9	18,84	18,84	22					1	14	31	240	131	Deamidat	53704,242	Keratin, type II cytoskeletal 8 OS=Homo sapiens OX=9606 GN=KRT8 PE=1 SV=7
60	P20908|CO5	465,3098	16,54	14,91	8,32	1,41	2,34	40	6646660,5	1,08E+07			20	59	25	8	9	Carbamid	183559,88	Collagen alpha-1(V) chain OS=Homo sapiens OX=9606 GN=COL5A1 PE=1 SV=3
481	P10599|THIO	244,8691	38,1	38,1	8,57	29,52	29,52	4	1616662,1		1677469,5	1462918,2	4	6	2	5	4	Carbamid	11737,492	Thioredoxin OS=Homo sapiens OX=9606 GN=TXN PE=1 SV=3
176	Q13609|DNS	106,8359	1,97	1,97	1,97	1,97	1,97	1	865526,8	560180,9	1456445,5	1391911,9	1	1	2	3	3	Deamidat	35503,86	Deoxyribonuclease gamma OS=Homo sapiens OX=9606 GN=DNASE1L3 PE=1 SV=1
142	Q8WXA3|RU	78,41393	0,99	0,99	0,99	0,99	0,99	1				274587,2	1	1	1	1	1		70010,21	RUN and FYVE domain-containing protein 2 OS=Homo sapiens OX=9606 GN=RUFY2 PE=1 SV=3
535	Q99932|SPA	36,07795	1,44	1,44	0	0	0	1					1	1					51138,812	Sperm-associated antigen 8 OS=Homo sapiens OX=9606 GN=SPAG8 PE=1 SV=3
1009	O95243|MBD	31,61936	1,03	0	0	1,03	0	1			63620,664		1			1			66050,9	Methyl-CpG-binding domain protein 4 OS=Homo sapiens OX=9606 GN=MBD4 PE=1 SV=1
590	P13500|CCL2	196,9481	24,24	24,24	0	0	0	3	3285012,5				2	18				Carbamid	11024,992	C-C motif chemokine 2 OS=Homo sapiens OX=9606 GN=CCL2 PE=1 SV=1
482	Q99536|VAT	230,0134	12,21	0	0	12,21	2,29	4			460324,88	161029,95	3			5	1	Carbamid	41920,24	Synaptic vesicle membrane protein VAT-1 homolog OS=Homo sapiens OX=9606 GN=VAT1 PE=1 SV=2
112	P14543|NID1	434,6675	19,25	11,39	11,15	4,17	14,19	24	1131933,5	2075328,8	369044,44	2435048,5	22	25	29	6	23	Carbamid	136377,03	Nidogen-1 OS=Homo sapiens OX=9606 GN=NID1 PE=1 SV=3

764	O75127|PTCI	37,26005	0,86	0	0	0,86	0	1			49049,004		1			1			78855,73	Pentatricopeptide repeat-containing protein 1, mitochondrial OS=Homo sapiens OX=9606 GN=PTCD1 PE=1 SV=2
616	P49356|FNTB	45,10995	1,37	0	0	1,37	0	1			52744,816		1			1		Deamidat	48773,61	Protein farnesyltransferase subunit beta OS=Homo sapiens OX=9606 GN=FNTB PE=1 SV=1
591	P55287|CAD1	88,88934	3,27	3,27	0	0	0	3	268395,53				3	3					87965,33	Cadherin-11 OS=Homo sapiens OX=9606 GN=CDH11 PE=1 SV=2
494	Q86XA9|HTR	66,28826	0,29	0	0,29	0	0,29	1				8710429	1		1		1		222003,94	HEAT repeat-containing protein 5A OS=Homo sapiens OX=9606 GN=HEATR5A PE=1 SV=2
22	Q8TDW5|SYT	25,59409	0,96	0	0	0	0,96	1					1				1		81522,9	Synaptotagmin-like protein 5 OS=Homo sapiens OX=9606 GN=SYTL5 PE=1 SV=1
867	Q9BUA3|SPN	96,76582	3,15	0	0	1,57	3,15	2			389081,3	338401,5	1			1	3		41036,8	Spindlin interactor and repressor of chromatin-binding protein OS=Homo sapiens OX=9606 GN=SPINDOC PE=1 SV=3
770	P30519|HMO	42,8563	1,9	0	0	1,9	0	1					1			1			36032,867	Heme oxygenase 2 OS=Homo sapiens OX=9606 GN=HMOX2 PE=1 SV=2
2	P52824|DGK	110,3147	0,64	0,64	0,64	0,64	0,64	1	49303,652	72603,85	821635,5	1381400,8	1	1	1	2	2		101154,89	Diacylglycerol kinase theta OS=Homo sapiens OX=9606 GN=DGKQ PE=1 SV=2
249	Q8NHV5|MO	100,8806	3,59	3,59	3,59	3,59	3,59	1	1704077,6	705186,9	3805421,8	1,56E+07	1	6	7	6	4		18249,72	Modulator of smoothened protein OS=Homo sapiens OX=9606 GN=MOSMO PE=2 SV=3
287	Q9C0H2|TTY	332,6674	16,25	0	0	5,74	16,25	9			296839,06	4746853,5	9			4	16	Oxidation	57544,72	Protein tweety homolog 3 OS=Homo sapiens OX=9606 GN=TTYH3 PE=1 SV=3
542	Q7Z3E5|ARM	24,90426	0,73	0	0	0,73	0	1			29270,012		1			1			91818,664	LisH domain-containing protein ARMC9 OS=Homo sapiens OX=9606 GN=ARMC9 PE=1 SV=3
93	Q96SM3|CPX	89,38999	0,82	0,82	0	0	0	1	58521,027				1	1					81667,6	Probable carboxypeptidase X1 OS=Homo sapiens OX=9606 GN=CPXM1 PE=2 SV=2
1198	Q7Z5L7|POD	31,08185	1,79	1,79	0	0	0	1					1	1					68975,86	Podocan OS=Homo sapiens OX=9606 GN=PODN PE=1 SV=2
71	P30531|SC6A	112,7286	1	1	1	1	1	1	63075,8	134069,75	54991,742	140715,61	1	1	1	2	2		67073,61	Sodium- and chloride-dependent GABA transporter 1 OS=Homo sapiens OX=9606 GN=SLC6A1 PE=1 SV=2
868	Q5VUA4|ZN3	54,29935	0,7	0,26	0,26	0	0,7	2				206264,03	1	1	1		2	Acetylatic	251112,36	Zinc finger protein 318 OS=Homo sapiens OX=9606 GN=ZNF318 PE=1 SV=2
454	Q8WXE0|CSK	35,01672	0,5	0,5	0,5	0	0	1	127115,24	68007,836			1	1	1			Acetylatic	126783,29	Caskin-2 OS=Homo sapiens OX=9606 GN=CASKIN2 PE=1 SV=2
237	P46976|GLYG	315,9483	22,29	0	0	22,29	22,29	8			2900419,5	6621269,5	8			24	33	Deamidat	39383,773	Glycogenin-1 OS=Homo sapiens OX=9606 GN=GYG1 PE=1 SV=4
28	P11047|LAM	540,7572	31,14	28,46	22,62	22,25	24,86	59	1,67E+07	6615867	6168485	1,38E+07	57	88	76	53	62	Carbamid	177602,86	Laminin subunit gamma-1 OS=Homo sapiens OX=9606 GN=LAMC1 PE=1 SV=3
770	Q8N7N1|F86	42,8563	2,03	0	0	2,03	0	1					1			1			32864,824	Putative protein N-methyltransferase FAM86B1 OS=Homo sapiens OX=9606 GN=FAM86B1 PE=2 SV=2
1199	O15303|GRM	25,99489	0,68	0	0	0,68	0	1			36895,15		1			1			95467,734	Metabotropic glutamate receptor 6 OS=Homo sapiens OX=9606 GN=GRM6 PE=1 SV=2
1200	P53999|TCP4	76,45841	8,66	8,66	0	0	0	1					1	1					14395,335	Activated RNA polymerase II transcriptional coactivator p15 OS=Homo sapiens OX=9606 GN=SUB1 PE=1 SV=3
269	P22735|TGM	326,6264	11,14	0	0	10,04	3,18	10			221703,86	24530,93	10			9	3		89786,7	Protein-glutamine gamma-glutamyltransferase K OS=Homo sapiens OX=9606 GN=TGM1 PE=1 SV=4
429	P36402|TCF7	54,15968	1,56	0	0	1,56	0	1			26059,824		1			1			41641,57	Transcription factor 7 OS=Homo sapiens OX=9606 GN=TCF7 PE=1 SV=3
483	A6NEL2|SWA	67,1162	3,4	0,76	2,65	0	0,76	4		439016,88		70899,88	2	1	3		1	Acetylatic	85742,43	Ankyrin repeat domain-containing protein SOWAHB OS=Homo sapiens OX=9606 GN=SOWAHB PE=1 SV=1
869	O60522|TDR	95,31281	0,57	0,29	0,29	0,57	0,29	2			1953030		1	2	2	5	5		236517,47	Tudor domain-containing protein 6 OS=Homo sapiens OX=9606 GN=TDRD6 PE=2 SV=2
881	Q96LT9|RNP	26,81628	1,16	0	0	1,16	0	1					1			1			58575,09	RNA-binding region-containing protein 3 OS=Homo sapiens OX=9606 GN=RNPC3 PE=1 SV=1
593	Q9BYE4|SPR	221,4088	61,64	0	0	61,64	61,64	3			894421,7	1087446,5	3			6	6	Carbamid	8157,6064	Small proline-rich protein 2G OS=Homo sapiens OX=9606 GN=SPRR2G PE=3 SV=1
288	P61981|1433	294,9358	25,91	21,86	11,74	19,84	19,43	8			19729,066		1	13	8	10	7	Oxidation	28302,586	14-3-3 protein gamma OS=Homo sapiens OX=9606 GN=YWHAG PE=1 SV=2
870	Q96QA5|GSD	86,13361	3,82	0	0	1,8	3,82	2			90021,8	138542,4	2			1	2	Acetylatic	49364,86	Gasdermin-A OS=Homo sapiens OX=9606 GN=GSDMA PE=1 SV=4
99	Q02818|NUC	435,5179	50,76	50,76	33,84	0	0	26	9719586	4099030,8			23	64	37			Deamidat	53879,3	Nucleobindin-1 OS=Homo sapiens OX=9606 GN=NUCB1 PE=1 SV=4
317	P61769|B2M	268,8219	52,94	47,06	32,77	5,04	26,89	7	6,45E+07	4,25E+07		2240177,8	7	59	97	1	9	Carbamid	13714,561	Beta-2-microglobulin OS=Homo sapiens OX=9606 GN=B2M PE=1 SV=1
93	Q6IQ20|NAP	26,88499	1,53	0	0	0	1,53	1				476980,78	1				1		45595,5	N-acyl-phosphatidylethanolamine-hydrolyzing phospholipase D OS=Homo sapiens OX=9606 GN=NAPEPLD PE=1 SV=2
250	Q9NZI2|KCIP	49,34548	2,64	0	0	0	2,64	1				673270,6	1				1	Deamidat	26817,395	Kv channel-interacting protein 1 OS=Homo sapiens OX=9606 GN=KCNIP1 PE=1 SV=2
354	P47929|LEG7	283,3043	50,74	0	0	42,65	19,12	7			790701,9	422203	7			7	3	Acetylatic	15075,05	Galectin-7 OS=Homo sapiens OX=9606 GN=LGALS7 PE=1 SV=2
871	P09936|UCHL	36,05584	7,17	7,17	0	0	0	2	75859,12				2	3					24824,338	Ubiquitin carboxyl-terminal hydrolase isozyme L1 OS=Homo sapiens OX=9606 GN=UCHL1 PE=1 SV=2
238	P27105|STON	317,5853	28,82	0	0	2,43	28,82	8				3329788,8	8			1	18	Oxidation	31730,791	Stomatin OS=Homo sapiens OX=9606 GN=STOM PE=1 SV=3
872	P17096|HMG	105,0799	14,02	14,02	14,02	7,48	0	2	498856,7	111199,06	17709,072		2	2	2	1			11676,02	High mobility group protein HMG-I/HMG-Y OS=Homo sapiens OX=9606 GN=HMGA1 PE=1 SV=3
873	Q9BXJ8|TAC	73,8635	1,75	0	0	1,75	0	1					1			3		Deamidat	40610,277	Ion channel TACAN OS=Homo sapiens OX=9606 GN=TMEM120A PE=1 SV=1
1032	O95450|ATS2	47,494	0,5	0,5	0,5	0	0	1	304970,78	254798,3			1	1	2				134754,8	A disintegrin and metalloproteinase with thrombospondin motifs 2 OS=Homo sapiens OX=9606 GN=ADAMTS2 PE=2 SV=2
68	P08254|MMP	450,299	44,23	27,67	44,03	0	34,59	31	850394,6	9,37E+07		6545037,5	30	25	197		37	Carbamid	53977,336	Stromelysin-1 OS=Homo sapiens OX=9606 GN=MMP3 PE=1 SV=2
20	P08779|K1C1	546,0474	80,55	13,95	19,45	80,55	71,25	66			6,85E+07	3,96E+07	27	17	27	403	236	Deamidat	51267,816	Keratin, type I cytoskeletal 16 OS=Homo sapiens OX=9606 GN=KRT16 PE=1 SV=4
130	P06396|GELS	408,4539	27,88	27,88	13,04	11,76	6,65	20	6296304	778400,1	385441,12	118029,01	20	35	15	10	5	Carbamid	85697,516	Gelsolin OS=Homo sapiens OX=9606 GN=GSN PE=1 SV=1
29	P07942|LAM	534,5424	26,37	21	18,14	16,8	21,84	62	9776218	5043020	2139096,8	9103780	55	80	73	44	62	Carbamid	198038,36	Laminin subunit beta-1 OS=Homo sapiens OX=9606 GN=LAMB1 PE=1 SV=2
444	O15061|SYNE	77,08476	0,38	0,38	0	0	0	1	116446,97				1	1					172867,75	Synemin OS=Homo sapiens OX=9606 GN=SYNM PE=1 SV=3
484	P28300|LYOX	219,9017	10,31	10,31	1,68	0	0	4	1617102,9	79281,02			4	7	2				46944,08	Protein-lysine 6-oxidase OS=Homo sapiens OX=9606 GN=LOX PE=1 SV=2
172	Q76M96|CCD	346,1106	13,79	13,79	0	0	0	16	1502086,8				15	18				Carbamid	108173,71	Coiled-coil domain-containing protein 80 OS=Homo sapiens OX=9606 GN=CCDC80 PE=1 SV=1
2	Q05DH4|FHI	36,16522	0,58	0,58	0,58	0,58	0	1	2536857,2	576682,5	524109,53		1	1	1	1			116621,25	FHF complex subunit HOOK-interacting protein 1A OS=Homo sapiens OX=9606 GN=FHIP1A PE=1 SV=2
594	Q12931|TRAF	143,1172	4,55	1,99	0	3,69	0,85	3					2	1		2	1	Deamidat	80109,99	Heat shock protein 75 kDa, mitochondrial OS=Homo sapiens OX=9606 GN=TRAP1 PE=1 SV=3
355	Q9H6X2|ANT	270,8168	10,82	0	0	0	10,82	7				739615	6				11	Carbamid	62788,973	Anthrax toxin receptor 1 OS=Homo sapiens OX=9606 GN=ANTXR1 PE=1 SV=2
485	P05120|PAI2	148,8547	10,36	4,34	10,36	0	0	4	58649,004	48572,883			4	2	4				46596,2	Plasminogen activator inhibitor 2 OS=Homo sapiens OX=9606 GN=SERPINB2 PE=1 SV=2
25	Q04695|K1C1	522,5601	79,17	9,72	12,73	79,17	56,94	55			1,80E+07	5319257,5	18	11	16	323	169	Oxidation	48105,66	Keratin, type I cytoskeletal 17 OS=Homo sapiens OX=9606 GN=KRT17 PE=1 SV=2
204	Q5T4F4|ZFY2	42,55261	1,46	0	0	0	1,46	1				226608,28	1				1		45843,45	Protrudin OS=Homo sapiens OX=9606 GN=ZFYVE27 PE=1 SV=1
428	Q12851|M4K	70,07965	0,85	0,85	0	0	0	1	250109,83				1	2					91555,95	Mitogen-activated protein kinase kinase kinase kinase 2 OS=Homo sapiens OX=9606 GN=MAP4K2 PE=1 SV=2
761	Q9UBX1|CAT	40,99163	1,24	1,24	0	0	0	1					1	1					53365,902	Cathepsin F OS=Homo sapiens OX=9606 GN=CTSF PE=1 SV=1
160	Q16270|IBP7	383,4796	57,09	57,09	10,64	0	0	15	1,69E+07	339131,06			14	66	8			Carbamid	29130,312	Insulin-like growth factor-binding protein 7 OS=Homo sapiens OX=9606 GN=IGFBP7 PE=1 SV=1
218	P28799|GRN_	336,2478	16,86	12,65	9,11	0	5,23	12	1999239,5	1092249			11	20	22		3	Carbamid	63544,453	Progranulin OS=Homo sapiens OX=9606 GN=GRN PE=1 SV=2
1115	P42338|PK3C	36,93215	0,56	0	0	0,56	0	1			259379,88		1			1		Deamidat	122762,32	Phosphatidylinositol 4,5-bisphosphate 3-kinase catalytic subunit beta isoform OS=Homo sapiens OX=9606 GN=PIK3CB PE=1 SV=1
1201	Q96EH3|MAS	56,69172	2,56	0	2,56	0	0	1		15856,893			1		1				26170,043	Mitochondrial assembly of ribosomal large subunit protein 1 OS=Homo sapiens OX=9606 GN=MALSU1 PE=1 SV=1
230	Q9HD33|RM4	39,20821	2,4	0	0	2,4	0	1			46140,46		1			1			29450,402	39S ribosomal protein L47, mitochondrial OS=Homo sapiens OX=9606 GN=MRPL47 PE=1 SV=2
1199	Q9P2H5|UBP	25,99489	0,59	0	0	0,59	0	1			36895,15		1			1			113405,02	Ubiquitin carboxyl-terminal hydrolase 35 OS=Homo sapiens OX=9606 GN=USP35 PE=1 SV=3
109	P27797|CALR	427,8575	55,4	42,21	35,01	16,31	10,07	25	1,83E+07	1,03E+07	346643,5	542080,25	25	65	49	6	7	Carbamid	48141,555	Calreticulin OS=Homo sapiens OX=9606 GN=CALR PE=1 SV=1
106	P08727|K1C1	387,6634	30,75	7,25	9	27	23,75	19			262278,06	118228,72	2	10	14	100	51	Acetylatic	44105,992	Keratin, type I cytoskeletal 19 OS=Homo sapiens OX=9606 GN=KRT19 PE=1 SV=4
948	Q9Y4F1|FARI	28,41772	0,57	0	0	0	0,57	1				53464,4	1				1		118632,93	FERM, ARHGEF and pleckstrin domain-containing protein 1 OS=Homo sapiens OX=9606 GN=FARP1 PE=1 SV=1
5	O95807|TM5	24,53426	3,82	0	0	3,82	0	1					1			1			17400,236	Transmembrane protein 50A OS=Homo sapiens OX=9606 GN=TMEM50A PE=1 SV=1
376	Q71UI9|H2A	155,9647	12,5	7,03	7,03	7,03	12,5	2	230969,52	229668,45	250080,11	1776298	2	3	4	2	7		13508,682	Histone H2A.V OS=Homo sapiens OX=9606 GN=H2AZ2 PE=1 SV=3
1202	Q9Y4F5|C170	27,37415	0,44	0	0	0,44	0	1			5041,0957		1			1			171688,03	Centrosomal protein of 170 kDa protein B OS=Homo sapiens OX=9606 GN=CEP170B PE=1 SV=4
486	O43493|TGO	204,8401	9,38	6,18	9,38	0	0	4	70164,16	168167,9			4	3	5				45879,57	Trans-Golgi network integral membrane protein 2 OS=Homo sapiens OX=9606 GN=TGOLN2 PE=1 SV=4
447	Q02539|H11_	182,3111	12,56	0	0	3,26	12,56	3				57619,617	1			1	3		21842,088	Histone H1.1 OS=Homo sapiens OX=9606 GN=H1-1 PE=1 SV=3
122	P07951|TPM	386,3293	54,23	54,23	33,8	6,69	4,58	22	790484,1	122384,18			4	36	21	3	2	Carbamid	32850,727	Tropomyosin beta chain OS=Homo sapiens OX=9606 GN=TPM2 PE=1 SV=1
460	P16520|GBB3	62,98388	2,06	0	0	2,06	0	1			20985,71		1			1			37221,137	Guanine nucleotide-binding protein G(I)/G(S)/G(T) subunit beta-3 OS=Homo sapiens OX=9606 GN=GNB3 PE=1 SV=1
1018	Q04671|P_HU	23,98892	0,72	0	0	0	0,72	1					1				1		92849,586	P protein OS=Homo sapiens OX=9606 GN=OCA2 PE=1 SV=2
846	Q8NC42|RN1	31,68901	1,5	0	0	1,5	0	1			197740,75		1			3			43164,527	E3 ubiquitin-protein ligase RNF149 OS=Homo sapiens OX=9606 GN=RNF149 PE=2 SV=2
356	O14672|ADA	272,0044	7,35	0	0	0	7,35	6				425596,4	6				7	Carbamid	84142,12	Disintegrin and metalloproteinase domain-containing protein 10 OS=Homo sapiens OX=9606 GN=ADAM10 PE=1 SV=1
1203	P29122|PCSK	28,23257	0,62	0	0	0,62	0	1			3634808,2		1			2			106420,08	Proprotein convertase subtilisin/kexin type 6 OS=Homo sapiens OX=9606 GN=PCSK6 PE=1 SV=1
198	P0DPH7|TBA	318,5372	24,89	3,33	0	24,89	12	8			310849,75		1	2		11	5	Deamidat	49959,543	Tubulin alpha-3C chain OS=Homo sapiens OX=9606 GN=TUBA3C PE=1 SV=1
896	P31749|AKT1	23,04849	1,25	0	0	1,25	0	1			340053,22		1			1			55686,414	RAC-alpha serine/threonine-protein kinase OS=Homo sapiens OX=9606 GN=AKT1 PE=1 SV=2
596	Q5VST9|OBS	74,41648	0,23	0	0,15	0,08	0	3			3721363,8		2		11	3			868483,7	Obscurin OS=Homo sapiens OX=9606 GN=OBSCN PE=1 SV=3
1204	Q5VYX0|RNL	32,02794	2,05	0	0	0	2,05	1					1				1	Deamidat	37847,297	Renalase OS=Homo sapiens OX=9606 GN=RNLS PE=1 SV=1
487	Q9UBP4|DKK	199,4139	13,43	9,43	9,14	0	0	4	283617,75	566833,94			4	7	5				38390,242	Dickkopf-related protein 3 OS=Homo sapiens OX=9606 GN=DKK3 PE=1 SV=2
76	P31939|PUR9	89,16936	1,01	0	0	1,01	1,01	1			81151,62	112538,66	1			1	1		64615,87	Bifunctional purine biosynthesis protein ATIC OS=Homo sapiens OX=9606 GN=ATIC PE=1 SV=3
42	Q05655|KPCI	71,57471	0,89	0,89	0	0	0	1	47370,332				1	1					77505,04	Protein kinase C delta type OS=Homo sapiens OX=9606 GN=PRKCD PE=1 SV=2
235	A8K7I4|CLCA	78,36797	0,66	0,66	0,66	0	0	1	201011,17				1	1	3				100225,555	Calcium-activated chloride channel regulator 1 OS=Homo sapiens OX=9606 GN=CLCA1 PE=1 SV=3
376	Q96QV6|H2A	279,6636	37,4	11,45	12,21	6,87	37,4	6	259248,44	294746,4	250080,11	2714422,5	6	4	6	2	11		14233,505	Histone H2A type 1-A OS=Homo sapiens OX=9606 GN=H2AC1 PE=1 SV=3
634	O15446|RPA3	71,26441	1,18	0	1,18	1,18	1,18	1		4961949	219925,28	3924922	1		11	8	1	Deamidat	54985,598	DNA-directed RNA polymerase I subunit RPA34 OS=Homo sapiens OX=9606 GN=POLR1G PE=1 SV=1
750	P13796|PLSL	140,2538	2,39	0,96	0	1,44	0	2			15519,787		1	1		2		Carbamid	70288,39	Plastin-2 OS=Homo sapiens OX=9606 GN=LCP1 PE=1 SV=6
880	Q9NQ87|HEY	63,51601	3,66	1,83	0	1,83	0	2	1249522,2		7258,855		2	2		1		Acetylatic	35087,164	Hairy/enhancer-of-split related with YRPW motif-like protein OS=Homo sapiens OX=9606 GN=HEYL PE=1 SV=2
1205	P68871|HBB_	49,58899	7,48	0	0	0	7,48	1				303635,16	1				1		15998,403	Hemoglobin subunit beta OS=Homo sapiens OX=9606 GN=HBB PE=1 SV=2
488	P11532|DMD	94,94569	0,73	0,49	0,16	0	0,24	4				297437,88	1	3	1		2	Deamidat	426749,62	Dystrophin OS=Homo sapiens OX=9606 GN=DMD PE=1 SV=3
2	Q9BX69|CAR	36,16522	0,58	0,58	0,58	0,58	0	1	2536857,2	576682,5	524109,53		1	1	1	1			116467,625	Caspase recruitment domain-containing protein 6 OS=Homo sapiens OX=9606 GN=CARD6 PE=1 SV=2
73	Q66K14|TBC9	81,78078	0,48	0,48	0	0,48	0	1	37493,39		68402,195		1	2		1			140524,75	TBC1 domain family member 9B OS=Homo sapiens OX=9606 GN=TBC1D9B PE=1 SV=3
188	P21359|NF1_	78,02858	0,21	0,21	0,21	0,21	0,21	1		193457,72	81789,61	329750,56	1	1	1	1	1		319372,28	Neurofibromin OS=Homo sapiens OX=9606 GN=NF1 PE=1 SV=2
881	Q96T58|MIN	57,85049	0,33	0,16	0	0,16	0	2					1	1		1		Deamidat	402248,22	Msx2-interacting protein OS=Homo sapiens OX=9606 GN=SPEN PE=1 SV=1
1206	Q14258|TRI2	31,82696	0,95	0	0	0	0,95	1				451220,03	1				1	Acetylatic	70973,4	E3 ubiquitin/ISG15 ligase TRIM25 OS=Homo sapiens OX=9606 GN=TRIM25 PE=1 SV=2
464	O95183|VAM	46,85813	6,03	0	0	0	6,03	1					1				1		12804,814	Vesicle-associated membrane protein 5 OS=Homo sapiens OX=9606 GN=VAMP5 PE=1 SV=1
882	Q8N0S2|SYCI	81,21083	3,42	0	0	1,71	1,71	2					1			1	1		39699,047	Synaptonemal complex central element protein 1 OS=Homo sapiens OX=9606 GN=SYCE1 PE=1 SV=2
775	O14497|ARI1	76,97985	0,26	0,26	0	0	0	1	21360,566				1	1					242044,61	AT-rich interactive domain-containing protein 1A OS=Homo sapiens OX=9606 GN=ARID1A PE=1 SV=3
376	Q6FI13|H2A2	279,6636	37,69	11,54	12,31	6,92	37,69	6	259248,44	294746,4	250080,11	2714422,5	6	4	6	2	11		14095,483	Histone H2A type 2-A OS=Homo sapiens OX=9606 GN=H2AC18 PE=1 SV=3
489	Q6UWP8|SBS	219,8454	8,47	0	0	0	8,47	4				55981,656	4				4		60540,83	Suprabasin OS=Homo sapiens OX=9606 GN=SBSN PE=1 SV=2
104	Q8TCS8|PNP	90,14129	0,77	0,77	0,77	0,77	0,77	1	272529,6		100229,48	94495,805	1	2	1	1	1		85950,62	Polyribonucleotide nucleotidyltransferase 1, mitochondrial OS=Homo sapiens OX=9606 GN=PNPT1 PE=1 SV=2
252	P58876|H2B1	317,3283	66,67	34,13	27,78	29,37	66,67	10	117890,07	89813,125	87343,49	987559,8	1	7	7	7	17	Oxidation	13936,161	Histone H2B type 1-D OS=Homo sapiens OX=9606 GN=H2BC5 PE=1 SV=2
1036	Q9BR61|ACB	27,37833	2,13	0	0	2,13	0	1			31607,432		1			1			31150,85	Acyl-CoA-binding domain-containing protein 6 OS=Homo sapiens OX=9606 GN=ACBD6 PE=1 SV=1

672	A1L3X0|ELOV	75,59264	2,14	2,14	2,14	2,14	2,14	1	74019,93	25637,965	84506,19		1	1	1	1	1		33356,31	Elongation of very long chain fatty acids protein 7 OS=Homo sapiens OX=9606 GN=ELOVL7 PE=1 SV=1
503	Q06278|AOX	56,75306	0,6	0	0	0	0,6	1					1				1		147917,9	Aldehyde oxidase OS=Homo sapiens OX=9606 GN=AOX1 PE=1 SV=2
598	P05114|HMG	206,1231	34	34	34	0	0	3	199488,6	80722,05			3	4	3			Deamidat	10658,897	Non-histone chromosomal protein HMG-14 OS=Homo sapiens OX=9606 GN=HMGN1 PE=1 SV=3
270	Q9P244|LRFN	58,03672	0,78	0	0,78	0,78	0	1		4008790,8	38897,34		1		1	2			82317,88	Leucine-rich repeat and fibronectin type III domain-containing protein 1 OS=Homo sapiens OX=9606 GN=LRFN1 PE=1 SV=2
261	Q9BY66|KDM	69,61014	0,39	0	0	0,39	0,39	1			156596,33	677938,1	1			1	1		174072,95	Lysine-specific demethylase 5D OS=Homo sapiens OX=9606 GN=KDM5D PE=1 SV=2
1044	P29376|LTK_	40,36036	0,69	0	0	0,69	0	1			63496,605		1			1			91681,266	Leukocyte tyrosine kinase receptor OS=Homo sapiens OX=9606 GN=LTK PE=1 SV=3
118	Q3SY84|K2C7	386,3703	21,03	4,02	7,65	17,78	18,55	22		29426,44			3	7	14	35	32	Deamidat	57291,535	Keratin, type II cytoskeletal 71 OS=Homo sapiens OX=9606 GN=KRT71 PE=1 SV=3
1207	Q8N5R6|CCD	24,61932	0,63	0,63	0	0	0	1	159047,6				1	1				Acetylatic	107215,87	Coiled-coil domain-containing protein 33 OS=Homo sapiens OX=9606 GN=CCDC33 PE=1 SV=3
1208	Q8NDV7|TNR	30,53471	0,31	0	0	0	0,31	1				52188,754	1				1		210297,19	Trinucleotide repeat-containing gene 6A protein OS=Homo sapiens OX=9606 GN=TNRC6A PE=1 SV=2
147	Q7Z628|ARH	30,49008	1,17	0	1,17	0	0	1		20245,879			1		1				67740,02	Neuroepithelial cell-transforming gene 1 protein OS=Homo sapiens OX=9606 GN=NET1 PE=1 SV=1
1098	O60269|GRIN	24,85937	1,31	0	0	1,31	0	1					1			1		Acetylatic	47450,29	G protein-regulated inducer of neurite outgrowth 2 OS=Homo sapiens OX=9606 GN=GPRIN2 PE=1 SV=2
6	Q9BSJ5|CQ0	72,24475	1,15	0	0	1,15	0	1			536444,44		1			3			67315,19	Uncharacterized protein C17orf80 OS=Homo sapiens OX=9606 GN=C17orf80 PE=1 SV=2
398	Q9HBE1|PAT.	32,09123	0,87	0,87	0,87	0	0	1	887625,8	83099,29			1	1	1				74059,97	POZ-, AT hook-, and zinc finger-containing protein 1 OS=Homo sapiens OX=9606 GN=PATZ1 PE=1 SV=1
200	P14625|ENPL	353,1892	14,45	14,45	0	0	0	13	378063,84				11	14					92468,86	Endoplasmin OS=Homo sapiens OX=9606 GN=HSP90B1 PE=1 SV=1
27	P52744|ZN13	57,95885	2,29	0	0	2,29	0	1			19414,406		1			1			30591,504	Zinc finger protein 138 OS=Homo sapiens OX=9606 GN=ZNF138 PE=1 SV=2
942	Q9UBV8|PEF	23,69609	2,11	0	0	2,11	0	1			65454,918		1			1			30380,885	Peflin OS=Homo sapiens OX=9606 GN=PEF1 PE=1 SV=1
65	Q9NZP8|C1R	142,1668	4,31	4,31	1,85	1,85	0	2	2282570,5	872878,7			2	4	2	1			53498,336	Complement C1r subcomponent-like protein OS=Homo sapiens OX=9606 GN=C1RL PE=1 SV=2
599	P08603|CFAH	67,0686	2,19	1,71	0	0,49	0	3	19175,998				2	2		1		Acetylatic	139096,31	Complement factor H OS=Homo sapiens OX=9606 GN=CFH PE=1 SV=4
428	Q8IVH8|M4K	70,07965	0,78	0,78	0	0	0	1	250109,83				1	2					101315,91	Mitogen-activated protein kinase kinase kinase kinase 3 OS=Homo sapiens OX=9606 GN=MAP4K3 PE=1 SV=1
1209	P34995|PE2R	34,45204	1,49	0	0	0	1,49	1					1				1		41801,277	Prostaglandin E2 receptor EP1 subtype OS=Homo sapiens OX=9606 GN=PTGER1 PE=2 SV=3
22	Q2M243|CCD	48,5298	0,91	0	0,91	0	0	1					1		1				75354,484	Coiled-coil domain-containing protein 27 OS=Homo sapiens OX=9606 GN=CCDC27 PE=1 SV=2
1210	P60763|RAC	38,16075	3,12	0	0	0	3,12	1				131051,61	1				1		21378,854	Ras-related C3 botulinum toxin substrate 3 OS=Homo sapiens OX=9606 GN=RAC3 PE=1 SV=1
39	Q9HCP0|KC1	114,5848	1,66	0	0	0	1,66	1				503371,78	1				2		48511,324	Casein kinase I isoform gamma-1 OS=Homo sapiens OX=9606 GN=CSNK1G1 PE=1 SV=1
1143	Q9UIL4|KIF2	112,4307	1,56	0	0	1,56	1,56	1					1			1	1		40686,3	Kinesin-like protein KIF25 OS=Homo sapiens OX=9606 GN=KIF25 PE=2 SV=2
227	Q6PJW8|CNS	97,30266	0,83	0,83	0,83	0	0	1	246061,45	69047,82			1	1	1			Carbamid	79596,92	Consortin OS=Homo sapiens OX=9606 GN=CNST PE=1 SV=3
1211	P05204|HMG	43,86702	13,33	13,33	13,33	0	0	1	131187,66	91222,516			1	1	1			Deamidat	9392,665	Non-histone chromosomal protein HMG-17 OS=Homo sapiens OX=9606 GN=HMGN2 PE=1 SV=3
1010	Q10587|TEF_	32,29033	1,98	0	0	1,98	0	1			22121,885		1			2			33247,613	Thyrotroph embryonic factor OS=Homo sapiens OX=9606 GN=TEF PE=1 SV=3
58	Q13029|PRDI	63,70379	0,41	0	0	0,41	0,41	1				237679,11	1			1	1		188915,4	PR domain zinc finger protein 2 OS=Homo sapiens OX=9606 GN=PRDM2 PE=1 SV=3
1183	A6NGR9|MR	25,73077	0,83	0	0	0,83	0	1					1			1			77186,25	Maestro heat-like repeat-containing protein family member 6 OS=Homo sapiens OX=9606 GN=MROH6 PE=4 SV=2
842	Q9H3S4|TPK	38,83734	2,47	2,47	0	0	0	1	77703,25				1	1					27265,275	Thiamin pyrophosphokinase 1 OS=Homo sapiens OX=9606 GN=TPK1 PE=1 SV=1
788	Q8IVH4|MM	29,62739	1,44	1,44	0	0	0	1	726644,5				1	1					46538,258	Methylmalonic aciduria type A protein, mitochondrial OS=Homo sapiens OX=9606 GN=MMAA PE=1 SV=1
600	Q13515|BFSF	94,54083	4,58	1,69	0	2,89	0	3					1	1		3			45879,586	Phakinin OS=Homo sapiens OX=9606 GN=BFSP2 PE=1 SV=1
173	Q08554|DSC1	383,1097	17	0	0	15,55	14,09	15			7593767	6610250	14			35	24	Carbamid	99986,83	Desmocollin-1 OS=Homo sapiens OX=9606 GN=DSC1 PE=1 SV=2
205	Q9Y6X0|SETE	24,5653	0,38	0,38	0	0	0	1	770080,5				1	2					175007,56	SET-binding protein OS=Homo sapiens OX=9606 GN=SETBP1 PE=1 SV=3
206	F5H284|PAL4	269,5659	28,05	11,59	11,59	28,05	28,05	6	212291,48	104125,625	604586,06	671093,94	4	3	3	9	8	Carbamid	18166,723	Peptidyl-prolyl cis-trans isomerase A-like 4D OS=Homo sapiens OX=9606 GN=PPIAL4D PE=3 SV=1
490	P07197|NFM	148,9669	2,4	0,98	0,98	2,4	0,98	3			30951,824		1	4	4	3	2	Deamidat	102472,055	Neurofilament medium polypeptide OS=Homo sapiens OX=9606 GN=NEFM PE=1 SV=3
224	Q6ZSZ5|ARH	101,0171	0,51	0,51	0,51	0	0	1	103782,13				1	1	1				151642,5	Rho guanine nucleotide exchange factor 18 OS=Homo sapiens OX=9606 GN=ARHGEF18 PE=1 SV=4
590	P80075|CCL8	96,79324	6,06	6,06	0	0	0	1	506907,44				1	3					11246,358	C-C motif chemokine 8 OS=Homo sapiens OX=9606 GN=CCL8 PE=1 SV=2
601	Q8IVL0|NAV	70,87794	0,8	0	0,55	0	0,25	3					1		2		1		255648	Neuron navigator 3 OS=Homo sapiens OX=9606 GN=NAV3 PE=1 SV=3
1212	O43151|TET3	26,8007	0,33	0	0	0	0,33	1				251942,77	1				1		193704,52	Methylcytosine dioxygenase TET3 OS=Homo sapiens OX=9606 GN=TET3 PE=1 SV=4
491	P09622|DLD	120,5353	6,68	0	6,68	0	0	4		44585,17			3		4				54177,25	Dihydrolipoyl dehydrogenase, mitochondrial OS=Homo sapiens OX=9606 GN=DLD PE=1 SV=2
492	Q9Y4K0|LOX	198,2355	3,88	3,88	1,68	0,78	3,1	4	38427,395			339890,6	3	9	7	3	5	Carbamid	86724,75	Lysyl oxidase homolog 2 OS=Homo sapiens OX=9606 GN=LOXL2 PE=1 SV=1
1160	Q96K78|AGR	24,41807	0,75	0	0	0	0,75	1				5785744,5	1				1	Acetylatic	88908,51	Adhesion G-protein coupled receptor G7 OS=Homo sapiens OX=9606 GN=ADGRG7 PE=1 SV=2
1061	Q9NUU7|DD	28,85608	1,26	0	0	1,26	0	1			67875,92		1			1			53975,016	ATP-dependent RNA helicase DDX19A OS=Homo sapiens OX=9606 GN=DDX19A PE=1 SV=1
1213	P55001|MFAI	29,20375	3,28	3,28	0	0	0	1	184302,38				1	1					20825,533	Microfibrillar-associated protein 2 OS=Homo sapiens OX=9606 GN=MFAP2 PE=1 SV=1
493	Q6UWF9|F18	153,9978	17,92	0	0	0	17,92	3				551908,7	3				5	Carbamid	19732,877	Protein FAM180A OS=Homo sapiens OX=9606 GN=FAM180A PE=2 SV=1
67	A1KZ92|PXD	171,2545	0,82	0,41	0	0	0,82	2	32190,668			172019,66	2	1			2	Carbamid	163686,05	Probable oxidoreductase PXDNL OS=Homo sapiens OX=9606 GN=PXDNL PE=1 SV=3
888	P61604|CH10	68,29165	14,71	7,84	6,86	0	0	2	28063,625				1	1	1				10931,6875	10 kDa heat shock protein, mitochondrial OS=Homo sapiens OX=9606 GN=HSPE1 PE=1 SV=2
109	Q9P2P1|NYN	76,04213	0,32	0	0,32	0	0	1		12289,932			1		1				208366,33	Protein NYNRIN OS=Homo sapiens OX=9606 GN=NYNRIN PE=2 SV=3
603	O75223|GGC	165,3279	14,36	0	0	9,04	9,04	3			94798,26	137843,22	3			3	2	Carbamid	21007,719	Gamma-glutamylcyclotransferase OS=Homo sapiens OX=9606 GN=GGCT PE=1 SV=1
104	O95399|UTS2	117,7338	6,45	6,45	6,45	6,45	6,45	1	980887,75	191173,34	218408,7	100684,97	1	2	3	2	1		14295,63	Urotensin-2 OS=Homo sapiens OX=9606 GN=UTS2 PE=1 SV=1
376	P16104|H2A	236,4661	29,37	6,29	6,29	6,29	29,37	4	230969,52	229668,45	250080,11	2471626,2	4	3	4	2	9		15144,567	Histone H2AX OS=Homo sapiens OX=9606 GN=H2AX PE=1 SV=2
539	P53675|CLH2	27,72617	0,73	0	0	0,73	0	1			40427,695		1			1		Carbamid	187029,9	Clathrin heavy chain 2 OS=Homo sapiens OX=9606 GN=CLTCL1 PE=1 SV=2
51	O95460|MAT	36,14	1,13	1,13	0	0	0	1	1105384,4				1	1					68486,914	Matrilin-4 OS=Homo sapiens OX=9606 GN=MATN4 PE=1 SV=3
394	Q16769|QPC	276,3698	16,9	16,9	3,05	0	0	6	1208201,5	91267,39			6	13	2				40876,543	Glutaminyl-peptide cyclotransferase OS=Homo sapiens OX=9606 GN=QPCT PE=1 SV=1
159	Q92830|KAT	117,8167	0,72	0	0	0	0,72	1				147559,02	1				1		93925,55	Histone acetyltransferase KAT2A OS=Homo sapiens OX=9606 GN=KAT2A PE=1 SV=3
17	P12109|CO6	591,3853	64,59	51,17	39,3	44,65	53,4	90	2,07E+08	4,35E+07	6,96E+07	6,37E+08	83	378	203	183	381	Deamidat	108529,39	Collagen alpha-1(VI) chain OS=Homo sapiens OX=9606 GN=COL6A1 PE=1 SV=3
78	Q96SK2|TM2	83,70251	1,07	1,07	1,07	0	0	1					1	1	1				62921,66	Transmembrane protein 209 OS=Homo sapiens OX=9606 GN=TMEM209 PE=1 SV=2
358	Q99538|LGM	239,7456	13,86	1,39	12,47	0	0	7		440192,7			6	1	10			Carbamid	49411,164	Legumain OS=Homo sapiens OX=9606 GN=LGMN PE=1 SV=1
1214	Q9NU22|MD	40,85797	0,13	0	0,13	0	0	1		1407810			1		2				632819,8	Midasin OS=Homo sapiens OX=9606 GN=MDN1 PE=1 SV=2
30	P22894|MMP	114,2276	2,14	2,14	2,14	0	0	1					1	1	1				53412,14	Neutrophil collagenase OS=Homo sapiens OX=9606 GN=MMP8 PE=1 SV=1
1215	Q96QU1|PCD	23,95141	0,41	0	0,41	0	0	1		500268,8			1		1			Acetylatic	216069,28	Protocadherin-15 OS=Homo sapiens OX=9606 GN=PCDH15 PE=1 SV=2
1031	Q7Z5Q1|CPE	33,52007	1,02	0	0	0	1,02	1					1				1		64944,152	Cytoplasmic polyadenylation element-binding protein 2 OS=Homo sapiens OX=9606 GN=CPEB2 PE=2 SV=3
113	P19013|K2C4	377,1709	19,42	9,04	11,92	18,27	19,42	19			1066664,8	847845,06	2	13	21	59	42	Deamidat	56144,023	Keratin, type II cytoskeletal 4 OS=Homo sapiens OX=9606 GN=KRT4 PE=1 SV=5
23	Q86YZ3|HOR	560,6226	20,74	1,4	3,26	19,58	17,96	70	213654,61	1678951,5	4,33E+07	3,56E+07	69	6	17	178	127	Deamidat	282389,9	Hornerin OS=Homo sapiens OX=9606 GN=HRNR PE=1 SV=2
1216	Q9H0W7|TH	26,77226	2,63	0	0	2,63	0	1			73271,13		1			1			26259,799	THAP domain-containing protein 2 OS=Homo sapiens OX=9606 GN=THAP2 PE=1 SV=1
890	Q9H4G4|GAP	150,8523	12,34	0	0	0	12,34	2				87808,13	2				2		17218,3	Golgi-associated plant pathogenesis-related protein 1 OS=Homo sapiens OX=9606 GN=GLIPR2 PE=1 SV=3
1217	Q96FQ6|S10	25,42666	10,68	0	0	10,68	0	1			30655,977		1			1			11801,392	Protein S100-A16 OS=Homo sapiens OX=9606 GN=S100A16 PE=1 SV=1
849	Q6IS14|IF5AL	29,93155	3,9	3,9	0	0	0	1	608444,94				1	1					16773,174	Eukaryotic translation initiation factor 5A-1-like OS=Homo sapiens OX=9606 GN=EIF5AL1 PE=2 SV=2
45	Q14767|LTBP	499,7792	24,99	24,99	10,98	3,13	8,84	41	5,08E+07	1550724,8	419011,34	744936	40	101	31	6	16	Carbamid	195051,5	Latent-transforming growth factor beta-binding protein 2 OS=Homo sapiens OX=9606 GN=LTBP2 PE=1 SV=3
1080	Q96L91|EP40	33,03733	0,19	0	0	0,19	0	1			176723		1			1			343489,12	E1A-binding protein p400 OS=Homo sapiens OX=9606 GN=EP400 PE=1 SV=4
318	Q4V9L6|TM1	321,4205	22,97	3,53	3,53	6,01	22,97	8	36940,53	38648,11	178079,86	2031987,4	8	1	1	2	11		29202,867	Transmembrane protein 119 OS=Homo sapiens OX=9606 GN=TMEM119 PE=1 SV=1
362	P56539|CAV	70,70054	6,62	0	0	6,62	6,62	1			1709394	273895,56	1			5	2		17259,299	Caveolin-3 OS=Homo sapiens OX=9606 GN=CAV3 PE=1 SV=1
77	P07237|PDIA	446,9233	52,76	52,76	28,54	9,65	4,53	30	9946448	729285,4	85788,086	45560,918	28	62	23	6	4	Carbamid	57116,363	Protein disulfide-isomerase OS=Homo sapiens OX=9606 GN=P4HB PE=1 SV=3
605	P01876|IGHA	88,12835	6,28	0	0	4,52	4,02	3			173936,72	176871,72	3			2	2	Carbamid	42848,508	Immunoglobulin heavy constant alpha 1 OS=Homo sapiens OX=9606 GN=IGHA1 PE=1 SV=3
732	Q8TAE8|G45	80,51206	2,7	2,7	2,7	2,7	2,7	1	110671,62		217832,83	95613,336	1	1	1	1	1	Deamidat	25383,83	Growth arrest and DNA damage-inducible proteins-interacting protein 1 OS=Homo sapiens OX=9606 GN=GADD45GIP1 PE=1 SV=1
1218	Q92896|GSL	24,12956	0,85	0	0	0	0,85	1				12480,025	1				1	Acetylatic	134551,84	Golgi apparatus protein 1 OS=Homo sapiens OX=9606 GN=GLG1 PE=1 SV=2
495	Q8IVF1|NTM	53,03056	0,68	0	0	0	0,68	1				29151,854	1				1	Deamidat	93890,234	NUT family member 2A OS=Homo sapiens OX=9606 GN=NUTM2A PE=2 SV=4
252	P23527|H2B1	299,3754	59,52	34,13	20,63	29,37	59,52	9	117890,07	89813,125	87343,49	987559,8	1	7	6	7	15	Oxidation	13906,134	Histone H2B type 1-O OS=Homo sapiens OX=9606 GN=H2BC17 PE=1 SV=3
200	Q58FF3|ENP	194,7558	5,26	5,26	0	0	0	3	81788,81				2	3					45858,99	Putative endoplasmin-like protein OS=Homo sapiens OX=9606 GN=HSP90B2P PE=5 SV=1
5	Q5SNT2|TM2	95,18884	0,9	0,9	0,9	0	0	1	7970265	233452,2			1	5	1				72235,67	Transmembrane protein 201 OS=Homo sapiens OX=9606 GN=TMEM201 PE=1 SV=1
137	P13637|AT1A	318,3988	9,28	0	0	0,79	9,28	9				350500,9	7			1	10	Carbamid	111748,54	Sodium/potassium-transporting ATPase subunit alpha-3 OS=Homo sapiens OX=9606 GN=ATP1A3 PE=1 SV=3
659	Q9NX74|DUS	42,47769	1,22	0	1,22	0	0	1		806622,25			1		3				55050,297	tRNA-dihydrouridine(20) synthase [NAD(P)+]-like OS=Homo sapiens OX=9606 GN=DUS2 PE=1 SV=1
1219	P34096|RNA	22,79074	7,48	7,48	0	0	0	1					1	1				Carbamid	16840,469	Ribonuclease 4 OS=Homo sapiens OX=9606 GN=RNASE4 PE=1 SV=3
262	Q96KR6|F21	106,8753	3,12	3,12	3,12	0	0	1					1	2	3				20424,258	Protein FAM210B, mitochondrial OS=Homo sapiens OX=9606 GN=FAM210B PE=1 SV=2
1	Q14416|GRM	77,53	0,69	0	0	0,69	0,69	1			271410,28	2124225,2	1			2	3		95567,805	Metabotropic glutamate receptor 2 OS=Homo sapiens OX=9606 GN=GRM2 PE=1 SV=2
428	P41252|SYIC_	70,07965	0,55	0,55	0	0	0	1	250109,83				1	2					144498,11	Isoleucine--tRNA ligase, cytoplasmic OS=Homo sapiens OX=9606 GN=IARS1 PE=1 SV=2
4	P02452|CO1	689,558	82,17	80,05	67,96	71,17	67,42	226	1,26E+09	3,40E+08	1,70E+08	1,49E+08	183	713	529	278	192	Acetylatic	138911,47	Collagen alpha-1(I) chain OS=Homo sapiens OX=9606 GN=COL1A1 PE=1 SV=6
564	Q9UQ16|DYN	29,83528	0,69	0	0	0,69	0	1			57581,89		1			1			97746,11	Dynamin-3 OS=Homo sapiens OX=9606 GN=DNM3 PE=1 SV=4
138	P0DMV9|HS	394,9992	25,27	5,3	0	12,48	23,09	17	85032,92		99487,875	526184,8	5	4		15	19	Deamidat	70052,23	Heat shock 70 kDa protein 1B OS=Homo sapiens OX=9606 GN=HSPA1B PE=1 SV=1
450	A0A2R8Y4L2|	94,55196	10,91	6,18	2,18	2,55	0	4	17078,705		853550,9		3	3	1	2			29173,514	Heterogeneous nuclear ribonucleoprotein A1-like 3 OS=Homo sapiens OX=9606 GN=HNRNPA1L3 PE=4 SV=1
239	P68366|TBA4	343,9825	27,46	3,35	0	25,22	9,82	10				29409,523	1	2		11	5	Deamidat	49924,4	Tubulin alpha-4A chain OS=Homo sapiens OX=9606 GN=TUBA4A PE=1 SV=1
664	Q9Y6X3|SCC	34,54774	0,98	0	0,98	0	0	1		34864,406			1		1				69081,836	MAU2 chromatid cohesion factor homolog OS=Homo sapiens OX=9606 GN=MAU2 PE=1 SV=2
183	P05155|IC1_	312,5532	21,2	7	21,2	0	0	12	270246,25	3937582,8			12	7	48			Carbamid	55154,184	Plasma protease C1 inhibitor OS=Homo sapiens OX=9606 GN=SERPING1 PE=1 SV=2
146	Q8WUX9|CHI	27,22116	1,32	0	0	0	1,32	1				29655,746	1				1	Deamidat	50910,914	Charged multivesicular body protein 7 OS=Homo sapiens OX=9606 GN=CHMP7 PE=1 SV=1
319	O00115|DNS	287,8207	21,39	18,61	17,5	0	0	8	839546,2	364341,25			8	10	12			Carbamid	39580,84	Deoxyribonuclease-2-alpha OS=Homo sapiens OX=9606 GN=DNASE2 PE=1 SV=2
1071	Q00532|CDKI	27,42501	1,68	0	1,68	0	0	1					1		1				41802,504	Cyclin-dependent kinase-like 1 OS=Homo sapiens OX=9606 GN=CDKL1 PE=1 SV=6
1178	Q9P2K6|KLH	53,57428	1,19	0	1,19	0	0	1		28655			1		1				56867,824	Kelch-like protein 42 OS=Homo sapiens OX=9606 GN=KLHL42 PE=1 SV=2
190	P06744|G6PI_	198,8363	17,74	17,74	6,99	1,25	0	14	1051236,2	223094,12	40344,656		12	15	6	1			63147,12	Glucose-6-phosphate isomerase OS=Homo sapiens OX=9606 GN=GPI PE=1 SV=4
1220	P08174|DAF_	25,91393	1,84	0	0	0	1,84	1				19466,457	1				1	Carbamid	41400,13	Complement decay-accelerating factor OS=Homo sapiens OX=9606 GN=CD55 PE=1 SV=4
182	P04350|TBB4	271,8908	13,96	0	0	13,96	6,31	6			544713,5	189163	6			8	4	Oxidation	49585,766	Tubulin beta-4A chain OS=Homo sapiens OX=9606 GN=TUBB4A PE=1 SV=2

220	Q14764|MVP	327,1786	11,87	0	0	8,96	8,4	12			3500720,8	382265,2	11			10	10	Acetylatic	99326,89	Major vault protein OS=Homo sapiens OX=9606 GN=MVP PE=1 SV=4
882	Q8IY82|DRC7	23,65603	0,69	0	0	0	0,69	1					1				1		103497,04	Dynein regulatory complex subunit 7 OS=Homo sapiens OX=9606 GN=DRC7 PE=1 SV=3
93	Q8TAT6|NPL	26,88499	0,99	0	0	0	0,99	1				476980,78	1				1		68120,23	Nuclear protein localization protein 4 homolog OS=Homo sapiens OX=9606 GN=NPLOC4 PE=1 SV=3
163	P14316|IRF2	89,8404	1,72	1,72	1,72	0	0	1	253479,38	118913,28			1	1	1				39354,43	Interferon regulatory factor 2 OS=Homo sapiens OX=9606 GN=IRF2 PE=1 SV=2
896	Q8N7G0|PO	53,55359	3,66	1,83	1,83	1,83	1,83	2			340053,22		1	1	1	1	1	Acetylatic	36051,14	POU domain, class 5, transcription factor 2 OS=Homo sapiens OX=9606 GN=POU5F2 PE=1 SV=1
897	Q9NRX5|SER	166,2292	4,42	0	0	0	4,42	2				254764,48	2				3		50494,945	Serine incorporator 1 OS=Homo sapiens OX=9606 GN=SERINC1 PE=1 SV=1
495	Q86Y26|NUT	53,03056	0,53	0	0	0	0,53	1				29151,854	1				1	Deamidat	120314,2	NUT family member 1 OS=Homo sapiens OX=9606 GN=NUTM1 PE=1 SV=2
372	P51878|CASP	63,27258	1,38	1,38	1,38	0	0	1	87615,94	21512,354			1	1	1			Carbamid	49735,645	Caspase-5 OS=Homo sapiens OX=9606 GN=CASP5 PE=1 SV=3
139	Q08380|LG3E	397,3119	31,45	10,94	14,02	7,01	31,45	16	731075,8	969980,9	262807,75	2,13E+07	16	9	15	4	54	Oxidation	65331	Galectin-3-binding protein OS=Homo sapiens OX=9606 GN=LGALS3BP PE=1 SV=1
1071	Q5W041|ARN	27,42501	0,69	0	0,69	0	0	1					1		1				96404,86	Armadillo repeat-containing protein 3 OS=Homo sapiens OX=9606 GN=ARMC3 PE=2 SV=2
57	Q5ST30|SYV	95,64761	0,66	0,66	0	0	0	1					1	5					118489,84	Valine--tRNA ligase, mitochondrial OS=Homo sapiens OX=9606 GN=VARS2 PE=1 SV=2
83	Q13822|ENP	416,8155	25,72	12,98	24,22	0	3,82	27	1,41E+07	3,10E+07		64013,758	25	21	92		4	Carbamid	98993,586	Ectonucleotide pyrophosphatase/phosphodiesterase family member 2 OS=Homo sapiens OX=9606 GN=ENPP2 PE=1 SV=3
285	P43699|NKX	42,64934	1,89	0	0	0	1,89	1				70901,19	1				1	Deamidat	38596,28	Homeobox protein Nkx-2.1 OS=Homo sapiens OX=9606 GN=NKX2-1 PE=1 SV=1
350	Q8NEV4|MY	27,74284	0,37	0	0	0,37	0	1			45709,625		1			1			186208,47	Myosin-IIIa OS=Homo sapiens OX=9606 GN=MYO3A PE=1 SV=2
770	Q96G04|EF2	42,8563	1,82	0	0	1,82	0	1					1			1			36915,395	Protein-lysine N-methyltransferase EEF2KMT OS=Homo sapiens OX=9606 GN=EEF2KMT PE=1 SV=2
221	Q13867|BLM	347,1742	20,88	0	0	20,88	16,48	12			2027038,8	1086605,2	11			16	10	Carbamid	52562,23	Bleomycin hydrolase OS=Homo sapiens OX=9606 GN=BLMH PE=1 SV=1
1221	P12757|SKIL	26,16646	0,88	0	0,88	0	0	1					1		2				76975,945	Ski-like protein OS=Homo sapiens OX=9606 GN=SKIL PE=1 SV=2
51	Q86X40|LRC	297,32061	1,63	0	0	0	1,63	1				69994,57	1				2		41912,043	Leucine-rich repeat-containing protein 28 OS=Homo sapiens OX=9606 GN=LRRC28 PE=2 SV=1
240	Q15293|RCN	326,5009	27,19	27,19	11,18	0	0	10	5477745,5	294733,1			9	20	6			Deamidat	38889,996	Reticulocalbin-1 OS=Homo sapiens OX=9606 GN=RCN1 PE=1 SV=1
435	Q00888|PSG	227,0488	10,26	0	10,26	0	0	5					1		11			Carbamid	47112,82	Pregnancy-specific beta-1-glycoprotein 4 OS=Homo sapiens OX=9606 GN=PSG4 PE=2 SV=3
75	P68133|ACTS	423,8546	38,46	32,36	9,55	38,46	29,71	22			1,07E+07	672794,6	1	34	7	121	42	Oxidation	42051,027	Actin, alpha skeletal muscle OS=Homo sapiens OX=9606 GN=ACTA1 PE=1 SV=1
241	P04040|CATA	338,9553	17,84	0	0	13,28	11,57	11			518892,3	752096,3	10			9	8		59756,168	Catalase OS=Homo sapiens OX=9606 GN=CAT PE=1 SV=3
900	Q63ZY3|KAN	76,89574	1,65	0	0	0	1,65	2				47852,31	1				3		91174,445	KN motif and ankyrin repeat domain-containing protein 2 OS=Homo sapiens OX=9606 GN=KANK2 PE=1 SV=1
52	Q4ZG55|GREI	95,16628	0,31	0	0	0,31	0	1					1			1			216467,3	Protein GREB1 OS=Homo sapiens OX=9606 GN=GREB1 PE=2 SV=1
6	P47902|CDX	149,33768	2,26	2,26	2,26	0	0	1	258931,9	88723,61			1	1	2				28137,877	Homeobox protein CDX-1 OS=Homo sapiens OX=9606 GN=CDX1 PE=1 SV=2
494	O60568|PLO	115,6487	4,2	0	4,2	0	2,17	4		16967,672		8710429	4		5		2		84785,15	Multifunctional procollagen lysine hydroxylase and glycosyltransferase LH3 OS=Homo sapiens OX=9606 GN=PLOD3 PE=1 SV=1
35	P35442|TSP2	514,8782	37,97	33,02	27,39	32,25	33,87	44	1,33E+07	4163045,8	6135056	1,91E+07	37	94	72	76	97	Carbamid	129991,234	Thrombospondin-2 OS=Homo sapiens OX=9606 GN=THBS2 PE=1 SV=2
901	O00585|CCL2	111,4868	9,7	9,7	0	0	0	2	228840,3				1	2				Carbamid	14646,14	C-C motif chemokine 21 OS=Homo sapiens OX=9606 GN=CCL21 PE=1 SV=1
1222	O15287|FAN	23,37854	0,96	0	0	0,96	0	1					1			1		Deamidat	68553,64	Fanconi anemia group G protein OS=Homo sapiens OX=9606 GN=FANCG PE=1 SV=1
242	Q96HF1|SFR	298,6335	24,75	24,75	0	0	0	9	2370950,5				8	17				Deamidat	33490,06	Secreted frizzled-related protein 2 OS=Homo sapiens OX=9606 GN=SFRP2 PE=1 SV=2
846	Q96NG3|OD	31,68901	0,89	0	0	0,89	0	1			197740,75		1			3			76655,31	Outer dynein arm-docking complex subunit 4 OS=Homo sapiens OX=9606 GN=ODAD4 PE=1 SV=2
1061	Q5VU97|CAH	28,85608	0,47	0	0	0,47	0	1			67875,92		1			1			142289,62	VWFA and cache domain-containing protein 1 OS=Homo sapiens OX=9606 GN=CACHD1 PE=2 SV=2
76	P08133|ANX	466,8432	48,14	0	0	40,71	31,05	32			5020458,5	2316813,5	30			57	35	Acetylatic	75873,266	Annexin A6 OS=Homo sapiens OX=9606 GN=ANXA6 PE=1 SV=3
606	P22392|NDK	137,8742	23,68	23,68	4,61	0	0	3	65593,58	32134,826			3	4	1				17298,031	Nucleoside diphosphate kinase B OS=Homo sapiens OX=9606 GN=NME2 PE=1 SV=1
1223	P05387|RLA2	114,1446	10,43	10,43	0	0	0	1	12695,494				1	2					11664,933	60S acidic ribosomal protein P2 OS=Homo sapiens OX=9606 GN=RPLP2 PE=1 SV=1
1224	P34059|GAL	23,11045	1,34	0	1,34	0	0	1		51620,75			1		1				58026,027	N-acetylgalactosamine-6-sulfatase OS=Homo sapiens OX=9606 GN=GALNS PE=1 SV=1
222	P31944|CASF	339,603	35,95	0	0	30,58	33,06	11			1215186	1188682,8	11			9	11	Oxidation	27679,512	Caspase-14 OS=Homo sapiens OX=9606 GN=CASP14 PE=1 SV=2
1067	P15918|RAG	124,35381	0,58	0	0	0,58	0	1			37122,6		1			1			119097,05	V(D)J recombination-activating protein 1 OS=Homo sapiens OX=9606 GN=RAG1 PE=1 SV=2
201	P06756|ITAV	355,5071	11,45	0	0	2,86	10,69	13			37585,902	848132	13			3	14	Carbamid	116037,86	Integrin alpha-V OS=Homo sapiens OX=9606 GN=ITGAV PE=1 SV=2
359	P35749|MYH	251,4258	2,99	0,91	0	2,99	0	7			77214,25		1	2		9			227339,23	Myosin-11 OS=Homo sapiens OX=9606 GN=MYH11 PE=1 SV=3
495	Q2LD37|BLTF	151,9318	0,48	0,12	0,12	0,12	0,36	4				29151,854	1	1	1	1	4	Deamidat	555481,75	Bridge-like lipid transfer protein family member 1 OS=Homo sapiens OX=9606 GN=BLTP1 PE=1 SV=2
1040	Q12873|CHD	23,64662	0,3	0	0	0	0,3	1				91289,55	1				1		226591,67	Chromodomain-helicase-DNA-binding protein 3 OS=Homo sapiens OX=9606 GN=CHD3 PE=1 SV=3
496	P12955|PEPD	220,6965	8,92	8,92	0	0	0	4	79900,375				4	5				Acetylatic	54548,17	Xaa-Pro dipeptidase OS=Homo sapiens OX=9606 GN=PEPD PE=1 SV=3
1225	P29474|NOS	24,10782	0,5	0,5	0	0	0	1					1	1					133274,78	Nitric oxide synthase, endothelial OS=Homo sapiens OX=9606 GN=NOS3 PE=1 SV=4
903	Q96KJ4|MSL	63,4271	1,71	0,85	0,85	0	0	2		22226,035			1	1	1				74540,484	Mesothelin-like protein OS=Homo sapiens OX=9606 GN=MSLNL PE=3 SV=3
283	P78386|KRT8	291,5569	10,06	1,38	1,38	5,13	8,88	7			159061,31	356090,03	5	2	2	7	9	Carbamid	55802,113	Keratin, type II cuticular Hb5 OS=Homo sapiens OX=9606 GN=KRT85 PE=1 SV=1
904	Q8TE56|ATS	165,94866	1,1	0	0,55	0,55	0	2			489244,44		1		1	1		Acetylatic	121126,945	A disintegrin and metalloproteinase with thrombospondin motifs 17 OS=Homo sapiens OX=9606 GN=ADAMTS17 PE=2 SV=2
320	P35318|ADM	262,0913	32,97	9,19	32,97	0	5,95	8	651384	2215061		94881,766	8	2	13		1		20420,379	Pro-adrenomedullin OS=Homo sapiens OX=9606 GN=ADM PE=1 SV=1
255	Q6IF42|OR2	52,44891	1,89	1,89	1,89	1,89	1,89	1				236152,39	1	1	1	1	1		35819,98	Olfactory receptor 2A2 OS=Homo sapiens OX=9606 GN=OR2A2 PE=2 SV=2
1210	P63000|RAC	138,16075	3,12	0	0	0	3,12	1				131051,61	1				1		21450,102	Ras-related C3 botulinum toxin substrate 1 OS=Homo sapiens OX=9606 GN=RAC1 PE=1 SV=1
504	Q96FG2|ELM	34,65174	1,57	0	0	1,57	0	1			44113,773		1			1			43046,195	ELMO domain-containing protein 3 OS=Homo sapiens OX=9606 GN=ELMOD3 PE=1 SV=2
42	Q8N118|CP4	196,36436	1,18	1,18	1,18	1,18	1,18	1	562619,4	146808,31	156077,06	54969,727	1	1	3	2	1		58875,246	Cytochrome P450 4X1 OS=Homo sapiens OX=9606 GN=CYP4X1 PE=1 SV=1
88	P28332|ADH	41,77088	1,63	1,63	0	0	0	1	1113543,1				1	2					39072,58	Alcohol dehydrogenase 6 OS=Homo sapiens OX=9606 GN=ADH6 PE=1 SV=3
608	Q9HAU5|REN	59,34212	1,42	0,94	0	0	0,47	3	115692,805				1	2			2	Acetylatic	147810	Regulator of nonsense transcripts 2 OS=Homo sapiens OX=9606 GN=UPF2 PE=1 SV=1
61	Q9BXY5|CAY	79,20356	1,44	1,44	1,44	0	0	1					1	1	3				63835,457	Calcyphosin-2 OS=Homo sapiens OX=9606 GN=CAPS2 PE=2 SV=2
8	P15924|DESP	667,9322	49,15	0,21	0,21	46,92	40,82	175			5,96E+07	5,08E+07	157	1	1	315	244	Carbamid	331773,88	Desmoplakin OS=Homo sapiens OX=9606 GN=DSP PE=1 SV=3
514	Q96RS6|NUD	44,08848	1,03	0	0	0	1,03	1					1				1		66755,836	NudC domain-containing protein 1 OS=Homo sapiens OX=9606 GN=NUDCD1 PE=1 SV=2
1226	Q9H9A7|RMI	28,83618	2,24	0	0	0	2,24	1				2494332	1				1	Carbamid	70144,44	RecQ-mediated genome instability protein 1 OS=Homo sapiens OX=9606 GN=RMI1 PE=1 SV=3
905	P20962|PTM	124,9551	11,76	11,76	10,78	0	0	2	171912,25	99761,43			2	3	1				11529,8955	Parathymosin OS=Homo sapiens OX=9606 GN=PTMS PE=1 SV=2
1063	Q8N145|LGI3	24,44333	1,09	0	0	1,09	0	1			245290,78		1			1			61704,496	Leucine-rich repeat LGI family member 3 OS=Homo sapiens OX=9606 GN=LGI3 PE=1 SV=1
543	Q8N961|ABT	68,53143	0,59	0,59	0	0	0	1	70058,26				1	1					113656,125	Ankyrin repeat and BTB/POZ domain-containing protein 2 OS=Homo sapiens OX=9606 GN=ABTB2 PE=2 SV=2
497	Q8TF66|LRC1	129,8633	6,88	0	0	0	6,88	4				320579,97	4				4	Carbamid	64366,188	Leucine-rich repeat-containing protein 15 OS=Homo sapiens OX=9606 GN=LRRC15 PE=2 SV=2
1227	Q07955|SRSF	41,99897	3,63	3,63	0	0	0	1	13914,349				1	1				Carbamid	27744,574	Serine/arginine-rich splicing factor 1 OS=Homo sapiens OX=9606 GN=SRSF1 PE=1 SV=2
29	Q7Z2Y5|NRK	115,7723	0,38	0,38	0,38	0,38	0,38	1	411798,44	248102,77	94237,42	370433,34	1	1	2	1	1		178479,44	Nik-related protein kinase OS=Homo sapiens OX=9606 GN=NRK PE=1 SV=2
1187	Q13219|PAP	50,75201	0,37	0,37	0,37	0	0,37	1	29039,11	15819,885		72394,5	1	1	1		1		180972,56	Pappalysin-1 OS=Homo sapiens OX=9606 GN=PAPPA PE=1 SV=3
970	P63120|VPK	127,98863	3,85	3,85	0	0	0	1	24682,994				1	2					17106,723	Endogenous retrovirus group K member 19 Pro protein OS=Homo sapiens OX=9606 GN=ERVK-19 PE=3 SV=1
1228	Q8N883|ZN6	25,71197	1,03	1,03	0	0	0	1	93775,37				1	1					67214,64	Zinc finger protein 614 OS=Homo sapiens OX=9606 GN=ZNF614 PE=1 SV=2
1229	Q9NRA2|S17	29,86704	2,02	0	0	2,02	0	1			20254,271		1			1		Carbamid	54639,547	Sialin OS=Homo sapiens OX=9606 GN=SLC17A5 PE=1 SV=2
512	Q8TAA3|PSM	98,15766	4,3	0	0	0	4,3	1				44183,73	1				1		28529,81	Proteasome subunit alpha-type 8 OS=Homo sapiens OX=9606 GN=PSMA8 PE=2 SV=3
396	P15311|EZRI	253,3905	8,02	6,48	1,71	1,54	0	6	136128,69				2	8	1	1			69412,805	Ezrin OS=Homo sapiens OX=9606 GN=EZR PE=1 SV=4
123	Q8WZ42|TITI	204,8169	0,39	0,11	0,13	0,16	0,13	22		1117945,9	6491223	2171092,5	10	7	10	19	9	Deamidat	3816030	Titin OS=Homo sapiens OX=9606 GN=TTN PE=1 SV=4
78	O95825|QOR	95,60358	1,72	1,72	1,72	0	0	1	258749,34	189501,06			1	1	2			Oxidation	38696,527	Quinone oxidoreductase-like protein 1 OS=Homo sapiens OX=9606 GN=CRYZL1 PE=1 SV=2
564	P50570|DYN	29,83528	0,69	0	0	0,69	0	1			57581,89		1			1			98064,305	Dynamin-2 OS=Homo sapiens OX=9606 GN=DNM2 PE=1 SV=2
1216	Q86YA3|ZGR	26,77226	0,29	0	0	0,29	0	1			73271,13		1			1			236601,66	Protein ZGRF1 OS=Homo sapiens OX=9606 GN=ZGRF1 PE=1 SV=3
712	Q8NEZ4|KM	33,73256	0,12	0	0,12	0	0	1		56877,754			1		2				541369,6	Histone-lysine N-methyltransferase 2C OS=Homo sapiens OX=9606 GN=KMT2C PE=1 SV=3
540	P56715|RP1	37,4525	0,28	0	0	0	0,28	1				22246,697	1				1		240661,28	Oxygen-regulated protein 1 OS=Homo sapiens OX=9606 GN=RP1 PE=1 SV=1
223	P50395|GDIB	336,5292	25,62	19,55	1,35	3,37	2,7	12	267501,47		53196,06	5443,1245	9	11	1	1	1	Carbamid	50663,242	Rab GDP dissociation inhibitor beta OS=Homo sapiens OX=9606 GN=GDI2 PE=1 SV=2
1230	Q9H223|EHD	79,50666	2,22	0	0	0	2,22	1					1				1		61175,125	EH domain-containing protein 4 OS=Homo sapiens OX=9606 GN=EHD4 PE=1 SV=1
382	P57727|TMP	42,21737	1,32	0	0	1,32	1,32	1				34022,58	1			1	1	Acetylatic	49404,664	Transmembrane protease serine 3 OS=Homo sapiens OX=9606 GN=TMPRSS3 PE=1 SV=2
360	P55072|TERA	271,0374	7,82	1,61	0	3,35	6,95	7	30763,76		66769,836	638483,44	7	1		3	9		89321,8	Transitional endoplasmic reticulum ATPase OS=Homo sapiens OX=9606 GN=VCP PE=1 SV=4
202	P29401|TKT	321,4569	18,46	17,5	6,74	2,25	0	12	1219099,2	15182,823	31181,19		11	16	4	2		Deamidat	67877,63	Transketolase OS=Homo sapiens OX=9606 GN=TKT PE=1 SV=3
584	P51610|HCF	32,2939	0,29	0	0	0	0,29	1				52988,613	1				1		208732,48	Host cell factor 1 OS=Homo sapiens OX=9606 GN=HCFC1 PE=1 SV=2
377	Q5W150|YTO	23,57562	4,29	0	4,29	0	0	1					1		1				15095,49	Putative uncharacterized protein MGC163334 OS=Homo sapiens OX=9606 PE=1 SV=1
328	Q9P2M7|CIN	38,18213	0,5	0,5	0,5	0	0	1	517809,8				1	1	1				137056,66	Cingulin OS=Homo sapiens OX=9606 GN=CGN PE=1 SV=3
138	Q6UWB1|I27	116,2287	0,94	0	0	0,94	0,94	1				62876,43	1			1	1		69473,79	Interleukin-27 receptor subunit alpha OS=Homo sapiens OX=9606 GN=IL27RA PE=1 SV=2
1231	Q15468|STIL	22,93061	0,47	0	0	0	0,47	1				184038,62	1				1		142954,53	SCL-interrupting locus protein OS=Homo sapiens OX=9606 GN=STIL PE=1 SV=2
498	P51991|ROA	118,0354	7,94	0	7,94	0	1,85	4		1543227,6		405568,5	4		12		1		39594,934	Heterogeneous nuclear ribonucleoprotein A3 OS=Homo sapiens OX=9606 GN=HNRNPA3 PE=1 SV=2
361	P00441|SOD	241,2601	32,47	24,03	0	22,08	16,88	7	606032,8		132098,25	36941,22	6	7		4	3	Carbamid	15935,737	Superoxide dismutase [Cu-Zn] OS=Homo sapiens OX=9606 GN=SOD1 PE=1 SV=2
196	A8MUU1|FB	211,8519	33,66	0	5,94	33,66	33,66	4			2001394,2	1397627,6	3		1	11	10	Deamidat	11298,834	Putative fatty acid-binding protein 5-like protein 3 OS=Homo sapiens OX=9606 GN=FABP5P3 PE=5 SV=1
593	P35325|SPR	180,0902	30,56	0	0	30,56	30,56	2			894421,7	1087446,5	2			5	5	Carbamid	7975,4277	Small proline-rich protein 2B OS=Homo sapiens OX=9606 GN=SPRR2B PE=2 SV=1
321	Q99816|TS10	279,9242	20,51	0	0	0	20,51	8				1065154	7				9	Acetylatic	43944,316	Tumor susceptibility gene 101 protein OS=Homo sapiens OX=9606 GN=TSG101 PE=1 SV=2
908	Q9BZI1|IRX2	56,31747	4,25	0	4,25	0	0	2		554399,56			2		2				49129,08	Iroquois-class homeodomain protein IRX-2 OS=Homo sapiens OX=9606 GN=IRX2 PE=1 SV=2
305	Q08357|S20	33,78748	0,92	0,92	0	0	0	1	253790,36				1	3					70392,414	Sodium-dependent phosphate transporter 2 OS=Homo sapiens OX=9606 GN=SLC20A2 PE=1 SV=1
376	Q16777|H2A	279,6636	37,98	11,63	12,4	6,98	37,98	6	259248,44	294746,4	250080,11	2714422,5	6	4	6	2	11		13988,369	Histone H2A type 2-C OS=Homo sapiens OX=9606 GN=H2AC20 PE=1 SV=4
909	P00533|EGFR	134,0603	1,4	0	0,5	0,5	1,4	2					1		1	1	2		134277,39	Epidermal growth factor receptor OS=Homo sapiens OX=9606 GN=EGFR PE=1 SV=2
499	Q4LDE5|SVE	95,03565	0,87	0,36	0,7	0	0	4	45808,19	41827,234			4	2	3			Carbamid	390170	Sushi, von Willebrand factor type A, EGF and pentraxin domain-containing protein 1 OS=Homo sapiens OX=9606 GN=SVEP1 PE=1 SV=3
1189	Q9UHA3|RLP	36,74253	3,68	0	0	0	3,68	1				1,67E+07	1				2		19621,094	Probable ribosome biogenesis protein RLP24 OS=Homo sapiens OX=9606 GN=RSL24D1 PE=1 SV=1
249	Q15562|TEA	79,5391	1,34	1,34	1,34	1,34	1,34	1	1444697,2	550149,44	488433,5	2769301	1	1	1	1	1		49242,51	Transcriptional enhancer factor TEF-4 OS=Homo sapiens OX=9606 GN=TEAD2 PE=1 SV=2
328	Q76FK4|NOL	65,72373	0,51	0	0	0,51	0,51	1			64781,062	89241,63	1			1	1		131616,48	Nucleolar protein 8 OS=Homo sapiens OX=9606 GN=NOL8 PE=1 SV=1
413	P68431|H31	118,3275	23,53	4,41	4,41	5,15	23,53	5	340159,38	464861,53	729542	5116208	4	1	1	1	7	Oxidation	15404,081	Histone H3.1 OS=Homo sapiens OX=9606 GN=H3C1 PE=1 SV=2
609	Q14112|NID	148,5748	2,04	0,73	0	0	2,04	3					1	1			4		151253,8	Nidogen-2 OS=Homo sapiens OX=9606 GN=NID2 PE=1 SV=3

1232	Q9NR82|KCN	25,54047	0,64	0	0	0	0,64	1					1				1			102179,08	Potassium voltage-gated channel subfamily KQT member 5 OS=Homo sapiens OX=9606 GN=KCNQ5 PE=1 SV=3
5	P08123|CO1A	682,3222	80,67	78,48	71,82	50,66	52,78	215	8,57E+08	2,89E+08	3,96E+07	6,91E+07	191	839	644	154	170	Acetylatic	129313,89	Collagen alpha-2(I) chain OS=Homo sapiens OX=9606 GN=COL1A2 PE=1 SV=7
1233	P0DOY3|IGLC	53,65772	9,43	0	0	9,43	9,43	1			96670,7	37221,582	1			1	1		11265,552	Immunoglobulin lambda constant 3 OS=Homo sapiens OX=9606 GN=IGLC3 PE=1 SV=1
29	Q8TCU6|PRE	93,3657	0,42	0	0,42	0	0,42	1				81946,266	1		1		1		186203,36	Phosphatidylinositol 3,4,5-trisphosphate-dependent Rac exchanger 1 protein OS=Homo sapiens OX=9606 GN=PREX1 PE=1 SV=3
1123	O15195|VILL_	23,45577	0,7	0	0	0,7	0	1			51930,484		1			1			95907,4	Villin-like protein OS=Homo sapiens OX=9606 GN=VILL PE=1 SV=3
630	Q6P4R8|NFR	50,61808	0,46	0	0	0,46	0,46	1			116166,08	198165,22	1			1	1		139001,23	Nuclear factor related to kappa-B-binding protein OS=Homo sapiens OX=9606 GN=NFRKB PE=1 SV=2
1234	P02766|TTHY	44,46783	4,08	0	4,08	4,08	0	1		79501,35	36570,387		1		2	1			15887,0205	Transthyretin OS=Homo sapiens OX=9606 GN=TTR PE=1 SV=1
322	P01040|CYTA	298,6444	84,69	0	19,39	55,1	65,31	8		27136,367	774618,56	2045075,8	8		3	8	9	Acetylatic	11006,488	Cystatin-A OS=Homo sapiens OX=9606 GN=CSTA PE=1 SV=1
123	Q6P587|FAHI	23,89835	2,68	0	2,68	0	0	1		65427,94			1		1				24842,941	Acylpyruvase FAHD1, mitochondrial OS=Homo sapiens OX=9606 GN=FAHD1 PE=1 SV=2
1235	O15164|TIF1/	22,8164	0,76	0	0	0,76	0	1					1			1			116831,23	Transcription intermediary factor 1-alpha OS=Homo sapiens OX=9606 GN=TRIM24 PE=1 SV=3
362	Q03135|CAV:	268,5627	33,71	0	0	27,53	29,21	6			2187145,5	480347,94	6			12	6	Oxidation	20471,615	Caveolin-1 OS=Homo sapiens OX=9606 GN=CAV1 PE=1 SV=4
1	P50851|LRBA	42,41132	0,21	0	0	0	0,21	1				208673,22	1				1		319107,88	Lipopolysaccharide-responsive and beige-like anchor protein OS=Homo sapiens OX=9606 GN=LRBA PE=1 SV=4
564	Q9NPF7|IL23	29,83528	3,17	0	0	3,17	0	1			57581,89		1			1			20729,734	Interleukin-23 subunit alpha OS=Homo sapiens OX=9606 GN=IL23A PE=1 SV=1
8	P52732|KIF1:	103,5417	0,66	0	0	0,66	0,66	1			190432,53	170457,27	1			1	1		119158,984	Kinesin-like protein KIF11 OS=Homo sapiens OX=9606 GN=KIF11 PE=1 SV=2
38	Q9H9A5|CNC	98,17121	0,81	0,81	0,81	0,81	0,81	1	3087237,5	1196134,4	87243,42	196865,27	1	2	2	1	1		82309,695	CCR4-NOT transcription complex subunit 10 OS=Homo sapiens OX=9606 GN=CNOT10 PE=1 SV=1
1236	P53985|MOT	63,1677	1,6	0	0	1,6	1,6	1			7829,176	12862,768	1			1	1		53944,145	Monocarboxylate transporter 1 OS=Homo sapiens OX=9606 GN=SLC16A1 PE=1 SV=3
912	P25787|PSA2	52,93394	5,98	0	0	5,98	2,99	2			203146,47	76320,59	2			2	1		25898,58	Proteasome subunit alpha type-2 OS=Homo sapiens OX=9606 GN=PSMA2 PE=1 SV=2
191	P09619|PGFF	368,5434	12,12	0	0	0	12,12	14				912892,25	12				16	Carbamid	123968,02	Platelet-derived growth factor receptor beta OS=Homo sapiens OX=9606 GN=PDGFRB PE=1 SV=1
323	Q8WVV4|PO	273,9516	12,05	0	0	10,36	7,47	8			243384,53	131394,78	8			8	5	Carbamid	68065,05	Protein POF1B OS=Homo sapiens OX=9606 GN=POF1B PE=1 SV=3
57	Q86UV5|UBP	58,29671	0,58	0,58	0	0	0	1	133760,92				1	1					119032,27	Ubiquitin carboxyl-terminal hydrolase 48 OS=Homo sapiens OX=9606 GN=USP48 PE=1 SV=1
1237	P52333|JAK3	30,30827	0,53	0,53	0	0	0	1	114838,36				1	1					125098,88	Tyrosine-protein kinase JAK3 OS=Homo sapiens OX=9606 GN=JAK3 PE=1 SV=2
1238	P62906|RL10.	26,43053	3,69	0	0	0	3,69	1				190933,22	1				1		24831,303	60S ribosomal protein L10a OS=Homo sapiens OX=9606 GN=RPL10A PE=1 SV=2
500	P02774|VTDE	230,5319	8,02	8,02	0	0	0	4	172313,05				4	5				Carbamid	52917,53	Vitamin D-binding protein OS=Homo sapiens OX=9606 GN=GC PE=1 SV=2
770	Q0VFZ6|CF2:	42,8563	1,09	0	0	1,09	0	1					1			1			66402,64	Cilia- and flagella- associated protein 210 OS=Homo sapiens OX=9606 GN=CFAP210 PE=1 SV=2
226	P62195|PRS8	82,66166	1,48	1,48	0	0	0	1	544477,3				1	1					45626,004	26S proteasome regulatory subunit 8 OS=Homo sapiens OX=9606 GN=PSMC5 PE=1 SV=1
5	P0DTL6|ZTRF	84,24882	1,73	1,49	1,73	0	1,49	2					1	3	2		1		43813,18	Zinc finger TRAF-type-containing protein 1 OS=Homo sapiens OX=9606 GN=ZFTRAF1 PE=2 SV=1
131	Q9NYP3|DON	26,63197	1,06	0	0	1,06	0	1					1			1			62746,816	Protein downstream neighbor of Son OS=Homo sapiens OX=9606 GN=DONSON PE=1 SV=2
610	Q07020|RL18	179,2944	17,02	0	0	0	17,02	3				65949,68	3				3		21634,482	60S ribosomal protein L18 OS=Homo sapiens OX=9606 GN=RPL18 PE=1 SV=2
1172	P24855|DNA:	29,77078	2,13	0	0	2,13	0	1			251203,05		1			1			31433,727	Deoxyribonuclease-1 OS=Homo sapiens OX=9606 GN=DNASE1 PE=1 SV=1
639	Q8IZL8|PELP:	26,4958	0,53	0	0,53	0	0	1					1		1				119699,625	Proline-, glutamic acid- and leucine-rich protein 1 OS=Homo sapiens OX=9606 GN=PELP1 PE=1 SV=2
37	Q9BXX0|EMII	506,6834	39,03	0	11,97	6,08	39,03	44		428275,16	531361,5	1,66E+07	43		17	8	81	Carbamid	115687,23	EMILIN-2 OS=Homo sapiens OX=9606 GN=EMILIN2 PE=1 SV=3
1122	Q9ULI4|KI26/	29,25815	0,32	0	0	0	0,32	1				173536,34	1				1		194589,62	Kinesin-like protein KIF26A OS=Homo sapiens OX=9606 GN=KIF26A PE=1 SV=3
235	Q9H3G5|CPV	94,14457	1,26	1,26	1,26	0	0	1	229314,33	22136,396			1	1	3				54163,785	Probable serine carboxypeptidase CPVL OS=Homo sapiens OX=9606 GN=CPVL PE=1 SV=2
56	O00422|SAP:	80,91282	3,92	0	0	3,92	3,92	1			1044845,06	7799888	1			2	4		17561,066	Histone deacetylase complex subunit SAP18 OS=Homo sapiens OX=9606 GN=SAP18 PE=1 SV=1
382	Q9H4A3|WN	42,21737	0,25	0	0	0,25	0,25	1				34022,58	1			1	1	Acetylatic	250793,75	Serine/threonine-protein kinase WNK1 OS=Homo sapiens OX=9606 GN=WNK1 PE=1 SV=2
1165	Q6NUJ2|CK0	26,4375	3,05	0	3,05	0	0	1					1		1				20622,596	Uncharacterized protein C11orf87 OS=Homo sapiens OX=9606 GN=C11orf87 PE=1 SV=2
22	Q9UBX3|DIC_	122,5736	2,44	2,44	2,44	2,44	2,44	2	1171430,9	984360	3810864	4,02E+07	1	2	6	2	6		31282,46	Mitochondrial dicarboxylate carrier OS=Homo sapiens OX=9606 GN=SLC25A10 PE=1 SV=2
69	Q8N1N4|K2C	468,1739	45,58	4,23	4,23	45,58	37,31	32			4899397	3571507,5	21	6	9	66	53	Carbamid	56865,824	Keratin, type II cytoskeletal 78 OS=Homo sapiens OX=9606 GN=KRT78 PE=1 SV=2
1196	P26232|CTNA	26,36778	0,63	0	0	0,63	0	1					1			1			105312,94	Catenin alpha-2 OS=Homo sapiens OX=9606 GN=CTNNA2 PE=1 SV=5
468	P10145|IL8_H	22,78029	6,06	0	0	0	6,06	1				1443703,5	1				2		11098,118	Interleukin-8 OS=Homo sapiens OX=9606 GN=CXCL8 PE=1 SV=1
243	P08476|INHB	320,1808	22,07	22,07	0	0	0	11	816585				11	15				Carbamid	47442,27	Inhibin beta A chain OS=Homo sapiens OX=9606 GN=INHBA PE=1 SV=2
40	P0C221|CC17	51,44929	0,76	0	0	0,76	0,76	1			36178,82	36294,188	1			1	1		93625,664	Coiled-coil domain-containing protein 175 OS=Homo sapiens OX=9606 GN=CCDC175 PE=4 SV=2
397	Q15238|PSG5	215,2673	14,03	2,09	14,03	0	0	6		15315,066			2	1	12			Carbamid	37713,125	Pregnancy-specific beta-1-glycoprotein 5 OS=Homo sapiens OX=9606 GN=PSG5 PE=1 SV=3
398	O94913|PCF1	151,9276	1,99	0,77	1,22	0,77	0,39	6	887625,8	83099,29	289892,6	353094,47	3	2	3	2	1		173050,19	Pre-mRNA cleavage complex 2 protein Pcf11 OS=Homo sapiens OX=9606 GN=PCF11 PE=1 SV=3
1239	P61371|ISL1_	34,35205	1,72	0	0	0	1,72	1					1				1		39035,75	Insulin gene enhancer protein ISL-1 OS=Homo sapiens OX=9606 GN=ISL1 PE=1 SV=1
439	Q96A05|VAT	104,925	9,73	0	0	0	9,73	3				101098,76	3				3		26074,482	V-type proton ATPase subunit E 2 OS=Homo sapiens OX=9606 GN=ATP6V1E2 PE=1 SV=1
915	Q14118|DAG	172,8357	3,46	3,46	0	0	0	2	14948,208				2	2					97441,484	Dystroglycan 1 OS=Homo sapiens OX=9606 GN=DAG1 PE=1 SV=2
1240	Q1A5X6|IQC.	27,11605	3,77	0	0	0	3,77	1				25546,465	1				1		18225,889	IQ domain-containing protein J OS=Homo sapiens OX=9606 GN=IQCJ PE=2 SV=1
611	P18084|ITB5_	203,6799	3,13	0	0	0	3,13	3				176477,4	3				4	Carbamid	88054,21	Integrin beta-5 OS=Homo sapiens OX=9606 GN=ITGB5 PE=1 SV=1
223	Q96PX9|PKH	58,63444	0,47	0,47	0	0	0	1	92286,85				1	1					139667,44	Pleckstrin homology domain-containing family G member 4B OS=Homo sapiens OX=9606 GN=PLEKHG4B PE=1 SV=4
454	A6NKF2|ARI:	35,01672	1,46	1,46	1,46	0	0	1	127115,24	68007,836			1	1	1			Acetylatic	44073,047	AT-rich interactive domain-containing protein 3C OS=Homo sapiens OX=9606 GN=ARID3C PE=1 SV=1
916	Q8IY81|SPB1	48,5162	1,42	0	0,71	0,71	0	2		221905,69			1		1	1		Acetylatic	96558,484	pre-rRNA 2'-O-ribose RNA methyltransferase FTSJ3 OS=Homo sapiens OX=9606 GN=FTSJ3 PE=1 SV=2
6	P35527|K1C9	654,8596	84,43	62,76	54,74	83,79	81,22	163	3,93E+07	4,76E+07	2,45E+09	1,62E+09	157	119	152	966	685	Acetylatic	62064,32	Keratin, type I cytoskeletal 9 OS=Homo sapiens OX=9606 GN=KRT9 PE=1 SV=3
291	P01008|ANT:	239,9852	12,07	6,9	9,7	0	0	9	107022,8	2995358			9	7	22				52602,43	Antithrombin-III OS=Homo sapiens OX=9606 GN=SERPINC1 PE=1 SV=1
78	Q8N442|GUF	98,68415	0,9	0,9	0,9	0	0,9	1	248306,47	147861,9		51556,223	1	2	1		1		74327,84	Translation factor GUF1, mitochondrial OS=Homo sapiens OX=9606 GN=GUF1 PE=1 SV=1
36	Q13423|NNT	99,98731	0,55	0,55	0,55	0,55	0,55	1	2467557,5	434323,44	193445,61	999734,94	1	1	1	2	1		113895,586	NAD(P) transhydrogenase, mitochondrial OS=Homo sapiens OX=9606 GN=NNT PE=1 SV=3
363	P25786|PSA1	289,7814	23,95	7,6	12,93	16,73	13,31	7	34906,883	52632,703	267296,44	185150,75	7	2	4	5	4	Carbamid	29555,588	Proteasome subunit alpha type-1 OS=Homo sapiens OX=9606 GN=PSMA1 PE=1 SV=1
328	Q5VWZ2|LYP	38,18213	2,53	2,53	2,53	0	0	1	517809,8				1	1	1				26316,414	Lysophospholipase-like protein 1 OS=Homo sapiens OX=9606 GN=LYPLAL1 PE=1 SV=3
739	Q9HB96|FAN	84,83708	1,31	1,12	1,12	1,12	1,31	2				938182,56	1	3	3	8	4		58710,926	Fanconi anemia group E protein OS=Homo sapiens OX=9606 GN=FANCE PE=1 SV=1
270	P13639|EF2_	273,8964	9,56	0	0,7	5,59	7,11	10		4008790,8	237502,6	175371,02	9		1	7	8		95338,14	Elongation factor 2 OS=Homo sapiens OX=9606 GN=EEF2 PE=1 SV=4
1207	Q96MI9|CBP(	24,61932	0,54	0,54	0	0	0	1	159047,6				1	1				Acetylatic	125330,23	Cytosolic carboxypeptidase 4 OS=Homo sapiens OX=9606 GN=AGBL1 PE=1 SV=3
997	P52895|AK1C	35,18394	1,86	0	1,86	0	0	1					1		1				36735,29	Aldo-keto reductase family 1 member C2 OS=Homo sapiens OX=9606 GN=AKR1C2 PE=1 SV=3
81	P51530|DNA:	93,6294	0,57	0,57	0,57	0,57	0,57	1	1,58E+07	9244560	462313,25	1002603,1	1	6	5	3	3		120414,7	DNA replication ATP-dependent helicase/nuclease DNA2 OS=Homo sapiens OX=9606 GN=DNA2 PE=1 SV=3
324	P00558|PGK1	272,4527	15,59	0	0	13,91	6,71	8			1367671,8	73602,8	8			7	5	Carbamid	44614,68	Phosphoglycerate kinase 1 OS=Homo sapiens OX=9606 GN=PGK1 PE=1 SV=3
75	P68032|ACTC	423,8546	38,46	32,36	9,55	38,46	29,71	22			1,07E+07	672794,6	1	34	7	121	42	Oxidation	42018,97	Actin, alpha cardiac muscle 1 OS=Homo sapiens OX=9606 GN=ACTC1 PE=1 SV=1
1004	P12268|IMDH	25,61178	1,17	0	0	0	1,17	1				50510,844	1				1		55804,98	Inosine-5'-monophosphate dehydrogenase 2 OS=Homo sapiens OX=9606 GN=IMPDH2 PE=1 SV=2
316	P0DP23|CALM	295,4051	41,61	38,93	30,87	33,56	30,87	7	5591565,5	2249549,2	2484052,8	2222430,5	7	13	11	10	10	Acetylatic	16837,582	Calmodulin-1 OS=Homo sapiens OX=9606 GN=CALM1 PE=1 SV=1
917	P62249|RS16	86,00331	10,96	0	0	10,96	0	2			10965,992		2			2		Carbamid	16445,312	40S ribosomal protein S16 OS=Homo sapiens OX=9606 GN=RPS16 PE=1 SV=2
745	Q01105|SET_	57,36063	5,52	3,45	0	2,07	0	2					1	1		1			33488,88	Protein SET OS=Homo sapiens OX=9606 GN=SET PE=1 SV=3
501	Q15286|RAB:	162,8735	16,92	0	0	8,46	11,44	4					1			3	3	Acetylatic	23025,215	Ras-related protein Rab-35 OS=Homo sapiens OX=9606 GN=RAB35 PE=1 SV=1
1241	O94856|NFA:	28,97614	0,59	0	0,59	0	0	1		3916,275			1		1				150026,78	Neurofascin OS=Homo sapiens OX=9606 GN=NFASC PE=1 SV=4
292	Q06481|APLF	248,4632	9,17	1,83	9,17	0	0	9	26012,47	364096,28			6	2	18			Carbamid	86955,77	Amyloid beta precursor like protein 2 OS=Homo sapiens OX=9606 GN=APLP2 PE=1 SV=2
659	Q70EL4|UBP4	42,47769	0,53	0	0,53	0	0	1		806622,25			1		3				122809,16	Ubiquitin carboxyl-terminal hydrolase 43 OS=Homo sapiens OX=9606 GN=USP43 PE=1 SV=2
6	Q502W7|CCC	76,21806	1,07	1,07	0	1,07	1,07	1	95524,04		1177229,9	711697,1	1	1		2	1		65314,703	Coiled-coil domain-containing protein 38 OS=Homo sapiens OX=9606 GN=CCDC38 PE=2 SV=1
327	P01861|IGHG	242,9029	10,35	0	0	10,35	0	4			533714,5		2			8		Carbamid	43831,66	Immunoglobulin heavy constant gamma 4 OS=Homo sapiens OX=9606 GN=IGHG4 PE=1 SV=2
203	P41222|PTGD	310,2623	43,16	40	25,79	12,63	16,32	8	1,31E+07	4842346	512131,1	952094,75	8	38	21	2	3	Oxidation	21028,816	Prostaglandin-H2 D-isomerase OS=Homo sapiens OX=9606 GN=PTGDS PE=1 SV=1
687	Q8TER5|ARH	38,66667	0,39	0	0,39	0	0	1		58114,875			1		1				164657,6	Rho guanine nucleotide exchange factor 40 OS=Homo sapiens OX=9606 GN=ARHGEF40 PE=1 SV=3
918	Q9Y639|NPTI	178,3486	5,03	0	0	0	5,03	2				137710,33	2				3	Carbamid	44387,18	Neuroplastin OS=Homo sapiens OX=9606 GN=NPTN PE=1 SV=2
1242	Q9BXM0|PRA	25,49164	0,41	0	0	0,41	0	1					1			1			154904,64	Periaxin OS=Homo sapiens OX=9606 GN=PRX PE=1 SV=2
919	Q8IW75|SPA	46,24966	3,14	0	0	1,69	1,45	2			15211,207	104471,26	2			1	1		47175,004	Serpin A12 OS=Homo sapiens OX=9606 GN=SERPINA12 PE=1 SV=1
96	Q8TAX9|GSD	22,80223	1,44	1,44	0	0	0	1	609901,94				1	1					47347,94	Gasdermin-B OS=Homo sapiens OX=9606 GN=GSDMB PE=1 SV=3
613	Q9UK41|VPS	125,313	19,91	0	0	0	19,91	3				585844	3				5	Acetylatic	25425,162	Vacuolar protein sorting-associated protein 28 homolog OS=Homo sapiens OX=9606 GN=VPS28 PE=1 SV=1
9	Q8N1T3|MYC	23,09759	0,58	0	0	0,58	0	1			30291,865		1			1			119036,79	Unconventional myosin-Ih OS=Homo sapiens OX=9606 GN=MYO1H PE=1 SV=2
1095	Q9BTE6|AAS	24,87802	1,46	1,46	0	0	0	1	328019,5				1	1					45479,55	Alanyl-tRNA editing protein Aarsd1 OS=Homo sapiens OX=9606 GN=AARSD1 PE=1 SV=2
901	O75200|NPIB	32,753	1,45	1,45	0	0	0	1	228840,3				1	1				Deamidat	47713,74	Nuclear pore complex-interacting protein family member B7 OS=Homo sapiens OX=9606 GN=NPIPB7 PE=3 SV=3
87	P14618|KPYN	458,0809	50,47	18,46	0	44,44	36,72	28	679637,6		8949034	4215319	26	12		47	27	Acetylatic	57936,883	Pyruvate kinase PKM OS=Homo sapiens OX=9606 GN=PKM PE=1 SV=4
93	Q8IUX7|AEBF	449,7396	23,75	17,88	7,25	3,02	20,98	26	3894298,8	278636,5	276725,06	5560804	24	31	12	5	31	Oxidation	130928,74	Adipocyte enhancer-binding protein 1 OS=Homo sapiens OX=9606 GN=AEBP1 PE=1 SV=1
2	P02751|FINC	690,0407	58,98	48,97	36,33	51,64	54,22	201	6,88E+08	1,89E+08	4,09E+08	9,18E+08	197	910	543	699	817	Carbamid	272320,34	Fibronectin OS=Homo sapiens OX=9606 GN=FN1 PE=1 SV=5
1243	Q9HCU5|PRE	35,84707	1,68	0	0	0	1,68	1					1				1		45468,168	Prolactin regulatory element-binding protein OS=Homo sapiens OX=9606 GN=PREB PE=1 SV=2
305	Q9C0D2|CE2:	33,78748	0,23	0,23	0	0	0	1	253790,36				1	3					295176,34	Centrosomal protein of 295 kDa OS=Homo sapiens OX=9606 GN=CEP295 PE=1 SV=4
315	P09972|ALDC	195,0205	10,99	1,65	0	10,99	1,65	3			29588,379		2	1		3	1	Carbamid	39455,86	Fructose-bisphosphate aldolase C OS=Homo sapiens OX=9606 GN=ALDOC PE=1 SV=2
16	P02538|K2C6	582,476	76,42	21,63	24,65	76,42	66,49	84			1,37E+07	8086519,5	5	25	50	477	305	Acetylatic	60044,945	Keratin, type II cytoskeletal 6A OS=Homo sapiens OX=9606 GN=KRT6A PE=1 SV=3
896	Q9Y2K7|KDM	23,04849	0,52	0	0	0,52	0	1			340053,22		1			1			132792,77	Lysine-specific demethylase 2A OS=Homo sapiens OX=9606 GN=KDM2A PE=1 SV=3
1081	Q0VAK6|LMC	23,83986	1,07	0	0	1,07	0	1					1			1			64914,227	Leiomodin-3 OS=Homo sapiens OX=9606 GN=LMOD3 PE=1 SV=1
255	Q8IVS2|FABD	52,44891	1,54	1,54	1,54	1,54	1,54	1				236152,39	1	1	1	1	1		42961,496	Malonyl-CoA-acyl carrier protein transacylase, mitochondrial OS=Homo sapiens OX=9606 GN=MCAT PE=1 SV=2
73	A8K979|ERI2	81,78078	0,87	0,87	0	0,87	0	1	37493,39		68402,195		1	2		1			77400,63	ERI1 exoribonuclease 2 OS=Homo sapiens OX=9606 GN=ERI2 PE=1 SV=2
151	Q69YH5|CDC.	97,12222	0,59	0,59	0,59	0,59	0,59	1	1478311,5	578411,6	90406,26	109973,81	1	2	2	1	1		112676,36	Cell division cycle-associated protein 2 OS=Homo sapiens OX=9606 GN=CDCA2 PE=1 SV=2
436	P55083|MFA	80,92539	12,16	9,8	12,16	0	0	5	1990910,5	1037002,6			4	11	7				28648,23	Microfibril-associated glycoprotein 4 OS=Homo sapiens OX=9606 GN=MFAP4 PE=1 SV=2
27	Q9C0G6|DYH	98,15766	0,17	0,17	0,17	0	0	1	218950,19				1	1	1				475982,56	Dynein axonemal heavy chain 6 OS=Homo sapiens OX=9606 GN=DNAH6 PE=2 SV=3
999	Q5TCY1|TTBK	44,83564	0,53	0,53	0,53	0	0,53	1	77391,94	303497,2		229608,75	1	2	6		2		142737,1	Tau-tubulin kinase 1 OS=Homo sapiens OX=9606 GN=TTBK1 PE=1 SV=2
230	P55081|MFAI	39,20821	1,37	0	0	1,37	0	1			46140,46		1			1			51958,44	Microfibrillar-associated protein 1 OS=Homo sapiens OX=9606 GN=MFAP1 PE=1 SV=2

614	P27824|CALX	68,15538	5,74	0	0	5,74	0	3			90199,586		3			3			67568,3	Calnexin OS=Homo sapiens OX=9606 GN=CANX PE=1 SV=2
1199	Q14833|GRM	25,99489	0,66	0	0	0,66	0	1			36895,15		1			1			101867,81	Metabotropic glutamate receptor 4 OS=Homo sapiens OX=9606 GN=GRM4 PE=1 SV=1
411	Q14008|CKA	29,13054	0,3	0	0	0	0,3	1				114344,34	1				1		225494,55	Cytoskeleton-associated protein 5 OS=Homo sapiens OX=9606 GN=CKAP5 PE=1 SV=3
1244	P18124|RL7_	73,12813	4,44	0	0	0	4,44	1				20506,184	1				1		29225,77	60S ribosomal protein L7 OS=Homo sapiens OX=9606 GN=RPL7 PE=1 SV=1
617	A6NFA1|TIKI	83,19873	1,16	0	0	1,16	0	1					1			1			57421,152	Metalloprotease TIKI2 OS=Homo sapiens OX=9606 GN=TRABD2B PE=1 SV=2
399	P02771|FETA	242,5038	7,72	7,72	0	0	2,96	6	3623167,8				6	13			2	Acetylatic	68677,56	Alpha-fetoprotein OS=Homo sapiens OX=9606 GN=AFP PE=1 SV=1
659	O60583|CCN	42,47769	0,82	0	0,82	0	0	1		806622,25			1		3				81028,94	Cyclin-T2 OS=Homo sapiens OX=9606 GN=CCNT2 PE=1 SV=2
615	Q5SYB0|FRPI	76,05875	1,2	0,82	0,82	0	0,38	3	382554,2				1	3	2		3	Acetylatic	173436,61	FERM and PDZ domain-containing protein 1 OS=Homo sapiens OX=9606 GN=FRMPD1 PE=1 SV=1
867	Q5DID0|URO	34,70901	0,46	0	0	0,46	0,46	1			389081,3	338401,5	1			1	1		144293,83	Uromodulin-like 1 OS=Homo sapiens OX=9606 GN=UMODL1 PE=2 SV=2
770	I6L899|GOG8	42,8563	0,95	0	0	0,95	0	1					1			1			71489,96	Golgin subfamily A member 8R OS=Homo sapiens OX=9606 GN=GOLGA8R PE=3 SV=1
50	Q9H993|ARM	93,17941	1,36	0	0	0	1,36	1				82518,06	1				1		51172,324	Damage-control phosphatase ARMT1 OS=Homo sapiens OX=9606 GN=ARMT1 PE=1 SV=1
673	Q96DT5|DYH	33,06132	0,13	0	0	0	0,13	1				1253976	1				1	Deamidat	520369,34	Dynein axonemal heavy chain 11 OS=Homo sapiens OX=9606 GN=DNAH11 PE=1 SV=4
224	Q02809|PLOI	335,8362	15,82	14,72	6,74	0	3,44	12	581599,3	96592,29		128580,09	11	17	8		3	Carbamid	83550,19	Procollagen-lysine,2-oxoglutarate 5-dioxygenase 1 OS=Homo sapiens OX=9606 GN=PLOD1 PE=1 SV=2
429	Q6ZTN6|AN1	118,8111	1,16	1,16	1,16	1,16	1,16	2	489938,22	91849,77	58832,07	232439,36	1	1	1	2	1		68171,83	Ankyrin repeat domain-containing protein 13D OS=Homo sapiens OX=9606 GN=ANKRD13D PE=1 SV=3
1158	P32189|GLPK	36,17757	1,25	0	0	1,25	0	1					1			1			61244,625	Glycerol kinase OS=Homo sapiens OX=9606 GN=GK PE=1 SV=3
204	Q9P2E9|RRBI	295,4359	6,45	5,89	3,55	0,43	0,43	11	6262586	104498,125		226608,28	10	10	6	1	1	Acetylatic	152456,22	Ribosome-binding protein 1 OS=Homo sapiens OX=9606 GN=RRBP1 PE=1 SV=5
12	P13647|K2C5	589,3161	68,64	27,97	33,73	68,64	60,68	93	773996,75	1152805,9	1,75E+08	1,29E+08	51	33	69	511	309	Carbamid	62378,34	Keratin, type II cytoskeletal 5 OS=Homo sapiens OX=9606 GN=KRT5 PE=1 SV=3
616	Q92804|RBP	84,27519	3,55	2,53	1,01	2,03	0	3	23313,305		52744,816		2	2	1	3		Deamidat	61829,94	TATA-binding protein-associated factor 2N OS=Homo sapiens OX=9606 GN=TAF15 PE=1 SV=1
4	O15212|PFD	98,53243	4,65	4,65	4,65	4,65	0	1	44638,28	49256,445	45390,445		1	2	2	1			14582,658	Prefoldin subunit 6 OS=Homo sapiens OX=9606 GN=PFDN6 PE=1 SV=1
617	P01903|DRA_	173,9824	12,2	0	0	12,2	0	3			128080,76		3			6			28620,947	HLA class II histocompatibility antigen, DR alpha chain OS=Homo sapiens OX=9606 GN=HLA-DRA PE=1 SV=2
56	Q9H190|SDC	148,0148	3,42	0	0	3,42	3,42	2			1608496,6	6315995	1			4	4	Acetylatic	31593,729	Syntenin-2 OS=Homo sapiens OX=9606 GN=SDCBP2 PE=1 SV=2
923	Q4LE39|ARI4	68,76745	1,07	0,61	0	0,46	0,46	2	207221,73				1	1		1	1		147809,48	AT-rich interactive domain-containing protein 4B OS=Homo sapiens OX=9606 GN=ARID4B PE=1 SV=2
364	P31151|S10A	276,5283	36,63	0	0	24,75	35,64	6			313610,56	158582,06	6			9	4	Carbamid	11470,973	Protein S100-A7 OS=Homo sapiens OX=9606 GN=S100A7 PE=1 SV=4
1143	O14522|PTPF	112,4307	0,42	0	0	0,42	0,42	1					1			1	1		162134,06	Receptor-type tyrosine-protein phosphatase T OS=Homo sapiens OX=9606 GN=PTPRT PE=1 SV=6
235	Q8NHS2|AAT	85,95554	1,43	1,43	1,43	0	0	1	164047,08	165661,67			1	1	1				47305,004	Putative aspartate aminotransferase, cytoplasmic 2 OS=Homo sapiens OX=9606 GN=GOT1L1 PE=2 SV=2
1245	Q16760|DGKI	49,04963	0,49	0	0	0	0,49	1				41473,586	1				1		134525,5	Diacylglycerol kinase delta OS=Homo sapiens OX=9606 GN=DGKD PE=1 SV=4
97	P54764|EPHA	97,62494	0,61	0,61	0,61	0,61	0,61	1	95119,79	167831,61	71115,19	96219,95	1	1	1	1	1		109859,97	Ephrin type-A receptor 4 OS=Homo sapiens OX=9606 GN=EPHA4 PE=1 SV=1
32	Q9NTK1|DEP	95,24703	2,83	2,83	0	2,83	2,83	1			753473,44	2035653,6	1	2		4	5		23405,549	Protein DEPP1 OS=Homo sapiens OX=9606 GN=DEPP1 PE=1 SV=2
100	O00391|QSO	438,1632	35,21	34,4	22,36	0,8	0	27	5995007,5	2042995,1			24	59	39	1		Acetylatic	82577,73	Sulfhydryl oxidase 1 OS=Homo sapiens OX=9606 GN=QSOX1 PE=1 SV=3
924	Q14019|COTI	67,15322	11,27	11,27	0	0	0	2	100674,21				2	3					15944,978	Coactosin-like protein OS=Homo sapiens OX=9606 GN=COTL1 PE=1 SV=3
900	P02649|APOI	53,13668	2,52	0	0	0	2,52	1				47852,31	1				1		36154,08	Apolipoprotein E OS=Homo sapiens OX=9606 GN=APOE PE=1 SV=1
1246	Q9Y6X8|ZHX	26,3409	0,96	0	0	0	0,96	1				77403,42	1				1		92307	Zinc fingers and homeoboxes protein 2 OS=Homo sapiens OX=9606 GN=ZHX2 PE=1 SV=1
249	Q9NRR5|UBC	100,8806	1	1	1	1	1	1	1704077,6	705186,9	3805421,8	1,56E+07	1	6	7	6	4		63852,93	Ubiquilin-4 OS=Homo sapiens OX=9606 GN=UBQLN4 PE=1 SV=2
925	Q8N3K9|CMY	44,60904	0,32	0,15	0	0,17	0	2	638082,25		154790,84		2	1		2		Deamidat	449211	Cardiomyopathy-associated protein 5 OS=Homo sapiens OX=9606 GN=CMYA5 PE=1 SV=3
437	P11233|RALA	123,5826	14,08	0	0	6,31	10,68	5				42300,168	2			2	4		23566,79	Ras-related protein Ral-A OS=Homo sapiens OX=9606 GN=RALA PE=1 SV=1
926	O75448|MED	42,55318	1,21	0,61	0	0	0,61	2				95245,69	1	1			1		110305,266	Mediator of RNA polymerase II transcription subunit 24 OS=Homo sapiens OX=9606 GN=MED24 PE=1 SV=1
1247	P49802|RGS7	58,33624	1,21	1,21	0	0	0	1					1	1					57668,34	Regulator of G-protein signaling 7 OS=Homo sapiens OX=9606 GN=RGS7 PE=1 SV=3
252	P57053|H2BF	306,803	61,9	29,37	23,02	29,37	61,9	9	117890,07	89813,125	87343,49	987559,8	1	6	5	7	15	Oxidation	13944,187	Histone H2B type F-S OS=Homo sapiens OX=9606 GN=H2BC12L PE=1 SV=2
438	P08754|GNA	232,2788	12,99	0	0	7,06	12,99	5					1			6	7		40532,188	Guanine nucleotide-binding protein G(i) subunit alpha-3 OS=Homo sapiens OX=9606 GN=GNAI3 PE=1 SV=3
1011	A0A087WUL8	41,75044	0,16	0	0	0,16	0,16	1			5235523	7,28E+07	1			2	4		440408,2	Neuroblastoma breakpoint family member 19 OS=Homo sapiens OX=9606 GN=NBPF19 PE=5 SV=1
1123	E9PAV3|NAC	23,45577	0,29	0	0	0,29	0	1			51930,484		1			1			205421,52	Nascent polypeptide-associated complex subunit alpha, muscle-specific form OS=Homo sapiens OX=9606 GN=NACA PE=1 SV=1
398	Q5JR12|PPM	32,09123	1,19	1,19	1,19	0	0	1	887625,8	83099,29			1	1	1				54834,277	Protein phosphatase 1J OS=Homo sapiens OX=9606 GN=PPM1J PE=1 SV=1
1248	Q9UIQ6|LCAI	36,25731	1,27	0	0	0	1,27	1				70910,414	1				1		117349,1	Leucyl-cystinyl aminopeptidase OS=Homo sapiens OX=9606 GN=LNPEP PE=1 SV=3
1178	Q01459|DIAC	53,57428	1,56	0	1,56	0	0	1		28655			1		1				43759,83	Di-N-acetylchitobiase OS=Homo sapiens OX=9606 GN=CTBS PE=1 SV=1
191	Q9Y4R8|TELC	93,05098	0,84	0	0	0	0,84	1					1				1		91747,02	Telomere length regulation protein TEL2 homolog OS=Homo sapiens OX=9606 GN=TELO2 PE=1 SV=2
1249	Q16880|CGT_	27,11062	1,11	0	0	1,11	0	1			55953,69		1			1			61437,74	2-hydroxyacylsphingosine 1-beta-galactosyltransferase OS=Homo sapiens OX=9606 GN=UGT8 PE=1 SV=2
1250	Q96KR7|PHA	38,33797	1,07	1,07	0	0	0	1	5895,04				1	1					62552,188	Phosphatase and actin regulator 3 OS=Homo sapiens OX=9606 GN=PHACTR3 PE=1 SV=1
725	P62191|PRS4	25,7136	1,36	0	0	0	1,36	1				1,48E+07	1				2	Acetylatic	49184,508	26S proteasome regulatory subunit 4 OS=Homo sapiens OX=9606 GN=PSMC1 PE=1 SV=1
250	Q9Y3F4|STRA	49,34548	1,71	0	0	0	1,71	1				673270,6	1				1	Deamidat	38438,31	Serine-threonine kinase receptor-associated protein OS=Homo sapiens OX=9606 GN=STRAP PE=1 SV=1
5	Q6ZQW0|I23	95,18884	1,43	1,43	1,43	0	0	1	7970265	233452,2			1	5	1				47075,156	Indoleamine 2,3-dioxygenase 2 OS=Homo sapiens OX=9606 GN=IDO2 PE=1 SV=4
1251	Q07021|C1QI	58,35568	3,9	3,9	0	0	0	1	11102,253				1	2					31362,236	Complement component 1 Q subcomponent-binding protein, mitochondrial OS=Homo sapiens OX=9606 GN=C1QBP PE=1 SV=1
174	Q96HY6|DDR	33,9707	1,91	0	0	1,91	0	1			235804,4		1			1			35610,863	DDRGK domain-containing protein 1 OS=Homo sapiens OX=9606 GN=DDRGK1 PE=1 SV=2
788	O00716|E2F3	29,62739	1,29	1,29	0	0	0	1	726644,5				1	1					49161,59	Transcription factor E2F3 OS=Homo sapiens OX=9606 GN=E2F3 PE=1 SV=1
413	Q71DI3|H32_	118,3275	23,53	4,41	4,41	5,15	23,53	5	340159,38	464861,53	729542	5116208	4	1	1	1	7	Oxidation	15388,0205	Histone H3.2 OS=Homo sapiens OX=9606 GN=H3C15 PE=1 SV=3
114	P12814|ACTN	426,4215	28,14	26,12	5,16	10,76	0,67	24	881217,94	65390,75	75049,11		11	41	9	11	1	Carbamid	103057,55	Alpha-actinin-1 OS=Homo sapiens OX=9606 GN=ACTN1 PE=1 SV=2
997	Q6ISU1|PTCR	35,18394	2,14	0	2,14	0	0	1					1		1				29265,564	Pre T-cell antigen receptor alpha OS=Homo sapiens OX=9606 GN=PTCRA PE=1 SV=1
1252	Q96N16|JKIP	42,81142	0,96	0	0	0	0,96	1				7724995,5	1				1		73209,15	Janus kinase and microtubule-interacting protein 1 OS=Homo sapiens OX=9606 GN=JAKMIP1 PE=1 SV=1
293	P08473|NEP_	256,9173	10,27	0	0	0	10,27	9				469876,94	8				10	Carbamid	85513,93	Neprilysin OS=Homo sapiens OX=9606 GN=MME PE=1 SV=2
6	Q58F21|BRD	76,21806	0,63	0,63	0	0,63	0,63	1	95524,04		1177229,9	711697,1	1	1		2	1		107954,484	Bromodomain testis-specific protein OS=Homo sapiens OX=9606 GN=BRDT PE=1 SV=4
90	Q13049|TRI3	111,9766	0,92	0,92	0	0,92	0,92	1	102637,01		798956,9	700055,2	1	1		1	1		71988,56	E3 ubiquitin-protein ligase TRIM32 OS=Homo sapiens OX=9606 GN=TRIM32 PE=1 SV=2
294	P07900|HS90	300,8226	11,07	8,61	0	9,02	3,83	9	34330,24				1	6		10	3		84659,71	Heat shock protein HSP 90-alpha OS=Homo sapiens OX=9606 GN=HSP90AA1 PE=1 SV=5
928	Q6IAA8|LTOF	178,6983	14,91	0	0	8,07	14,91	2			21508,006	177466,97	2			1	3		17744,818	Ragulator complex protein LAMTOR1 OS=Homo sapiens OX=9606 GN=LAMTOR1 PE=1 SV=2
109	P46734|MP2I	88,65927	1,73	1,73	1,73	0	0	1	1444307,8	950155,06			1	5	4				39318,383	Dual specificity mitogen-activated protein kinase kinase 3 OS=Homo sapiens OX=9606 GN=MAP2K3 PE=1 SV=2
1253	Q13740|CD1	62,91679	1,54	0	0	0	1,54	1				67790,93	1				1		65102,277	CD166 antigen OS=Homo sapiens OX=9606 GN=ALCAM PE=1 SV=2
1254	Q8IZT8|HS3S	61,65413	1,73	0	0	0	1,73	1				119894,2	1				1		40408,188	Heparan sulfate glucosamine 3-O-sulfotransferase 5 OS=Homo sapiens OX=9606 GN=HS3ST5 PE=1 SV=1
1255	P62750|RL23	118,9564	8,33	0	0	0	8,33	1					1				1		17695,059	60S ribosomal protein L23a OS=Homo sapiens OX=9606 GN=RPL23A PE=1 SV=1
476	Q9Y6L7|TLL2_	84,0716	1,28	1,28	0	0	0	1					1	1					113556,66	Tolloid-like protein 2 OS=Homo sapiens OX=9606 GN=TLL2 PE=1 SV=1
8	Q9NRL3|STRI	60,21952	0,8	0	0	0,8	0,8	1			1629398,8	554100,6	1			2	1		80596,09	Striatin-4 OS=Homo sapiens OX=9606 GN=STRN4 PE=1 SV=2
140	Q12805|FBLN	395,5523	31,85	31,85	0	0	0	17	7044180				16	44				Carbamid	54640,543	EGF-containing fibulin-like extracellular matrix protein 1 OS=Homo sapiens OX=9606 GN=EFEMP1 PE=1 SV=2
105	Q9UKZ9|PCO	110,4438	1,45	1,45	1,45	0	0	1	342002,44				1	2	1			Carbamid	45716,5	Procollagen C-endopeptidase enhancer 2 OS=Homo sapiens OX=9606 GN=PCOLCE2 PE=1 SV=1
1122	P48637|GSHE	29,25815	1,27	0	0	0	1,27	1				173536,34	1				1		52384,82	Glutathione synthetase OS=Homo sapiens OX=9606 GN=GSS PE=1 SV=1
40	Q7Z794|K2C1	466,0054	32,35	7,44	7,27	22,66	28,89	37	143481,38		1257391,8	1092180,8	14	12	24	165	116	Acetylatic	61901,41	Keratin, type II cytoskeletal 1b OS=Homo sapiens OX=9606 GN=KRT77 PE=1 SV=3
578	Q16473|TEN	42,43897	2,89	0	0	2,89	0	1			31635,004		1			1			33739,727	Putative tenascin-XA OS=Homo sapiens OX=9606 GN=TNXA PE=5 SV=2
325	Q14108|SCRE	300,2538	12,97	0	0	11,3	12,97	7			821393	3007573	7			8	12	Carbamid	54290,156	Lysosome membrane protein 2 OS=Homo sapiens OX=9606 GN=SCARB2 PE=1 SV=2
73	P35579|MYH	454,325	15,51	5,36	0,51	13,52	0,31	33	942454,4	84900,57	900570,56	28043,12	27	15	1	36	1	Carbamid	226532,23	Myosin-9 OS=Homo sapiens OX=9606 GN=MYH9 PE=1 SV=4
1256	Q6NUT2|D19	26,36875	0,79	0,79	0,79	0	0	1	49681,99	46054,62			1	1	1				87373,805	Probable C-mannosyltransferase DPY19L2 OS=Homo sapiens OX=9606 GN=DPY19L2 PE=1 SV=2
109	Q9Y2F5|ICE1	88,65927	0,26	0,26	0,26	0	0	1	1444307,8	950155,06			1	5	4				247890,78	Little elongation complex subunit 1 OS=Homo sapiens OX=9606 GN=ICE1 PE=1 SV=5
95	P55196|AFAD	106,0132	0,33	0,33	0,33	0,33	0,33	1	2927749	1123188,2	254597,66	341997,72	1	4	3	1	1		206804,22	Afadin OS=Homo sapiens OX=9606 GN=AFDN PE=1 SV=3
660	Q15058|KIF1	25,21471	0,36	0	0	0,36	0	1			89165,93		1			1			186491,58	Kinesin-like protein KIF14 OS=Homo sapiens OX=9606 GN=KIF14 PE=1 SV=1
929	Q9Y696|CLIC	49,99641	6,72	0	0	6,72	2,37	2			166445,72		1			3	1		28772,102	Chloride intracellular channel protein 4 OS=Homo sapiens OX=9606 GN=CLIC4 PE=1 SV=4
1257	A5D8V6|VP3	51,09072	2,25	0	0	0	2,25	1				61790,504	1				1		38658,957	Vacuolar protein sorting-associated protein 37C OS=Homo sapiens OX=9606 GN=VPS37C PE=1 SV=2
150	F5GYI3|UBA1	45,67938	1,57	0	1,57	0	0	1					1		1				40591,902	Ubiquitin-associated protein 1-like OS=Homo sapiens OX=9606 GN=UBAP1L PE=2 SV=1
1258	P05386|RLA1	26,51783	8,77	0	8,77	0	0	1		28662,662			1		1				11513,932	60S acidic ribosomal protein P1 OS=Homo sapiens OX=9606 GN=RPLP1 PE=1 SV=1
83	Q13615|MTN	78,4077	0,5	0,5	0,5	0	0	1	207145,14	1469270,9			1	1	3				133618,92	Myotubularin-related protein 3 OS=Homo sapiens OX=9606 GN=MTMR3 PE=1 SV=3
850	P28838|AMP	33,86671	1,16	1,16	0	0	0	1	2162869				1	2					56166,344	Cytosol aminopeptidase OS=Homo sapiens OX=9606 GN=LAP3 PE=1 SV=3
1003	P10827|THA_	57,74165	1,43	0	0	1,43	1,43	1			1136429,6	1072534	1			4	4		54815,527	Thyroid hormone receptor alpha OS=Homo sapiens OX=9606 GN=THRA PE=1 SV=1
618	Q92484|ASM	146,2699	5,52	0	5,52	0	0	3		254561,9			3		3				51260,4	Acid sphingomyelinase-like phosphodiesterase 3a OS=Homo sapiens OX=9606 GN=SMPDL3A PE=1 SV=2
1259	Q96EV2|RBM	29,31694	0,51	0	0	0	0,51	1				1064834,6	1				1		129985,9	RNA-binding protein 33 OS=Homo sapiens OX=9606 GN=RBM33 PE=1 SV=3
376	Q9BTM1|H2A	279,6636	37,98	11,63	12,4	6,98	37,98	6	259248,44	294746,4	250080,11	2714422,5	6	4	6	2	11		14019,405	Histone H2A.J OS=Homo sapiens OX=9606 GN=H2AJ PE=1 SV=1
1260	P43004|EAA2	26,60586	1,05	1,05	0	0	0	1	283890,62				1	1					62104,234	Excitatory amino acid transporter 2 OS=Homo sapiens OX=9606 GN=SLC1A2 PE=1 SV=2
1061	Q9UMR2|DD	28,85608	1,25	0	0	1,25	0	1			67875,92		1			1			53926,85	ATP-dependent RNA helicase DDX19B OS=Homo sapiens OX=9606 GN=DDX19B PE=1 SV=1
326	P22528|SPR1	316,8506	61,8	0	0	61,8	59,55	8			190087,2	161168,7	1			27	14	Carbamid	9887,557	Cornifin-B OS=Homo sapiens OX=9606 GN=SPRR1B PE=1 SV=2
27	Q8IWT6|LRC	82,94455	0,86	0,86	0,86	0,86	0,86	1		188288,19		237893,3	1	2	3	1	2		94198,734	Volume-regulated anion channel subunit LRRC8A OS=Homo sapiens OX=9606 GN=LRRC8A PE=1 SV=1
1261	P04156|PRIO	74,74937	3,56	3,56	3,56	0	0	1	247014,08	58810,21			1	2	2				27661,162	Major prion protein OS=Homo sapiens OX=9606 GN=PRNP PE=1 SV=1
428	Q96LJ7|DHRS	70,07965	2,24	2,24	0	0	0	1	250109,83				1	2					33909,02	Dehydrogenase/reductase SDR family member 1 OS=Homo sapiens OX=9606 GN=DHRS1 PE=1 SV=1
503	Q08722|CD4	150,2468	9,6	0	0	0	9,6	4				238546,25	4				4		35213,637	Leukocyte surface antigen CD47 OS=Homo sapiens OX=9606 GN=CD47 PE=1 SV=1
1262	A5PKW4|PSD	25,22808	0,78	0,78	0	0	0	1					1	1				Acetylatic	109542,8	PH and SEC7 domain-containing protein 1 OS=Homo sapiens OX=9606 GN=PSD PE=1 SV=2
930	P23246|SFPQ	45,50363	1,7	0,85	0,85	0	0	2		37245,836			1	1	1				76149,34	Splicing factor, proline- and glutamine-rich OS=Homo sapiens OX=9606 GN=SFPQ PE=1 SV=2
68	Q96BZ8|LEN	67,74845	2,27	0	2,27	0	0	1		549368,8			1		2				30528,127	Leukocyte receptor cluster member 1 OS=Homo sapiens OX=9606 GN=LENG1 PE=1 SV=2
22	P12110|CO6	552,685	45,93	38,17	23,95	32,09	38,57	70	3,93E+07	1,75E+07	5,43E+07	5,19E+08	59	118	124	142	278	Carbamid	108579,42	Collagen alpha-2(VI) chain OS=Homo sapiens OX=9606 GN=COL6A2 PE=1 SV=4
1263	Q7L4E1|MIG	24,32171	1,01	0	0	1,01	0	1			29140,309		1			1			65530,83	Mitoguardin 2 OS=Homo sapiens OX=9606 GN=MIGA2 PE=1 SV=1

619	Q99590|SCAF	62,39521	1,44	0,62	0	0,62	0,82	3			662663,06		1	1		1	3		164652,25	Protein SCAF11 OS=Homo sapiens OX=9606 GN=SCAF11 PE=1 SV=2
1129	P39023|RL3_	43,35169	1,74	0	0	0	1,74	1				33642,63	1				1		46108,887	60S ribosomal protein L3 OS=Homo sapiens OX=9606 GN=RPL3 PE=1 SV=2
141	Q562R1|ACTE	352,7626	19,68	12,77	3,99	17,02	19,41	12				3307931,8	1	15	4	52	27	Carbamid	42003,164	Beta-actin-like protein 2 OS=Homo sapiens OX=9606 GN=ACTBL2 PE=1 SV=2
996	Q8WWN8|AI	80,99365	0,39	0,39	0,39	0,39	0,39	1	6230665,5	2880302,8	1864814,6	5602360,5	1	1	1	1	2		169844,25	Arf-GAP with Rho-GAP domain, ANK repeat and PH domain-containing protein 3 OS=Homo sapiens OX=9606 GN=ARAP3 PE=1 SV=1
932	P25788|PSA3	61,90135	5,1	0	0	5,1	2,75	2			58870,77	42917,85	2			2	1		28433,223	Proteasome subunit alpha type-3 OS=Homo sapiens OX=9606 GN=PSMA3 PE=1 SV=2
933	P32780|TF2H	40,5717	2,19	0	0	2,19	0	2			27898,795		1			2			62031,598	General transcription factor IIH subunit 1 OS=Homo sapiens OX=9606 GN=GTF2H1 PE=1 SV=1
11	P07996|TSP1	587,7539	65,98	56,5	47,35	57,35	60,6	82	1,01E+08	4,59E+07	7,42E+07	1,78E+08	74	291	218	211	273	Carbamid	129382,664	Thrombospondin-1 OS=Homo sapiens OX=9606 GN=THBS1 PE=1 SV=2
205	Q4ZHG4|FND	316,7295	6,23	6,23	0	0	0	13	1498810,4				12	16					205557,9	Fibronectin type III domain-containing protein 1 OS=Homo sapiens OX=9606 GN=FNDC1 PE=2 SV=4
3	B3KU38|IQIP	74,84779	1,42	0	0	1,42	1,24	2			38447,664		2			3	1		62247,953	IQCJ-SCHIP1 readthrough transcript protein OS=Homo sapiens OX=9606 GN=IQCJ-SCHIP1 PE=1 SV=2
935	P47756|CAP2	38,98536	5,15	0	0	5,15	0	2			59878,3		2			2			30628,73	F-actin-capping protein subunit beta OS=Homo sapiens OX=9606 GN=CAPZB PE=1 SV=5
235	P01374|TNFB	85,95554	2,93	2,93	2,93	0	0	1	164047,08	165661,67			1	1	1				22296,756	Lymphotoxin-alpha OS=Homo sapiens OX=9606 GN=LTA PE=1 SV=2
244	Q14766|LTBP	327,7881	5,64	5,64	0,35	0	0	11	802119,8				9	14	1			Carbamid	186796,1	Latent-transforming growth factor beta-binding protein 1 OS=Homo sapiens OX=9606 GN=LTBP1 PE=1 SV=4
3	Q9P2G1|AKIB	41,04887	0,55	0	0,55	0	0	1		31847,021			1		1			Deamidat	122001,56	Ankyrin repeat and IBR domain-containing protein 1 OS=Homo sapiens OX=9606 GN=ANKIB1 PE=1 SV=3
365	Q8NHP8|PLB	271,9378	11,04	11,04	1,53	0	0	7	799880,8	52199,26			7	10	1			Carbamid	65471,83	Putative phospholipase B-like 2 OS=Homo sapiens OX=9606 GN=PLBD2 PE=1 SV=2
94	Q13835|PKP1	460,0164	36,14	0,8	0,8	26,51	29,59	28			2162075,8	2253878	27	2	1	28	29	Carbamid	82860,555	Plakophilin-1 OS=Homo sapiens OX=9606 GN=PKP1 PE=1 SV=2
540	Q9H511|KLH	37,4525	0,95	0	0	0	0,95	1				22246,697	1				1		70246,086	Kelch-like protein 31 OS=Homo sapiens OX=9606 GN=KLHL31 PE=2 SV=1
327	P0DOX5|IGG	309,056	16,48	0	0	16,48	1,78	8			680663		4			15	1	Carbamid	49328,797	Immunoglobulin gamma-1 heavy chain OS=Homo sapiens OX=9606 PE=1 SV=2
1264	P61313|RL15	48,19709	4,41	0	0	0	4,41	1				111132,9	1				1		24146,068	60S ribosomal protein L15 OS=Homo sapiens OX=9606 GN=RPL15 PE=1 SV=2
249	P62987|RL40	312,1252	47,66	47,66	40,62	47,66	47,66	8	7896435,5	3367563,2	1,28E+07	5,91E+07	8	34	29	35	49	Oxidation	14728,311	Ubiquitin-60S ribosomal protein L40 OS=Homo sapiens OX=9606 GN=UBA52 PE=1 SV=2
616	Q52LJ0|FA98	45,10995	1,39	0	0	1,39	0	1			52744,816		1			1		Deamidat	45547,19	Protein FAM98B OS=Homo sapiens OX=9606 GN=FAM98B PE=1 SV=2
681	Q96LW2|KS6	51,85208	1,46	0	0	1,46	0	1			116246,125		1			1			46191,305	Ribosomal protein S6 kinase-related protein OS=Homo sapiens OX=9606 GN=RSKR PE=1 SV=2
71	P15144|AMP	457,3065	29,58	16,75	13,44	24,51	16,96	28	1429644,9	1153735	6276485,5	7332226	27	21	18	40	36	Carbamid	109539,59	Aminopeptidase N OS=Homo sapiens OX=9606 GN=ANPEP PE=1 SV=4
504	Q4G0P3|HYD	129,8326	0,47	0,12	0,12	0,35	0	4			759095		2	1	2	5		Deamidat	575892,1	Hydrocephalus-inducing protein homolog OS=Homo sapiens OX=9606 GN=HYDIN PE=1 SV=3
252	P06899|H2B1	299,3754	59,52	34,13	20,63	29,37	59,52	9	117890,07	89813,125	87343,49	987559,8	1	7	6	7	15	Oxidation	13904,162	Histone H2B type 1-J OS=Homo sapiens OX=9606 GN=H2BC11 PE=1 SV=3
926	Q9P225|DYH	24,46977	0,14	0	0	0	0,14	1				95245,69	1				1		507698,2	Dynein axonemal heavy chain 2 OS=Homo sapiens OX=9606 GN=DNAH2 PE=1 SV=3
505	P61586|RHO	100,3581	13,47	0	0	13,47	9,33	4			378677,8	340597,66	3			5	3		21768,123	Transforming protein RhoA OS=Homo sapiens OX=9606 GN=RHOA PE=1 SV=1
439	P36543|VATE	146,3287	16,37	2,65	0	2,65	13,72	5				159018,78	4	1		1	4	Acetylatic	26145,3	V-type proton ATPase subunit E 1 OS=Homo sapiens OX=9606 GN=ATP6V1E1 PE=1 SV=1
328	P02792|FRIL	279,2653	35,43	3,43	3,43	28,57	32	8	517809,8		1466424,6	1007456,44	8	1	1	9	9		20019,67	Ferritin light chain OS=Homo sapiens OX=9606 GN=FTL PE=1 SV=2
146	Q9BWH6|RP	27,22116	0,43	0	0	0	0,43	1				29655,746	1				1	Deamidat	152754,72	RNA polymerase II-associated protein 1 OS=Homo sapiens OX=9606 GN=RPAP1 PE=1 SV=3
693	Q5T5J6|SWT	62,80522	0,67	0	0	0,67	0	1			57405,086		1			1			103221,58	Transcriptional protein SWT1 OS=Homo sapiens OX=9606 GN=SWT1 PE=2 SV=1
271	P07711|CATL	304,2528	24,62	22,82	20,42	0	5,71	8	3545433	2701208		29421,637	7	21	28		2	Carbamid	37564,125	Procathepsin L OS=Homo sapiens OX=9606 GN=CTSL PE=1 SV=2
1265	Q96EB6|SIR1	26,32761	0,8	0	0	0,8	0	1			608259,8		1			1			81681	NAD-dependent protein deacetylase sirtuin-1 OS=Homo sapiens OX=9606 GN=SIRT1 PE=1 SV=2
1042	Q6ZU69|S31F	32,33427	0,45	0	0	0,45	0	1			62760,535		1			1			148095,98	Protein SPATA31F1 OS=Homo sapiens OX=9606 GN=SPATA31F1 PE=2 SV=4
596	Q5XKP0|MIC	31,85088	5,08	0	0	5,08	0	1			3721363,8		1			3			13087,245	MICOS complex subunit MIC13 OS=Homo sapiens OX=9606 GN=MICOS13 PE=1 SV=1
903	P30038|AL4A	50,07074	1,07	0	1,07	0	0	1		22226,035			1		1				61719,465	Delta-1-pyrroline-5-carboxylate dehydrogenase, mitochondrial OS=Homo sapiens OX=9606 GN=ALDH4A1 PE=1 SV=3
1266	Q6UWF7|NX	55,33417	1,1	0	1,1	0	0	1		68730,03			1		1				62262,887	NXPE family member 4 OS=Homo sapiens OX=9606 GN=NXPE4 PE=2 SV=1
204	Q6NUK4|REE	42,55261	2,35	0	0	0	2,35	1				226608,28	1				1		29263,902	Receptor expression-enhancing protein 3 OS=Homo sapiens OX=9606 GN=REEP3 PE=1 SV=1
78	Q6YHK3|CD1	433,808	14,33	13,08	10,93	0	1,73	28	3166910,2	1478795,2		51556,223	23	44	37		3	Deamidat	161689,08	CD109 antigen OS=Homo sapiens OX=9606 GN=CD109 PE=1 SV=2
506	Q08752|PPID	168,6462	4,86	3,24	4,86	3,24	3,24	3		198834,83			1	2	3	3	2	Acetylatic	40763,574	Peptidyl-prolyl cis-trans isomerase D OS=Homo sapiens OX=9606 GN=PPID PE=1 SV=3
252	Q6DN03|H2B	161,9766	9,84	9,84	9,84	6,74	9,84	3	117890,07	89813,125	87343,49	987559,8	1	3	4	2	5		21472,045	Putative histone H2B type 2-C OS=Homo sapiens OX=9606 GN=H2BC20P PE=5 SV=3
221	Q9HC29|NOI	86,38369	0,67	0	0	0,67	0,67	1			268741,34	254677,05	1			1	1		115282,914	Nucleotide-binding oligomerization domain-containing protein 2 OS=Homo sapiens OX=9606 GN=NOD2 PE=1 SV=1
1059	Q6ZN18|AEB	26,05076	1,16	0	0	1,16	1,16	1			28698,484	80936,39	1			1	1		54466,55	Zinc finger protein AEBP2 OS=Homo sapiens OX=9606 GN=AEBP2 PE=1 SV=2
222	Q96NW7|LRR	86,95845	0,39	0	0	0,39	0	1			161786,6		1			1		Carbamid	172580,75	Leucine-rich repeat-containing protein 7 OS=Homo sapiens OX=9606 GN=LRRC7 PE=1 SV=1
1267	Q02487|DSC	25,83116	0,67	0,67	0	0	0	1					1	1					99961,68	Desmocollin-2 OS=Homo sapiens OX=9606 GN=DSC2 PE=1 SV=1
1268	P19525|E2AK	33,14779	1,09	0	0	1,09	0	1					1			1			62094,258	Interferon-induced, double-stranded RNA-activated protein kinase OS=Homo sapiens OX=9606 GN=EIF2AK2 PE=1 SV=2
620	P61421|VA0	218,6306	9,69	0	0	0	9,69	3				190711,52	3				4		40329,094	V-type proton ATPase subunit d 1 OS=Homo sapiens OX=9606 GN=ATP6V0D1 PE=1 SV=1
400	Q969P0|IGSF	222,4849	7,18	1,14	0,98	0,98	7,18	6	191851,17			546397,1	5	4	2	2	9		65034,24	Immunoglobulin superfamily member 8 OS=Homo sapiens OX=9606 GN=IGSF8 PE=1 SV=1
1022	Q8NHY3|GA2	28,43442	0,68	0	0,68	0	0	1		122104,8			1		1				96519,84	GAS2-like protein 2 OS=Homo sapiens OX=9606 GN=GAS2L2 PE=1 SV=1
1269	O43818|U3IP	26,62863	1,26	0	0	1,26	0	1			23824,734		1			1			51840,652	U3 small nucleolar RNA-interacting protein 2 OS=Homo sapiens OX=9606 GN=RRP9 PE=1 SV=1
621	P60981|DEST	162,3148	14,55	14,55	3,64	4,24	4,24	3					1	3	1	1	1		18505,719	Destrin OS=Homo sapiens OX=9606 GN=DSTN PE=1 SV=3
123	Q86UU5|GGN	29,86649	0,92	0	0,92	0	0	1		253011,45			1		1				66698,516	Gametogenetin OS=Homo sapiens OX=9606 GN=GGN PE=1 SV=2
622	Q86UP3|ZFH	54,52673	0,53	0	0,17	0,17	0,2	3				2424746,8	2		1	1	1		393729,7	Zinc finger homeobox protein 4 OS=Homo sapiens OX=9606 GN=ZFHX4 PE=1 SV=1
18	P48668|K2C6	577,5061	74,11	21,63	24,65	74,11	62,77	81			73430,8	119093,375	2	25	50	472	297	Acetylatic	60024,953	Keratin, type II cytoskeletal 6C OS=Homo sapiens OX=9606 GN=KRT6C PE=1 SV=3
186	Q92674|CENI	97,96496	0,79	0,79	0,79	0,79	0,79	1	404544,56	132516,72	213150,33	123433,66	1	1	2	1	1		86720,414	Centromere protein I OS=Homo sapiens OX=9606 GN=CENPI PE=1 SV=2
14	A6NJZ7|RIM	100,3808	0,37	0,37	0,37	0	0,37	1	688577,7			23365,338	1	2	1		1	Carbamid	180949,72	RIMS-binding protein 3C OS=Homo sapiens OX=9606 GN=RIMBP3C PE=1 SV=3
507	O14686|KMT	167,1178	0,43	0,22	0,33	0,22	0,43	4			660523,25	1253976	1	7	9	2	5		593389,4	Histone-lysine N-methyltransferase 2D OS=Homo sapiens OX=9606 GN=KMT2D PE=1 SV=2
211	A4D1S5|RAB	98,04495	2,76	2,76	0	0	0	1	66990,9				1	1				Carbamid	24399,844	Ras-related protein Rab-19 OS=Homo sapiens OX=9606 GN=RAB19 PE=1 SV=2
823	Q9Y6N9|USH	23,2552	1,09	0	1,09	0	0	1		140092,28			1		1				62210,84	Harmonin OS=Homo sapiens OX=9606 GN=USH1C PE=1 SV=3
1270	P03973|SLPI	50,99022	5,3	0	0	5,3	5,3	1			68312,3	69324,89	1			1	1	Carbamid	14326,083	Antileukoproteinase OS=Homo sapiens OX=9606 GN=SLPI PE=1 SV=2
508	Q14574|DSC	180,9029	3,46	0	0	1	3,46	4			105186,8	603395,75	4			1	4		99968,984	Desmocollin-3 OS=Homo sapiens OX=9606 GN=DSC3 PE=1 SV=3
1271	Q8TF61|FBX4	48,55804	0,69	0	0,69	0	0	1		38690,793			1		1				94496,34	F-box only protein 41 OS=Homo sapiens OX=9606 GN=FBXO41 PE=2 SV=5
26	Q8N6M6|AM	25,02506	0,73	0	0	0,73	0	1			56336,875		1			1			93572,41	Aminopeptidase O OS=Homo sapiens OX=9606 GN=AOPEP PE=1 SV=2
243	Q96G03|PGM	80,71943	0,98	0,98	0	0	0	1	337568,3				1	1					68283,39	Phosphopentomutase OS=Homo sapiens OX=9606 GN=PGM2 PE=1 SV=4
84	O95631|NET	27,33654	0,99	0	0	0	0,99	1				98403,8	1				1		67748,28	Netrin-1 OS=Homo sapiens OX=9606 GN=NTN1 PE=1 SV=2
230	Q8IWW6|RH	28,61401	0,83	0	0	0,83	0	1			47442,062		1			1			96254,43	Rho GTPase-activating protein 12 OS=Homo sapiens OX=9606 GN=ARHGAP12 PE=1 SV=1
940	P49448|DHE	49,11904	3,41	0	3,41	0	0	2					2		2				61433,996	Glutamate dehydrogenase 2, mitochondrial OS=Homo sapiens OX=9606 GN=GLUD2 PE=1 SV=2
148	Q92813|IOD2	33,4275	2,2	2,2	0	0	0	1	151675,5				1	1					30489,45	Type II iodothyronine deiodinase OS=Homo sapiens OX=9606 GN=DIO2 PE=1 SV=4
87	P42566|EPS1	100,6363	0,67	0,67	0	0,67	0,67	1	112304,12			279099,3	1	1		1	1		98655,74	Epidermal growth factor receptor substrate 15 OS=Homo sapiens OX=9606 GN=EPS15 PE=1 SV=2
1272	P23396|RS3_	35,25897	5,35	0	0	0	5,35	1				27906,203	1				1		26688,31	40S ribosomal protein S3 OS=Homo sapiens OX=9606 GN=RPS3 PE=1 SV=2
142	Q8N103|TAG	78,41393	0,82	0,82	0,82	0,82	0,82	1				274587,2	1	1	1	1	1		80703	T-cell activation Rho GTPase-activating protein OS=Homo sapiens OX=9606 GN=TAGAP PE=1 SV=1
7	P34932|HSP7	29,77415	0,71	0	0	0,71	0	1					1			1			94330,914	Heat shock 70 kDa protein 4 OS=Homo sapiens OX=9606 GN=HSPA4 PE=1 SV=4
660	O43896|KIF1	25,21471	0,54	0	0	0,54	0	1			89165,93		1			1			122946,55	Kinesin-like protein KIF1C OS=Homo sapiens OX=9606 GN=KIF1C PE=1 SV=3
509	P29034|S10A	168,5659	25,51	0	0	25,51	0	4			771697,94		4			7		Carbamid	11116,761	Protein S100-A2 OS=Homo sapiens OX=9606 GN=S100A2 PE=1 SV=3
441	Q96CG8|CTH	247,2962	18,93	18,93	10,7	0	0	5	4749519,5	126453,31			4	12	6			Carbamid	26223,947	Collagen triple helix repeat-containing protein 1 OS=Homo sapiens OX=9606 GN=CTHRC1 PE=1 SV=1
272	Q06830|PRD	346,787	49,25	41,21	15,08	45,23	36,68	10	942425,06		2139034	1071372,1	9	16	3	16	10		22110,352	Peroxiredoxin-1 OS=Homo sapiens OX=9606 GN=PRDX1 PE=1 SV=1
51	Q9Y2D9|ZN6	52,12899	1,16	0	0	0	1,16	1				22094,525	1				1		69744,086	Zinc finger protein 652 OS=Homo sapiens OX=9606 GN=ZNF652 PE=1 SV=3
510	P08648|ITA5	99,50215	4,48	0	0	0	4,48	4				105622,86	4				5		114536,49	Integrin alpha-5 OS=Homo sapiens OX=9606 GN=ITGA5 PE=1 SV=2
1273	P30825|CTR1	81,56643	1,59	0	0	0	1,59	1				53273,88	1				1	Carbamid	67638,37	High affinity cationic amino acid transporter 1 OS=Homo sapiens OX=9606 GN=SLC7A1 PE=1 SV=1
942	Q6ZTQ4|CDH	65,12635	1,36	0	0,68	0,68	0	2			65454,918		1		1	1			97976,96	Cadherin-related family member 3 OS=Homo sapiens OX=9606 GN=CDHR3 PE=1 SV=1
273	P08238|HS90	312,5891	13,26	8,7	0	10,64	6,08	10			48131,22		2	6		10	5		83264,195	Heat shock protein HSP 90-beta OS=Homo sapiens OX=9606 GN=HSP90AB1 PE=1 SV=4
1	Q86YV5|PRA	38,93573	0,5	0	0	0	0,5	1				280077,25	1				1		149623,56	Inactive tyrosine-protein kinase PRAG1 OS=Homo sapiens OX=9606 GN=PRAG1 PE=1 SV=4
274	P17661|DESM	252,9915	8,51	6,81	8,51	6,81	6,81	6					1	13	12	8	8	Deamidat	53535,785	Desmin OS=Homo sapiens OX=9606 GN=DES PE=1 SV=3
1274	Q92874|DNS	59,1357	2,01	0	0	0	2,01	1				159360,53	1				1		32853,2	Deoxyribonuclease-1-like 2 OS=Homo sapiens OX=9606 GN=DNASE1L2 PE=1 SV=1
5	Q8N1G0|ZN6	95,18884	0,49	0,49	0,49	0	0	1	7970265	233452,2			1	5	1				129528,65	Zinc finger protein 687 OS=Homo sapiens OX=9606 GN=ZNF687 PE=1 SV=1
316	P0DP25|CALM	295,4051	41,61	38,93	30,87	33,56	30,87	7	5591565,5	2249549,2	2484052,8	2222430,5	7	13	11	10	10	Acetylatic	16837,582	Calmodulin-3 OS=Homo sapiens OX=9606 GN=CALM3 PE=1 SV=1
394	Q5T4B2|GT2	60,23641	1,01	1,01	0	0	0	1	151075				1	2					67591,84	Inactive glycosyltransferase 25 family member 3 OS=Homo sapiens OX=9606 GN=CERCAM PE=1 SV=1
148	G9CGD6|CNI	42,83809	0,67	0,67	0	0	0	1	70875,31				1	1					100364,836	CNK3/IPCEF1 fusion protein OS=Homo sapiens OX=9606 GN=CNK3/IPCEF1 PE=1 SV=1
275	P48740|MAS	336,5855	15,45	12,59	15,45	0	9,16	10	945780,44	6910987		242062,66	9	12	35		6	Carbamid	79246,664	Mannan-binding lectin serine protease 1 OS=Homo sapiens OX=9606 GN=MASP1 PE=1 SV=3
567	Q9BXB4|OSB	45,91409	0,8	0	0	0	0,8	1				103208,62	1				1	Acetylatic	83643,25	Oxysterol-binding protein-related protein 11 OS=Homo sapiens OX=9606 GN=OSBPL11 PE=1 SV=2
943	P04233|HG2	43,28749	4,05	2,03	0	2,03	0	2	26535,736				1	1		1			33515,586	HLA class II histocompatibility antigen gamma chain OS=Homo sapiens OX=9606 GN=CD74 PE=1 SV=3
1275	P09211|GSTP	44,03919	5,24	5,24	0	0	0	1	3597,4358				1	3					23355,826	Glutathione S-transferase P OS=Homo sapiens OX=9606 GN=GSTP1 PE=1 SV=2
681	Q12906|ILF3_	31,66304	0,78	0	0	0	0,78	1					1				1		95338,37	Interleukin enhancer-binding factor 3 OS=Homo sapiens OX=9606 GN=ILF3 PE=1 SV=3
131	Q86TI0|TBCD	26,63197	0,51	0	0	0,51	0	1					1			1			133083,98	TBC1 domain family member 1 OS=Homo sapiens OX=9606 GN=TBC1D1 PE=1 SV=2
34	O95479|G6PE	148,1728	0,88	0,88	0,88	0,76	0,76	2	427831,3	83577,14			1	5	3	2	2		88892,68	GDH/6PGL endoplasmic bifunctional protein OS=Homo sapiens OX=9606 GN=H6PD PE=1 SV=2
89	Q8TAM6|ERN	114,708	3,17	3,17	3,17	0	0	1	145734,8	4900,202			1	2	1				32782,94	Ermin OS=Homo sapiens OX=9606 GN=ERMN PE=1 SV=1
70	Q59EK9|RUN	66,2057	1,35	1,35	0	0	0	1	56448,73				1	1				Deamidat	49747,04	RUN domain-containing protein 3A OS=Homo sapiens OX=9606 GN=RUNDC3A PE=1 SV=2
948	Q99700|ATX	47,62974	0,91	0	0	0	0,91	2				53464,4	1				2		140282,81	Ataxin-2 OS=Homo sapiens OX=9606 GN=ATXN2 PE=1 SV=2
1276	Q7RTM1|OTC	34,75746	0,98	0	0	0	0,98	1				204391,92	1				1		67353,484	Proton channel OTOP1 OS=Homo sapiens OX=9606 GN=OTOP1 PE=2 SV=1
630	P56703|WNT	50,61808	1,69	0	0	1,69	1,69	1			116166,08	198165,22	1			1	1		39645,117	Proto-oncogene Wnt-3 OS=Homo sapiens OX=9606 GN=WNT3 PE=1 SV=2
19	P02533|K1C1	549,7058	80,08	22,46	31,78	80,08	77,33	70	158208,17	129083,42	1,00E+08	5,44E+07	19	22	38	484	287	Oxidation	51561,465	Keratin, type I cytoskeletal 14 OS=Homo sapiens OX=9606 GN=KRT14 PE=1 SV=4
3	Q9H0H0|INT	74,42973	0,5	0	0	0,5	0,5	1			1378542,6	1230783,9	1			3	2		134323,3	Integrator complex subunit 2 OS=Homo sapiens OX=9606 GN=INTS2 PE=1 SV=2
46	P60709|ACTE	469,6543	58,4	52,53	27,73	55,2	48,27	31	1230912	2542,2307	1927608,8	317536,9	1	65	16	140	53	Carbamid	41736,73	Actin, cytoplasmic 1 OS=Homo sapiens OX=9606 GN=ACTB PE=1 SV=1

88	P36955|PEDF	427,4649	49,52	49,52	33,49	9,81	6,22	24	7,31E+07	1,34E+07	161519,03	199324,62	23	129	90	6	4	Deamidat	46312,203	Pigment epithelium-derived factor OS=Homo sapiens OX=9606 GN=SERPINF1 PE=1 SV=4
1277	Q9Y6M5|ZNT	29,70958	1,97	0	0	0	1,97	1					1				1		55299,81	Proton-coupled zinc antiporter SLC30A1 OS=Homo sapiens OX=9606 GN=SLC30A1 PE=1 SV=3
305	Q8WUM9|S2	33,78748	0,88	0,88	0	0	0	1	253790,36				1	3					73699,67	Sodium-dependent phosphate transporter 1 OS=Homo sapiens OX=9606 GN=SLC20A1 PE=1 SV=1
567	Q5T6S3|PHF1	45,91409	1,03	0	0	0	1,03	1				103208,62	1				1	Acetylatic	65590,914	PHD finger protein 19 OS=Homo sapiens OX=9606 GN=PHF19 PE=1 SV=1
1115	O00329|PK3C	36,93215	0,57	0	0	0,57	0	1			259379,88		1			1		Deamidat	119479,13	Phosphatidylinositol 4,5-bisphosphate 3-kinase catalytic subunit delta isoform OS=Homo sapiens OX=9606 GN=PIK3CD PE=1 SV=2
1158	Q96AA8|JKIP	36,17757	0,86	0	0	0,86	0	1					1			1			94933,84	Janus kinase and microtubule-interacting protein 2 OS=Homo sapiens OX=9606 GN=JAKMIP2 PE=1 SV=1
949	P51148|RAB5	109,8108	8,33	0	0	0	8,33	2				91993,33	1				2		23482,553	Ras-related protein Rab-5C OS=Homo sapiens OX=9606 GN=RAB5C PE=1 SV=2
1063	P0CG00|ZSA5	24,44333	1,21	0	0	1,21	0	1			245290,78		1			1			56243,86	Putative zinc finger and SCAN domain-containing protein 5D OS=Homo sapiens OX=9606 GN=ZSCAN5DP PE=5 SV=1
1040	Q14392|LRC3	23,64662	0,91	0	0	0	0,91	1				91289,55	1				1		71979,055	Transforming growth factor beta activator LRRC32 OS=Homo sapiens OX=9606 GN=LRRC32 PE=1 SV=1
1179	Q7Z5R6|AB1I	37,06379	0,9	0,9	0	0	0	1	7278510,5				1	3					73182,67	Amyloid beta A4 precursor protein-binding family B member 1-interacting protein OS=Homo sapiens OX=9606 GN=APBB1IP PE=1 SV=1
246	P43235|CATK	328,4853	36,17	33,13	20,36	0	6,38	11	2233977,5	1400882,8		105267,88	10	18	18		3	Carbamid	36966,008	Cathepsin K OS=Homo sapiens OX=9606 GN=CTSK PE=1 SV=1
376	Q93077|H2A1	279,6636	37,69	11,54	12,31	6,92	37,69	6	259248,44	294746,4	250080,11	2714422,5	6	4	6	2	11		14105,464	Histone H2A type 1-C OS=Homo sapiens OX=9606 GN=H2AC6 PE=1 SV=3
901	F8W1W9|NP	32,753	1,4	1,4	0	0	0	1	228840,3				1	1				Deamidat	49181,086	Nuclear pore complex-interacting protein family member B9 OS=Homo sapiens OX=9606 GN=NPIPB9 PE=3 SV=1
1278	Q9NZR1|TMC	23,60683	1,99	0	0	0	1,99	1				505115,1	1				1		39595,043	Tropomodulin-2 OS=Homo sapiens OX=9606 GN=TMOD2 PE=1 SV=1
10	Q8TDM6|DLG	93,49783	0,36	0	0	0,36	0,36	1			1,23E+07	1,26E+07	1			4	3		213867,6	Disks large homolog 5 OS=Homo sapiens OX=9606 GN=DLG5 PE=1 SV=4
225	P07339|CATD	356,4574	32,04	31,8	20,63	8,01	5,83	12	4460421	3758529,8	235341,97	323504,9	12	28	31	7	4	Carbamid	44552,22	Cathepsin D OS=Homo sapiens OX=9606 GN=CTSD PE=1 SV=1
95	P23142|FBLN	434,9141	30,58	30,58	26,74	20,48	18,78	22	3,62E+07	9848153	1486360	2992910,2	21	101	71	21	20	Carbamid	77214,05	Fibulin-1 OS=Homo sapiens OX=9606 GN=FBLN1 PE=1 SV=4
206	Q9Y536|PAL4	269,5659	28,05	11,59	11,59	28,05	28,05	6	212291,48	104125,625	604586,06	671093,94	4	3	3	9	8	Carbamid	18181,795	Peptidyl-prolyl cis-trans isomerase A-like 4A OS=Homo sapiens OX=9606 GN=PPIAL4A PE=2 SV=1
1040	Q9Y4C4|MFH	23,64662	0,57	0	0	0	0,57	1				91289,55	1				1		116950	Malignant fibrous histiocytoma-amplified sequence 1 OS=Homo sapiens OX=9606 GN=MFHAS1 PE=1 SV=2
251	Q5CZ79|AN2	77,62438	0,73	0,73	0	0	0	1	1080851,2				1	1					93908,586	Ankyrin repeat domain-containing protein 20B OS=Homo sapiens OX=9606 GN=ANKRD20A8P PE=2 SV=2
1072	Q86UU1|PHL	27,34611	0,44	0	0	0	0,44	1				132547,64	1				1		151161,81	Pleckstrin homology-like domain family B member 1 OS=Homo sapiens OX=9606 GN=PHLDB1 PE=1 SV=1
83	Q15438|CYH1	71,68612	1,51	0	1,51	0	0	1					1		1				46412,664	Cytohesin-1 OS=Homo sapiens OX=9606 GN=CYTH1 PE=1 SV=1
1279	Q8NBP7|PCS	29,63141	1,01	0	1,01	0	0	1		29072,06			1		1			Carbamid	74286,21	Proprotein convertase subtilisin/kexin type 9 OS=Homo sapiens OX=9606 GN=PCSK9 PE=1 SV=3
259	P09661|RU2A	74,25249	2,35	0	0	0	2,35	1				206989,28	1				1		28415,562	U2 small nuclear ribonucleoprotein A' OS=Homo sapiens OX=9606 GN=SNRPA1 PE=1 SV=2
295	P07195|LDHB	313,8652	26,35	26,35	17,96	5,39	5,39	9	953236,7	155544,08			6	17	18	5	2	Carbamid	36638,484	L-lactate dehydrogenase B chain OS=Homo sapiens OX=9606 GN=LDHB PE=1 SV=2
1280	Q6NTE8|MRN	23,04298	1,75	1,75	0	0	0	1					1	1					37743,23	MRN complex-interacting protein OS=Homo sapiens OX=9606 GN=MRNIP PE=1 SV=2
87	Q5T5Y3|CAM	40,01609	0,37	0	0	0,37	0,37	1			127495,2	284008,75	1			1	1	Acetylatio	177972	Calmodulin-regulated spectrin-associated protein 1 OS=Homo sapiens OX=9606 GN=CAMSAP1 PE=1 SV=2
159	Q8N957|ANK	117,8167	0,52	0	0	0	0,52	1				147559,02	1				1		129670,055	Ankyrin repeat and fibronectin type-III domain-containing protein 1 OS=Homo sapiens OX=9606 GN=ANKFN1 PE=2 SV=3
283	P78385|KRT8	300,0498	10,34	1,42	1,42	5,27	9,13	7			69957	356090,03	5	2	2	7	9	Carbamid	54195,406	Keratin, type II cuticular Hb3 OS=Homo sapiens OX=9606 GN=KRT83 PE=1 SV=2
1281	P34741|SDC2	40,25008	4,48	0	0	0	4,48	1				63173,43	1				1		22159,91	Syndecan-2 OS=Homo sapiens OX=9606 GN=SDC2 PE=1 SV=2
626	Q15836|VAM	200,4634	36	0	0	0	36	3				85873,38	3				3	Acetylatio	11309,086	Vesicle-associated membrane protein 3 OS=Homo sapiens OX=9606 GN=VAMP3 PE=1 SV=3
296	P62820|RAB1	308,9013	41,95	0	0	30,73	33,17	9			115217,945	314147,7	3			8	7		22677,746	Ras-related protein Rab-1A OS=Homo sapiens OX=9606 GN=RAB1A PE=1 SV=3
627	Q13510|ASAI	159,2211	7,09	0	7,09	0	0	3		53192,08			3		4			Carbamid	44659,64	Acid ceramidase OS=Homo sapiens OX=9606 GN=ASAH1 PE=1 SV=5
96	Q14055|CO9A	165,0542	2,76	0,87	1,02	0,87	0	3		428412,22			1	1	1	1			65130,938	Collagen alpha-2(IX) chain OS=Homo sapiens OX=9606 GN=COL9A2 PE=1 SV=2
511	P22087|FBRL	162,8863	4,98	1,87	2,18	1,87	4,98	4				49768,574	1	2	2	2	4		33784,22	rRNA 2'-O-methyltransferase fibrillarin OS=Homo sapiens OX=9606 GN=FBL PE=1 SV=2
48	P63261|ACTG	469,2287	58,4	52,53	23,2	55,2	48,27	31	275621,5		354296,47	38986,57	1	58	13	136	51	Carbamid	41792,836	Actin, cytoplasmic 2 OS=Homo sapiens OX=9606 GN=ACTG1 PE=1 SV=1
543	Q96N67|DOC	68,53143	0,28	0,28	0	0	0	1	70058,26				1	1					242560,64	Dedicator of cytokinesis protein 7 OS=Homo sapiens OX=9606 GN=DOCK7 PE=1 SV=4
1079	Q9BV73|CP2	24,10124	0,25	0	0	0,25	0	1			223339,5		1			1		Deamidat	281137,1	Centrosome-associated protein CEP250 OS=Homo sapiens OX=9606 GN=CEP250 PE=1 SV=2
1	Q5GFL6|VWA	115,3679	0,93	0,93	0,93	0,93	0,93	1	243674,56	306046,03	2751129	3,25E+07	1	4	7	4	5		82012,21	von Willebrand factor A domain-containing protein 2 OS=Homo sapiens OX=9606 GN=VWA2 PE=1 SV=1
401	P29279|CCN2	272,0204	16,62	16,62	13,75	0	0	6	1581937	111884,766			6	10	6			Carbamid	38091,434	CCN family member 2 OS=Homo sapiens OX=9606 GN=CCN2 PE=1 SV=2
398	Q9Y2Y8|PRG3	30,11886	2,67	0	0	2,67	2,67	1			289892,6	353094,47	1			1	1		25405,518	Proteoglycan 3 OS=Homo sapiens OX=9606 GN=PRG3 PE=1 SV=2
475	O15068|MCF	25,13488	0,53	0	0	0	0,53	1				2624083,2	1				1		128109,09	Guanine nucleotide exchange factor DBS OS=Homo sapiens OX=9606 GN=MCF2L PE=1 SV=2
770	Q9HCM1|RES	42,8563	0,34	0	0	0,34	0	1					1			1			194857,27	Retroelement silencing factor 1 OS=Homo sapiens OX=9606 GN=RESF1 PE=1 SV=3
142	Q7Z7G0|TAR	403,9596	17,7	5,06	7,02	6,09	16,85	19	750693,56	663681,25	166407,22	4926605,5	19	6	13	7	33	Carbamid	117895,07	Target of Nesh-SH3 OS=Homo sapiens OX=9606 GN=ABI3BP PE=1 SV=2
1225	Q8NFG4|FLC	24,10782	1,04	1,04	0	0	0	1					1	1					64473,305	Folliculin OS=Homo sapiens OX=9606 GN=FLCN PE=1 SV=1
519	Q8WUM0|NU	32,15548	0,52	0	0	0,52	0	1			100736,945		1			1			128979,02	Nuclear pore complex protein Nup133 OS=Homo sapiens OX=9606 GN=NUP133 PE=1 SV=2
94	Q8N6G2|TEX	64,53661	2,08	0	0	2,08	2,08	1			68159,61	66022,19	1			1	1	Deamidat	33595,12	Testis-expressed protein 26 OS=Homo sapiens OX=9606 GN=TEX26 PE=2 SV=1
190	P16118|F261	35,96386	1,27	1,27	0	0	0	1					1	1					54681,293	6-phosphofructo-2-kinase/fructose-2,6-bisphosphatase 1 OS=Homo sapiens OX=9606 GN=PFKFB1 PE=1 SV=3
1282	P54802|ANAG	28,81995	1,08	0	1,08	0	0	1		48999,766			1		1				82265,69	Alpha-N-acetylglucosaminidase OS=Homo sapiens OX=9606 GN=NAGLU PE=1 SV=2
1121	Q9GZU8|PIP3	34,83939	2,36	0	0	0	2,36	1				131281,55	1				1		28912,164	PSME3-interacting protein OS=Homo sapiens OX=9606 GN=PSME3IP1 PE=1 SV=1
1283	Q00587|BOR	32,54995	1,53	0	0	1,53	0	1			11255,904		1			1			40294,812	Cdc42 effector protein 1 OS=Homo sapiens OX=9606 GN=CDC42EP1 PE=1 SV=1
93	Q6PJP8|DCR1	110,4367	0,58	0,58	0,58	0,58	0,58	1	252959,12	39869,65	25816,422	253356,88	1	1	1	1	1		116399,555	DNA cross-link repair 1A protein OS=Homo sapiens OX=9606 GN=DCLRE1A PE=1 SV=3
1284	Q9UKW4|VA	25,08894	0,83	0	0	0,83	0	1			87476,32		1			1			97775,875	Guanine nucleotide exchange factor VAV3 OS=Homo sapiens OX=9606 GN=VAV3 PE=1 SV=1
672	Q2M3G0|AB	66,51751	0,48	0	0	0,48	0	1			480016,22		1			1			138640,81	ATP-binding cassette sub-family B member 5 OS=Homo sapiens OX=9606 GN=ABCB5 PE=1 SV=4
846	Q502W6|VW	31,68901	0,46	0	0	0,46	0	1			197740,75		1			3			145747,86	von Willebrand factor A domain-containing protein 3B OS=Homo sapiens OX=9606 GN=VWA3B PE=1 SV=3
1177	Q15003|CND	42,31192	0,81	0,81	0	0,81	0	1	115653,875		424286,38		1	1		2			82563,17	Condensin complex subunit 2 OS=Homo sapiens OX=9606 GN=NCAPH PE=1 SV=3
65	P00736|C1R_	471,1331	38,16	37,3	33,05	6,38	17,16	34	6,85E+07	2,73E+07	282507,8	879003,94	32	126	147	7	15	Carbamid	80118,55	Complement C1r subcomponent OS=Homo sapiens OX=9606 GN=C1R PE=1 SV=2
26	Q8IWV2|CNT	99,03803	0,58	0	0	0,58	0	1			60504,12		1			1			113453,64	Contactin-4 OS=Homo sapiens OX=9606 GN=CNTN4 PE=1 SV=1
504	Q9H7L2|KI3X	44,5827	1,7	0	0	1,7	0	1			714981,25		1			3			38777,3	Putative killer cell immunoglobulin-like receptor-like protein KIR3DX1 OS=Homo sapiens OX=9606 GN=KIR3DX1 PE=5 SV=2
1285	P05543|THBG	99,92004	2,41	2,41	0	0	0	1	22116,705				1	1					46324,5	Thyroxine-binding globulin OS=Homo sapiens OX=9606 GN=SERPINA7 PE=1 SV=2
339	Q92928|RAB1	246,2687	29,35	0	0	23,88	20,4	6			38113,543	60400,61	1			6	4		22016,96	Putative Ras-related protein Rab-1C OS=Homo sapiens OX=9606 GN=RAB1C PE=5 SV=2
283	Q8WYA0|IFT	40,32876	0,89	0	0	0,89	0	1			115794,38		1			1			79745,66	Intraflagellar transport protein 81 homolog OS=Homo sapiens OX=9606 GN=IFT81 PE=1 SV=1
880	Q8WUJ1|NEU	45,71551	2,27	2,27	0	0	0	1	1249522,2				1	2					28689,643	Neuferricin OS=Homo sapiens OX=9606 GN=CYB5D2 PE=1 SV=1
133	P07093|GDN	403,0945	46,23	41,46	25,13	24,37	19,6	18	2,11E+07	8313454,5	2325429,8	2177229,2	17	54	27	18	15	Carbamid	44002,22	Glia-derived nexin OS=Homo sapiens OX=9606 GN=SERPINE2 PE=1 SV=1
413	Q6NXT2|H3C	99,24007	18,52	4,44	4,44	5,19	18,52	4	340159,38	464861,53	729542	4931197	3	1	1	1	6	Oxidation	15213,731	Histone H3.3C OS=Homo sapiens OX=9606 GN=H3-5 PE=1 SV=3
770	P0C5J1|F86B	42,8563	1,82	0	0	1,82	0	1					1			1			36771,25	Putative protein N-methyltransferase FAM86B2 OS=Homo sapiens OX=9606 GN=FAM86B2 PE=1 SV=1
505	P08134|RHO	92,41126	10,36	0	0	10,36	6,22	3			276513,75	251414,78	2			4	2		22006,357	Rho-related GTP-binding protein RhoC OS=Homo sapiens OX=9606 GN=RHOC PE=1 SV=1
1074	O15265|ATX	29,53156	0,67	0	0	0,67	0	1			11944,6		1			1			95451,48	Ataxin-7 OS=Homo sapiens OX=9606 GN=ATXN7 PE=1 SV=1
1234	Q9UPS6|SET1	44,46783	0,31	0	0,31	0,31	0	1		79501,35	36570,387		1		2	1			212802,97	Histone-lysine N-methyltransferase SETD1B OS=Homo sapiens OX=9606 GN=SETD1B PE=1 SV=3
177	Q05639|EF1A	296,9755	12,96	3,89	0	12,96	8,21	7	128500,32		1532550,5	1448941,8	7	2		14	7		50470,156	Elongation factor 1-alpha 2 OS=Homo sapiens OX=9606 GN=EEF1A2 PE=1 SV=1
628	P31150|GDIA	128,9491	7,16	7,16	0	0	0	3	2494,3628				1	3					50582,72	Rab GDP dissociation inhibitor alpha OS=Homo sapiens OX=9606 GN=GDI1 PE=1 SV=2
1286	Q9P2S2|NRX	28,92551	0,35	0	0	0,35	0	1			441651,28		1			1			184981,95	Neurexin-2 OS=Homo sapiens OX=9606 GN=NRXN2 PE=2 SV=1
955	P99999|CYC_	122,1846	23,81	23,81	0	0	0	2	327747,2				2	3					11748,72	Cytochrome c OS=Homo sapiens OX=9606 GN=CYCS PE=1 SV=2
512	O14818|PSA7	233,1897	14,11	0	0	9,68	14,11	4			205440,67	265496,56	4			3	5		27886,844	Proteasome subunit alpha type-7 OS=Homo sapiens OX=9606 GN=PSMA7 PE=1 SV=1
1287	O95502|NPT	24,73591	1,2	1,2	0	0	0	1	48399,3				1	1					52846,203	Neuronal pentraxin receptor OS=Homo sapiens OX=9606 GN=NPTXR PE=3 SV=2
1040	Q8N461|FXL1	23,64662	1,25	0	0	0	1,25	1				91289,55	1				1		51657,785	F-box/LRR-repeat protein 16 OS=Homo sapiens OX=9606 GN=FBXL16 PE=2 SV=2
1143	Q15102|PA1E	112,4307	2,6	0	0	2,6	2,6	1					1			1	1		25734,238	Platelet-activating factor acetylhydrolase IB subunit alpha1 OS=Homo sapiens OX=9606 GN=PAFAH1B3 PE=1 SV=1
956	Q9UKU9|ANG	126,0696	3,04	1,22	0	0	1,83	2					1	1			1		57104,453	Angiopoietin-related protein 2 OS=Homo sapiens OX=9606 GN=ANGPTL2 PE=1 SV=1
89	P26038|MOE	441,7287	39,17	32,24	24,61	18,54	8,49	29	2941706,8	1191235,4	229970,17	59440,04	23	36	24	14	7		67820,04	Moesin OS=Homo sapiens OX=9606 GN=MSN PE=1 SV=3
52	Q5QGS0|NEX	54,84768	0,4	0	0	0,4	0,4	1			6541792	2502327,8	1			2	2	Carbamid	167550,52	Neurite extension and migration factor OS=Homo sapiens OX=9606 GN=NEXMIF PE=1 SV=1
1288	P10301|RRAS	23,07738	3,21	0	0	0	3,21	1				70924,914	1				1		23480,488	Ras-related protein R-Ras OS=Homo sapiens OX=9606 GN=RRAS PE=1 SV=1
957	P16112|PGCA	79,84662	0,55	0	0,28	0	0,55	2		13665,613		155566,69	2		1		2	Carbamid	261328,52	Aggrecan core protein OS=Homo sapiens OX=9606 GN=ACAN PE=1 SV=3
364	Q86SG5|S1A7	212,3999	24,75	0	0	12,87	24,75	3			218764,25	158582,06	3			4	3	Carbamid	11304,882	Protein S100-A7A OS=Homo sapiens OX=9606 GN=S100A7A PE=1 SV=3
198	P0DPH8|TBA	318,5372	24,89	3,33	0	24,89	12	8			310849,75		1	2		11	5	Deamidat	49959,543	Tubulin alpha-3D chain OS=Homo sapiens OX=9606 GN=TUBA3D PE=1 SV=1
940	P00367|DHE3	49,11904	3,41	0	3,41	0	0	2					2		2				61397,863	Glutamate dehydrogenase 1, mitochondrial OS=Homo sapiens OX=9606 GN=GLUD1 PE=1 SV=2
1289	P01009|A1AT	26,24104	1,67	0	1,67	0	0	1		996645,9			1		1				46736,547	Alpha-1-antitrypsin OS=Homo sapiens OX=9606 GN=SERPINA1 PE=1 SV=3
138	P0DMV8|HS7	394,9992	25,27	5,3	0	12,48	23,09	17	85032,92		99487,875	526184,8	5	4		15	19	Deamidat	70052,23	Heat shock 70 kDa protein 1A OS=Homo sapiens OX=9606 GN=HSPA1A PE=1 SV=1
770	Q96NJ5|KLH3	42,8563	0,97	0	0	0,97	0	1					1			1			70362,44	Kelch-like protein 32 OS=Homo sapiens OX=9606 GN=KLHL32 PE=1 SV=2
206	P62937|PPIA	352,8379	60	27,27	27,27	60	60	12	620350,1	104125,625	1756612,9	1266081,8	9	18	21	19	18	Deamidat	18012,49	Peptidyl-prolyl cis-trans isomerase A OS=Homo sapiens OX=9606 GN=PPIA PE=1 SV=2
1290	Q9Y3B8|ORN	26,5788	2,53	0	0	2,53	0	1			57433,465		1			1			26832,75	Oligoribonuclease, mitochondrial OS=Homo sapiens OX=9606 GN=REXO2 PE=1 SV=3
27	P0DJG4|THE	57,95885	1,29	0	0	1,29	0	1			19414,406		1			1			53028,207	Testicular haploid expressed gene protein-like OS=Homo sapiens OX=9606 GN=THEGL PE=2 SV=1
629	O00622|CCN	191,3588	9,19	9,19	2,1	0	0	3	250230,56	68825,875			3	3	1			Carbamid	42026,586	CCN family member 1 OS=Homo sapiens OX=9606 GN=CCN1 PE=1 SV=1
250	Q9Y2W7|CSE	49,34548	2,34	0	0	0	2,34	1				673270,6	1				1	Deamidat	29231,223	Calsenilin OS=Homo sapiens OX=9606 GN=KCNIP3 PE=1 SV=1
30	P03956|MMP	495,1866	58,42	46,7	58,42	0	37,1	47	2,14E+07	1,92E+08		9773932	44	105	325		49	Deamidat	54006,9	Interstitial collagenase OS=Homo sapiens OX=9606 GN=MMP1 PE=1 SV=3
960	O94782|UBP1	89,43633	1,53	0,76	0,76	1,53	0,76	2			223699,64		1	1	1	2	1		88207,24	Ubiquitin carboxyl-terminal hydrolase 1 OS=Homo sapiens OX=9606 GN=USP1 PE=1 SV=1
152	Q9ULJ7|ANR	97,08453	0,42	0,42	0,42	0	0	1	206241,6	63925,11			1	2	1			Carbamid	155859,19	Ankyrin repeat domain-containing protein 50 OS=Homo sapiens OX=9606 GN=ANKRD50 PE=1 SV=4
1291	P78539|SRPX	118,0549	2,8	2,8	0	0	2,8	1				23616,379	1	1			1		51571,92	Sushi repeat-containing protein SRPX OS=Homo sapiens OX=9606 GN=SRPX PE=1 SV=1
513	P25067|CO8A	187,3075	3,41	2,56	0,85	1,71	0,85	4	351230,7				1	7	3	3	4	Carbamid	67244,09	Collagen alpha-2(VIII) chain OS=Homo sapiens OX=9606 GN=COL8A2 PE=1 SV=2
706	P0DMR1|HNI	43,84666	3,41	3,41	0	0	0	1	79983,87				1	2					32029,445	Heterogeneous nuclear ribonucleoprotein C-like 4 OS=Homo sapiens OX=9606 GN=HNRNPCL4 PE=3 SV=1
14	P51674|GPM	100,3808	2,16	2,16	2,16	0	2,16	1	688577,7			23365,338	1	2	1		1	Carbamid	31209,537	Neuronal membrane glycoprotein M6-a OS=Homo sapiens OX=9606 GN=GPM6A PE=1 SV=2
14	P35555|FBN1	613,303	37,3	36,61	19,68	1,01	9,79	104	6,26E+07	6006540,5	27650,828	2819858	93	276	99	4	34	Carbamid	312297,5	Fibrillin-1 OS=Homo sapiens OX=9606 GN=FBN1 PE=1 SV=4
901	E9PQR5|NPIE	32,753	1,39	1,39	0	0	0	1	228840,3				1	1				Deamidat	49616,844	Nuclear pore complex-interacting protein family member B8 OS=Homo sapiens OX=9606 GN=NPIPB8 PE=3 SV=1

367	Q96D15|RCN	278,7343	23,17	23,17	9,76	0	0	6	1864544,8	261506,6			6	16	5			Oxidation	37492,977	Reticulocalbin-3 OS=Homo sapiens OX=9606 GN=RCN3 PE=1 SV=1
94	Q9H4B8|DPE	96,43889	1,43	0	0	0	1,43	1				75865,734	1				1		53687,363	Dipeptidase 3 OS=Homo sapiens OX=9606 GN=DPEP3 PE=1 SV=2
1292	P37837|TALD	72,95464	2,67	2,67	0	0	0	1	73252,164				1	1					37540,12	Transaldolase OS=Homo sapiens OX=9606 GN=TALDO1 PE=1 SV=2
329	P05089|ARGI	291,9245	19,25	1,86	0	19,25	18,94	8	133473,9		2550000	2018150,2	8	1		12	10	Carbamid	34734,938	Arginase-1 OS=Homo sapiens OX=9606 GN=ARG1 PE=1 SV=2
1156	Q9C0D5|TAN	27,61164	0,48	0	0	0	0,48	1					1				1		202219,25	Protein TANC1 OS=Homo sapiens OX=9606 GN=TANC1 PE=1 SV=3
514	Q9Y6C5|PTC	109,46	1,5	0,5	0,5	0	1	3					1	1	6		3	Deamidat	130544,17	Protein patched homolog 2 OS=Homo sapiens OX=9606 GN=PTCH2 PE=1 SV=2
207	Q08629|TICN	350,3263	30,98	30,98	0	0	0	13	2332635				12	25				Carbamid	49124,254	Testican-1 OS=Homo sapiens OX=9606 GN=SPOCK1 PE=1 SV=1
226	P31946|1433	339,8781	42,28	42,28	18,7	18,29	21,95	11	1284211,6	141211,28			4	18	11	10	8	Carbamid	28082,393	14-3-3 protein beta/alpha OS=Homo sapiens OX=9606 GN=YWHAB PE=1 SV=3
443	P53801|PTTG	264,5351	25	0	0	21,67	25	5			505590,38	2991665,5	4			4	12	Carbamid	20323,734	Pituitary tumor-transforming gene 1 protein-interacting protein OS=Homo sapiens OX=9606 GN=PTTG1IP PE=1 SV=1
630	Q4VX76|SYTL	174,4276	2,95	1,97	0,98	1,97	1,97	3			116166,08	198165,22	1	2	1	2	2	Carbamid	68559,58	Synaptotagmin-like protein 3 OS=Homo sapiens OX=9606 GN=SYTL3 PE=1 SV=3
961	Q07507|DERM	112,5102	10,95	10,95	0	0	0	2	96069,34				2	4				Carbamid	24004,826	Dermatopontin OS=Homo sapiens OX=9606 GN=DPT PE=1 SV=2
660	Q12756|KIF1	25,21471	0,36	0	0	0,36	0	1			89165,93		1			1			191064,14	Kinesin-like protein KIF1A OS=Homo sapiens OX=9606 GN=KIF1A PE=1 SV=2
108	Q13464|ROCI	60,04453	0,44	0,44	0	0	0	1	544477,3				1	1					158174,67	Rho-associated protein kinase 1 OS=Homo sapiens OX=9606 GN=ROCK1 PE=1 SV=1
584	Q14003|KCN	32,2939	0,79	0	0	0	0,79	1				52988,613	1				1		80578,03	Potassium voltage-gated channel subfamily C member 3 OS=Homo sapiens OX=9606 GN=KCNC3 PE=1 SV=3
1239	Q96A47|ISL2	34,35205	1,67	0	0	0	1,67	1					1				1		39768,473	Insulin gene enhancer protein ISL-2 OS=Homo sapiens OX=9606 GN=ISL2 PE=1 SV=1
962	Q96DR8|MU	67,62816	17,78	0	0	17,78	7,78	2			1590626,8	2483633,2	2			3	5	Deamidat	9039,233	Mucin-like protein 1 OS=Homo sapiens OX=9606 GN=MUCL1 PE=1 SV=1
1293	Q2M1Z3|RHC	24,38863	0,42	0	0,42	0	0	1		117195,56			1		1				156985	Rho GTPase-activating protein 31 OS=Homo sapiens OX=9606 GN=ARHGAP31 PE=1 SV=2
963	P20618|PSB1	81,37886	12,03	0	0	0	12,03	2				7342,887	2				2		26489,363	Proteasome subunit beta type-1 OS=Homo sapiens OX=9606 GN=PSMB1 PE=1 SV=2
283	B1AK53|ESPN	40,32876	0,7	0	0	0,7	0	1			115794,38		1			1			91732,54	Espin OS=Homo sapiens OX=9606 GN=ESPN PE=1 SV=1
23	P11274|BCR_	88,97265	0,47	0	0	0,47	0,47	1			25847,486	25132,662	1			1	1		142819,34	Breakpoint cluster region protein OS=Homo sapiens OX=9606 GN=BCR PE=1 SV=2
444	P52565|GDIR	249,7951	22,06	22,06	0	0	0	5	541093,25				4	10					23207,104	Rho GDP-dissociation inhibitor 1 OS=Homo sapiens OX=9606 GN=ARHGDIA PE=1 SV=3
1025	Q8NCW5|NN	27,11249	2,08	0	0	0	2,08	1				76108,34	1				2		31674,59	NAD(P)H-hydrate epimerase OS=Homo sapiens OX=9606 GN=NAXE PE=1 SV=2
330	P60033|CD81	244,9851	25	0	0	18,22	25	4			379846,8	4449802	4			9	17	Carbamid	25809,395	CD81 antigen OS=Homo sapiens OX=9606 GN=CD81 PE=1 SV=1
613	Q2KHR3|QSE	47,05321	0,4	0	0	0	0,4	1				494669,56	1				1		189972,1	Glutamine and serine-rich protein 1 OS=Homo sapiens OX=9606 GN=QSER1 PE=1 SV=3
331	Q92743|HTR	303,6321	16,46	16,46	5,21	2,29	8,33	8	1015980,56	87612,65	42552,86	189889,36	7	20	2	1	4	Carbamid	51286,95	Serine protease HTRA1 OS=Homo sapiens OX=9606 GN=HTRA1 PE=1 SV=1
1294	O60507|TPST	26,17028	1,62	0	0	1,62	0	1			98621,195		1			1			42188,39	Protein-tyrosine sulfotransferase 1 OS=Homo sapiens OX=9606 GN=TPST1 PE=1 SV=1
445	Q92520|FAM	232,4279	17,62	17,62	14,1	0	0	5	142011,2	130265,72			5	5	4				24680,463	Protein FAM3C OS=Homo sapiens OX=9606 GN=FAM3C PE=1 SV=1
1121	Q96T76|MMS	34,83939	0,58	0	0	0	0,58	1				131281,55	1				1		113289,555	MMS19 nucleotide excision repair protein homolog OS=Homo sapiens OX=9606 GN=MMS19 PE=1 SV=2
1040	Q9BWV7|TTL	23,64662	1,01	0	0	0	1,01	1				91289,55	1				1		67336,09	Probable tubulin polyglutamylase TTLL2 OS=Homo sapiens OX=9606 GN=TTLL2 PE=2 SV=3
466	Q9Y625|GPC	34,32951	1,08	1,08	1,08	0	1,08	1	13068,148	17805,719			1	1	1		1	Carbamid	62735,52	Glypican-6 OS=Homo sapiens OX=9606 GN=GPC6 PE=1 SV=1
1295	Q14956|GPN	30,46914	1,57	1,57	0	0	0	1	210941,06				1	1					63922,543	Transmembrane glycoprotein NMB OS=Homo sapiens OX=9606 GN=GPNMB PE=1 SV=2
1296	Q9BYE3|LCE3	118,316	19,57	0	0	19,57	19,57	1			75846,56	55261,2	1			1	1	Carbamid	9443,5205	Late cornified envelope protein 3D OS=Homo sapiens OX=9606 GN=LCE3D PE=1 SV=1
238	Q8TAV4|STM	114,2811	2,41	0	0	2,41	2,41	1				1330150,9	1			1	2		32135,457	Stomatin-like protein 3 OS=Homo sapiens OX=9606 GN=STOML3 PE=1 SV=1
682	Q5VVH2|FKB	59,72122	5,56	0	0	0	5,56	1					1				1		12179,013	Peptidyl-prolyl cis-trans isomerase FKBP1C OS=Homo sapiens OX=9606 GN=FKBP1C PE=3 SV=1
659	Q5VWM4|PR	42,47769	1,27	0	1,27	0	0	1		806622,25			1		3				53654,81	PRAME family member 8 OS=Homo sapiens OX=9606 GN=PRAMEF8 PE=2 SV=2
1153	Q6ZP82|CC1	24,38223	0,41	0	0,41	0	0	1					1		1				166261,25	Coiled-coil domain-containing protein 141 OS=Homo sapiens OX=9606 GN=CCDC141 PE=1 SV=2
787	Q5T7P8|SYT6	28,86495	1,18	0	0	0	1,18	1				264093,28	1				1		57324,99	Synaptotagmin-6 OS=Homo sapiens OX=9606 GN=SYT6 PE=1 SV=3
57	P02545|LMN	486,9533	54,97	54,07	30,87	8,58	0	41	1,51E+07	1550217,9	164894,67		39	81	33	8		Carbamid	74139,484	Prelamin-A/C OS=Homo sapiens OX=9606 GN=LMNA PE=1 SV=1
966	Q96MT8|CEP	80,86674	1,71	0	0	0,85	0,85	2				2,36E+07	1			1	2		81343,875	Centrosomal protein of 63 kDa OS=Homo sapiens OX=9606 GN=CEP63 PE=1 SV=1
332	O15118|NPC	313,1596	5,56	0	0	0,55	5,56	8				837441,6	7			1	10	Carbamid	142166,84	NPC intracellular cholesterol transporter 1 OS=Homo sapiens OX=9606 GN=NPC1 PE=1 SV=2
504	Q08345|DDR	44,5827	0,66	0	0	0,66	0	1			714981,25		1			3			101127,64	Epithelial discoidin domain-containing receptor 1 OS=Homo sapiens OX=9606 GN=DDR1 PE=1 SV=1
514	Q86YD1|PTO	44,08848	1,44	0	0	0	1,44	1					1				1		46868,53	Prostate tumor-overexpressed gene 1 protein OS=Homo sapiens OX=9606 GN=PTOV1 PE=1 SV=1
543	Q1RMZ1|SAM	68,53143	1,48	1,48	0	0	0	1	70058,26				1	1					46323,824	S-adenosylmethionine sensor upstream of mTORC1 OS=Homo sapiens OX=9606 GN=BMT2 PE=1 SV=1
429	Q12809|KCN	118,8111	0,6	0,6	0,6	0,6	0,6	2	489938,22	91849,77	58832,07	232439,36	1	1	1	2	1		126654,945	Potassium voltage-gated channel subfamily H member 2 OS=Homo sapiens OX=9606 GN=KCNH2 PE=1 SV=1
159	Q9UJ55|MAG	79,9183	0,48	0	0	0,48	0,48	1			48044,566	135469,5	1			1	1		132822,12	MAGE-like protein 2 OS=Homo sapiens OX=9606 GN=MAGEL2 PE=1 SV=2
480	P56962|STX1	85,08199	1,99	0	0	1,99	1,99	1				25529,691	1			1	1		33403,496	Syntaxin-17 OS=Homo sapiens OX=9606 GN=STX17 PE=1 SV=2
1294	Q9Y2K3|MYH	26,17028	0,31	0	0	0,31	0	1			98621,195		1			1			224618,97	Myosin-15 OS=Homo sapiens OX=9606 GN=MYH15 PE=1 SV=5
1297	Q3MJ13|WDI	51,64509	0,54	0,54	0	0	0	1	136324,05				1	1					123425,125	WD repeat-containing protein 72 OS=Homo sapiens OX=9606 GN=WDR72 PE=2 SV=2
329	P43034|LIS1_	58,51812	1,46	1,46	0	1,46	1,46	1	133473,9		419747,28	265960,53	1	1		1	1		46637,965	Platelet-activating factor acetylhydrolase IB subunit beta OS=Homo sapiens OX=9606 GN=PAFAH1B1 PE=1 SV=2
115	P17936|IBP3_	413,7091	61,17	61,17	15,81	0	0	21	2,56E+07	346461,12			20	88	9			Deamidat	31674,203	Insulin-like growth factor-binding protein 3 OS=Homo sapiens OX=9606 GN=IGFBP3 PE=1 SV=2
1254	Q9UNS2|CSN	61,65413	1,42	0	0	0	1,42	1				119894,2	1				1		47873,13	COP9 signalosome complex subunit 3 OS=Homo sapiens OX=9606 GN=COPS3 PE=1 SV=3
5	Q8NBF2|NHL	95,18884	0,83	0,83	0,83	0	0	1	7970265	233452,2			1	5	1				79443,58	NHL repeat-containing protein 2 OS=Homo sapiens OX=9606 GN=NHLRC2 PE=1 SV=1
360	Q96FN5|KIF1	65,74255	0,93	0	0	0	0,93	1				3201,734	1				1		70660,07	Kinesin-like protein KIF12 OS=Homo sapiens OX=9606 GN=KIF12 PE=1 SV=3
87	Q5JPF3|AN3	40,01609	0,34	0	0	0,34	0,34	1			127495,2	284008,75	1			1	1	Acetylatic	199748,11	Ankyrin repeat domain-containing protein 36C OS=Homo sapiens OX=9606 GN=ANKRD36C PE=1 SV=3
148	P54652|HSP7	377,5791	20,5	7,2	0	17,53	17,53	16	222550,81			58527,72	3	8		26	28	Deamidat	70020,97	Heat shock-related 70 kDa protein 2 OS=Homo sapiens OX=9606 GN=HSPA2 PE=1 SV=1
252	Q5QNW6|H2	317,3283	66,67	34,13	27,78	29,37	66,67	10	117890,07	89813,125	87343,49	987559,8	1	7	7	7	17	Oxidation	13920,161	Histone H2B type 2-F OS=Homo sapiens OX=9606 GN=H2BC18 PE=1 SV=3
73	Q9NR97|TLR	35,77164	0,58	0	0	0,58	0	1			67731,13		1			1			119828,195	Toll-like receptor 8 OS=Homo sapiens OX=9606 GN=TLR8 PE=1 SV=1
6	Q8IUD2|RB6	49,33768	0,54	0,54	0,54	0	0	1	258931,9	88723,61			1	1	2				128086,39	ELKS/Rab6-interacting/CAST family member 1 OS=Homo sapiens OX=9606 GN=ERC1 PE=1 SV=1
515	Q969H8|MYD	105,2059	17,34	17,34	0	0	0	4	794961,6				4	5					18795,203	Myeloid-derived growth factor OS=Homo sapiens OX=9606 GN=MYDGF PE=1 SV=1
368	O14786|NRP	218,9353	5,74	0	3,03	0,65	2,71	7		44731,715		91618,58	5		4	1	3	Carbamid	103134,336	Neuropilin-1 OS=Homo sapiens OX=9606 GN=NRP1 PE=1 SV=3
515	Q96BY6|DOC	26,32922	0,27	0,27	0	0	0	1					1	1					249530,92	Dedicator of cytokinesis protein 10 OS=Homo sapiens OX=9606 GN=DOCK10 PE=1 SV=3
247	P31947|1433	329,2072	36,69	13,31	9,27	36,69	13,31	10			286210,56		4	10	6	15	5	Oxidation	27774,059	14-3-3 protein sigma OS=Homo sapiens OX=9606 GN=SFN PE=1 SV=1
994	P01614|KVD	36,45289	5,79	5,79	0	0	0	1	92659,414				1	1					13310,112	Immunoglobulin kappa variable 2D-40 OS=Homo sapiens OX=9606 GN=IGKV2D-40 PE=1 SV=2
235	Q14674|ESPL	85,95554	0,28	0,28	0,28	0	0	1	164047,08	165661,67			1	1	1				233175,47	Separin OS=Homo sapiens OX=9606 GN=ESPL1 PE=1 SV=3
1298	P31040|SDH	24,83735	1,36	0	0	1,36	0	1					1			1			72691,51	Succinate dehydrogenase [ubiquinone] flavoprotein subunit, mitochondrial OS=Homo sapiens OX=9606 GN=SDHA PE=1 SV=2
28	Q96EK2|PF2	91,29875	1,32	1,32	1,32	1,32	1,32	1	762553,2	335383,72	263353,2	563512,5	1	1	1	1	1		57454,707	PHD finger protein 21B OS=Homo sapiens OX=9606 GN=PHF21B PE=1 SV=1
926	Q96GN5|CDA	24,46977	1,32	0	0	0	1,32	1				95245,69	1				1		52206,258	Cell division cycle-associated 7-like protein OS=Homo sapiens OX=9606 GN=CDCA7L PE=1 SV=2
967	P42357|HUT	48,17644	1,98	0,91	0,91	1,07	1,07	2			46487,13	49012,637	1	1	1	1	1		72697,62	Histidine ammonia-lyase OS=Homo sapiens OX=9606 GN=HAL PE=1 SV=1
84	O00634|NET	27,33654	1,03	0	0	0	1,03	1				98403,8	1				1		61466,055	Netrin-3 OS=Homo sapiens OX=9606 GN=NTN3 PE=1 SV=1
346	O94850|DEN	29,32948	0,84	0	0	0,84	0	1			147630,33		1			1			75995,69	Dendrin OS=Homo sapiens OX=9606 GN=DDN PE=1 SV=3
1299	O94851|MIC	28,30486	0,31	0	0	0	0,31	1				32530,209	1				1	Oxidation	219061,31	[F-actin]-monooxygenase MICAL2 OS=Homo sapiens OX=9606 GN=MICAL2 PE=1 SV=2
1221	Q99650|OSM	26,16646	0,61	0	0,61	0	0	1					1		2				110508,516	Oncostatin-M-specific receptor subunit beta OS=Homo sapiens OX=9606 GN=OSMR PE=1 SV=1
29	Q15560|TCEA	115,7723	2,01	2,01	2,01	2,01	2,01	1	411798,44	248102,77	94237,42	370433,34	1	1	2	1	1		33600,766	Transcription elongation factor A protein 2 OS=Homo sapiens OX=9606 GN=TCEA2 PE=1 SV=1
153	O75106|AOC	100,7645	0,93	0,93	0,93	0	0	1	1619739,4	422202,72			1	5	4				83673,37	Retina-specific copper amine oxidase OS=Homo sapiens OX=9606 GN=AOC2 PE=1 SV=2
144	Q9Y5Y0|FLVC	72,05529	1,08	1,08	1,08	0	0	1	2205535,5	616605,9			1	7	4			Deamidat	59862,887	Feline leukemia virus subgroup C receptor-related protein 1 OS=Homo sapiens OX=9606 GN=FLVCR1 PE=1 SV=1
1032	Q8TE59|ATS1	47,494	0,5	0,5	0,5	0	0	1	304970,78	254798,3			1	1	2				134047,72	A disintegrin and metalloproteinase with thrombospondin motifs 19 OS=Homo sapiens OX=9606 GN=ADAMTS19 PE=1 SV=2
1300	Q9C091|GRB	42,58664	0,42	0	0	0,42	0	1			262672,5		1			1			214354,34	GREB1-like protein OS=Homo sapiens OX=9606 GN=GREB1L PE=1 SV=2
41	P07355|ANX	497,6285	75,22	45,13	7,37	74,34	69,32	41	15405,145		1,39E+07	6495724,5	7	19	3	135	106	Carbamid	38604,035	Annexin A2 OS=Homo sapiens OX=9606 GN=ANXA2 PE=1 SV=2
298	Q8N257|H2B	283,1597	59,52	23,02	9,52	18,25	59,52	9				36598,527	1	5	3	5	14	Oxidation	13908,106	Histone H2B type 3-B OS=Homo sapiens OX=9606 GN=H2BC26 PE=1 SV=3
142	Q9UQR0|SCN	88,34358	0,86	0	0,86	0,86	0,86	1		55211,223	28992,957	216936,48	1		1	1	2		77256,8	Sex comb on midleg-like protein 2 OS=Homo sapiens OX=9606 GN=SCML2 PE=1 SV=1
1067	Q9NRC6|SPT	24,35381	0,16	0	0	0,16	0	1			37122,6		1			1			416750	Spectrin beta chain, non-erythrocytic 5 OS=Homo sapiens OX=9606 GN=SPTBN5 PE=1 SV=2
227	Q9Y240|CLC1	343,3532	32,2	32,2	18,58	0	0	11	3066457,8	406230,75			11	23	11			Deamidat	35694,63	C-type lectin domain family 11 member A OS=Homo sapiens OX=9606 GN=CLEC11A PE=1 SV=1
235	Q8NGT1|OR2	85,95554	1,9	1,9	1,9	0	0	1	164047,08	165661,67			1	1	1				35045,133	Olfactory receptor 2K2 OS=Homo sapiens OX=9606 GN=OR2K2 PE=2 SV=3
84	Q96PY6|NEK	44,39447	0,48	0	0	0	0,48	1				29320,566	1				1		142828,39	Serine/threonine-protein kinase Nek1 OS=Homo sapiens OX=9606 GN=NEK1 PE=1 SV=2
413	Q5TEC6|H37_	79,49931	13,97	0	0	5,15	13,97	3			729542	1768835,8	2			1	4	Oxidation	15430,102	Histone H3-7 OS=Homo sapiens OX=9606 GN=H3-7 PE=1 SV=1
970	Q8N6G6|ATL	50,59925	0,74	0,74	0	0	0	2	50530,586				2	3					193409,22	ADAMTS-like protein 1 OS=Homo sapiens OX=9606 GN=ADAMTSL1 PE=1 SV=4
1301	A2RU67|F234	48,60814	0,96	0	0	0	0,96	1				127533,836	1				1		67038,55	Protein FAM234B OS=Homo sapiens OX=9606 GN=FAM234B PE=1 SV=1
1302	A7KAX9|RHG	33,59106	0,29	0	0	0	0,29	1				21281,805	1				1		230529,02	Rho GTPase-activating protein 32 OS=Homo sapiens OX=9606 GN=ARHGAP32 PE=1 SV=1
591	Q9Y6N8|CAD	26,75112	0,76	0,76	0	0	0	1	191915,34				1	1					88451,33	Cadherin-10 OS=Homo sapiens OX=9606 GN=CDH10 PE=1 SV=2
901	A6NHN6|NPI	32,753	1,35	1,35	0	0	0	1	228840,3				1	1				Deamidat	51266,74	Nuclear pore complex-interacting protein family member B15 OS=Homo sapiens OX=9606 GN=NPIPB15 PE=3 SV=3
84	Q93038|TNR	28,22482	1,44	1,44	0	0	0	1	472656,12				1	1					45384,97	Tumor necrosis factor receptor superfamily member 25 OS=Homo sapiens OX=9606 GN=TNFRSF25 PE=1 SV=2
869	Q8IWZ3|ANK	28,30982	0,24	0	0	0,24	0	1			1953030		1			1			269457,5	Ankyrin repeat and KH domain-containing protein 1 OS=Homo sapiens OX=9606 GN=ANKHD1 PE=1 SV=1
1233	P0DOY2|IGLC	53,65772	9,43	0	0	9,43	9,43	1			96670,7	37221,582	1			1	1		11293,564	Immunoglobulin lambda constant 2 OS=Homo sapiens OX=9606 GN=IGLC2 PE=1 SV=1
1303	P17301|ITA2_	37,37518	1,19	0	0	0	1,19	1					1				1	Carbamid	129296,38	Integrin alpha-2 OS=Homo sapiens OX=9606 GN=ITGA2 PE=1 SV=2
13	P02461|CO3	581,9637	53,68	51,64	44,88	10,23	22,44	96	9,04E+07	2,15E+07	1076423,6	6102571,5	73	226	157	17	38	Oxidation	138564,28	Collagen alpha-1(III) chain OS=Homo sapiens OX=9606 GN=COL3A1 PE=1 SV=4
1304	Q8IVV2|LOXI	29,08254	0,29	0,29	0	0	0	1	254281,47				1	2					235677,45	Lipoxygenase homology domain-containing protein 1 OS=Homo sapiens OX=9606 GN=LOXHD1 PE=2 SV=4
230	Q01484|ANK	39,20821	0,15	0	0	0,15	0	1			46140,46		1			1			433714,66	Ankyrin-2 OS=Homo sapiens OX=9606 GN=ANK2 PE=1 SV=4
516	P62879|GBB	160,9656	12,65	0	0	7,06	7,65	4			163266,73		2			4	2	Carbamid	37331,027	Guanine nucleotide-binding protein G(I)/G(S)/G(T) subunit beta-2 OS=Homo sapiens OX=9606 GN=GNB2 PE=1 SV=3
1305	O14732|IMP	23,97142	3,12	0	0	3,12	0	1			31110,557		1			1			31320,889	Inositol monophosphatase 2 OS=Homo sapiens OX=9606 GN=IMPA2 PE=1 SV=1
1148	Q9BYE2|TMP	46,73671	1,37	1,37	0	1,37	1,37	1	705888,9		71171,73	70945,36	1	3		1	1		63167,145	Transmembrane protease serine 13 OS=Homo sapiens OX=9606 GN=TMPRSS13 PE=2 SV=5
285	O95096|NKX	42,64934	2,56	0	0	0	2,56	1				70901,19	1				1	Deamidat	30132,574	Homeobox protein Nkx-2.2 OS=Homo sapiens OX=9606 GN=NKX2-2 PE=2 SV=1

38	P09871|C1S_	488,1164	47,24	47,24	37,5	7,85	16,28	39	6,43E+07	2,07E+07	1137482	1507731	37	168	102	7	14	Carbamid	76684,43	Complement C1s subcomponent OS=Homo sapiens OX=9606 GN=C1S PE=1 SV=1
246	P16083|NQO	97,35769	2,6	2,6	2,6	0	2,6	1	323452,25	311461,88		16182,259	1	1	1		1		25918,564	Ribosyldihydronicotinamide dehydrogenase [quinone] OS=Homo sapiens OX=9606 GN=NQO2 PE=1 SV=5
864	P15313|VATE	89,06034	1,95	0	0	0	1,95	1				73897,914	1				3		56833,035	V-type proton ATPase subunit B, kidney isoform OS=Homo sapiens OX=9606 GN=ATP6V1B1 PE=1 SV=3
45	O14513|NCKI	102,0146	0,31	0,31	0,31	0,31	0,31	1	865487,94	181649,64	66948,86	167803,1	1	2	1	1	1		208537,05	Nck-associated protein 5 OS=Homo sapiens OX=9606 GN=NCKAP5 PE=1 SV=2
1143	P68402|PA1E	112,4307	2,62	0	0	2,62	2,62	1					1			1	1		25569,27	Platelet-activating factor acetylhydrolase IB subunit alpha2 OS=Homo sapiens OX=9606 GN=PAFAH1B2 PE=1 SV=1
972	P41271|NBL1	77,29874	7,73	7,73	7,73	0	0	2	1078393,8	384553,28			2	5	3			Carbamid	19407,95	Neuroblastoma suppressor of tumorigenicity 1 OS=Homo sapiens OX=9606 GN=NBL1 PE=1 SV=2
228	P24592|IBP6_	316,7541	32,5	27,5	22,08	2,5	4,17	11	4631568	2798171		48198,32	7	18	13	1	1	Carbamid	25322,428	Insulin-like growth factor-binding protein 6 OS=Homo sapiens OX=9606 GN=IGFBP6 PE=1 SV=1
163	Q8TBJ4|PLPR	89,8404	1,85	1,85	1,85	0	0	1	253479,38	118913,28			1	1	1				35794,574	Phospholipid phosphatase-related protein type 1 OS=Homo sapiens OX=9606 GN=PLPPR1 PE=1 SV=1
634	Q8N1I0|DOC	95,03209	0,61	0	0,31	0,61	0,31	2		4961949	219925,28	3924922	1		11	10	1	Deamidat	225206,25	Dedicator of cytokinesis protein 4 OS=Homo sapiens OX=9606 GN=DOCK4 PE=1 SV=3
3	P0DPB3|SCHI	74,84779	1,64	0	0	1,64	1,44	2			38447,664		2			3	1		53479,95	Schwannomin-interacting protein 1 OS=Homo sapiens OX=9606 GN=SCHIP1 PE=1 SV=1
513	O75582|KS6A	27,77183	0,75	0,75	0	0	0	1	351230,7				1	1				Carbamid	89865,414	Ribosomal protein S6 kinase alpha-5 OS=Homo sapiens OX=9606 GN=RPS6KA5 PE=1 SV=1
635	P20073|ANX/	103,5193	6,15	0	0	2,87	6,15	3			135705,72	309774,16	3			2	3		52739,24	Annexin A7 OS=Homo sapiens OX=9606 GN=ANXA7 PE=1 SV=3
973	P51692|STA5	137,0156	1,52	0	0	0,76	1,52	2				96269,87	1			2	2		89865,88	Signal transducer and activator of transcription 5B OS=Homo sapiens OX=9606 GN=STAT5B PE=1 SV=2
266	Q9UHJ6|SHPI	24,39508	1,26	0	1,26	0	0	1		104756,59			1		1				51504,83	Sedoheptulokinase OS=Homo sapiens OX=9606 GN=SHPK PE=1 SV=4
974	Q6DT37|MRC	69,33119	0,77	0	0,39	0	0,39	2				7724995,5	1		1		1		172459,27	Serine/threonine-protein kinase MRCK gamma OS=Homo sapiens OX=9606 GN=CDC42BPG PE=1 SV=2
1306	O00423|EMA	30,48029	0,74	0	0,74	0	0	1					1		2				89860,88	Echinoderm microtubule-associated protein-like 1 OS=Homo sapiens OX=9606 GN=EML1 PE=1 SV=3
1183	O15054|KDM	25,73077	0,37	0	0	0,37	0	1					1			1			176632,34	Lysine-specific demethylase 6B OS=Homo sapiens OX=9606 GN=KDM6B PE=1 SV=4
793	P62277|RS13_	33,14592	4,64	0	0	0	4,64	1					1				1		17222,303	40S ribosomal protein S13 OS=Homo sapiens OX=9606 GN=RPS13 PE=1 SV=2
124	Q7RTS7|K2C7	387,5158	18,71	4,35	6,62	18,71	17,39	21					1	6	14	38	31	Deamidat	57865,387	Keratin, type II cytoskeletal 74 OS=Homo sapiens OX=9606 GN=KRT74 PE=1 SV=2
446	Q14532|K1H2	168,6128	4,24	1,56	1,56	4,24	1,56	4			337436,84		2	2	4	11	6	Acetylatic	50342,996	Keratin, type I cuticular Ha2 OS=Homo sapiens OX=9606 GN=KRT32 PE=1 SV=3
15	P04259|K2C6	580,4205	77,3	21,81	24,82	77,3	66,31	84			2834828	1930832	2	30	52	526	347	Acetylatic	60066,953	Keratin, type II cytoskeletal 6B OS=Homo sapiens OX=9606 GN=KRT6B PE=1 SV=5
50	Q9NR99|MXF	497,1161	13,44	6,79	3,71	0	8,88	44	2737008	297859,38		2217974	42	29	13		33	Carbamid	312150,28	Matrix-remodeling-associated protein 5 OS=Homo sapiens OX=9606 GN=MXRA5 PE=1 SV=3
1307	Q9P2T1|GMP	40,21013	3,45	0	0	3,45	0	1					1			1		Carbamid	37874,383	GMP reductase 2 OS=Homo sapiens OX=9606 GN=GMPR2 PE=1 SV=1
22	Q8WW34|TM	36,56846	3,08	3,08	0	0	3,08	1					1	1			1		21841,688	Transmembrane protein 239 OS=Homo sapiens OX=9606 GN=TMEM239 PE=1 SV=3
57	P20700|LMNI	81,07773	1,37	1,37	0	0	0	1	313491,56				1	1					66408,336	Lamin-B1 OS=Homo sapiens OX=9606 GN=LMNB1 PE=1 SV=2
327	P01859|IGHG	217,8114	9,37	0	0	9,37	0	4			381978,12		1			7		Carbamid	43805,766	Immunoglobulin heavy constant gamma 2 OS=Homo sapiens OX=9606 GN=IGHG2 PE=1 SV=3
161	Q9P2B2|FPRI	398,5372	17,29	0	0	14,9	17,29	17			1146151,8	3662509	16			19	23	Carbamid	98556,45	Prostaglandin F2 receptor negative regulator OS=Homo sapiens OX=9606 GN=PTGFRN PE=1 SV=2
1123	O00220|TR10	23,45577	1,28	0	0	1,28	0	1			51930,484		1			1			50089,008	Tumor necrosis factor receptor superfamily member 10A OS=Homo sapiens OX=9606 GN=TNFRSF10A PE=1 SV=3
973	P42229|STA5	137,0156	1,51	0	0	0,76	1,51	2				96269,87	1			2	2		90647	Signal transducer and activator of transcription 5A OS=Homo sapiens OX=9606 GN=STAT5A PE=1 SV=1
1308	O15145|ARP(	47,48592	6,18	0	0	6,18	0	1					1			1			20546,666	Actin-related protein 2/3 complex subunit 3 OS=Homo sapiens OX=9606 GN=ARPC3 PE=1 SV=3
10	P13645|K1C1	604,8795	73,46	48,8	43,15	70,55	72,43	107	9201071	1,15E+07	8,98E+08	7,10E+08	84	77	93	596	473	Acetylatic	58827,086	Keratin, type I cytoskeletal 10 OS=Homo sapiens OX=9606 GN=KRT10 PE=1 SV=6
1269	Q8WXX5|DN.	26,62863	2,31	0	0	2,31	0	1			23824,734		1			1			29909,709	DnaJ homolog subfamily C member 9 OS=Homo sapiens OX=9606 GN=DNAJC9 PE=1 SV=1
980	Q92878|RAD!	121,3185	0,91	0,46	0	0	0,46	2				125992,35	1	1			2		153892,17	DNA repair protein RAD50 OS=Homo sapiens OX=9606 GN=RAD50 PE=1 SV=1
75	P63267|ACTH	417,4441	35,64	32,45	9,57	35,64	29,79	21			1,07E+07	672794,6	1	34	7	119	42	Oxidation	41876,87	Actin, gamma-enteric smooth muscle OS=Homo sapiens OX=9606 GN=ACTG2 PE=1 SV=1
848	Q9HCU4|CELI	45,57737	0,21	0,21	0,21	0	0	1	259197,53	127323,11			1	1	1				317452,5	Cadherin EGF LAG seven-pass G-type receptor 2 OS=Homo sapiens OX=9606 GN=CELSR2 PE=1 SV=1
637	Q03001|DYST	167,0772	0,17	0,16	0,16	0,09	0,17	3			49569,656	32313,193	1	3	3	4	6		860661,8	Dystonin OS=Homo sapiens OX=9606 GN=DST PE=1 SV=4
1309	Q04721|NOT	27,4342	0,24	0	0	0,24	0	1					1			1			265404,78	Neurogenic locus notch homolog protein 2 OS=Homo sapiens OX=9606 GN=NOTCH2 PE=1 SV=3
77	P08651|NFIC	47,90091	1,18	1,18	0	0	0	1					1	1					55674,973	Nuclear factor 1 C-type OS=Homo sapiens OX=9606 GN=NFIC PE=1 SV=2
79	P98095|FBLN	457,3338	27,11	13,77	6	11,74	26,01	26	2062147,8	433721	1216597,2	1,28E+07	26	21	11	21	62	Carbamid	126572,71	Fibulin-2 OS=Homo sapiens OX=9606 GN=FBLN2 PE=1 SV=2
787	Q9NPB6|PAR	28,86495	1,73	0	0	0	1,73	1				264093,28	1				1		37388,316	Partitioning defective 6 homolog alpha OS=Homo sapiens OX=9606 GN=PARD6A PE=1 SV=1
446	O76013|KRT3	162,4103	3,64	1,5	1,5	3,64	1,5	3					1	2	4	10	6	Acetylatic	52247,066	Keratin, type I cuticular Ha6 OS=Homo sapiens OX=9606 GN=KRT36 PE=1 SV=1
316	P02585|TNN(	115,2304	4,38	4,38	4,38	4,38	4,38	1	1716701,5	712812,94	542627,6	626409,44	1	4	3	2	3	Oxidation	18122,047	Troponin C, skeletal muscle OS=Homo sapiens OX=9606 GN=TNNC2 PE=1 SV=2
398	O95573|ACSI	30,11886	0,83	0	0	0,83	0,83	1			289892,6	353094,47	1			1	1		80420,086	Fatty acid CoA ligase Acsl3 OS=Homo sapiens OX=9606 GN=ACSL3 PE=1 SV=3
384	Q6NUP7|PP4	108,8513	0,69	0	0	0,69	0	1			1303591,6		1			2			99451,98	Serine/threonine-protein phosphatase 4 regulatory subunit 4 OS=Homo sapiens OX=9606 GN=PPP4R4 PE=1 SV=1
285	Q9H2Z4|NKX	42,64934	1,98	0	0	0	1,98	1				70901,19	1				1	Deamidat	36179,03	Homeobox protein Nkx-2.4 OS=Homo sapiens OX=9606 GN=NKX2-4 PE=1 SV=3
34	P05997|CO5A	502,9045	33,56	32,42	13,41	2,33	3,2	53	5,18E+07	1,66E+07	3241093	5359952	41	78	40	6	9	Oxidation	144910,1	Collagen alpha-2(V) chain OS=Homo sapiens OX=9606 GN=COL5A2 PE=1 SV=3
1310	Q8NBI5|S43A	42,56101	1,83	0	0	0	1,83	1					1				1		54528,844	Equilibrative nucleobase transporter 1 OS=Homo sapiens OX=9606 GN=SLC43A3 PE=1 SV=2
233	Q75LS8|FKB9	111,7402	18,31	18,31	0	0	0	3	320485,75				3	6				Carbamid	15590,586	Putative FK506-binding protein 9-like protein OS=Homo sapiens OX=9606 GN=FKBP9P1 PE=5 SV=1
299	Q96P63|SPB1	316,4458	20	2,47	0	17,53	16,05	9			1768913,5	1587637,6	8	4		15	9	Carbamid	46276,418	Serpin B12 OS=Homo sapiens OX=9606 GN=SERPINB12 PE=1 SV=1
192	P06865|HEXA	364,4728	21,93	20,6	10,4	0	0	14	1684111,4	396737,47			12	22	16				60702,832	Beta-hexosaminidase subunit alpha OS=Homo sapiens OX=9606 GN=HEXA PE=1 SV=2
1044	P16157|ANK1	40,36036	0,32	0	0	0,32	0	1			63496,605		1			1			206264,77	Ankyrin-1 OS=Homo sapiens OX=9606 GN=ANK1 PE=1 SV=3
73	Q8WY21|SOF	111,8822	0,6	0,6	0	0,6	0	1	72660,6		57984,617		1	1		1			129634,84	VPS10 domain-containing receptor SorCS1 OS=Homo sapiens OX=9606 GN=SORCS1 PE=1 SV=3
193	P40925|MDH	355,5786	35,03	35,03	8,98	0	0	14	1204741,8	26101,338			13	17	4			Carbamid	36426,12	Malate dehydrogenase, cytoplasmic OS=Homo sapiens OX=9606 GN=MDH1 PE=1 SV=4
981	P25705|ATPA	166,3745	3,44	0	0	3,44	0	2			93340,34		2			4			59750,625	ATP synthase subunit alpha, mitochondrial OS=Homo sapiens OX=9606 GN=ATP5F1A PE=1 SV=1
300	O14773|TPP1	304,5105	16,16	14,56	8,88	0	0	9	1847779,9	585566,25			9	11	9				61247,926	Tripeptidyl-peptidase 1 OS=Homo sapiens OX=9606 GN=TPP1 PE=1 SV=2
1168	P40424|PBX1	28,99176	1,63	1,63	0	0	0	1					1	2					46625,81	Pre-B-cell leukemia transcription factor 1 OS=Homo sapiens OX=9606 GN=PBX1 PE=1 SV=1
447	P10412|H14_	222,4689	21,92	5,48	0	3,2	21,92	5				63738,027	3	1		1	5		21865,266	Histone H1.4 OS=Homo sapiens OX=9606 GN=H1-4 PE=1 SV=2
131	P00338|LDHA	407,1341	51,2	51,2	41,87	26,2	28,31	21	1,19E+07	6770160	1239694,4	832918,75	15	60	59	17	11	Carbamid	36688,72	L-lactate dehydrogenase A chain OS=Homo sapiens OX=9606 GN=LDHA PE=1 SV=2
982	Q96HU8|DIR/	52,05381	9,05	0	0	5,53	3,52	2			181053,16	4992,647	2			2	1	Carbamid	22484,94	GTP-binding protein Di-Ras2 OS=Homo sapiens OX=9606 GN=DIRAS2 PE=1 SV=1
283	Q8NBF6|AVL	99,77915	0,93	0	0	0,93	0	1			26690,072		1			1		Carbamid	71947,42	Late secretory pathway protein AVL9 homolog OS=Homo sapiens OX=9606 GN=AVL9 PE=1 SV=1
249	Q9UMX0|UB(	100,8806	1,02	1,02	1,02	1,02	1,02	1	1704077,6	705186,9	3805421,8	1,56E+07	1	6	7	6	4		62518,89	Ubiquilin-1 OS=Homo sapiens OX=9606 GN=UBQLN1 PE=1 SV=2
80	Q5D862|FILA	429,4504	10,87	0,46	0,42	9,79	7,28	23	26379,58	40201,84	8216587	6813494	23	1	1	44	35	Deamidat	248072,58	Filaggrin-2 OS=Homo sapiens OX=9606 GN=FLG2 PE=1 SV=1
56	Q96LP2|FA81	80,91282	1,33	0	0	1,33	1,33	1			1044845,06	7799888	1			2	4		52022,453	Protein FAM81B OS=Homo sapiens OX=9606 GN=FAM81B PE=1 SV=4
61	P01024|CO3_	473,7055	21,95	19	11,18	0	0,54	39	6009136,5	1491809,4		70890,69	37	52	35		1	Carbamid	187148,06	Complement C3 OS=Homo sapiens OX=9606 GN=C3 PE=1 SV=2
150	Q9NWW0|HI	45,67938	4,35	0	4,35	0	0	1					1		1				15290,625	Host cell factor C1 regulator 1 OS=Homo sapiens OX=9606 GN=HCFC1R1 PE=1 SV=1
600	Q99797|MIPE	25,82969	0,98	0,98	0	0	0	1					1	1					80640,73	Mitochondrial intermediate peptidase OS=Homo sapiens OX=9606 GN=MIPEP PE=1 SV=2
421	Q53F39|MPP	46,69381	1,52	1,52	0	0	0	1	31704,02				1	1					45141,02	Metallophosphoesterase 1 OS=Homo sapiens OX=9606 GN=MPPE1 PE=1 SV=2
248	P16070|CD44	329,9312	13,07	1,08	1,08	11,46	11,86	9	170973,89	392798,66	1422933	9447887	9	1	1	19	30	Carbamid	81537,62	CD44 antigen OS=Homo sapiens OX=9606 GN=CD44 PE=1 SV=3
149	P25940|CO5A	328,1773	6,88	5,33	1,66	1,03	0,34	18	3831860				5	19	9	3	1		172120,78	Collagen alpha-3(V) chain OS=Homo sapiens OX=9606 GN=COL5A3 PE=1 SV=3
1034	A6NCE7|MP3	34,97028	5,6	0	0	0	5,6	1				227544,36	1				1		14627,986	Microtubule-associated proteins 1A/1B light chain 3 beta 2 OS=Homo sapiens OX=9606 GN=MAP1LC3B2 PE=1 SV=1
638	Q05707|COE/	141,7608	1,45	1	0	0	0,45	3	14484,233				2	3			1		193515,47	Collagen alpha-1(XIV) chain OS=Homo sapiens OX=9606 GN=COL14A1 PE=1 SV=3
17	Q6IWH7|ANC	71,92906	0,64	0,64	0,64	0,64	0,64	1	5179777	1402151,8	421829,3	3694553	1	5	3	2	4		105531,54	Anoctamin-7 OS=Homo sapiens OX=9606 GN=ANO7 PE=1 SV=2
635	Q9BQ04|RBN	35,40345	1,67	0	0	1,67	1,67	1			35780,35	203642,52	1			1	1		40149,62	RNA-binding protein 4B OS=Homo sapiens OX=9606 GN=RBM4B PE=1 SV=1
1311	Q8TDY4|ASA	28,97654	0,66	0	0,66	0	0	1		134069,75			1		1				99155,42	Arf-GAP with SH3 domain, ANK repeat and PH domain-containing protein 3 OS=Homo sapiens OX=9606 GN=ASAP3 PE=1 SV=1
83	O14638|ENPI	38,47108	0,8	0	0,8	0	0	1		57077,92			1		1				100124,33	Ectonucleotide pyrophosphatase/phosphodiesterase family member 3 OS=Homo sapiens OX=9606 GN=ENPP3 PE=1 SV=2
639	P26373|RL13_	61,94634	10,43	0	2,84	4,27	3,32	3				39475,867	3		1	1	1		24261,473	60S ribosomal protein L13 OS=Homo sapiens OX=9606 GN=RPL13 PE=1 SV=4
369	Q15758|AAA	275,3175	12,57	0	0	7,76	8,87	7			316993,34	1490401,6	6			3	8		56598,336	Neutral amino acid transporter B(0) OS=Homo sapiens OX=9606 GN=SLC1A5 PE=1 SV=2
221	O00584|RNT2	117,7638	2,34	0	0	2,34	2,34	1			190715,88	224205,34	1			2	1		29480,803	Ribonuclease T2 OS=Homo sapiens OX=9606 GN=RNASET2 PE=1 SV=2
518	P48509|CD15	191,0611	12,25	0	0	8,7	12,25	4			371560,38	3625586	4			3	4	Carbamid	28295,102	CD151 antigen OS=Homo sapiens OX=9606 GN=CD151 PE=1 SV=3
725	Q96DA0|ZG1(	25,7136	2,88	0	0	0	2,88	1				1,48E+07	1				2	Acetylatic	22739,027	Zymogen granule protein 16 homolog B OS=Homo sapiens OX=9606 GN=ZG16B PE=1 SV=3

Note. Accession, Uniprot accession; -10lgP, MS Score; Coverage (%), Sequence coverage of the protein by identified peptides; Area Sample, the sum of areas of identified unique peptides; #Peptides, the number of identified peptides; #Unique, the number of identified unique peptides.

Table S3. Intersecting and unique proteins for all investigated samples.

sHTSF /sNDFs (329)			sHTSFs/sNDFs/vHTSFs/vNDFs (319)			vNDFs (295)			sHTSFs (209)		sHTSFs/sNDFs/vNDFs (188)		vHTSFs (101)
Q9P107	P01034	Q9GZV1	P30101	P14209	P61981	Q5ZPR3	Q8NB49	Q8ND94	Q15631	Q9UL03	P03956	P53621	P0DOX5
Q9HBG7	P63279	P19021	P00736	P32119	Q5T4S7	Q9BQJ4	P20645	P61106	Q9BTX7	O15145	P28072	P30502	O76081
P06865	Q9GZX9	P48723	Q99877	Q17RW2	P25786	Q9H611	Q9Y2I6	Q969E2	O00443	Q13867	P41252	P28066	P47929
P05155	Q9P232	O76076	P13639	Q13885	Q13162	Q9Y6G9	Q15039	Q86YB2	Q96IY6	Q9UNZ2	P05388	Q9NQC7	Q96QR8
Q86U42	P43652	Q06481	Q9P2P6	P13987	P13611	P14868	Q99698	Q92633	Q9P2V4	Q13361	P50990	Q3BBV0	O60716
P05120	Q07065	Q5VT97	P80723	Q5XKE5	P12111	P49327	O60701	Q8WAS	P49802	P09429	Q9P281	P07942	Q00613
Q15011	Q96AY3	P29279	P68431	P63261	P07437	P12821	Q9BZG1	Q13488	P16870	Q15436	Q5TAG4	Q8IZT6	Q08828
P01008	P41271	Q01105	P06899	Q09666	Q9H299	O60674	Q9Y4F1	Q9UKS6	O43583	Q9NR12	Q16181	P22314	P84157
P16112	P16278	O43505	Q07954	P07339	P01889	Q12986	P15954	Q9P0L0	Q15365	P19367	Q14203	Q7Z406	Q9NSB4
Q9P225	Q8NBP7	P53634	Q14315	Q99613	P58107	Q8WWI5	Q8IZ83	P21926	Q8TD17	P98088	Q5TI25	Q12884	P52738
P40926	Q13442	Q15262	Q08378	Q7Z7G0	P04075	P60033	Q8WV41	Q96EY5	O95084	Q8TF46	P49746	P31150	P03973
Q9HC35	Q9H254	Q12765	Q8IUX7	Q04695	P0DP25	Q9HBH7	Q96MM6	Q0D2K0	Q16543	Q09028	Q6GPI1	Q3BBV2	Q96NJ6
Q8IZ73	Q7Z4V5	Q06828	P02751	Q14993	P02533	O95297	O73689	P20340	Q9UII2	P06307	O43151	P30454	Q8NCT1
P17174	O00391	P40925	P08123	Q99536	P02458	Q99732	Q9H444	Q9NX76	O00148	Q9BXR6	Q6P3W6	P01024	Q86WR7
O75326	O00585	O00584	P04239	P07737	O95678	Q99832	O00139	P15559	Q8NDH2	P29622	Q8N660	P30740	Q8NF86
Q6UX15	O95881	Q9Y646	Q9BTM1	P23527	Q6BMK4	Q9H2V7	Q9BTU6	Q9Y334	Q9NY46	Q9NYQ8	Q8NHW5	Q9BUF5	Q96C24
[illegible]	[illegible]	[illegible]	[illegible]	[illegible]	[illegible]	[illegible]	[illegible]	[illegible]	[illegible]	[illegible]	[illegible]	[illegible]	[illegible]

sNDFs (88)	vHTSFs/vNDFs (81)	sHTSFs/vNDFs (63)	sHTSFs/sNDFs/vHTSFs (51)	sNDFs/vNDFs (28)	sHTSFs/vHTSFs/vNDFs (22)	sNDFs/vHTSFs (20)	sNDFs/vHTSFs/vNDFs (14)	sHTSFs/vHTSFs (11)
Q92847	Q99816	Q14108	P09651	P49257	Q01082	Q08345	P02792	Q9UKN7
Q16706	O43657	Q9Y3Y2	P20929	Q8N122	Q8IVF2	Q6UWP8	P08195	Q14696
O43292	O43491	P18084	P17096	P61247	Q08431	P10828	O15118	O00468
Q52LW3	P01876	Q13813	Q01130	Q14839	P08133	Q2LD37	Q8NEY1	O60285
O43570	Q9C0H2	O75787	Q9UJJ9	P29375	Q9NYU2	P14923	P04040	Q8WXG1
Q16560	P51148	P78371	P51991	Q96DN6	P08962	P0CG04	Q9UFH2	P24593
Q8IW41	Q9P215	P16144	Q8WWM1	P09619	P35613	O15078	Q96A84	P21741
Q15782	P51798	P51587	Q14766	Q96EB6	Q5IPF3	Q86TJ2	Q15751	Q6KB66
Q96NN9	Q9UNM6	Q8WUJ3	P02760	Q9HCL0	Q9ULL5	B9A064	Q08554	P34741
Q9UK76	P51149	Q01518	P06454	Q17RM4	Q02224	P01591	P20930	P07359
P48307	Q14764	Q14254	Q9H1E3	Q9H6X2	Q9Y315	Q5D862	P51572	P05154
Q9ULL4	P61626	Q9HBG6	Q5BKZ1	Q7Z5B4	P31025	A6NNM8	Q5T749	
O14795	P0DPK2	Q9H4G4	Q92673	P15880	O75151	Q5VYK3	O43237	
Q6IQ26	O95166	Q04446	P28300	Q6ZUX3	Q9H3U5	P0DOX8	Q9NZT1	
Q8TF76	Q15468	O43504	Q96RW7	Q96QD6	P35749	Q01094		
Q2TAM9	Q03113	Q99829	Q12805	A0A8I5KQE6	P62269	Q3SW79		
Q9NW08	Q53TN4	P43246	O95965	Q9H0X4	Q13616	Q07955		
Q6UXH1	Q9P282	O15530	P20933	O00203	P78527	Q9BR76		
A6NI15	Q9HBL0	O43399	P20962	P52333	P42892	P10827		
P43234	Q04941	Q06278	P09486	Q8N3D4	A7E2Y1	Q02413		
P49908	Q8WUM4	Q86V81	P61916	P27708	A6QL64			
Q12792	P61586	P49588	Q9Y256	Q96M63	P15502			
Q6ZUT9	Q15836	P00387	P02771	Q4ADV7				
O75443	P23396	O15230	P17936	Q9UEU0				
B7ZW38	Q9UPA5	P17655	Q16270	Q6EDU4				
Q7Z7L7	Q9NVM1	P61026	O60437	P06213				
Q8N883	Q9UGM3	O00299	P05089	P08865				
Q53TS9	O14495	P46777	P35318	Q6V0I7				
Q96DB5	P0DPK5	Q8TF66	P08294					
Q9BZI1	Q9Y2J2	Q14011	P29308					
P15151	P61224	A1L4H1	Q16610					
Q9NVC3	Q9NZM1	O75083	O75368					
P21246	Q96TA1	P62829	P01009					
P07910	O60637	Q13200	Q9NQ87					
Q9H6L4	Q8TAD7	P16234	P41222					
Q13342	P05556	O00231	O60888					
P98082	P46976	P13489	Q14247					
Q8N4K5	Q13641	P51153	Q8TAT2					
P10588	Q9NUM4	Q8NEG5	Q96CG8					
Q13591	P52824	P61970	Q8N9Z2					
P0DMR1	P08473	Q07021	Q9NP84					
Q86Y26	Q3KNW1	P05543	Q9Y217					
A4D1E9	P08754	Q4I6C6	P15586					
P42167	Q9NVJ2	P62333	P02545					
O75398	P04899	P26232	Q6TDU7					
Q8NGB8	P23634	P25686	Q05682					
Q96L15	P01877	P62820	O00115					
Q96P15	Q9H8M9	Q8N8U9	P20810					
Q6PGQ1	Q8N357	P52907	P07108					
P49736	P62879	P30511	Q15942					
O60812	P62263	Q9H0U4	Q9UHL4					
P51397	Q14344	P30050						
Q16629	P0DOY2	Q9UHQ4						
Q01844	Q9HAV0	Q9C093						
Q8TER0	Q15771	Q15286						
P40879	Q5VW32	P46940						
P03950	P25311	P15121						
B2RXH8	P56539	Q8N1P7						
Q9Y2H5	P04216	Q9NZN4						
Q8NGI9	Q03135	P53396						
Q9H7M6	O00560	P49390						
Q70CQ4	Q15758	P17813						
P21781	P0DOY3	O95782						
Q9NWM0	Q14204							
Q8N3K9	Q9UL33							
Q03519	P60520							
Q8IXL6	P31944							
Q9UHB6	P48681							
Q9NUG4	P62873							
Q9UM22	A0A494C071							
Q8IVV2	Q92485							
O00763	Q14C86							
P35368	P62834							
Q15050	P48509							
P48742	P53801							
P16066	O15400							
Q9H313	Q9H0R8							
Q9Y5Y6	P17600							
Q9UBK8	P17302							
Q711Q0	P27105							
P14324	P01040							
P61086								
P00742								
Q6UWV2								
P42166								
P28330								
Q53HL2								
Q03167								

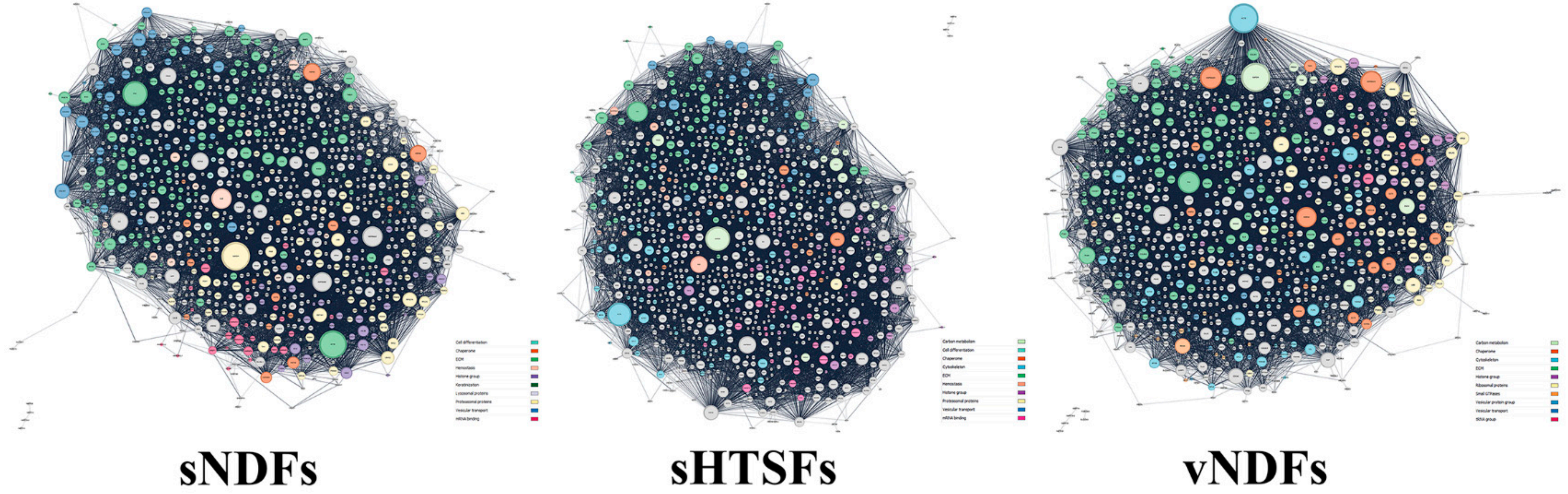

Figure S1. Size and color-coded interaction network for sNDFs, sHTSFs and vNDFs samples.

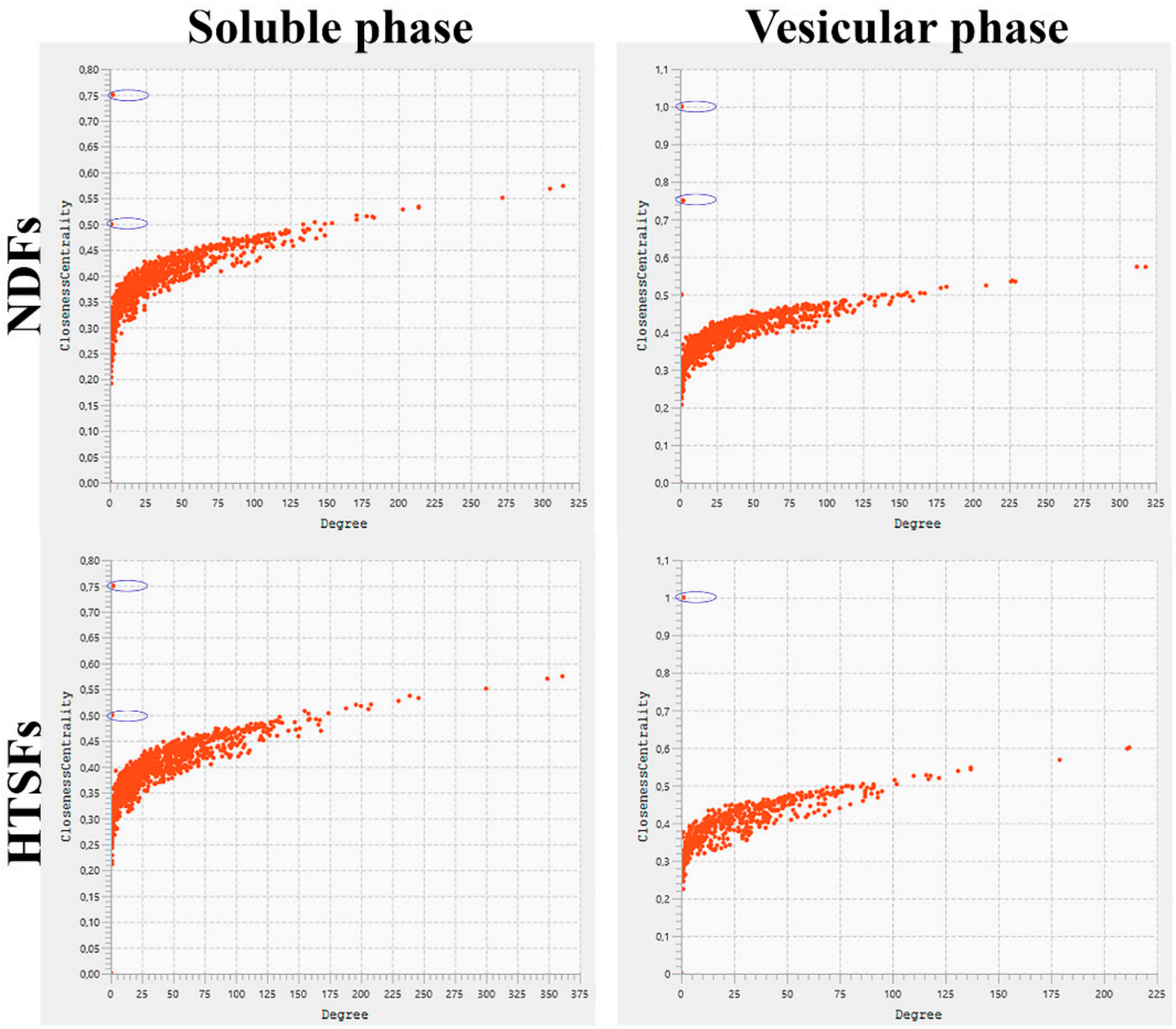

Figure S2. Centrality-degree scatter plot for all nodes in the investigated networks. The ellipses indicate high centrality nodes.

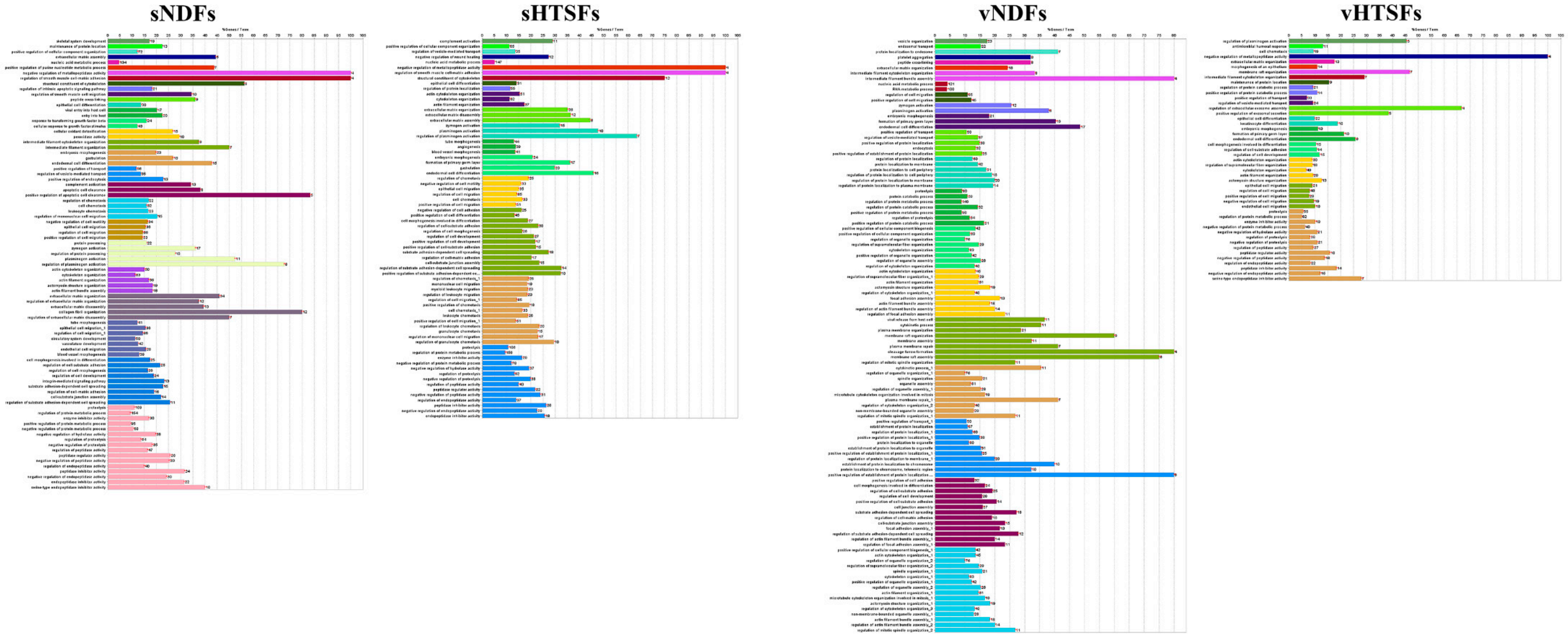

Figure S3. Stack graph for GO biological process terms in the investigated networks.
